# Primate cognition in zoos: Reviewing the impact of zoo‐based research over 15 years

**DOI:** 10.1002/ajp.23369

**Published:** 2022-03-14

**Authors:** Emma S. McEwen, Elizabeth Warren, Sadie Tenpas, Benjamin Jones, Kresimir Durdevic, Emilie Rapport Munro, Josep Call

**Affiliations:** ^1^ School of Psychology and Neuroscience University of St Andrews St Andrews UK

**Keywords:** methodology, primate cognition, zoo research

## Abstract

Primate cognition research is reliant on access to members of the study sp ecies and logistical infrastructures to conduct observations and experiments. Historically founded in research centers and private collections, and spreading to modern zoos, sanctuaries, and the field, primate cognition has been investigated in diverse settings, each with benefits and challenges. In our systematic review of 12 primatology, animal behavior, and animal cognition journals over the last 15 years, we turn a spotlight on zoos to quantify their current impact on the field and to highlight their potential as robust contributors to future work. To put zoo‐based research in context, we compare zoos to three other site types: university‐owned or independent research centers, sanctuaries, and field sites. We assess the contributions of zoos across several critical considerations in primate cognition research, including number of investigations, species diversity, sample size, research topic diversity, and methodology. We identified 1119 publications reporting studies of primate cognition, almost 25% of which report research conducted in zoos. Across publications, zoo‐based research has greater species diversity than research centers and covers a diverse range of research topics. Although our review is merely a snapshot of primate cognition research, our findings suggest that zoos may present advantages to researchers regarding species diversity, and lack some of the methodological constraints of field sites, allowing greater ease of access to a diverse range of subjects for cognition investigations. We suggest that zoos have great potential as key contributors for future investigations in primate cognition. Finally, we shed light on the symbiotic relationship that can emerge between researchers and zoos, forming partnerships that bring unique advantages to both parties.

## Highlights

- Zoo‐based research contributes ~25% of publications in primate cognition
- Zoos offer high species diversity in cognitive research
- Research in zoos encompasses a wide and balanced array of topics within primate cognition

## Graphical abstract

Locations of all sites indentified in this study as locations of primate cognition research. Site type is indicated by color, point size indicates the number of studies from each site.

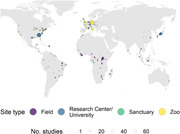

**Abbreviations**
ABC: Animal Behavior and Cognition
AJP: American Journal of Primatology
ANB: Animal Behavior
ANC: Animal Cognition
BEH: Behavior
BEP: Behavioral Processes
CNR: Institute of the Science and Technology of Cognition, Consiglio Nazionale delle Ricerche/National Research Council
CRC/Antwerp University: Centre for Research and Conservation/Antwerp University
ETH: Ethology
FOP: Folia Primatologica
IJP: International Journal of Primatology
JCP: Journal of Comparative Psychology
JEP: ALC: Journal of Experimental Psychology: Animal Learning and Cognition
PRM: Primates

## 1 INTRODUCTION

The scientific study of nonhuman primate (hereafter, primate) cognition originated in psychological laboratories at the turn of the 20th century (Kinnaman, 1902; Thorndike, 1901). Within the same decade, zoo‐based research began to contribute to the discipline as well. For instance, Haggerty (1909, 1913) conducted early research on imitation in capuchin monkeys and problem solving in orangutans at the New York Zoological Park. A little later, cognitive studies were conducted with several species of primates at Vilas Park Zoological Gardens and Bronx Park Zoo (e.g., Harlow et al., 1932; Maslow & Harlow, 1932). Yerkes, one of the founding innovators in primate cognition, began his studies on primate learning and problem solving in private primate collections (Yerkes, 1916, 1925, 1927), considered by many to be the precursors of modern zoos.

Despite its dual origin, the subsequent expansion and consolidation of the discipline of primate cognition took place mainly in research centers and university laboratories around the world, some of which were specifically devoted to the study of primate cognition and behavior (Köhler, 1925; Yerkes, 1943; and see Frisch, 1959 for an overview of the Japan Monkey Center). Nevertheless, zoo‐based research continued to sporadically contribute to the discipline, often with studies focused on replications and extensions of classical problem‐solving tasks. For instance, inspired by Köhler's work, Guillaume and Meyerson (1930; for review, see Guillaume & Meyerson,^1987^) conducted an extensive series of studies on problem‐solving in several species of monkeys and apes at the menagerie of the Natural History Museum in Paris, as well as at the Pasteur Institute research center.

Once primate cognition research in laboratories became firmly established, some researchers turned their attention towards field studies to complement the laboratory work. In fact, Yerkes was responsible for sending several of his associates, including Nissen, Bingham, and Carpenter on field expeditions to investigate the behavior of chimpanzees, gorillas, and howler monkeys, respectively (Bingham, 1932; Carpenter, 1964; Nissen, 1932). As the value of field studies was realized, pioneers such as Goodall, Itani, Imanishi, and Kummer established long‐term field sites and research programs, independent of the work in laboratories, in habitat countries of several primate species (Goodall, 1986; Imanishi, 1957; Itani, 1963; Kummer, 1982). Throughout the 20th century, field primatology also developed across several sites in India (for a review, see Singh et al., 2020) and South America (for a review, see Strier & Mendes, 2009). Initially, work in these field sites focused on behavior, but the 1980s saw a gradual introduction of investigations into primate cognition (e.g., Boesch & Boesch, 1984; Cheney & Seyfarth, 1982; see also Cheney & Seyfarth, 2018).

In the last few decades, modern zoos have become a valuable and steady contributor to primate cognition research. For instance, Kitahara‐Frisch and colleagues conducted a series of studies on toolmaking at Tama Zoo in Tokyo (Kitahara‐Frisch, 1987; Kitahara‐Frisch & Norikoshi, 1982). Although this study is reminiscent of the classical work on problem‐solving, a key innovation is that they used the data from field studies as the inspiration for their work. More specifically, Kitahara‐Frisch and Norikoshi (1982) investigated the spontaneous invention of leaf‐sponging for water by captive chimpanzees. Other researchers initiated zoo‐based programs that built upon some of the early work in primate cognition, but also brought new ideas and connected with other disciplines, such as developmental psychology. Antinucci (1989), for instance, conducted a series of Piagetian studies with capuchin monkeys, macaques, and a gorilla housed at the Rome Zoo (Bioparco). This study was made possible by the collaboration between the Institute of the Science and Technology of Cognition, CNR, and Rome Zoo, started in 1984 and continuing to this day; the earliest and one of the most successful collaborations of its kind. Zoo‐based research programs continued to emerge throughout the late 20th century, and laid the groundwork for the proliferation of primate cognition research found in zoos today.

Perhaps driven by a decline in funding for research centers and university‐based laboratories, and a related decrease in the number of species and number of animals available in those facilities, more researchers have turned to alternative sites to investigate questions of cognition (although this may not be the case for all areas; e.g., cognitive studies involving neuroscience are likely to take place in research centers). Zoos embrace and promote biodiversity, and some have become more research‐oriented and more willing to embark on long‐term research programs outside of welfare, conservation, and veterinary medicine (see Stoinski et al., 1998, for an example across North American zoos). This has offered suitable conditions for the emergence of several models for studying primate cognition.

Some zoos have created their own research programs, such as the Think Tank at the Smithsonian National Zoological Park in Washington DC, and the Lester E. Fisher Center at Lincoln Park Zoo, Chicago (Bielick & Doering, 1997; Ross, 2016). Other zoos have established long‐term collaborations with research institutions and universities to carry out research (e.g., Burgers Zoo and Utrecht University; Zoo Atlanta and Georgia State University & Emory University; Antwerp Zoo & Planckendael Park and CRC/Antwerp University) (Shumaker, 2018). A third group of zoos has forged long‐term collaborations in which research institutes and universities have made substantial capital investments to create new infrastructure and absorb some of the personnel costs associated with the research programs (e.g., Zoo Leipzig, Wolfgang Köhler Primate Research Centre; Edinburgh Zoo, Living Links Centre and Budongo Research Unit; Furivik Zoo, Lund University Primate Research Station; Rome Zoo, Institute of the Science and Technology of Cognition, CNR).

These developments, some of them relatively recent, suggest that zoos can play an important role in the study of primate cognition. To fully understand the potential for zoo‐based research in this field, we must ask: what is the real impact that zoos have in the discipline of primate cognition? What lessons can we learn that could enhance zoos' contribution to the study of primate cognition in the future?

To answer these questions, we conducted a systematic review of animal behavior, animal cognition, and primatological literature and extracted all articles reporting empirical research on primate cognition. For each primate cognition study, we scored the species investigated (including its sample size), the topic, the location where the research took place, and the methods used. The purpose of this review was to estimate the magnitude of the contribution of cognitive research done in zoos relative to three other location types: field, research center/university laboratories, and sanctuaries (see Cronin et al., 2017, for a similar site‐type comparison). While zoo‐based research encompasses areas of study other than cognition, such as behavior and welfare, these were beyond the scope of this study. Previous work has thoroughly reviewed the species involved and methods used in cognitive research in zoos (Hopper, 2017), the range of research in primate sanctuaries (Ross & Leinwand, 2020), and the extent of primate cognition research using touchscreens in zoos (Egelkamp & Ross, 2019). Here, we compare the number of articles published, the diversity of species investigated, and the level of technological sophistication required by each investigation, to place the impact of zoo‐based primate cognition research in context with other site types. Finally, we explore whether any of those indicators have changed over the 15 years included in our review.

## 2 METHOD

### 2.1 Journals

**Figure 1:**
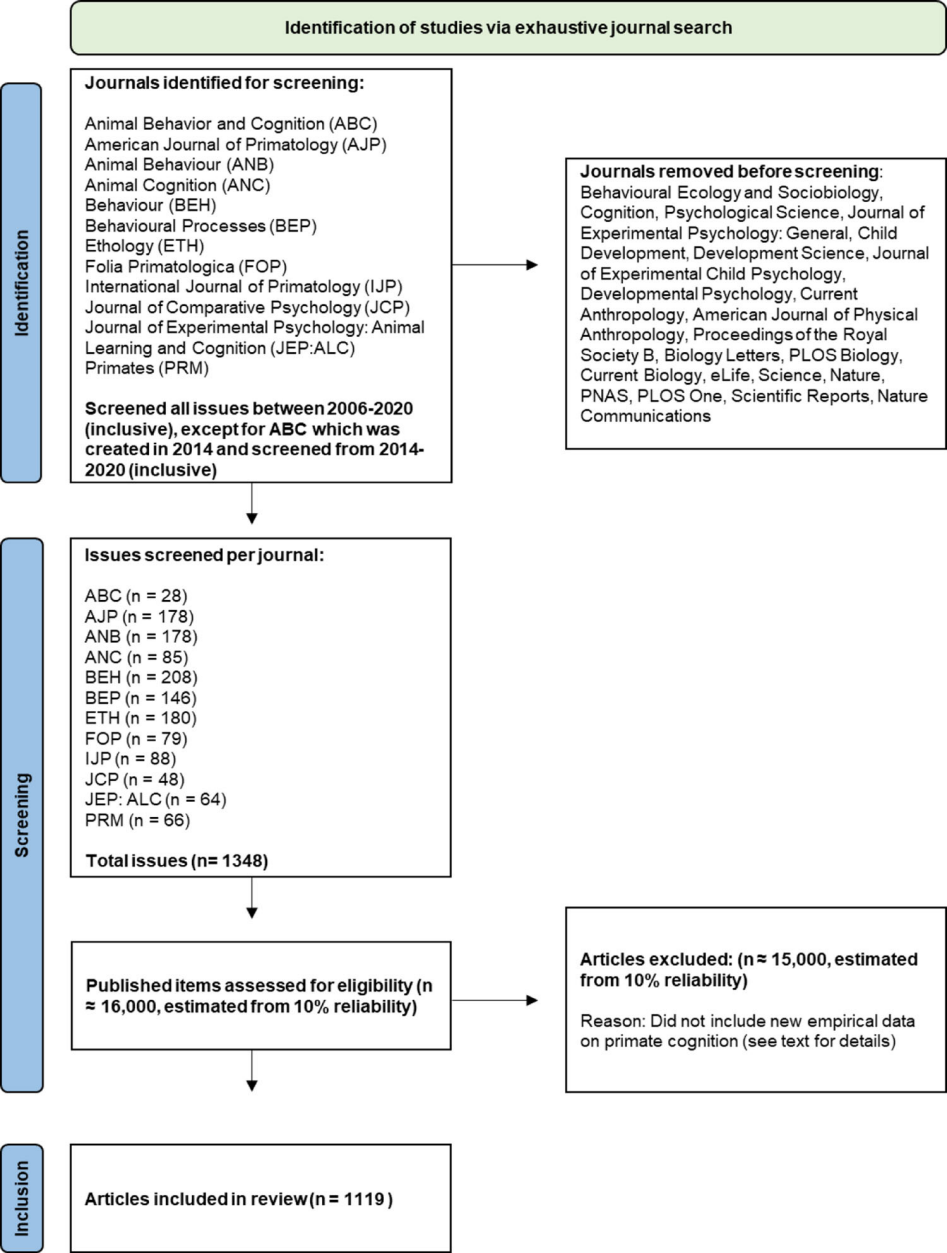
Flow chart outlining the review process. See supporting information for details of how estimates were obtained. Adapted PRISMA flow diagram (Page et al., 2021)

We conducted a systematic review of primate cognition articles from 12 journals (see Table 2) published between 2006 and 2020 (note that Animal Behavior and Cognition was founded in 2014 and has no publications before that year). The journals were selected from an initial pool of 30 journals publishing primatology, animal behavior, animal cognition, developmental science, physical anthropology research, and general research. Due to time constraints, and to ensure we could cover 15 years, reviewing every paper in every journal targeted, we reduced the number of areas. We narrowed our sample to include four journals each from the three disciplines most likely to publish primate cognition work: primatology, animal behavior, and animal cognition (see Figure 1 for an overview of our screening process).

**Table 1:** List of reviewed journals organized by topic

Journal type	Journal names
Primatology	American Journal of Primatology, International Journal of Primatology, Primates, Folia Primatologica
Animal cognition	Journal of Comparative Psychology, Journal of Experimental Psychology: Animal Learning and Cognition, Animal Cognition, Animal Behavior and Cognition
Animal behavior	Animal Behaviour, Ethology, Behaviour, Behavioural Processes

**Table 2:** Overview of coding criteria

Category	Items	Definition
Topic	Space and objects	Navigation and travel, spatial memory, searching for hidden objects, tracking object displacements, object individuation, spatio‐temporal inference, mental rotation of object orientation, detours and mazes, target prediction, and relational spatial mapping. For example, Noser and Byrne (2014).
	Tools and causality	Object manipulation, tool‐use, tool properties, tool‐making, tool‐use and problem solving, causal reasoning, means‐ends reasoning, causality. For example, Völter and Call (2014).
	Features and categories	Discrimination learning, cross‐modal matching, natural categorization, relational categories, identity, oddity, analogies, and classification. For example, Zakrzewski et al. (2018).
	Quantities and time	Numerosity, ordinality, transitivity, counting, summation, multiplication, Piagetian conservation, proportions, probabilities, timing and discounting, and irrational choices. For example, Broihanne et al. (2019).
	Social knowledge and interaction	Knowledge of social relationships, prosociality and helping, reciprocity and interchange, social comparison, inequity aversion, coordination and collaboration, behavioral economics. For example, Xia et al. (2013).
	Social strategies and communication	Deception, social tool‐use, vocal communication, gestural communication, ape language, inter‐specific communication with humans. For example, Coye et al. (2018).
	Social learning and culture	Social learning mechanisms, traditions, teaching, social learning strategies, social transmission, and culture. For example, E. Price and Caldwell (2007).
	Theory of mind and metacognition	Knowledge of psychological states of perception and attention, goals and intentions, knowledge and beliefs, self‐knowledge, meta‐memory, information seeking For example, Mulcahy (2016).
Apparatus	No apparatus	No apparatus was used to facilitate data collection. Enclosure features and platforms to present food were not considered apparatuses. This may also include a behavioral demonstration from an experimenter, with no props. For example, Jaeggi et al. (2010).
	Experimenter props	Experimenter manipulation of physical items visible to the subject, but not touched or manipulated by the subject. For example, Drayton et al. (2016).
	Speaker	Presentation of audio stimuli. For example, Matthews and Snowdon (2011).
	Picture	Presentation of nondigital visual stimuli (such as a photograph). For example, Boggiani et al. (2018).
	Monitor	Presentation of visual stimuli on a computerized screen display. For example, Gao and Tomonaga (2020).
	Object options	Apparatus involving two or more items between which the subject must make a selection (or a single item in a go/no‐go task). Selections could be made by behaviors such as gazing, pointing, approaching, and reaching. Importantly, the subject's actions toward the apparatus serve only to make a selection. For example, Pladevall et al. (2020).
	Tokens	Apparatus involving items which the subjects could exchange between conspecifics and/or experimenters.
	Tools and trays	Apparatus involving physical items with which the subject could retrieve or process rewards, either by themselves or by manipulating another object. Additionally, this definition included pulling trays, which the subject could manipulate to pull a reward within an obtainable distance. For example, Judge and Bruno (2012).
	Puzzle	Apparatus involving a physical item that the subject manipulates to learn about the apparatus itself, such as how to retrieve a reward. This definition included large mazes/arrays, through which the subject could move to receive a reward. For example, Dean et al. (2011).
Recording materials	Computer	Any computerized or digital means of automatically recording subject responses (e.g., a computerized button which records the number and timing of presses). For example, Bigelow and Poremba (2013).
	Touchscreen	A means of interacting with a monitor, or other digital display, through direct touching of the display itself or via a technological feature which records touches. For example, Marsh et al. (2013).
	Eye‐tracking	Specialized eye‐tracking software which records the light reflection from the retinas in real time, to determine the orientation of the subject's eyes. For example, Kano et al. (2012).
	Joystick	A means of interacting with a monitor or other digital display through the use of a motorized joystick. For example, Beran et al. (2015).
	Audio recording	Equipment to record the auditory signals or behavior of the subjects, such as a microphone. For example, Wheeler and Hammerschmidt (2013).
	GPS tracking	Any equipment used to determine or track the geographical location of subjects. For example, Shaffer (2014).
Site Type	Research Center/University	Primates were housed in a private or university‐affiliated research center, laboratory, or collection.
	Zoo	Primates were housed in a zoo, including research centers stationed within zoos.
	Sanctuary	Primates were housed at a sanctuary, defined as a facility generally having a rehabilitation focus where the animals would not naturally be living and, in some cases, can be semi‐free ranging.
	Field	Data were collected at a field site, defined as a location where animals are free ranging in their natural or rewilded habitats, including sites with tourist access and food provisioning.

**Table 3:** Overview of technological sophistication scoring system

	No computerized element	Computerized element
No direct interaction (passive)	1	2
Direct interaction (active)	3	4

### 2.2 Articles

Articles were subject to inclusion or exclusion based on three key criteria. First, we evaluated the study species of the article, and included it if at least one of the investigated species was a nonhuman primate species (i.e., from the order Primates, excluding hominids). Second, we evaluated the research topic, and included articles if the main investigation focused on cognition, defined here as the investigation of any ability or mechanism falling within one of the topics covered in an adapted version of Primate Cognition (Tomasello & Call, 1997; see Table 3 for details). This criterion excluded studies in which cognition was a component or a task requirement, but the main question of the investigation focused on other elements of primate behavior, such as behavioral descriptions, neuroscience, perception, personality, emotion, handedness, welfare, or medicine (see supporting information for more information). While cognition plays a supporting role in many of these research areas, we only included studies in which cognition was the primary target of the investigation. Last, we only included articles containing new research or data on primate cognition—review articles, meta‐analyses, and articles presenting re‐analyzed data without the inclusion of new data were omitted (see Figure 1 for an overview of our screening process).

We conducted a 2‐step fully systematic review of each journal, evaluating every article from every issue for the time period listed, first for inclusion (step 1), and then, if included, for data extraction (step 2). We did not use any search terms, filters, or external databases to facilitate this process, to avoid search biases and to capture the most accurate possible picture. Reviewers determined the study species, topic, and nature of each study through a combination of article titles, abstracts, main texts of the papers, and, in some cases, supporting information. We processed 12 journals over 15 years, resulting in a list of 1119 primate cognition articles included in our final data set. Details of the development of the coding scheme can be found in the supporting information. A full list of references of articles included in this data set can be found in the supporting information.

### 2.3 Data

Each reviewer (Emma S. McEwen, Elizabeth Warren, Sadie Tenpas, Kresimir Durdevic, Benjamin Jones, and Emilie Rapport Munro) coded the data from each article they selected for inclusion. We extracted certain variables, including author name(s), journal, year, reference, abstract text, species, study site name, and *site type* (field, research center/university, sanctuary, zoo), directly from the text of the article, including supporting information. In instances where this information was not provided in the text of the study, we filled in the information based on inferences made from the text, where possible. These inferences included the names of institutions listed in the statement of ethics or the acknowledgments, with respect to care for the primates or logistical facilitation of the research. We inferred that these locations were the names of the study site if the location listed was known to the reviewers as one that houses primates, for example, from information in other papers included in the review. In some cases, we were able to infer the study site name and *site type* from the names of the study subjects, based on information in other papers included in the review, or similarly, by following references to other papers which detailed the housing conditions and experimental history of the subjects. Where we could not determine the name of the study site, we could occasionally still infer the *site type* from language within the text, for example, “subjects were housed in the laboratory.” Notably, we did not use data such as author affiliation, apparatus, research topic, and other methodological information to make these inferences.

We extracted certain other variables such as *sample size* and *method* from the text of each paper, with some inference. We recorded the total number of subjects for each study as it was listed, if given in the text of the paper. In some studies, particularly observational studies, no specific number of subjects was listed due to the nature of the observations, the changeable size and composition of wild primate groups, or the lack of identifying information about the subjects at some sites. In these cases, we recorded the overall group size, if provided (but did not include these in analyses) and recorded the *sample size* as unavailable. We recorded *method* (observational or experimental) according to a criterion relating to experimental intervention. If the researchers intervened in the subjects' behavior in any way, including the provision of tools, apparatuses, or other materials, for subsequent observation, we classified the investigation as an experiment. Interventions that occurred as part of the normal husbandry of the animals, outside of the specific investigation (e.g., habitual food provisioning of a wild population, normal husbandry routines in a zoo, sanctuary, or laboratory), we did not consider experimental manipulations, and these did not preclude designation as an observational study.

We determined the remainder of the variables including *topic*, *apparatus*, and *recording material*, in accordance with the developed coding scheme (see Table 3 for definitions, and supporting information for details), based on information in the abstract, methods, discussion, and/or supporting information for each article. In terms of methodology, we coded the a*pparatus*, which denoted any materials used to present the stimuli to the subject, and *recording materials*, referring to any materials used to collect, monitor, or record the subject's response to the stimuli. *Recording materials* (see Table 3) did not include the use of live observational coding, or basic video recording, such as the use of a standard video camera, because its use has become ubiquitous in the last two decades, and often it is not stated whether studies have used live or video coding.

## 3 STATISTICAL ANALYSES

Only descriptive, not inferential, statistics are presented in this review. Our data set is not a sample of papers, but a population of papers published in our selected journals, as we assessed every item in every journal for each of the 15 years included. Additionally, our intention was not to generalize beyond the scope of this review, as we acknowledge that the results may differ if applied to different journals or disciplines (e.g., neither zoos nor field sites feature prominently in neuroscientific research). As such, when we describe differences or trends, these are purely descriptive, qualitative differences, and should not be interpreted as statistically significant or necessarily generalizable to all journals publishing research on primate cognition.

### 3.1 Data processing

In the case of studies covering multiple species, sites, topics, or apparatus types, we input separate entries for each and indicated these “duplicate” entries. We considered each duplicate as a separate data point only for analyses concerning the duplicated variable. For example, in an analysis concerning the number of species studied, a paper with two species under one paradigm would be represented twice, but one which included one species under two paradigms would appear only once. On the other hand, an analysis concerning the number of studies investigating a certain topic, a study with multiple species would only be represented once. Importantly, when counting the overall number of articles, these duplicate cases were also only counted once.

For 23 cases, we were unable to determine the *site type*, and so these articles were dropped for comparisons between site types. Generally, when presenting the descriptive statistics obtained in this review, we first present the absolute values, then apply corrections to these values, described below. We used R (version R‐4.0.2) for processing descriptive statistics and to create figures.

### 3.2 Location data

In some cases, when assessing the number of studies conducted in each site type, articles were counted more than once, as some studies were conducted at multiple sites. Each study was counted once per site type, that is, a study conducted in a sanctuary and a zoo was counted once for sanctuary, and once for zoo, whereas a study conducted in two zoos was counted only once. All cases for which we could determine the *site type* (this was possible in all but 23 cases) were included in all *site type* comparisons, but only studies for which we could determine the *site name* are included in summaries of individual locations.

### 3.3 Species classification

Taxonomy data followed Groves (2018). We completed analyses to the level of species and did not include subspecies data due to reporting inconsistencies. In the case of capuchins, due to a reclassification of robust capuchins from *Cebus* to *Sapajus* following Alfaro et al. (2012), we reclassified all cases in which the genus was listed as *Cebus* for affected species (*apella, flavius, cay, nigritus, robustus*, and *xanthosternos*) as the genus *Sapajus*. Further, due to inconsistent reporting, the inclusion of several hybrid subjects, and the reclassification of Sumatran orangutans (*Pongo abelii*) as a distinct species to Bornean orangutans (*Pongo pygmaeus*) (Groves, 2001), we collapsed all cases of orangutans at the level of genus (*Pongo*). In other cases where only the genus was given (genus *Sapajus*, 7 cases), or where a subject was a hybrid (*Nomascus*, 1 case, *Varecia*, 1 case) we removed the data from counts of distinct species but included those studies for all other analyses. For some comparisons, we grouped species by the following taxonomic groups: Hominidae (great apes), Hylobatidae (lesser apes), Cercopithecidae (Afro‐Eurasian monkeys [previously referred to as old world monkeys]), Ceboidea (monkeys of the Americas [previously referred to as new world monkeys]), and Prosimii (prosimians).

### 3.4 Topics, apparatus, and recording method

In some cases, one study covered multiple topics, or used multiple apparatuses or recording methods. In these cases, each topic, apparatus, and recording method was counted; thus, some studies were counted more than once in these overviews and the sums of proportions may exceed 100%.

### 3.5 Trends over time

For each year included in this review (2006–2020), we counted the number of new locations identified to give a cumulative overview. That is, for each year, we counted how many distinct locations were identified, which had not been identified in previous years. We also calculated the number of articles each year and the number of species studied each year across each site type.

### 3.6 Technological sophistication

To quantitatively summarize the specific and technological requirements of apparatuses and recording methods used in each site type, one reviewer (Emma S. McEwen) scored each study on an ordinal scale of “technological sophistication,” based on the subjects' interaction with the apparatus (passive/active) and whether any aspect was computerized or not (see Table 1). Scores increase in technological sophistication: a higher score indicates more specialized or sophisticated apparatuses and recording devices, whereas low scores indicate that the study could be run with less sophisticated equipment. We used the following scoring system:

0 = No apparatus.

1 = Subjects do not interact directly with the apparatus, and the apparatus is noncomputerized (includes experimenter props, picture).

2 = Subjects do not need to interact with the apparatus, but the apparatus and/or recording device is computerized (includes speaker, monitor, eye‐tracking, audio recording, GPS). Also includes object options, whether computerized or not.

3 = Subjects interact directly with the apparatus (there are specific requirements), but the equipment is not computerized (includes tokens, tools and trays, puzzle).

4 = Subjects interact directly with the apparatus (there are specific requirements) and there is some computerized element (includes touchscreen, joystick, puzzle with computerized recording device).

For each article, we calculated the mean technological sophistication score for each location type to provide a grand mean (and SD) technological sophistication score for each site type across all articles. For example, if one article conducted at a research center/university contained two separate studies (e.g., using different methodology for different groups of primates), with technological sophistication scores of 0 and 2, the mean of 1 was submitted to the calculation of the grand mean for research centers/universities. For another article with one experiment conducted in a zoo with a score of 2, and one experiment in a sanctuary with a score of 3, these two scores were submitted separately to the calculations of the grand means for zoos and sanctuaries, respectively.

### 3.7 Data availability

The data that support the findings of this study are openly available on the OSF at: https://osf.io/jf3tz/?view_only=3670701ac8624a4b87319ba7b131870e

### 3.8 Reliability

We assessed inter‐rater reliability using Cohen's kappa with IBM SPSS Statistics version 26. We calculated two types of reliability: inclusion and complete reliability, corresponding to our steps 1 and 2 coding, respectively.

#### 3.8.1 Inclusion reliability

Each reviewer randomly selected 10% of the total issues they reviewed (total number of issues reviewed = 1348; 135 issues included in the reliability assessment). The issues were selected by computerized randomization from the total list of issues reviewed by each person. From those issues, we compiled all published items (excluding obvious omissions such as book reviews, editorials, obituaries, and corrections) into a master file, and each reviewer (Emma S. McEwen, Elizabeth Warren, Sadie Tenpas, Kresimir Durdevic, Benjamin Jones, and Emilie Rapp Munro) assessed whether they would have included the items in the review based on our inclusion criteria. Each reviewer was blind to the original reviewer's decision during this process. In total, across the 135 issues included in this reliability assessment, 1643 articles were assessed, all of which were assessed by all reviewers. To analyze the reliability of our inclusion criteria, we compared each reviewer's inclusion decision for each article and found excellent agreement between reviewers (mean kappa = 0.83, SEM = 0.01, *n* = 15, range = 0.90–0.75).

#### 3.8.2 Complete reliability

Following the completion of data extraction for all allocated journals and years, each of the six reviewers randomly selected approximately 10% of their included articles (total number of articles included in review = 1119; *n* = 114 articles included in the reliability assessment for all variables except *sample size*, for which n was lower due to sample sizes coded as unavailable, and which ranged from 85 to 103 between pairs of reviewers). The articles were selected by computerized randomization from the total list of included articles reviewed by each person. We then compiled these articles into a master file, which each reviewer Emma S. McEwen, Elizabeth Warren, Sadie Tenpas, Kresimir Durdevic, Benjamin Jones, and Emilie Rapport Munro) coded in accordance with the developed coding scheme detailed previously to assess inter‐rater reliability. Each reviewer was blind to the original reviewer's decisions during this process, and each reviewer assessed each of the 114 items. To analyze the reliability of our coding scheme, we compared each reviewer's data extraction for the following variables: *topic*, *site type*, *apparatus*, *recording material*, and *sample size*, for the 10% of articles selected for this analysis. For cases in which more than one category was chosen (i.e., if a reviewer coded more than one topic or apparatus), if at least one topic or apparatus coincided between reviewers, we coded this as an agreement. If two coders listed the same two topics or apparatuses, we coded this as a single case of agreement. That is, for each variable in an article, we coded no commonality between categorizations as no agreement, and one or more commonality as a single agreement. We found very good agreement between reviewers across all variables (*topic*: mean kappa = 0.81, SEM = 0.02, *N* = 15, range = 0.94–0.71; *site type*: mean kappa = 0.87, SEM = 0.01, *N* = 15, range = 0.95–0.80; *apparatus*: mean kappa =  0.86, SEM = 0.01, *N* = 15, range = 0.90–0.78; and *recording material*: mean kappa = 0.86, SEM = 0.02, *N* = 15, range = 0.94–0.78). We used a Pearson correlation to assess the reliability of the variable *sample size*. We did not include cases in which at least one reviewer had reported the sample size as unavailable. To aggregate the sample sizes for studies with multiple groups of primates (i.e., housed in separate locations and/or different species), we calculated the mean sample size per article for the reliability assessment. Very strong agreement was found between reviewers (mean = 0.91, SEM = 0.01, range = 1.00–0.82).

## 4 RESULTS

### 4.1 Locations, species, and topics

#### 4.1.1 Locations

**Figure 2:**
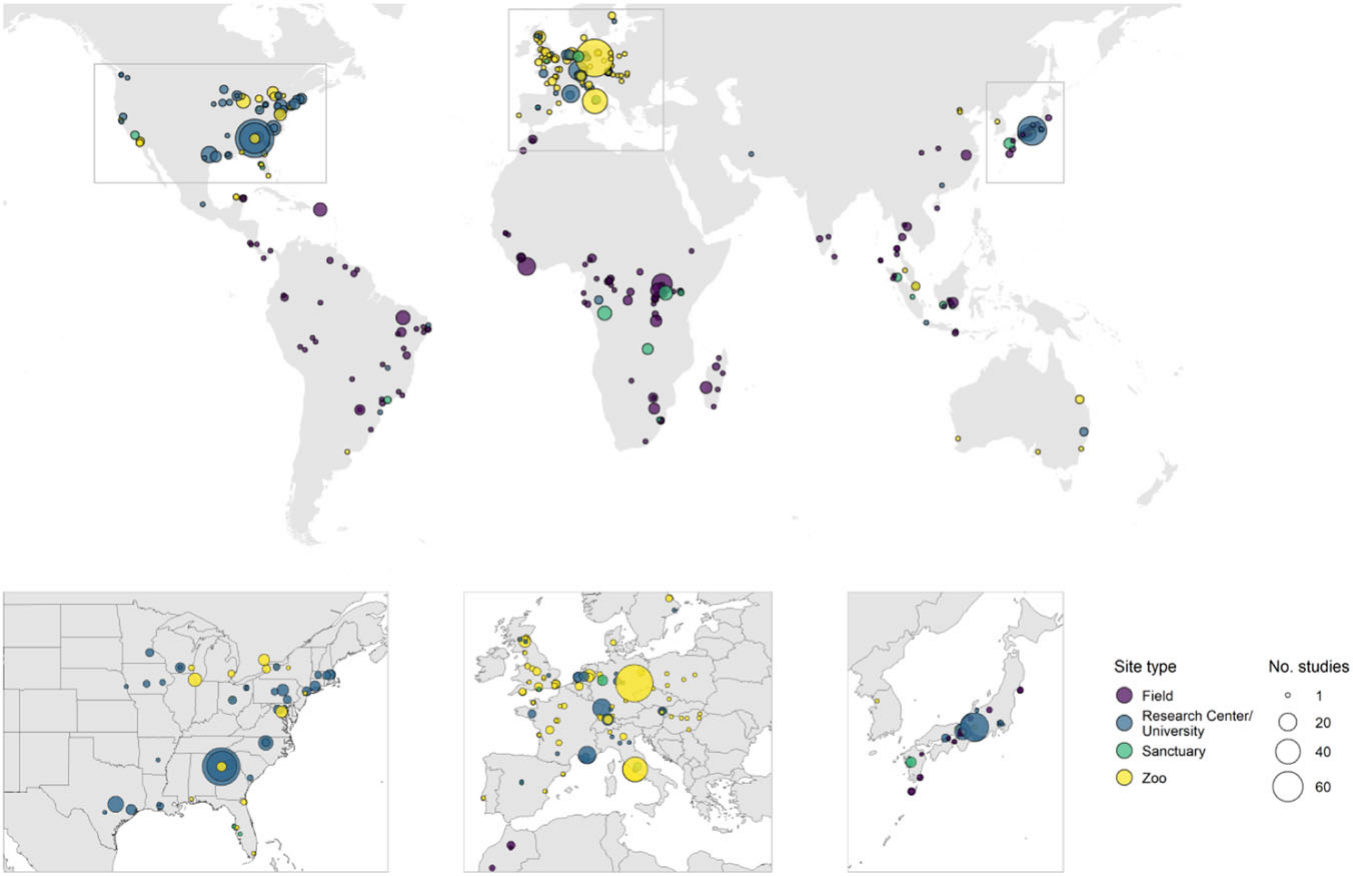
Locations of all sites included in this study (co‐ordinate data shown in supporting information). Site type is indicated by color, point size indicates the number of studies from each site. Adapted from Primates et al. (2019) and Watzek (2019)

We identified 325 distinct research locations in this study, summarized in Figure 2 (see supporting information for a full list of all locations). The distribution of studies across the four different site types among the 1119 articles included in the final sample are shown in Table 4, and the number of articles from each location is also depicted in Figure 2.

**Table 4:** Overview of site types

	Field	Research Center/University	Sanctuary	Zoo
No. Locations	128	81	21	95
No. Articles	302	488	68	270
No. Species	68	43	19	44

The majority of the articles included in our review report research conducted in research centers/universities (43.6%), followed by field sites (27%), closely followed by zoos (24.1%), and the smallest number from sanctuaries (6.08%) (Table 4); research centers/universities are the most productive site type, by this measure.

From Figure 2, certain outliers can be observed with considerably higher numbers of articles. Table 5 outlines all zoos with three or more articles included in this review, as well as which primate groups were studied in each location. The number of articles over the years indicated that a number of zoos showed long‐term collaborations with researchers from various research centers/universities. Moreover, three of the zoos which produced the most articles (Leipzig, Rome, Edinburgh) possessed a long‐term research collaboration involving capital investments from research centers/universities. Three zoos (Parco Natura Viva‐Garda Zoological Park, Rome Zoo, Howletts Wild Animal Park) exhibited the largest taxonomic coverage, with articles focusing on at least three of the five taxonomic groups considered.

**Table 5:** Overview of zoos with three or more articles in this review

Location	No. articles	Hominidae	Hylobatidae	Cerco‐pithecidae	Ceboidea	Prosimii
*Leipzig Zoo* a	95	**X**				
*Rome Zoo (Bioparco)* b	41			**X**	**X**	**X**
Lincoln Park Zoo	12	**X**		**X**		
Apenheul Primate Park	10	**X**				
*Edinburgh Zoo* c	9	**X**			**X**	
Smithsonian National Zoological Park	9	**X**	**X**			
Toronto Zoo	8	**X**		**X**		
Zoo Atlanta	6	**X**				**X**
Royal Burgers Zoo	5	**X**				
Zürich Zoo	5	**X**	**X**			
Buffalo Zoo	4	**X**		**X**		
Howletts Wild Animal Park	4	**X**	**X**	**X**		
La Vallée des Singes	4	**X**			**X**	
Marwell Wildlife Zoological Park	4		**X**	**X**		
Planckendael Zoo	4	**X**				
Rockhampton Botanical and Zoological Gardens	4	**X**				
San Diego Wild Animal Park	4	**X**			**X**	
Singapore Zoo	4	**X**				
Twycross Zoo	4	**X**				**X**
Berlin Zoo	3	**X**				
Detroit Zoo	3	**X**				
*Furuvik Zoo* d	3	**X**				
Paignton Zoo	3		**X**	**X**		
Parco Natura Viva‐Garda Zoological Park	3	**X**		**X**	**X**	**X**
San Diego Zoo	3	**X**				

*Note*: Crosses indicate the groups of primates studied. Locations with long‐term collaborations with research institutions are italicized, with affiliations listed in table footnotes. Primate icons adapted from BioRender.com (2021a; 2021b; 2021c; 2021d).

^a^ Wolfgang Köhler Primate Research Center, affiliated with Max Planck Institute for Evolutionary Anthropology.

^b^ Affiliated with Institute of the Science and Technology of Cognition, CNR.

^c^ Living Links Centre & Budongo Research Unit, affiliated with Scottish Universities: University of St Andrews, University of Stirling, and University of Edinburgh.

^d^ Lund University Primate Research Station, affiliated with Lund University.

To provide a measure of how many articles are produced by each site type corrected for overall number, we calculated a “productivity index” for each site type by dividing the number of articles (i.e., the number of primate cognition articles included in this review) by the number of locations (see Table 6). Another way to conceptualize this is the average number of publications per location, within each site type. This analysis was only possible for locations and articles for which location name information was available.

$$\mathrm{Productivity}\;\mathrm{index}=\frac{\mathrm{Number}\;\mathrm{of}\;\mathrm{articles}}{\mathrm{Number}\;\mathrm{of}\;\mathrm{locations}}.$$

**Table 6:** Productivity index for each site type

	Field	Research Center/University	Sanctuary	Zoo
Productivity index	2.35	5.96	3.19	2.84

These data show that, of the four site types, research centers/universities are the most productive; they contribute to the most primate cognition articles compared to field sites, sanctuaries, and zoos, and this pattern is also found when considering the overall number of sites. Research centers/universities are followed by sanctuaries as the next most productive site, and zoos and field sites are the least productive by this index. It should be noted, however, that within our productivity index we only considered those sites which were identified in this review as contributing to research. These results would likely differ greatly if we considered all possible locations which house primates. For example, while it may be the case that many research centers housing primates produce research, it is not the case that research is conducted in all zoos housing primates. Therefore, the term “productivity” should be interpreted with caution, and only captures the average number of publications per location within this sample.

#### 4.1.2 Species

**Figure 3:**
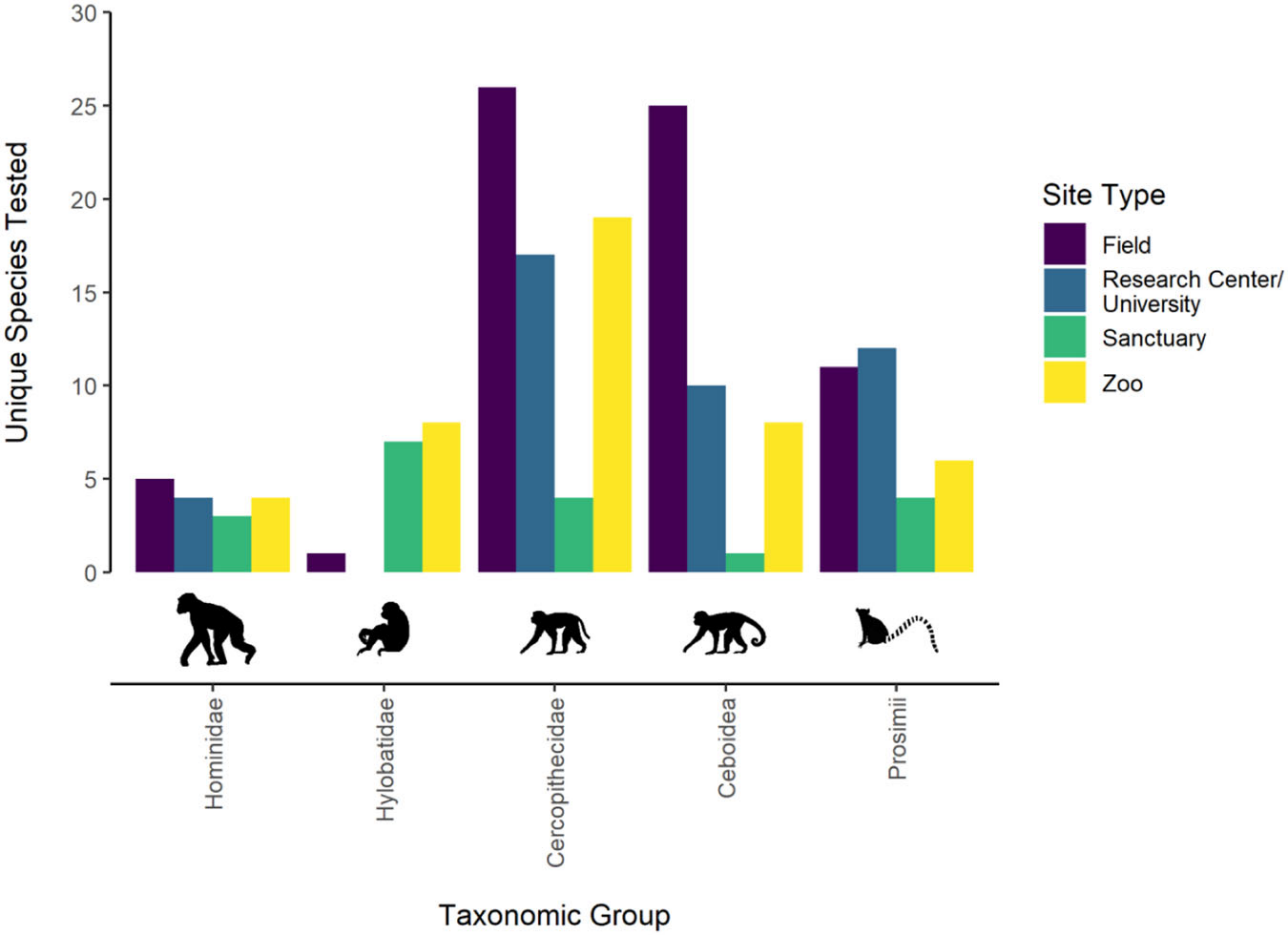
Number of unique species tested at each site type (indicated by color) within each primate group. Icons indicate examples of primates belonging to each classification. Primate icons adapted from BioRender.com (2021a; 2021b; 2021c; 2021d)

The number of unique species tested was highest among field sites (68), followed by zoos (44) and research centers/universities (43), and the lowest number was among sanctuaries (19) (Table 4). The number of unique species tested within each primate group at each site type is shown in Figure 3 (see Table S1 for details).

From these values, we calculated a “publication species diversity index” whereby the number of species tested at each site type was corrected for number of articles from each site type, to give an indication of the species diversity among publications from the four site types. We further calculated a publication species diversity index within each primate group (Table 7).

$$\mathrm{Publication}\;\mathrm{species}\;\mathrm{diversity}\;\mathrm{index}=\frac{\mathrm{Number}\;\mathrm{of}\;\mathrm{species}}{\mathrm{Number}\;\mathrm{of}\;\mathrm{articles}}$$

**Table 7:** Number of species, number of articles, and publication species diversity index across primate taxa for each site type

	Field	Research Center/University	Sanctuary	Zoo
	Species/Articles			
	Publication species diversity index			
Hominidae	5/113	4/125	3/54	4/182
	*0.04*	*0.032*	*0.06*	*0.02*
Hylobatidae	1/3	0/0	7/4	8/7
	*0.33*	*–*	*1.75*	*1.14*
Cercopithecidae	26/104	17/197	4/6	19/34
	*0.25*	*0.09*	*0.67*	*0.56*
Ceboidea	25/68	10/187	1/4	8/51
	*0.37*	*0.05*	*0.25*	*0.16*
Prosimii	11/14	12/25	4/1	6/7
	*0.79*	*0.48*	*4*	*0.86*
Total	68/302	43/488	19/68	44/270
	*0.23*	*0.09*	*0.28*	*0.16*

*Note*: The different number of species in each taxon renders inter‐taxon comparisons uninformative but not inter‐site comparisons within each taxon.

From the absolute values (shown in Table 4), we see that more species are studied in the wild, similar numbers are studied in zoos and research centers/universities, and fewer in sanctuaries. If, however, these values are corrected for the number of articles from each site type (publication species diversity index, Table 7), field sites and sanctuaries produce more diverse publications than zoos, but all produce more diverse publications than research centers/universities. We note that we lack information about the total number of species located at each site type. Our data simply describe which species we found had been studied at each site type, and not the proportion of overall available species that have been studied at each.

For Hominidae, we see that there are large numbers of publications across all site types. Although the index score is higher here for sanctuaries, this is an artifact of the small number of great ape species. The indexes for Hylobatidae show greater diversity in sanctuaries, but these results should be interpreted with caution, given the small number of articles included here.

For both groups of monkeys, research centers/universities have the lowest diversity scores; there are fewer species studied here across a greater number of articles. Cercopithecidae had the greatest diversity in sanctuaries and zoos, and research centers had the lowest diversity indexes. Ceboidea had the greatest diversity in field sites, and the lowest in research centers, by this index.

In prosimians, diversity among field and zoo studies were similar, and again both scored more highly than research centers/universities. The index score from sanctuaries is difficult to interpret here, given that only one study was included.

**Figure 4:**
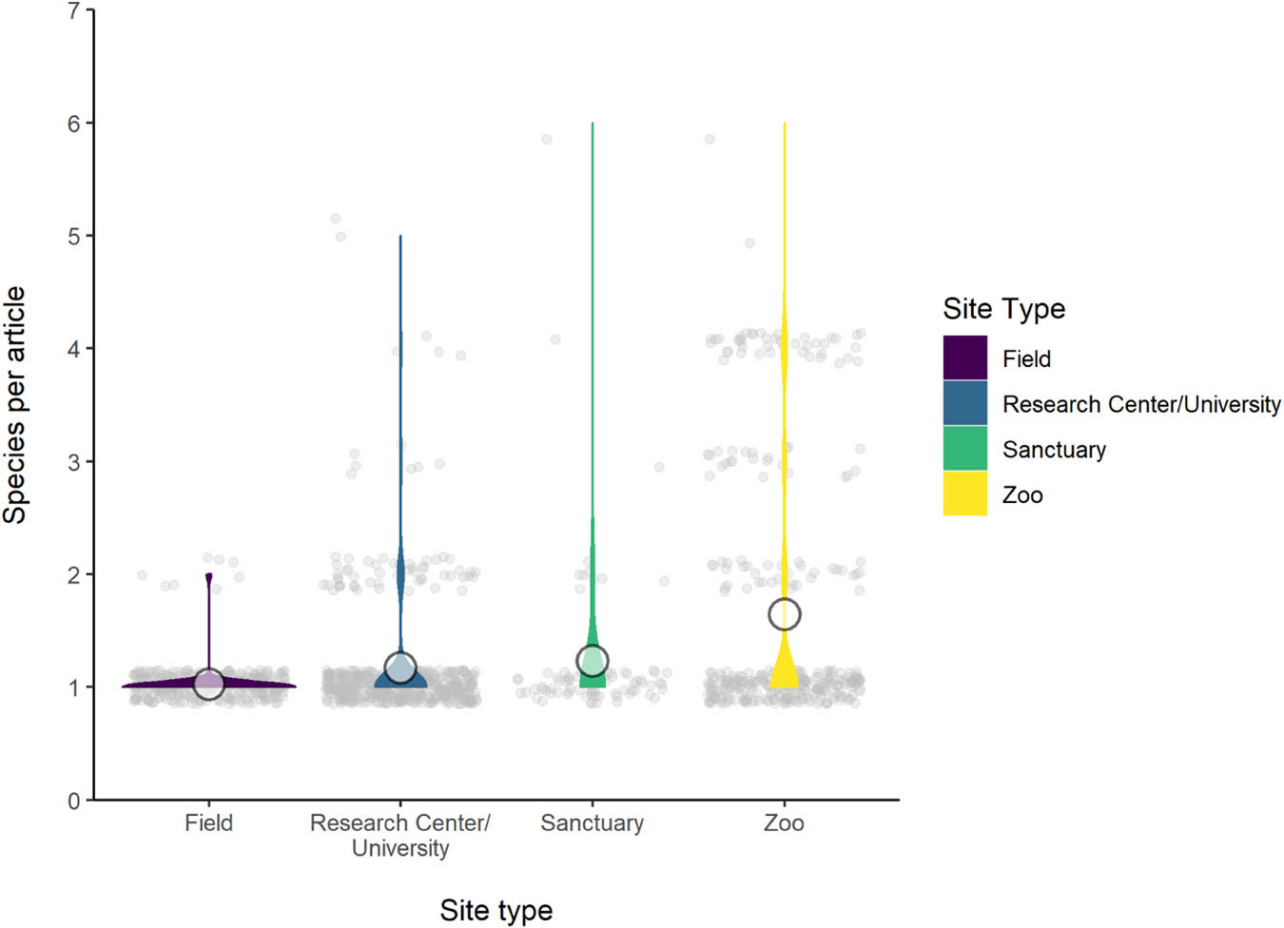
Violin plots indicate the number of unique species per location, per article, across the site types (indicated by color). Circles indicate mean number of species per paper

Last, we counted the number of species from each location included in any given article (Figure 4). For example, if one article contained data from two species in one zoo, and one species in another zoo, these were counted as separate entries, rather than one cumulative entry of three species for the whole article. This was in order not to inflate the number of species per location for studies with multi‐site collaborations, and to give an indication of how many species were included from each individual institution among the four site types. While the means were low across the site types, zoos had the largest mean number of species per location, per article, as well as the highest variability (mean number of species per field site, per article = 1.02 [SD = 0.16], per research center/university, per article = 1.17 [SD = 0.52], per sanctuary, per article = 1.23 [SD = 0.74], and per zoo, per article = 1.64 [SD = 1.10]). Although studies conducted in the field and in research centers/universities had the highest and second highest relative number of species, respectively, zoo‐based studies were the most likely to have more species included from one location in one article than studies from other site types.

#### 4.1.3 Topics

**Figure 5:**
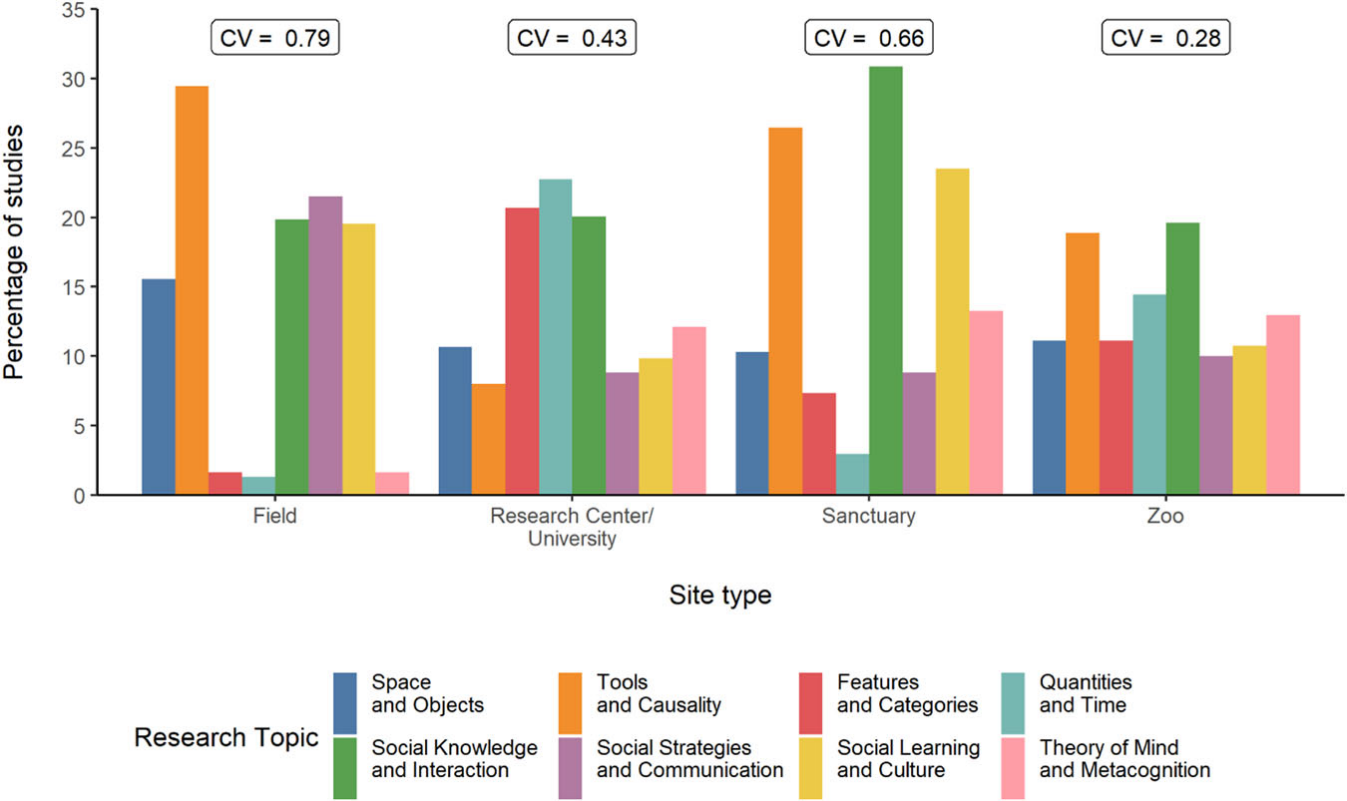
Percentage of studies is categorized as each topic of cognition (indicated by color) from each site type. Note that some studies investigated more than one topic and were counted once per topic, hence the sum of the proportions may exceed 100%. The coefficient of variation (CV) is displayed above the bars

The most and least common research topics in field sites were Tools and Causality (29.47%) and Quantities and Time (1.32%), respectively. Conversely, in research centers/universities, most common were Quantities and Time (22.75%), and least common were Tools and Causality (7.99%). Social knowledge and Interaction was the most common topic in both sanctuaries and zoos (sanctuaries: 30.88%, zoos: 19.63%), but Quantities and Time was least common in sanctuaries (2.94%), whereas Social Strategies and Communication was least common in zoos (10%) (Figure 5, Table S2).

To investigate the amount of variation in topics studied across site types, we also calculated the coefficient of variation (SD/mean) for the proportions of studies on each topic, for each site type (Figure 5, Table S3). A higher coefficient of variation indicates more variation in the proportions, suggesting possible biases towards certain topics.

Although all topics were studied in all site types, the coefficients of variation show vast differences in the heterogeneity of research topics among the site types. Zoos had the smallest coefficient of variation, indicating the most balance between proportions of research areas studied. Field sites had the most variation in proportions; some topics are investigated in many publications, while others are studied very little in field sites.

### 4.2 Methods, apparatuses, and sample sizes

**Figure 6:**
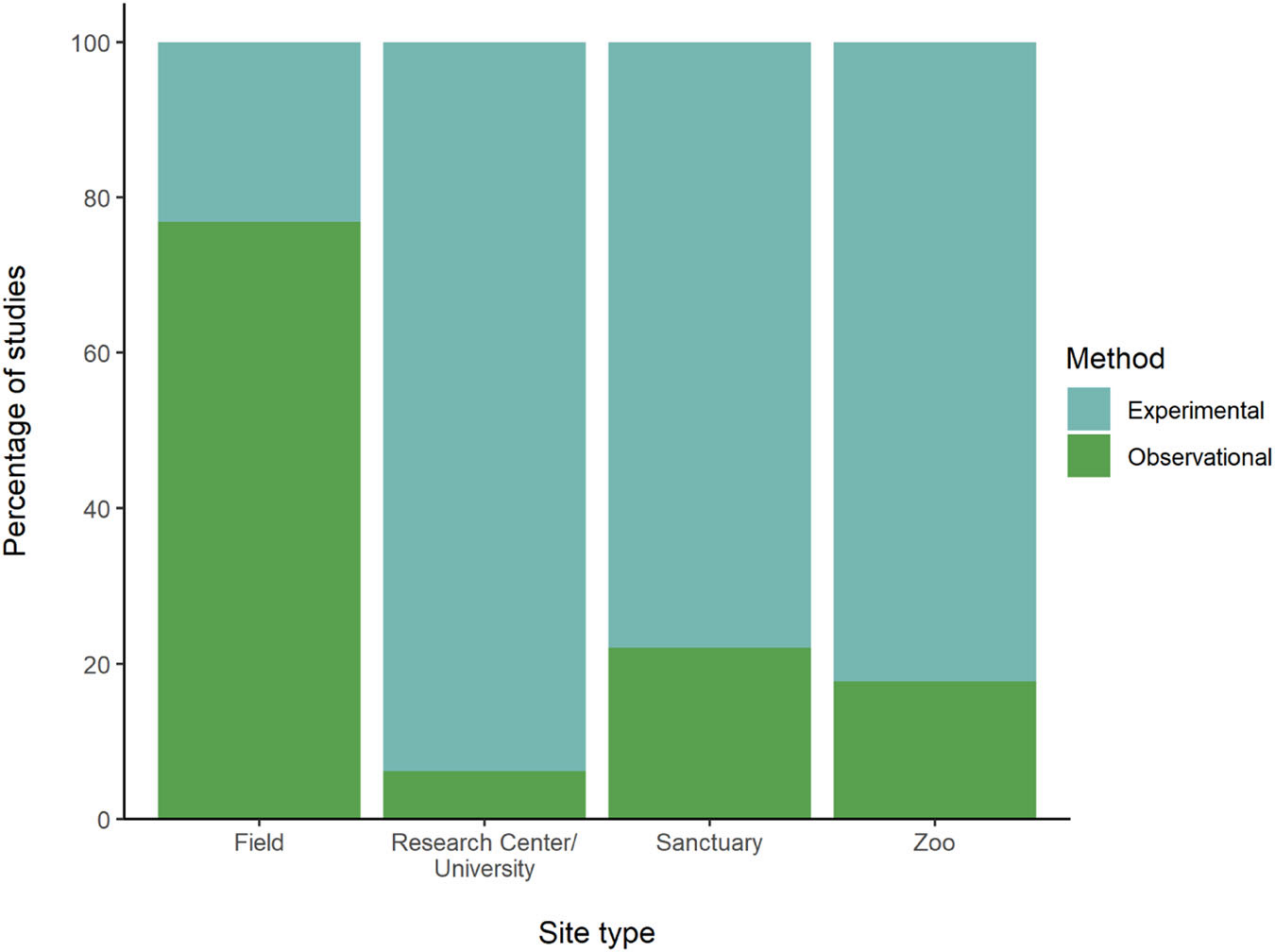
Proportion of observational and experimental studies (indicated by color) conducted at each site type

**Figure 7:**
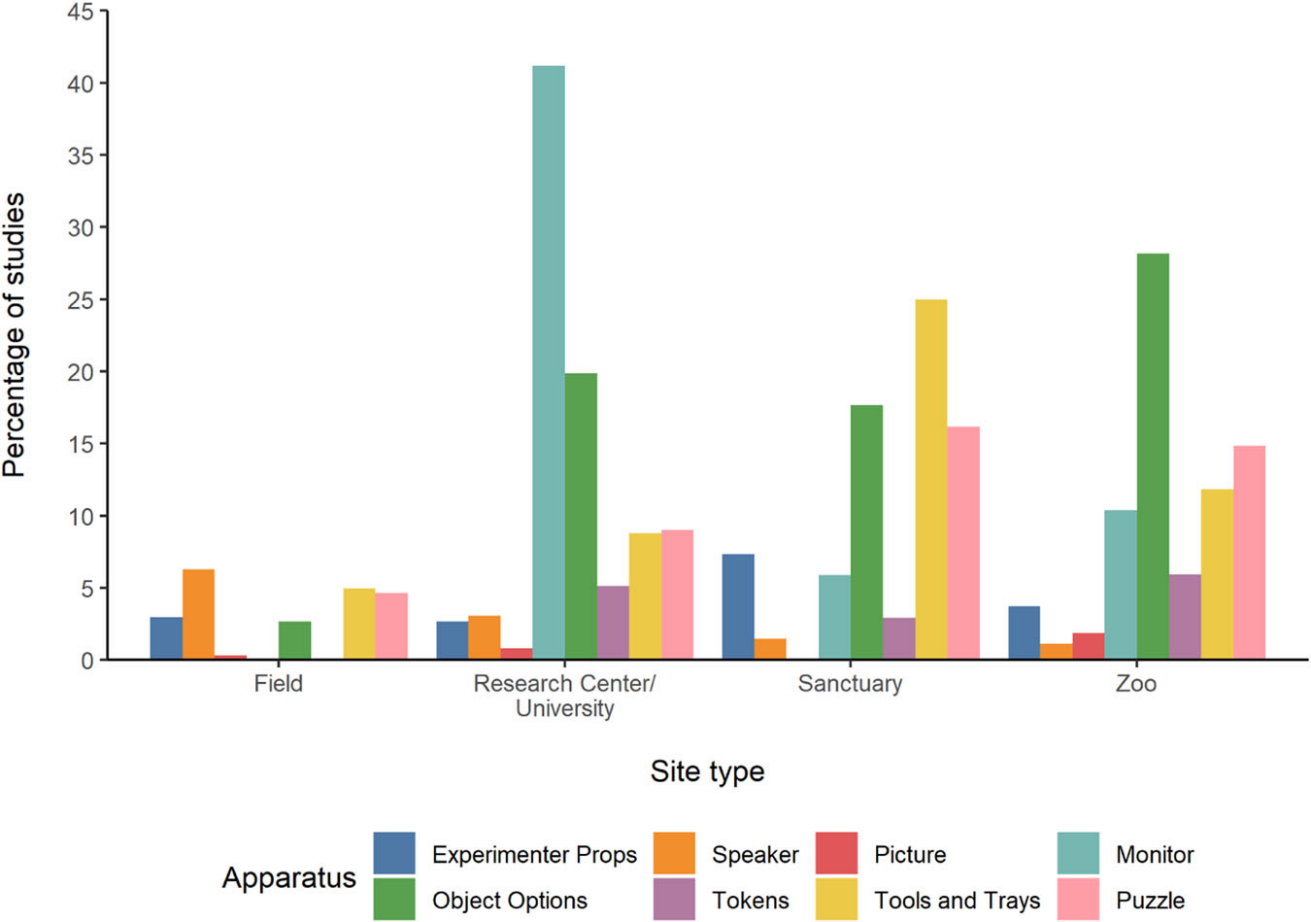
Percentage of studies using each apparatus type (indicated by color) from each site type. Note that some studies used more than one apparatus and were counted once per apparatus, hence the sum of the proportions may exceed 100%

**Figure 8:**
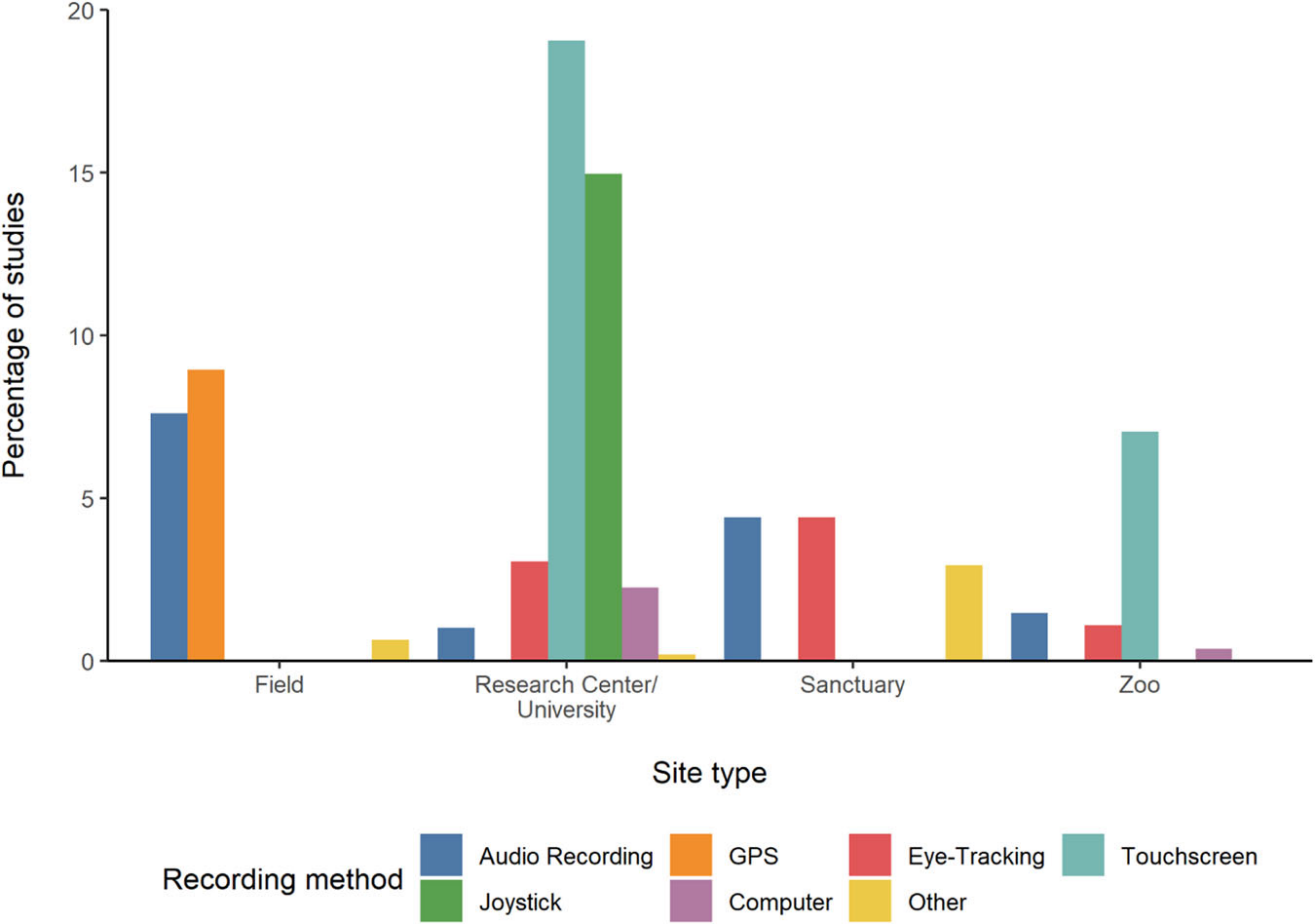
Percentage of studies using each recording method (indicated by color) from each site type. Note that some studies used more than one recording method and were counted once per recording method, hence the sum of the proportions may exceed 100%

The proportions of observational and experimental studies within each site type are shown in Figure 6 (see also Table S4). The proportion of experimental studies was considerably higher than observational studies in all site types other than field sites. An overview of the types of apparatus used for each study in each site type is shown in Figure 7 (see also Table S5). Instances of additional recording methods from each site are shown in Figure 8 (see also Table S6).

**Figure 9:**
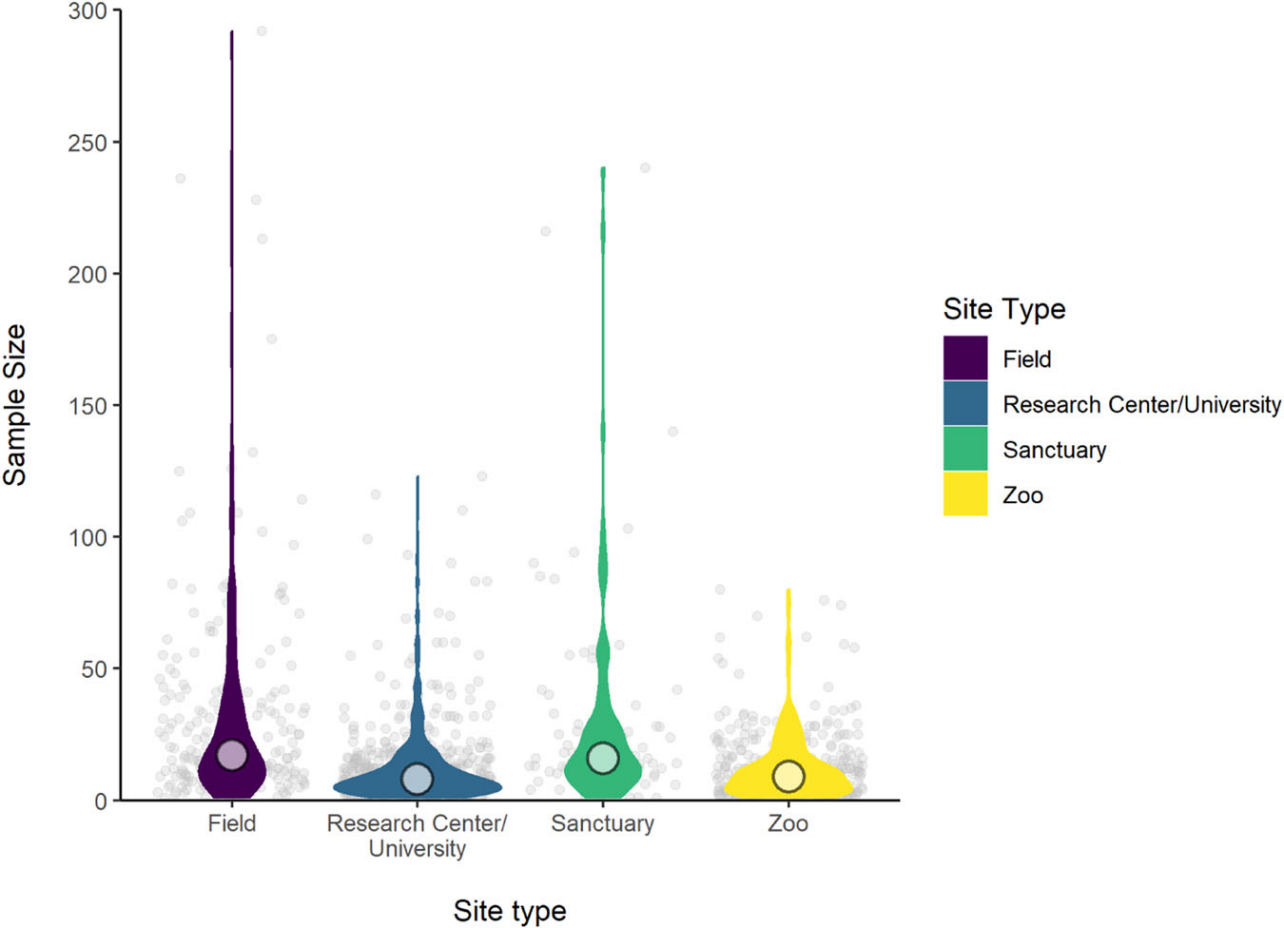
Distribution (violin) and median (circle) of sample sizes across the different site types (indicated by color). Note that one outlier was omitted from this figure for clarity (sample size = 506, from a research center/university)

The distribution of sample sizes, for those papers for which this information was available, is shown in Figure 9. For each article, we counted how many subjects were studied at each location, regardless of species (see Table 8 for summary statistics). For example, if five chimpanzees were tested at one zoo, and six chimpanzees and three orangutans were tested at another zoo, these were counted as two separate entries of *N *= 5 and *N *= 9 for zoo one and zoo two, respectively. This was to provide an overview of how many individuals participated in research at each individual institution among the four site types. Research centers/universities and zoos had considerably smaller, although comparable, median sample sizes (8 and 9, respectively) than field and sanctuary studies (17 and 16, respectively). It should be noted, however, that field sites were the most likely of the site types not to report a sample size (e.g., reporting overall group sizes in observational studies rather than number of focal subjects, which can be difficult to gauge when subjects cannot be individually identified) and so caution should be taken when interpreting this data.

**Table 8:** Medians and interquartile ranges of sample sizes per article at each site type

	Field	Research Center/University	Sanctuary	Zoo
Median	17	8	16	9
IQR	27	11	18.2	12

### 4.3 Trends over time

**Figure 10:**
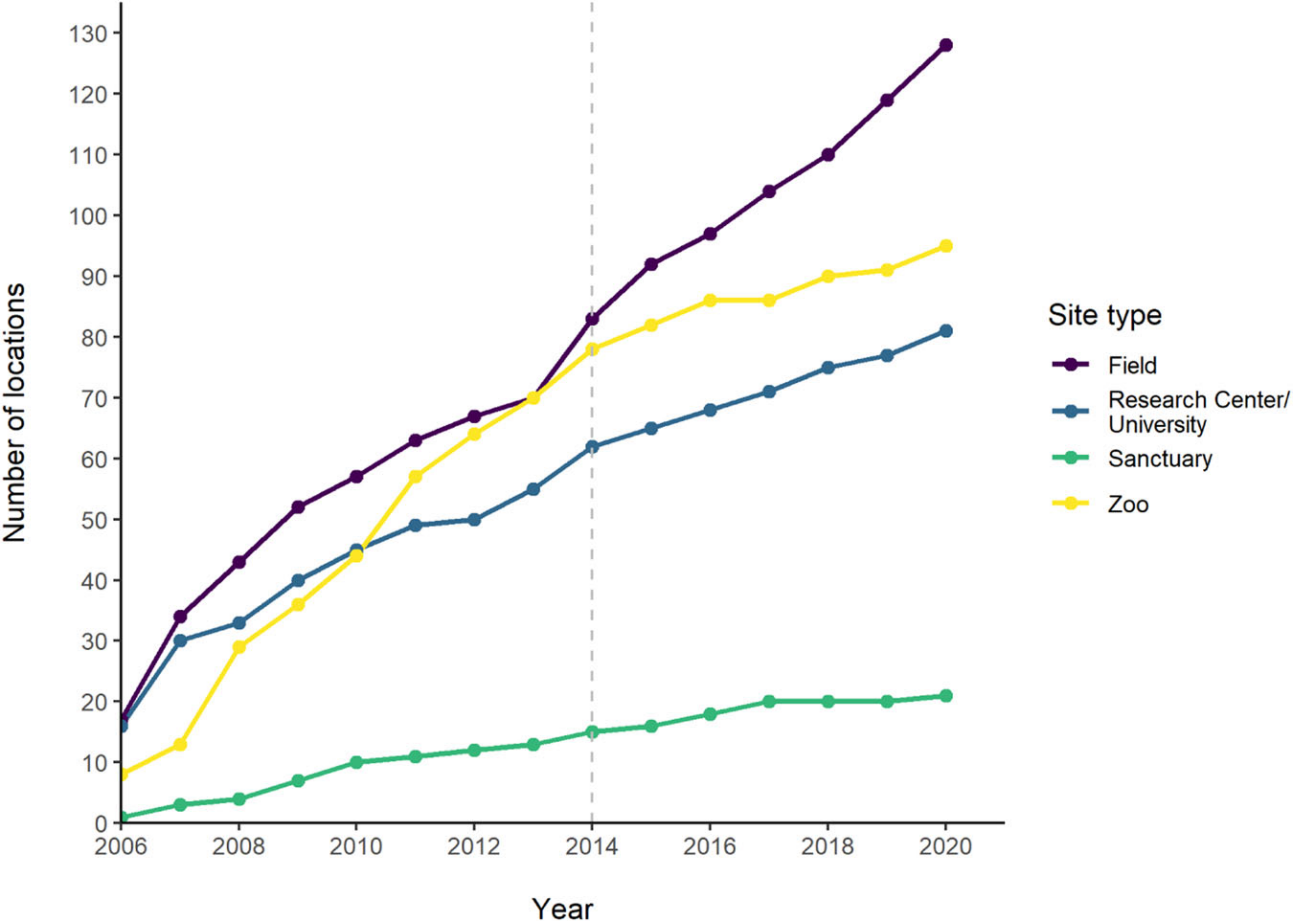
Cumulative count of locations of each site type across the years 2006–2020. Gray dashed line indicates the year from which 12 journals were included, before this only 11 journals were included

**Figure 11:**
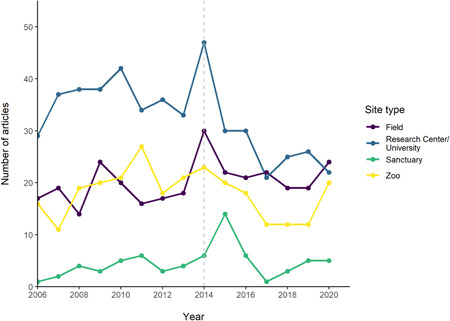
Number of articles from each site published in the journals included in this review across the years 2006–2020. Color indicates site type. Gray dashed line indicates the year from which 12 journals were included, before this only 11 journals were included

**Figure 12:**
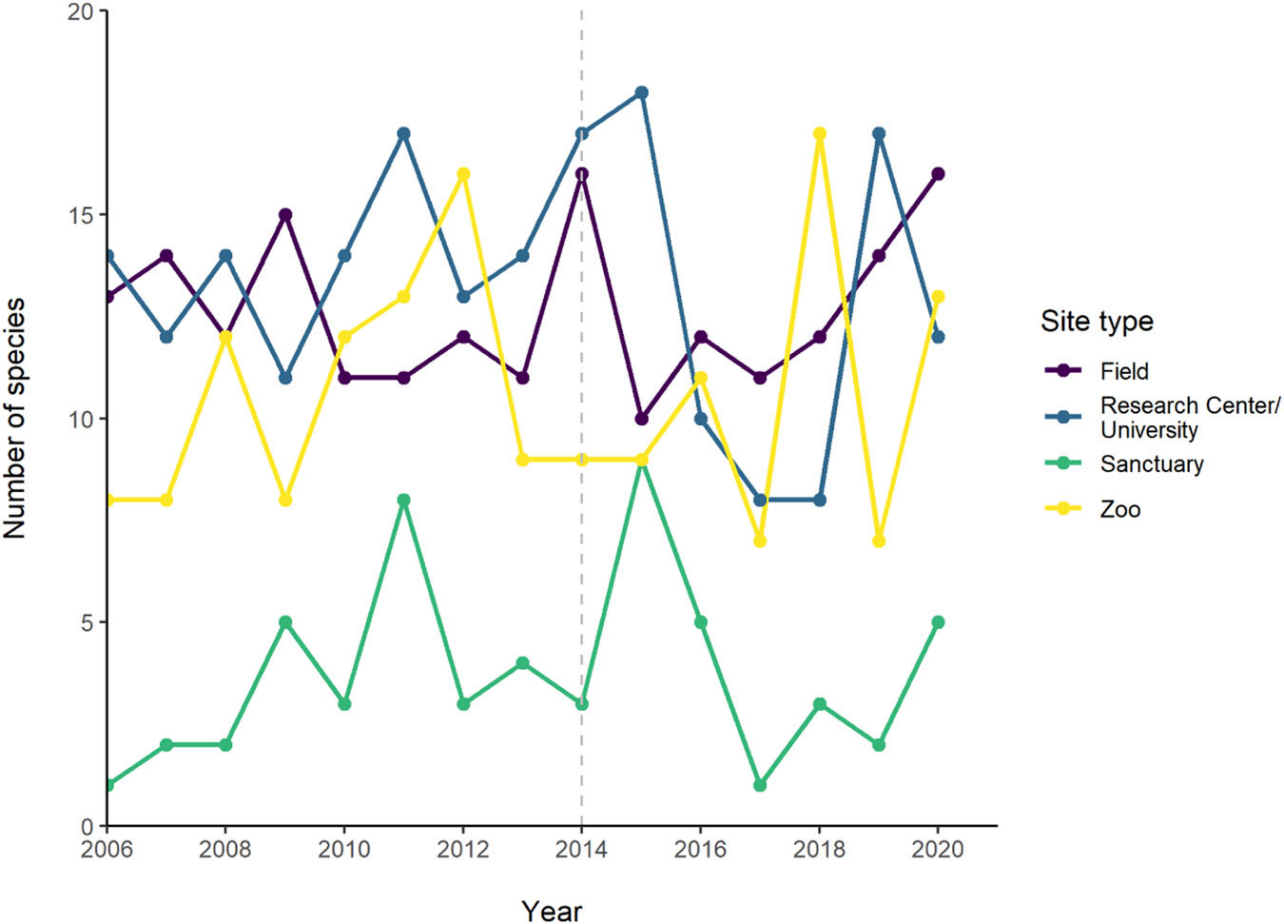
Number of primate species studied at each site type across the years 2006–2020. Color indicates site type. Gray dashed line indicates the year from which 12 journals were included, before this only 11 journals were included

The cumulative count of locations increased each year for all site types (Figure 10, Table S7). This was to be expected, as we identified more sites as we counted over the years, but it is of note that none of the site types reached a plateau; all four continued to increase in number. No clear trends were seen in the number of articles from each site published each year, and a similar number of articles from each site type were identified in the first and last year included in this review (Figure 11, Table S8). Likewise, no clear trends in number of species in each site type across the years were seen (Figure 12, Table S9).

#### 4.3.1 Summary

Next, we provide a brief research profile for each site type (see Table 9).

**Table 9:** Summary of key indices across all site types

	Field	Research Center/University	Sanctuary	Zoo
Productivity index	2.35	5.96	3.19	2.84
Publication species diversity index	0.23	0.09	0.28	0.16
Mean number of species per article (±SD)	1.02 (±0.16)	1.17 (±0.52)	1.23 (±0.74)	1.64 (±1.10)
Topic variability index	0.79	0.43	0.66	0.28
Technological sophistication (mean ± SD)	0.74 (±1.10)	2.64 (±1.31)	1.79 (±1.24)	1.90 (±1.27)

*Note*: Publication species diversity index indicates the number of species/number of articles. Topic variability index refers to the coefficient of variation of the proportions of topics studied.

Field sites were the lowest in productivity, middling in diversity of species among publications, tended to have one species per paper, had high variation in proportions of research topics (indicating some biases towards certain topics), and tended to use less technologically advanced apparatuses and recording materials.

Research centers/universities were the most productive site type, but with very low diversity of species among these publications, some variation in proportions of research topics, and tended to use the most technologically sophisticated (i.e., more computerized and specialized) apparatuses and recording materials.

Sanctuaries were the second most productive site type, with the most diverse publications and a slightly higher mean number of species per paper than field sites and research centers/universities. Sanctuaries had a relatively high topic variability index, indicating a specialization bias, and the second lowest technological sophistication scores.

Finally, zoos were middling in productivity (slightly higher than field sites), and in publication species diversity. Zoos had the highest average number of species per article of all site types, and the least variation among proportions of topics studied indicating minimal bias and reasonable balance between topics. In terms of technological sophistication, zoos were the second highest after research centers/universities.

## 5 DISCUSSION

In this systematic review, we examined 15 years of 12 journals to investigate the impact of zoos on primate cognition research. We described the differences between four types of sites in which primate cognition research is conducted: zoos, field sites, research centers/universities, and sanctuaries. Specifically, we looked at the number of articles from each site type, the species diversity across site types, diversity of topic of cognition within and between site types, research methods implemented in the different locations, and sample sizes. With a focus on zoos, we have presented an overview of the ways in which primate cognition research has been implemented in different settings.

Our review showed that, while the majority of primate cognition research takes place in research centers/universities, almost 25% takes place in zoos, a contribution similar in magnitude to that of field sites (in which just over 25% of research takes place), indicating that zoos are contributing to our discipline to a reasonably large extent. Research centers/universities contributed to the largest number of articles, and this level of productivity was found even when we adjusted for the overall number of sites. While we acknowledge the productivity impact of research centers/universities, it is also important to consider the contribution of other sites in other aspects.

Species diversity is an important consideration in primate cognition research. That is, to better understand a particular cognitive trait, or how it may have evolved in primate species, it is important to study a diverse number of species across the phylogenetic tree. We found in our review that a little over 20% (110) of the approximately 500 known (Burgin et al., 2018) primate species have been studied across the different site types, a slightly higher figure than the 15% noted in Primates et al. (2019).

We assessed species breadth across different site types. Looking at the overall number of species studied at each location type, the largest number are studied in field sites, and the least in sanctuaries. The number of species studied in zoos and research centers/universities was almost equal. When the overall number of articles by site type is taken into account, research centers/universities were the least diverse in terms of species studied, a trend we found for all primate groups. Zoos offered even greater species diversity than field sites for all primate group except Hominidae and Ceboidea (great apes and monkeys of the Americas). Sanctuaries' species diversity scores were also relatively high across primate groups. One primate group (Hylobatidae/lesser apes) was not studied in research centers/universities at all, further highlighting the value of alternative site types. We also found that it was slightly more common for zoo‐based research to include multiple species per location per article, which could be an important consideration for the practicality of future research as it may mean that a research program at only one site could offer opportunities for multi‐species comparisons. In fact, the combination of a good inter‐ and intra‐article species diversity makes zoos (and sanctuaries) two of the most suitable locations to study primates from a comparative perspective.

Primate cognition research encapsulates a wide array of topics. To provide a complete picture of the minds of different primate species, and to understand the evolution of our own cognitive abilities, it is important to ask diverse research questions covering a broad range of skills. While some of the eight broad topics that we considered are underrepresented in certain sites, such as Theory of Mind and Metacognition in field studies, and Quantities and Time in sanctuaries, the distribution of topics studied in zoos is remarkably balanced. This finding may suggest that zoos, compared to other site types, both attract researchers from diverse sub‐fields in primate cognition and are well‐suited to the study of a wide range of topics. There may, however, be other contributing factors not captured in this data set, such as funding availability, established research programs continuing certain lines of work, and legal and logistical constraints in habitat countries, to name but a few.

Research in our discipline utilizes a multitude of methods and apparatuses. Often, we require apparatuses to be specialized in such ways which make them safe and practical to present to primates and recording methods which allow us to measure nonverbal responses. Overall, we saw that experimental research was most common in zoos, sanctuaries, and research centers/universities, but that observational studies were more common in the field. Interestingly, the proportion of observational studies was considerably low in research centers/universities, but higher in zoos and sanctuaries (approximately 20% of studies in these settings was observational, compared to just over 5% in research centers/universities). Hayden et al (2021) point out that zoos lend themselves to observational studies well, as they, unlike research centers, are designed specifically in consideration of unobtrusive, clear viewing of primates. Similarly, sanctuaries, with their often semi‐wild enclosure styles, may invite the use of field techniques such as remote video monitoring for both animal care and observational research, as in Hansen et al. (2018).

With regard to the apparatuses used, we found that research in zoos and research centers/universities used all types of apparatuses considered in this review, and all apparatuses other than ‘picture' were also used in sanctuaries, suggesting that each of these settings can accommodate a range of methods. Neither tokens nor monitors were used in field studies, possibly due to practical constraints.

We also coded any recording methods used beyond simple video or pen and paper recording of behavior. Studies in research centers/universities were the most likely to use additional recording methods, although notably field research was the only site type to use GPS technology and the most likely to use audio recording, and zoos were the only site other than research centers/universities to use touchscreens. These results show the large amount of research possible without technologically sophisticated recording methods, whilst also highlighting the possibilities for their use in the different settings.

We acknowledge that other factors contribute to methods, apparatus selection, and recording materials, such as funding availability, prior training or experience of participants, and ongoing research set‐ups. Different methodologies serve different purposes and come with different advantages and disadvantages, which may depend on the species to which they are presented, and the topic under consideration. Moreover, differences in technological sophistication could relate to expenses, access to electricity, physical barriers between experimenters and subjects, durability of materials, and, in some cases, the most suitable apparatus for a study design may be a technologically simple one.

Sample sizes are also important considerations in cognitive research, and especially in primate cognition research in which they tend to be particularly small, often resulting in underpowered studies (Primates et al., 2019). We found that sanctuaries and field studies tend to have larger sample sizes than research centers/universities and zoos. Although zoos had the second smallest average sample sizes, these were just above, yet comparable to those found in research centers/universities. Initiatives such as Primates et al. (2019) are already making progress by organizing multi‐site collaborations in which a single experimental protocol is presented in numerous sites to achieve large sample sizes of primates spanning across multiple taxa. In our review, we saw that multi‐institution collaborations more often included zoos than other site types. Among the studies which took place across multiple locations, 53.41% included at least one zoo, 38.64% at least one research center/university, 26.14% at least one field site, and 15.91% at least one sanctuary, which could indicate that working in those locations with smaller sample sizes encourages more multi‐site collaboration (though other factors such as geographical proximity may also play a key role). Although sample sizes in zoo‐based research tend to be small, it is not a problem unique to zoos and we hope that more multi‐site collaborations succeed in alleviating this issue which has been so inherent in our field.

No clear change in number of locations or number of articles over time was observed, possibly indicating that the years included in our review were too late to capture the point in time at which the shift towards zoo‐based research occurred. Further, no clear change over time was observed when considering the number of species studied at each site type. In total, our review included data from almost a hundred zoos, which further indicates the magnitude of their contribution. Although sanctuaries were the smallest category in terms of number of publications, this may reflect difficulty in accessing such facilities and is symptomatic of the fact that they are few in number (indeed, we identified only 21 primate sanctuaries in this review), and their value should not be understated.

Zoos have been the central focus of this review, but we must acknowledge that each of the site types included present benefits and challenges. Field research offers both vast species diversity and ecological validity, but this study is not without challenges. Logistical and financial barriers such as travel considerations and research licenses, as well as legal and practical considerations of experimental interventions in otherwise undisturbed wildlife can present obstacles to conducting field research. Research centers/universities offer experimental control and frequent access to primate participants, but often come with financial costs of housing and managing the animals. In some cases, research centers may also lack ecological validity, due to nonnaturalistic housing and rearing conditions. Sanctuaries can provide experimental control whilst often housing primates who have spent some of their lifetimes outside of captivity, and/or who are semi‐free ranging, presenting an opportunity to study more naturalistic behaviors than some other captive settings. Sanctuaries are few in number, however, and can also involve financial and logistical barriers relating to travel and permission to conduct research. Zoos offer the opportunity to study primates across diverse taxa in controlled experimental settings, with detailed information of each animal's history (Hayden et al, 2021). Zoo‐based research is not immune to practical challenges, however, including limited or restricted access to animals, willingness to collaborate with researchers, and travel considerations. Each site type brings different constraints and advantages, not all of which can be captured in this study.

Another interesting facet of zoo‐based research is the impact of relationships between zoos and research institutions. In some cases, a longstanding relationship exists between a zoo and a research center, such as the Wolfgang Köhler Primate Research Center in Leipzig Zoo and the Max Planck Institute for Evolutionary Anthropology, Rome (Bioparco) Zoo with the Institute of the Science and Technology of Cognition, CNR, and the Living Links Centre and Budongo Research Unit at Edinburgh Zoo with Scottish universities; three of the zoos which produced the most articles in this study. This sort of collaboration may lead to greater impact of certain sites, both for the researchers and for the zoo, and further investigation into these relationships would be of interest for future work.

Although the approach taken in this systematic review was thorough, we must emphasize that this is only a snapshot of the diverse range of primate cognition research conducted across the world. It is important to acknowledge that, while we worked with our own definition of cognition, this may not be universally agreed upon. Indeed, there were certain topic areas which proved particularly challenging to categorize, such as social behavior, perception (which was not included as a topic of cognition here), and vocal communication. This may have resulted in slightly over‐ or under‐representing the number of studies conducted. Our reliability scores, however, indicate that at least within our working definition of cognition, there was consistency in scoring. Furthermore, this review should not be taken as an exhaustive list of studies from the last 15 years, and it may be the case that our journal selection introduced certain biases into our literature review. Whilst we tried to choose representative journals in three different, yet connected, fields, many other journals publish primate cognition research, and it may be the case that certain trends were not captured here. For example, researchers in certain topics or working in certain settings may publish more frequently in developmental journals, nonspecialist journals, and mega‐journals, which were not included here. We note that the aim was not to cover all fields, rather only those we targeted (primatology, animal behavior, and animal cognition). This study also did not include papers without novel data (i.e., reanalyzed data, meta‐analyses), which may have resulted in understating the impact of some sites in terms of number of publications produced. With these caveats in mind, however, this study provides an overview of the variation between sites at which research in the field of primate cognition is being conducted.

The focus of this study was to investigate how the field of primate cognition benefits from zoos. It is also important, however, to consider the positive impacts that zoo‐based research can have on the primates and institutions themselves. Primate cognition research conducted in zoos can have positive implications such as contributing to animal welfare and enhancing science education (Egelkamp & Ross, 2019).

A key benefit of primate cognition research in zoos is the cognitive enrichment provided to the animals; for example, offering primates the choice to engage in novel tasks and solve problems (MacDonald & Ritvo, 2016). Cognitive challenges which offer opportunities for exploration and overcoming challenges to achieve goals are important for captive animal welfare, including that of primates (Clark, 2011, 2017). Zoo‐housed primates have been seen repeatedly choosing to engage with research (Herrelko et al., 2012). Importantly, assessments of welfare have found no negative impacts of cognitive research on chimpanzees (Herrelko et al., 2012), and have even found reductions in aggressive behavior and increases in affiliative behavior in crested macaques during periods of cognitive research (Whitehouse et al., 2013). Further, positive reinforcement training similar to that used in many cognitive tasks has been linked to reductions in stress‐related behaviors in chimpanzees (Pomerantz & Terkel, 2009).

While the primary goal of primate cognition research is for the researchers themselves to learn about their participants, a secondary positive outcome of research in zoos is public education. In a number of zoos, visitors are able to watch animals participate in research, which teaches the public about our field and the ways in which we conduct our research (Hopper, 2017; MacDonald & Ritvo, 2016). In some cases, having on‐show research with capuchins and squirrel monkeys has led to increased visitor engagement with exhibits (Bowler et al., 2012) and visitors to a zoo‐based research center with crested macaques reported learning more about primates and conservation when seeing a scientist present working the animals (Waller et al., 2012). Additionally, visitors who observed research or husbandry demonstrations at a great ape exhibit and Lincoln Park Zoo reported increased interest in apes and knowledge of the zoo's research and conservation work (Price et al., 2015). There is also evidence that, for Japanese macaques, visitor presence at an on‐show research exhibit does not negatively impact the animal's welfare, participation rates, or task accuracy (Huskisson et al., 2021). Primate cognition research in zoos can benefit public engagement and science education which, in turn, could further benefit the discipline and the animals.

We have presented here (to our knowledge) the first fully systematic review of over a decade of primate cognition research to compare number of publications, species diversity, research methods, and sample sizes across four different site types: field sites, research centers/universities, sanctuaries, and zoos. We developed a coding scheme which we hope others can use to replicate and extend this study, to look at other trends and more specific nuances in primate cognition research. We hope that this study will encourage more research in zoos, both by highlighting some of the possible methods and by illustrating the benefits of working in these settings, such as species diversity, controlled experiments with numerous apparatuses, and observational studies. Further, we hope that our data set can be used as a resource for others to explore what is possible in different research settings, with different apparatuses, and within different sub‐fields of primate cognition. Whilst we have found that a reasonable proportion of studies now include zoo‐based research, we also hope that this grows. The potential of zoos to contribute to primate cognition research may still be somewhat overlooked, and some may not consider the attractive possibilities that conducting research in these settings offers. Zoos hold incredible potential for diverse studies, both in terms of species and topic diversity, and this review only covers some of the work that has already been done, not the limitations of what might be possible. Future work should consider the full range of possibilities offered by zoos, and how research‐focused zoos can further improve their research productivity and publication species diversity scores. Doing so will ensure that their impact on the field of primate cognition will continue to grow.

## AUTHOR CONTRIBUTIONS

Josep Call provided the initial concept. Emma S. McEwen managed the project. Emma S. McEwen, Elizabeth Warren, Sadie Tenpas, Kresimir Durdevic, and Josep Call developed the coding scheme. Emma S. McEwen, Elizabeth Warren, Sadie Tenpas, Kresimir Durdevic, Benjamin Jones, and Emilie Rapport Munro reviewed articles and collated data. Emma S. McEwen and Benjamin Jones processed and analyzed the data. Sadie Tenpas and Josep Call conducted the reliability analyses. Emma S. McEwen, Elizabeth Warren, Sadie Tenpas, Benjamin Jones, Emilie Rapport Munro, and Josep Call wrote the manuscript. All authors approved the final version of the manuscript for submission.

### 1 OPEN RESEARCH BADGES

This article has earned an Open Data badge for making publicly available the digitally‐shareable data necessary to reproduce the reported results. The data is available at https://osf.io/jf3tz/?view_only=3670701ac8624a4b87319ba7b131870e.

## Supporting information

Supporting information.

Supporting information.

Supporting information.

## Data Availability

The data that support the findings of this study are openly available on the OSF at: https://osf.io/jf3tz/?view_only=3670701ac8624a4b87319ba7b131870e
