# Supplementary material for: Primate cognition in zoos: Reviewing the impact of zoo‐based research over 15 years
Source: Am J Primatol. 2022 Mar 14;84(10):e23369. doi: 10.1002/ajp.23369 (PMC9786910; doi:10.1002/ajp.23369)
Supplement: Supplementary file 1 — Supporting information. [file AJP-84-e23369-s001.docx]

**Supplementary Materials**

*Development and use of the coding scheme*

Four reviewers (ESM, EW, ST, KD) worked collectively during an initial 8-week investigation period, in order to fully develop and finesse the coding scheme for data collection. Throughout the development of the coding scheme, and subsequently during its use in the main period of data collection, ambiguous cases occasionally arose, where the topic or method could not be easily categorized into one of the existing definitions. During the development period, these ambiguous cases were discussed between JC and the four reviewers and resulted in changes to the coding scheme to better ensure that it fully captured the diversity of methods used in primate cognition research. During the main data collection period, ambiguous cases were discussed amongst the reviewers, and the final decision for each item was decided by group consensus. If no consensus could be reached regarding apparatus or recording method, the final decision was made by ESM, and regarding inclusion or topic, the case was passed on to JC, who made the final decision. Following the finalization of the coding scheme, six reviewers (ESM, EW, ST, KD, BJ, ERM) worked independently to process each article; each year of each journal was randomly assigned to one of the six reviewers, who evaluated each article for inclusion, and then collected the relevant information from those articles to be included (see Table 2 in main text).

The list of topic areas and sub-topics was based on the table of contents of Primate Cognition (Tomasello and Call, 1997) (see below for details).

We included both *apparatus* and *experimenter intervention* in the coding scheme, to help us to decide when a study qualified as experimental or observational, and for the possibility of future investigations into aspects of methodology not captured by our *apparatus* variable, such as demonstrations from experimenters. We separated *apparatus* and *recording method* after realizing that just one variable was not enough to capture all methodology, and to separate what subjects interacted with and how their responses were recorded. For example, in a study in which a primate looked at shapes on a touchscreen and made selections with their fingers, subjects interacted with a monitor (the *apparatus*), and their responses were recorded by a touchscreen *(recording method*).

Changes were made, or new rules added, after coding had begun, in response to ambiguous cases that arose. For example, we had to decide whether Apparatus should be coded according to its *purpose*, or its *material*. We decided on the latter: for example, any time a subject had to make a choice between two objects, no matter the purpose of the task or what kind of object they were looking at, we defined it as ‘Object Options’. Whenever such an adjustment was made, previous articles were recoded to ensure that they fit with the change.

It became clear that the most difficult part would be deciding whether a paper qualified as ‘Cognition’ or not. We came across many studies that examined behaviors which seemed to involve cognitive processes, but did not explicitly address this cognitive engagement. We decided to only include studies that ask a question about cognition, rather than all studies whose primary behavioral focus included some form of cognitive engagement. Also, to aid us in determining whether or not a certain process was cognitive, we used the concepts of intentionality and flexibility as a guiding principle: if a behavior was intentional, and was flexibly applied across contexts, it could be counted as cognitive. We also posed ourselves the question of whether we should include studies investigating sensory perception in which cognition is only relevant in that it is required for the understanding of that perception; after discussions, we decided against the inclusion of these papers.

*Choice of journals and articles to code*

Originally, we selected 30 journals corresponding to the following areas: primatology, animal behavior, animal cognition, developmental science, physical anthropology, and general journals. When coding began, we realized that this was not feasible in the timeframe we had available for this project. We decided to cut down the number of journals we included in the review, so that we could include more years, giving us a more historical perspective on the impact of zoos upon primate cognition research. We therefore removed the more general and broader journals on our original list, such as Scientific Reports and Child Development, focusing instead upon journals that are specifically focused upon cognition or primates, with the inclusion of a few wider behavior journals. We looked at the past 15 years for 12 journals (American Journal of Primatology; International Journal of Primatology; Primates; Folia Primatologica; Journal of Comparative Psychology; Journal of Experimental Psychology: Animal Learning and Cognition; Animal Cognition; Behavioural Processes; Animal Behavior and Cognition; Animal Behaviour; Ethology; and Behaviour). We note that as we focused on major journals, this means that some work from some regions which may publish elsewhere (such as non-English language journals) was not included. Rather than using filters to identify relevant articles within our chosen journals, we decided to look individually at every single article from every issue, and identify and code those that focused both on primates and cognition. This was a purposeful decision, as we wanted to conduct an extremely thorough review, and although the use of keywords and filters would undoubtedly have made the process far quicker, we felt that we would have ended up with very different results, and would have lost much nuance and depth in doing so.

*Estimating the number of screened articles*

A list of all articles screened and excluded was not available. We thus estimated the number of items assessed (figure 1 in main text) from the 10% of issues included in our reliability assessment, for which these values were available. From the inclusion reliability analysis, there were 120 articles included, and 1605 articles excluded (using the original coder’s inclusion and exclusion decisions). This suggested that approximately 6.96% of all articles screened were included, and 93.04% were excluded. Using these percentages, and the final number of articles included in the review (1,119), this gave us an estimate of 16,077 screened articles, and 14,958 excluded articles.

*Topic areas*

Below is the full list of topics and sub-topics used for classification of individual papers, adapted from the table of contents in Tomasello and Call (1997).

1. Topic 1: Space and Objects
   1. Navigation and travel
      1. Spatial memory
      2. Navigation and travel
   2. Searching for hidden objects
      1. Delayed response
      2. Frames of reference
         1. Allocentric vs. egocentric frames of reference
         2. Landmark use and geometric cues
         3. Encoding space vs. features
      3. What is where
         1. Sortal object individuation
         2. Inferences
   3. Tracking object displacements
      1. Stage 5 visible displacements
      2. Stage 6 invisible displacements
      3. Transpositions and rotations
   4. Other forms of spatial understanding
      1. Mental rotation of object orientation
      2. Detours and mazes
      3. Target prediction
      4. Relational spatial mapping
         1. Container sets
         2. Scale models
2. Topic 2: Tools and Causality
   1. Object manipulation
      1. Object exploration
      2. Object properties
      3. Puzzle boxes and artificial fruits
      4. Food processing
      5. Drawing and symbolic play
   2. Tool-use
      1. Sequential tool-use
      2. Tool properties
      3. Tool modification and manufacture
      4. Innovation and creativity
      5. Insight, foresight and planning
   3. Causal understanding
      1. Tool-use
         1. Cloth problem
         2. String problem
         3. Stick problem
         4. Stick-and-trap problem
         5. Floating peanut and hook problems
      2. Causal inferences
         1. Visual cues: arrested motion, trails, balance beam
         2. Auditory cues: shaken cup
         3. Proprioceptive cues: pulling strings, lifting bottles
      3. Interventions: blicket detector
3. Topic 3: Features and Categories
   1. Discrimination Learning
      1. Learning sets
      2. Reversal learning and interference
      3. Conditional discrimination
      4. Cross-modal perception
   2. Natural Categories
      1. Concept formation
      2. Levels of abstraction
      3. Levels of representation
      4. Functional categories
   3. Relational Categories
      1. Identity
      2. Oddity
      3. Sameness/difference
      4. Analogies
         1. Features
         2. Spatial
         3. Size
   4. Classification
      1. Sorting after formal training
      2. Sorting after informal training
      3. Sorting without training
4. Topic 4: Quantities and Time
   1. Estimating Numerousness
      1. Absolute numerousness
      2. Relative numerousness
   2. Ordinality and Transitivity
      1. Monkeys
      2. Apes
   3. Counting, Summation, and Multiplication
      1. Counting
      2. Summation and Subtraction
      3. Multiplication
   4. Conservation of Quantities
      1. Monkeys
      2. Apes
   5. Proportions and probabilities
      1. Proportions
      2. Risk and uncertainty
      3. Probabilities
      4. Samples and populations
   6. Timing and discounting
      1. Time estimation
      2. Delayed gratification
      3. Temporal discounting
   7. Irrational choice
      1. Quantity estimation
         1. Reversed-reward contingency
         2. Less-is-more effect
         3. Visual illusions
      2. Investment and past payoffs
         1. Negative contrast effect
         2. Sunk cost effect
         3. Endowment effect
         4. Hot hand effect
5. Topic 5: Social Knowledge and Interaction
   1. The social field
      1. Knowledge of individuals
      2. Knowledge of behavior
      3. Knowledge of direct relationships
      4. Knowledge of third-party relationships
   2. Prosociality and helping
      1. Resource distribution
      2. Food donation
      3. Instrumental helping
   3. Reciprocity and interchange
      1. Reciprocity
      2. Interchange
      3. Bartering and exchange
      4. Trust
      5. Revenge, spite, and schadenfreude
   4. Social comparison
      1. Inequity aversion and fairness
      2. Image scoring and eavesdropping
      3. Reputation
   5. Coordination and collaboration
      1. Coalitions and alliances
         1. Functions
         2. Tactics
         3. Collective movements
      2. Chimpanzee cooperative hunting
      3. Cooperative problem-solving
         1. Identical roles
         2. Complementary roles
      4. Economic games
         1. Prisoner’s dilemma
         2. Stag hunt and assurance game
         3. Chicken and snowdrift
         4. Trust game
         5. Battle of the sexes
         6. Tragedy of the commons
         7. Ultimatum and dictator
6. Topic 6: Social Strategies and Communication
   1. Social Strategies: deception
      1. Active concealment
      2. Active misleading
      3. Counter-deception
      4. Experimental approaches
   2. Social strategies: social tool-use
   3. Intentional Communication: gestures
      1. Chimpanzees
      2. Other apes
      3. Monkeys
   4. Intentional Communication: vocalizations
      1. Vervet monkeys
      2. Other monkeys
      3. Lemurs
      4. Apes
   5. Communication with humans
      1. Gestural communication with humans
      2. Linguistic symbols
      3. Combinations of symbols
7. Topic 7: Social Learning and Culture
   1. Behavioral traditions in the wild
      1. Japanese macaque food-processing
      2. Chimpanzee tool-use
      3. Chimpanzee food choice
      4. Chimpanzee communication
      5. Capuchin tool-use
      6. Capuchin nose/eye poking
      7. Other primate species
   2. Social learning mechanisms
      1. Social facilitation
      2. Observational conditioning
      3. Observational learning
         1. Emulation
         2. Imitation
         3. Over-imitation
         4. Rational imitation
   3. Teaching
   4. Social learning strategies
      1. State-based biases
      2. Frequency-dependent biases
   5. Social transmission
      1. Corruption and conformity
8. Topic 8: Theory of Mind and Metacognition
   1. Understanding behavior, perception, and attention
   2. Understanding goals and intentions
   3. Understanding knowledge and beliefs
   4. Understanding self
      1. Mirrors
      2. Appearance-reality
      3. Metacognition
         1. Monitoring
         2. Control
         3. Confidence judgments

*Coding scheme*

Below are details of all of the elements coded by the reviewers, excluding the topic areas, which are detailed above.

The first step, once navigating to the journal issue for review, was to decide whether each article should be included. For this, we used the following procedure:

- Step 1: does the study include non-human primates?
- Step 2: is the study looking at an aspect of cognition?
  - Examples of things not included: handedness, personality, emotion
- Step 3: also exclude reviews, analyses of pre-existing data sets (only include cases in which they have collected data with primates)

| **Category** | **Items** |
| --- | --- |
| Authors |  |
| Title |  |
| Year |  |
| Journal | AJP, IJP, PRM, FOP, JCP, JEP: ALC, ANC, BEP, ABC, ANB, ETH, BEH |
| Reference |  |
| Abstract |  |
| Topic | (See above) |
| Species (new row for each species used within a study) | Common name (Latin name) |
| N | Sample size (not group size—if no sample size, put NA, and write group size in Comments).  If more than one group is tested (i.e., different species and/or across different site), list all sample sizes.  If more than one sample size is provided across experiments, but it is the same group of primates, use maximum number (i.e., if Experiment 1 had 9 subjects but one dropped out in Experiment 2, code as 9) |
| Methods | Observational, Experimental |
| Apparatus: what is used to present the stimuli/what did subjects interact with at test | - No apparatus (experimenter demonstration and no props, observational studies)   Example: a gaze-following study with no props   - Experimenter props (apparatus used/manipulated by experimenter, but not interacted with by subjects)   Example: a bucket covering experimenter’s head in a gaze following study   - Speaker   Example: playback studies   - Picture (physical, not on-screen) - Monitor   Examples: eye-tracking with computer screen, touchscreen   - Object options (Subjects select between items. For example, they are presented with two cups and must point at one to select it. Selections can be made by e.g., pointing/gaze/approach/reach. Subject’s actions serve only to make a selection. Includes go/no go e.g. reaching into box or not reaching into a box) Examples: violations of expectancy, working memory cup games - Token (subjects give something to another individual/an experimenter)   Examples: token exchange, passing a tool, food exchange, reciprocity   - Tools and trays (subjects use a tool/object to retrieve or process a reward, or interact with apparatus via a separate object. Includes pulling a rope or baited tray towards oneself, such as to move food closer) Example: Zone of latent solutions, cooperative tray pulling, cracking nuts with stones - Puzzle (physical apparatus that subjects physically manipulate. Subject’s actions used to manipulate/learn about apparatus. Includes mazes/arrays, considered large puzzles) Example: pressing a button to release juice from an apparatus, puzzle boxes, artificial fruits, trap door tasks |
| Experimenter intervention: was the experimenter’s behavior part of the stimuli? | - No (observation only) - Indirect (Experimenter facilitation e.g. apparatus is presented/manipulated, but experimenter behaviour is irrelevant (i.e., could be automated/moved with strings and should not influence subjects’ behaviour) - Direct (Experimenter behavior presentation e.g. experimenter pointing (beyond attention getting/encouraging participation)/display/gaze/body position/communication is important for the experimental manipulation) |
| Recording method: how was the data recorded (not incl. pen-and-paper or video camera)? | - Computer (e.g. computerized buttons) - Touchscreen - Joystick - Audio recording - Eye-tracking - GPS tracking |
| Additional | Anything that was required for the experimental question not yet coded (e.g. biological samples) |
| Location name |  |
| Location type | - Research center/university - Sanctuary (animals do not naturally live there, can be semi-free ranging) - Zoo - Field (free ranging primates in their natural habitat, including species reintroduces to certain areas) |
| Location country |  |
| Duplicate y/n | Duplicate rows were used when multiple species, apparatus types, recording materials, and/or experimenter intervention types were used within a study |
| Comments | Group size (where sample size not given), additional notes |

*Raw data tables*

1. Total numbers of species (divided into the major primate groups) that participated in studies included in this review, by site type

| Classification | Field | Research Center/University | Sanctuary | Zoo |
| --- | --- | --- | --- | --- |
| Ceboidea | 25 | 10 | 1 | 8 |
| Cercopithecidae | 26 | 17 | 4 | 19 |
| Hominidae | 5 | 4 | 3 | 4 |
| Hylobatidae | 1 | 0 | 7 | 8 |
| Prosimii | 11 | 12 | 4 | 6 |

1. Percentages of studies for each topic type per site type

| Location type | Features and Categories | Quant-ities and Time | Social Know-ledge and Inter-action | Social Learn-ing and Culture | Social Strategies and Communi-cation | Space and Objects | Theory of Mind and Metacog-nition | Tools and  Causality |
| --- | --- | --- | --- | --- | --- | --- | --- | --- |
| Field | 1.655629 | 1.324503 | 19.86755 | 19.53642 | 21.52318 | 15.56291 | 1.655629 | 29.4702 |
| Research Center/University | 20.69672 | 22.7459 | 20.08197 | 9.836066 | 8.811475 | 10.65574 | 12.09016 | 7.991803 |
| Sanctuary | 7.352941 | 2.941176 | 30.88235 | 23.52941 | 8.823529 | 10.29412 | 13.23529 | 26.47059 |
| Zoo | 11.11111 | 14.44444 | 19.62963 | 10.74074 | 10 | 11.11111 | 12.96296 | 18.88889 |

1. Topic area coefficient of variation per site type

| Location type | mean | SD | cv | max | min |  |
| --- | --- | --- | --- | --- | --- | --- |
| Field | 13.8245 | 10.88011 | 0.787016 | 29.4702 | 1.324503 |  |
| Research Center/University | 14.11373 | 6.016796 | 0.426308 | 22.7459 | 7.991803 |  |
| Sanctuary | 15.44118 | 10.15817 | 0.657862 | 30.88235 | 2.941176 |  |
| Zoo | 13.61111 | 3.760152 | 0.276256 | 19.62963 | 10 |  |

1. Proportions of observational and experimental studies per site type

| Location type | No. Experimental | No. Observational | % Experimental | % Observational |
| --- | --- | --- | --- | --- |
| Field | 70 | 232 | 23.17881 | 76.82119 |
| Research Center/University | 458 | 30 | 93.85246 | 6.147541 |
| Sanctuary | 53 | 15 | 77.94118 | 22.05882 |
| Zoo | 223 | 48 | 82.28782 | 17.71218 |

1. Numbers of studies using each apparatus type per site type

| Location type | Experi-menter Props | Monitor | No apparatus | Object Options | Picture | Puzzle | Speaker | Tokens | Tools and Trays |
| --- | --- | --- | --- | --- | --- | --- | --- | --- | --- |
| Field | 9 | 0 | 241 | 8 | 1 | 14 | 19 | 0 | 15 |
| Research Center/  University | 13 | 201 | 67 | 97 | 4 | 44 | 15 | 25 | 43 |
| Sanctuary | 5 | 4 | 23 | 12 | 0 | 11 | 1 | 2 | 17 |
| Zoo | 10 | 28 | 71 | 76 | 5 | 40 | 3 | 16 | 32 |

1. Numbers of studies using each recording method per site type

| Location type | Audio Recording | Computer | Eye-Tracking | GPS | Joystick | None | Other | Touchscreen |
| --- | --- | --- | --- | --- | --- | --- | --- | --- |
| Field | 23 | 0 | 0 | 27 | 0 | 254 | 2 | 0 |
| Research Center/  University | 5 | 11 | 15 | 0 | 73 | 294 | 1 | 93 |
| Sanctuary | 3 | 0 | 3 | 0 | 0 | 60 | 2 | 0 |
| Zoo | 4 | 1 | 3 | 0 | 0 | 245 | 0 | 19 |

1. Yearly number of new locations per site type (cumulative)

| Year | Field | Research Center/University | Sanctuary | Zoo |
| --- | --- | --- | --- | --- |
| 2006 | 17 | 16 | 1 | 8 |
| 2007 | 34 | 30 | 3 | 13 |
| 2008 | 43 | 33 | 4 | 29 |
| 2009 | 52 | 40 | 7 | 36 |
| 2010 | 57 | 45 | 10 | 44 |
| 2011 | 63 | 49 | 11 | 57 |
| 2012 | 67 | 50 | 12 | 64 |
| 2013 | 70 | 55 | 13 | 70 |
| 2014 | 83 | 62 | 15 | 78 |
| 2015 | 92 | 65 | 16 | 82 |
| 2016 | 97 | 68 | 18 | 86 |
| 2017 | 104 | 71 | 20 | 86 |
| 2018 | 110 | 75 | 20 | 90 |
| 2019 | 119 | 77 | 20 | 91 |
| 2020 | 128 | 81 | 21 | 95 |

1. Yearly number of articles per site type

| Year | Field | Research Center/University | Sanctuary | Zoo |
| --- | --- | --- | --- | --- |
| 2006 | 17 | 29 | 1 | 16 |
| 2007 | 19 | 37 | 2 | 11 |
| 2008 | 14 | 38 | 4 | 19 |
| 2009 | 24 | 38 | 3 | 20 |
| 2010 | 20 | 42 | 5 | 21 |
| 2011 | 16 | 34 | 6 | 27 |
| 2012 | 17 | 36 | 3 | 18 |
| 2013 | 18 | 33 | 4 | 21 |
| 2014 | 30 | 47 | 6 | 23 |
| 2015 | 22 | 30 | 14 | 20 |
| 2016 | 21 | 30 | 6 | 18 |
| 2017 | 22 | 21 | 1 | 12 |
| 2018 | 19 | 25 | 3 | 12 |
| 2019 | 19 | 26 | 5 | 12 |
| 2020 | 24 | 22 | 5 | 20 |

1. Yearly number of species studied per site type

| Year | Field | Research Center/University | Sanctuary | Zoo |
| --- | --- | --- | --- | --- |
| 2006 | 13 | 14 | 1 | 8 |
| 2007 | 14 | 12 | 2 | 8 |
| 2008 | 12 | 14 | 2 | 12 |
| 2009 | 15 | 11 | 5 | 8 |
| 2010 | 11 | 14 | 3 | 12 |
| 2011 | 11 | 17 | 8 | 13 |
| 2012 | 12 | 13 | 3 | 16 |
| 2013 | 11 | 14 | 4 | 9 |
| 2014 | 16 | 17 | 3 | 9 |
| 2015 | 10 | 18 | 9 | 9 |
| 2016 | 12 | 10 | 5 | 11 |
| 2017 | 11 | 8 | 1 | 7 |
| 2018 | 12 | 8 | 3 | 17 |
| 2019 | 14 | 17 | 2 | 7 |
| 2020 | 16 | 12 | 5 | 13 |
