# Supplementary material for: Primate cognition in zoos: Reviewing the impact of zoo‐based research over 15 years
Source: Am J Primatol. 2022 Mar 14;84(10):e23369. doi: 10.1002/ajp.23369 (PMC9786910; doi:10.1002/ajp.23369)
Supplement: Supplementary file 2 — Supporting information. [file AJP-84-e23369-s002.docx]

| Country | Location name | Location type | No. papers | Latitude | Longitude |
| --- | --- | --- | --- | --- | --- |
| Argentina | Centro de Investigaciones Ecológicas Subtropicales’ | Field | 1 | -25.6817 | -54.4457 |
| Argentina | Iguazú National Park | Field | 6 | -25.6785 | -54.4663 |
| Argentina | La Plata Zoo and Botanical Park | Zoo | 1 | -34.9094 | -57.9397 |
| Australia | Adelaide Zoo | Zoo | 1 | -34.9146 | 138.6061 |
| Australia | Perth Zoo | Zoo | 1 | -31.9754 | 115.8528 |
| Australia | Rockhampton Botanical and Zoological Gardens | Zoo | 4 | -23.398 | 150.491 |
| Australia | Symbio Wildlife Park | Zoo | 1 | -34.2055 | 150.9687 |
| Australia | University of New England | Research Center/University | 4 | -30.4901 | 151.6393 |
| Austria | Konrad Lorenz Institute for Evolution and Cognition | Research Center/University | 4 | 48.31087 | 16.32379 |
| Austria | Primate Sanctuary Gänserndorf | Sanctuary | 1 | 48.30662 | 16.71272 |
| Austria | University of Vienna | Research Center/University | 3 | 48.21303 | 16.3601 |
| Austria | Vienna Zoo | Zoo | 1 | 48.1821 | 16.3028 |
| Belgium | Planckendael Zoo | Zoo | 4 | 51 | 4.517606 |
| Bolivia | Department of the Pando, at the research camp “Callimico” | Field | 1 | -10.7989 | -66.9988 |
| Botswana | Moremi Game Reserve | Field | 1 | -19.3169 | 22.926 |
| Brazil | Atlantic Forest Reserve, Pernambuco | Field | 2 | -8.01028 | -35.0231 |
| Brazil | Atlantic Forest, Bahia State | Field | 1 | -12.2853 | -41.9295 |
| Brazil | Baracuhy Biological Field Station | Field | 1 | -7.52367 | -36.2917 |
| Brazil | Brasilia National Park | Field | 1 | -15.7391 | -47.9261 |
| Brazil | Caatinga biome | Field | 3 | -13.7487 | -41.1225 |
| Brazil | Camaragibe | Field | 1 | -8.0233 | -34.9945 |
| Brazil | Carlos Botelho State Park | Field | 2 | -24.1166 | -47.9832 |
| Brazil | Fazenda Boa Vista | Field | 12 | -5.48327 | -42.1667 |
| Brazil | Guarei (São Paulo) | Field | 1 | -23.3712 | -48.1844 |
| Brazil | Instituto Brasileiro de Meio Ambiente e dos Recursos Renovaveis | Research Center/University | 1 | -26.2425 | -48.6384 |
| Brazil | Laboratorio Tropical de Primatologia (LTP) | Research Center/University | 1 | -7.14569 | -34.8608 |
| Brazil | Mamiraua´ Sustainable Development Reserve | Field | 1 | -2.26667 | -65.6833 |
| Brazil | Morro da Glória | Field | 1 | -21.7482 | -43.3553 |
| Brazil | Nhumirim Farm | Field | 1 | -18.9833 | -56.6542 |
| Brazil | Poço das Antas Biological Reserve and Fazenda Afetiva-Jorge | Field | 1 | -22.544 | -42.2809 |
| Brazil | Porto Alegre | Field | 1 | -30.0325 | -51.2304 |
| Brazil | Primate Center of the University of Brasilia | Research Center/University | 1 | -16.5 | -46.5 |
| Brazil | Reserva Particular do Patrimônio Natural Santuário do Caraça | Field | 1 | -8.71043 | -36.2187 |
| Brazil | Serra da Capivara National Park | Field | 5 | -8.69685 | -42.5723 |
| Brazil | Serra das Confusões National Park | Field | 1 | -9.03733 | -43.5707 |
| Brazil | Tiete Ecological Park | Sanctuary | 3 | -23.4953 | -46.52 |
| Brazil | Unidade de Conservação Parque Estadual da Mata da Pimenteira | Field | 1 | -7.93552 | -38.3005 |
| Brazil | Zoobotanical Park (Federal University of Acre) | Field | 1 | -9.9561 | -67.8648 |
| Cameroon | Dja Biosphere Reserve | Field | 1 | 3 | 13 |
| Cameroon | Kagwene Mountain | Field | 1 | 6.127778 | 9.743056 |
| Canada | Toronto Zoo | Zoo | 8 | 43.8179 | -79.1854 |
| Central African Republic | Bai Hokou (Dzanga-Ndoki National Park) | Field | 3 | 2.405163 | 16.20172 |
| China | Beijing Normal University | Research Center/University | 1 | 39.962 | 116.3663 |
| China | Beijing Zoo | Zoo | 1 | 39.93913 | 116.3396 |
| China | Breeding Center for Endangered Animals in Beijing Wildlife Park | Zoo | 1 | 39.51721 | 116.3997 |
| China | Dalongtan site in Shennongjia Nature Reserve | Field | 1 | 31.49161 | 110.3043 |
| China | Francois’ Langur Breeding Center in Wuzhou | Research Center/University | 1 | 23.477 | 111.2791 |
| China | Mt. Huangshan National Reserve | Field | 6 | 30.12917 | 118.1469 |
| China | Nanwan Nature Reserve | Field | 1 | 18.39 | 109.98 |
| China | Yuhuangmiao area, Zhouzhi National Nature Reserve | Field | 1 | 30.57 | 105.46 |
| Costa Rica | El Zota Biological Field Station | Field | 1 | 10.5568 | -83.736 |
| Costa Rica | Lomas Barbudal Biological Reserve | Field | 1 | 10.506 | -85.3708 |
| Costa Rica | Santa Rosa National Park | Field | 1 | 10.8379 | -85.7051 |
| Côte d'Ivoire | Taï National Park | Field | 20 | 5.690011 | -6.93951 |
| Czech Republic | Liberec Zoo | Zoo | 1 | 50.7779 | 15.0809 |
| Czech Republic | Olomouc Zoo | Zoo | 1 | 49.6334 | 17.3434 |
| Democtratic Republic of Congo | Bili-Uéré region | Field | 2 | 4.537 | 25.332 |
| Democtratic Republic of Congo | Cuvette Centrale | Field | 1 | 0.5 | 18 |
| Democtratic Republic of Congo | Lola ya Bonobo Sanctuary | Sanctuary | 12 | -4.48994 | 15.26843 |
| Democtratic Republic of Congo | LuiKotale, Salonga National Park | Field | 5 | -1.75049 | 21.84464 |
| Democtratic Republic of Congo | Luo Scientific Reserve | Field | 3 | 0.1401 | 22.56 |
| Denmark | Givskud Zoo | Zoo | 2 | 55.80829 | 9.351685 |
| Ecuador | Proyecto Primates Research Site, Yasuni National Park | Field | 3 | -1.08333 | -75.9167 |
| Ecuador | Tiputini Biodiversity Station | Field | 1 | -0.63833 | -76.15 |
| Ethiopia | Awash National Park | Field | 1 | 9.0833 | 40 |
| France | Beauval Zoo | Zoo | 2 | 47.2477 | 1.3531 |
| France | Centre de Primatologie de l’Université de Strasbourg | Research Center/University | 23 | 48.6272 | 7.692843 |
| France | Centre National de la Recherche Scientifique Marseille | Research Center/University | 4 | 43.23159 | 5.440561 |
| France | La Boissière du Doré Zoo | Zoo | 1 | 47.24361 | 1.198417 |
| France | La Forêt des Singes | Zoo | 2 | 44.80417 | 1.633527 |
| France | La Palmyre Zoo | Zoo | 1 | 45.6867 | -1.1649 |
| France | La Vallée des Singes | Zoo | 4 | 46.24281 | 0.290174 |
| France | Ménagerie du Jardin des Plantes (National Museum of Natural History in Paris) | Zoo | 2 | 48.84211 | 2.356286 |
| France | Parc Zoologique & Botanique de Mulhouse | Zoo | 1 | 47.7317 | 7.3474 |
| France | Parc Zoologique de Thoiry | Zoo | 1 | 48.86419 | 1.797278 |
| France | Station Biologique de Paimpont, Université de Rennes | Research Center/University | 4 | 48.00393 | -2.22866 |
| France | Station de Primatologie of the Centre National de la Recherche Scientifique (CNRS), Rousset | Research Center/University | 20 | 43.48101 | 5.620889 |
| France | Université de Toulouse, Centre de recherche Cerveau et Cognition | Research Center/University | 1 | 43.60624 | 1.399761 |
| Gabon | Centre International de Recherche Medicale de Franceville | Research Center/University | 4 | -1.61505 | 13.58217 |
| Gabon | Loango National Park | Field | 1 | -2.1538 | 9.5896 |
| Gabon | Moukalaba-Doudou National Park | Field | 2 | -2.54734 | 10.40841 |
| Gabon | Pongara National Park and Wonga-Wongue Presidential Park | Field | 1 | -0.12 | 9.33 |
| Germany | Affenberg Salem | Zoo | 1 | 47.7626 | 9.2448 |
| Germany | Affenwald Wildlife Park | Research Center/University | 1 | 51.3948 | 10.7452 |
| Germany | Allwetterzoo Münster | Zoo | 2 | 51.94807 | 7.590139 |
| Germany | Berlin Zoo | Zoo | 3 | 52.50799 | 13.33776 |
| Germany | German Primate Center, Gottingen | Research Center/University | 4 | 51.56097 | 9.952895 |
| Germany | Institute of Zoology, (University of Veterinary Medicine, Hannover) | Research Center/University | 1 | 52.35483 | 9.801556 |
| Germany | Max Planck Institute for Biological Cybernetics | Research Center/University | 1 | 48.52362 | 9.053553 |
| Germany | NaturZoo, Rheine | Zoo | 2 | 52.29538 | 7.42465 |
| Germany | Nurnberg Zoo | Zoo | 1 | 49.4473 | 11.1451 |
| Germany | Tierpark Berlin | Zoo | 1 | 52.5023 | 13.5314 |
| Germany | Wilhelma Zoo | Zoo | 1 | 48.8042 | 9.208 |
| Germany | Wolfgang Köhler Primate Research Center, Leipzig Zoo | Zoo | 95 | 51.34854 | 12.37165 |
| Germany | Zoo Dortmund | Zoo | 1 | 51.4737 | 7.4683 |
| Guinea | Bossou | Field | 5 | 7.648746 | -8.50537 |
| Guinea | Seringbara Study Site, Nimba | Field | 3 | 7.630556 | -8.46242 |
| Guyana | Upper Essequibo Conservation Concession | Field | 1 | 5.65894 | -58.6041 |
| Hungary | Budapest Zoo | Zoo | 1 | 47.519 | 19.0776 |
| Hungary | Debrecen Zoo | Zoo | 1 | 47.5314 | 21.62598 |
| Hungary | Gyor Zoo | Zoo | 1 | 47.6947 | 17.6689 |
| Hungary | Jászberény Zoos | Zoo | 1 | 47.50305 | 19.91179 |
| Hungary | Nyíregyháza Zoo | Zoo | 1 | 48.0018 | 21.7238 |
| Hungary | Szeged Zoo | Zoo | 1 | 46.25463 | 20.1486 |
| India | Arunachala Hill | Field | 1 | 12.18 | 79.04 |
| India | Bandipur National Park | Field | 2 | 11.75857 | 76.44531 |
| India | Campbell Bay (Great Nicobar Island) | Field | 1 | 7.0076 | 93.9048 |
| India | Great Nicobar Biosphere Reserve | Field | 1 | 7.03 | 93.8 |
| Indonesia | Batu M'Belin (QBM) | Sanctuary | 4 | 3.37 | 98.5927 |
| Indonesia | Borneo Nature Foundation Natural Laboratory of Peat Swamp Forest (Palangkaraya) | Field | 1 | -2.20387 | 113.9011 |
| Indonesia | Danau Alo | Sanctuary | 1 | -0.9275 | 102.9447 |
| Indonesia | Kaja Island | Sanctuary | 2 | -2.85851 | 114.2153 |
| Indonesia | Ketambe research station | Field | 1 | 3.668574 | 97.74681 |
| Indonesia | Laboratorium Alam Hutan Gambut: Natural Laboratory for the Study of Peat Swamp Forest | Field | 1 | -2.31677 | 113.9081 |
| Indonesia | Nyaru Menteng Rescue Center | Sanctuary | 1 | -2.10944 | 113.8206 |
| Indonesia | Orangutan Care Center and Quarantine (OCCQ) | Sanctuary | 3 | -2.73231 | 111.6069 |
| Indonesia | Pasir Panjang | Sanctuary | 1 | 1.2761 | 103.7919 |
| Indonesia | Primate Research Center IPB | Research Center/University | 1 | -6.58533 | 106.8174 |
| Indonesia | Sacred Monkey Forest Sanctuary | Field | 1 | -8.5188 | 115.2586 |
| Indonesia | Suaq Balimbing Research area | Field | 1 | 3.066667 | 97.41667 |
| Indonesia | Sungai Lading in Central Kalimantan | Field | 1 | -1.6815 | 113.3824 |
| Indonesia | Tanjung Puting National Park (TPNP) | Field | 1 | -3.055 | 111.9184 |
| Indonesia | Tuanan, Central Kalimantan | Field | 6 | -2.15 | 114.4333 |
| Indonesia | Uluwatu Temple, Bali | Field | 2 | -8.8291 | 115.0849 |
| Iran | Kerman Neuroscience Research Cente | Research Center/University | 1 | 30.29241 | 57.06456 |
| Italy | Center of Primatology HSR | Research Center/University | 1 | 45.4668 | 9.1905 |
| Italy | Department of Biomedical and Specialty Surgical Sciences, University of Ferrara | Research Center/University | 1 | 44.83333 | 11.62611 |
| Italy | Department of Neuroscience, University of Parma. | Research Center/University | 1 | 44.80044 | 10.32553 |
| Italy | Instituto Superiore di Sanità | Research Center/University | 2 | 41.90414 | 12.51789 |
| Italy | Parco Faunistico di Piano dell’Abatino | Sanctuary | 4 | 42.2372 | 12.8364 |
| Italy | Parco Natura Viva-Garda Zoological Park | Zoo | 3 | 45.47981 | 10.79847 |
| Italy | Rome Zoo (Bioparco) | Zoo | 41 | 41.9169 | 12.4879 |
| Japan | Arashiyama, Kyoto Prefecture | Field | 2 | 35.0094 | 135.6668 |
| Japan | Awajishima Monkey Center | Field | 1 | 34.24502 | 134.8832 |
| Japan | Brain Science Institute of Tamagawa University (BSI) | Research Center/University | 1 | 35.56575 | 139.4634 |
| Japan | Fukuchiyama City Zoo | Zoo | 1 | 35.3107 | 135.1387 |
| Japan | Graduate School of Letters, Kyoto University | Research Center/University | 18 | 35.02624 | 135.7808 |
| Japan | Great Ape Research Institute (GARI) of Hayashibara Biochemical Laboratories | Research Center/University | 4 | 34.52884 | 133.9941 |
| Japan | Iwatayama Monkey Park | Field | 5 | 35.0114 | 135.6766 |
| Japan | Japan Monkey Centre, Inuyama | Zoo | 1 | 35.3889 | 136.9586 |
| Japan | Jigokudani Monkey Park, Nagano | Field | 2 | 36.7327 | 138.4621 |
| Japan | Katsuyama | Field | 2 | 36.0738 | 136.5264 |
| Japan | Keio University | Research Center/University | 1 | 35.649 | 139.7429 |
| Japan | Kinkazan Island | Field | 2 | 38.2971 | 141.5713 |
| Japan | Koshima island | Field | 2 | 31.45162 | 131.3759 |
| Japan | Kumamoto Sanctuary | Sanctuary | 7 | 32.64085 | 130.4825 |
| Japan | Kyoto City Zoo | Zoo | 1 | 35.0127 | 135.7864 |
| Japan | Minoo, Osaka Prefecture | Field | 1 | 34.8068 | 135.4423 |
| Japan | National Center of Neurology and Psychiatry | Research Center/University | 1 | 35.73719 | 139.4761 |
| Japan | National Institute for Neuroscience | Research Center/University | 1 | 35.72522 | 139.4766 |
| Japan | Primate Research Institute, Kyoto University | Research Center/University | 55 | 35.38797 | 136.9582 |
| Japan | RIKEN Center for Brain Science | Research Center/University | 1 | 35.77897 | 139.6128 |
| Japan | Takasakiyama Natural Zoo | Field | 1 | 33.2583 | 131.5328 |
| Japan | University of Toyama | Research Center/University | 1 | 36.69457 | 137.1866 |
| Japan | Wild Monkey Park Shodoshima Island | Field | 1 | 34.51789 | 134.243 |
| Japan | Yakushima Island | Field | 3 | 30.3446 | 130.5127 |
| Jersey | Durrell Zoo | Zoo | 1 | 49.2278 | -2.07539 |
| Kenya | Isecheno area of the Kakamega Forest | Field | 1 | 0.246947 | 34.87133 |
| Kenya | Laikipia Plateau | Field | 2 | 0.285845 | 36.82577 |
| Kenya | Mpala Research Centre | Field | 1 | 0.29234 | 36.89834 |
| Kenya | Sweetwaters Chimpanzee Sanctuary | Sanctuary | 2 | -0.00426 | 36.96367 |
| Liberia | Kpala, Yarpea-Mah Administrative District, Nimba County | Field | 1 | 7.17056 | -8.66444 |
| Madagascar | Ankarafa Forest | Field | 1 | -14.3768 | 47.77003 |
| Madagascar | Ankarafantsika National Park | Field | 3 | -16.2233 | 46.9417 |
| Madagascar | Berenty Reserve | Field | 1 | -25.005 | 46.3203 |
| Madagascar | Kirindy Forest | Field | 9 | -20.8167 | 44.15 |
| Madagascar | Ranomafana National Park | Field | 1 | -21.2641 | 47.4193 |
| Madagascar | Réserve Naturelle Intégrale Zahamena | Field | 1 | -17.7009 | 48.9192 |
| Malaysia | Taiping Zoo | Zoo | 1 | 4.855 | 100.751 |
| Mexico | Animaya Zoo | Zoo | 1 | 20.9825 | -89.6898 |
| Mexico | Centenario Zoo | Zoo | 2 | 20.9693 | -89.6402 |
| Mexico | Insti-tuto Nacional de Psiquiatría | Research Center/University | 1 | 19.2927 | -99.1524 |
| Mexico | Otoch Ma’ax Yetel Kooh Reserve | Field | 3 | 20.61667 | -87.6167 |
| Mexico | Putna Laguna | Field | 1 | 20.64583 | -87.6314 |
| Morocco | Ifrane National Park | Field | 4 | 33.56301 | -5.23715 |
| Morocco | La Carriere Toumliline | Field | 1 | 33.25 | -5.25 |
| Morocco | Middle Atlas Mountains of Morocco | Field | 2 | 31.06194 | -7.91611 |
| Netherlands | Apenheul Primate Park | Zoo | 10 | 52.21556 | 5.918901 |
| Netherlands | Biomedical Primate Research Centre in Rijswijk | Research Center/University | 6 | 52.0346 | 4.357697 |
| Netherlands | Ouwehands Zoo | Zoo | 1 | 51.9574 | 5.5903 |
| Netherlands | Royal Burgers Zoo | Zoo | 5 | 52.0102 | 5.9014 |
| Netherlands | Utrecht University | Research Center/University | 6 | 52.0852 | 5.1757 |
| Nigeria | Gashaka Gumti National Park | Field | 4 | 7.5424 | 11.6158 |
| Nigeria | Ngel Nyaki Forest Reserve | Field | 1 | 7.0876 | 11.0534 |
| Panama | Barro Colorado Island | Field | 1 | 9.1521 | -79.8465 |
| Panama | Jicarón Island (Coiba National Park) | Field | 1 | 7.4693 | -81.7568 |
| Peru | Cocha Cashu Biological Station in ManuNational Park | Field | 1 | -11.8882 | -71.4075 |
| Peru | La Estacion Biologica Los Amigos | Field | 1 | -12.5746 | -70.0674 |
| Poland | ZOO Lódz | Zoo | 1 | 51.76101 | 19.41259 |
| Poland | ZOO Poznan | Zoo | 1 | 52.39974 | 17.0058 |
| Poland | ZOO Warszawa | Zoo | 1 | 52.26352 | 21.01782 |
| Poland | ZOO Wroclaw | Zoo | 1 | 51.10604 | 17.07498 |
| Portugal | Lisbon Zoo | Zoo | 1 | 38.7444 | -9.1707 |
| Puerto Rico | Cayo Santiago | Field | 11 | 18.1564 | -65.7338 |
| Republic of Congo | Goualougo Triangle | Field | 3 | 3.05 | 16.9 |
| Republic of Congo | Likouala swamp | Field | 1 | 1.55447 | 17.16589 |
| Republic of Congo | Mbeli Bai | Field | 3 | 2.6195 | 16.6117 |
| Republic of Congo | Mondika Research Center | Field | 2 | 2.57269 | 16.58164 |
| Republic of Congo | Tchimpounga Sanctuary | Sanctuary | 7 | 51.66315 | 7.815817 |
| Rwanda | Karisoke Research Centre | Field | 1 | -1.49987 | 29.6315 |
| Rwanda | Nyungwe National Park | Field | 1 | -2.4812 | 29.2151 |
| Rwanda | Volcanoes National Park | Field | 3 | -1.4316 | 29.5689 |
| Senegal | Centre de Recherche de Primatologie (CRP) | Field | 1 | 12.95303 | -12.9391 |
| Senegal | Fongoli Savanah Chimpanzee Project | Field | 1 | 12.66667 | -12.2167 |
| Senegal | Niokolo Koba National Park | Field | 1 | 13.01 | -12.9448 |
| Singapore | Singapore Zoo | Zoo | 4 | 1.404627 | 103.7935 |
| South Africa | Blouberg Nature Reserve | Field | 5 | -23.0179 | 29.06915 |
| South Africa | Hogsback | Field | 1 | -32.5952 | 26.9323 |
| South Africa | Inkawu Vervet Project (IVP), Mawana Game Reserve in Kwazulu-Natal | Field | 3 | -28.0053 | 31.20408 |
| South Africa | Lajuma Research Centre in the Soutpansberg Mountains, Limpopo Province | Field | 1 | -23.1125 | 29.19364 |
| South Africa | Loskop Dam Nature Reserve | Field | 7 | -25.4244 | 29.38711 |
| South Africa | Mawana Game Reserve | Field | 2 | -28.0055 | 31.2059 |
| South Africa | Wild Animal Trauma Centre and Haven (WATCH) | Sanctuary | 1 | -27.7997 | 30.74111 |
| South Korea | Seoul Zoo | Zoo | 1 | 37.4275 | 127.017 |
| Spain | Barcelona Zoo | Zoo | 1 | 41.3864 | 2.1897 |
| Spain | Primate Rescue Centre Rainfer | Sanctuary | 1 | 40.4168 | -3.7038 |
| Spain | Universidad Autónoma de Madrid | Research Center/University | 1 | 40.5467 | -3.6944 |
| Spain | Valencia Zoo | Zoo | 1 | 39.478 | -0.4076 |
| Sri Lanka | Kaludiyapokuna forest reserve | Field | 1 | 7.867222 | 80.73361 |
| Suriname | Brownsberg Nature Park | Field | 1 | 4.9434 | -55.1707 |
| Suriname | Central Suriname Nature Preserve | Field | 2 | 4.1346 | -56.1142 |
| Sweden | Lund University Primate Research Station, Furuvik Zoo | Zoo | 3 | 60.65228 | 17.33649 |
| Sweden | The Astrid Fagræus laboratory (KM-F) | Research Center/University | 1 | 59.35155 | 18.03107 |
| Switzerland | Basel Zoo | Zoo | 2 | 47.54755 | 7.579246 |
| Switzerland | University of Zürich | Research Center/University | 9 | 47.3743 | 8.551 |
| Switzerland | Zoo al Maglio | Zoo | 1 | 45.9848 | 8.8769 |
| Switzerland | Zürich Zoo | Zoo | 5 | 47.38693 | 8.577611 |
| Tanzania | Gombe National Park | Field | 2 | -4.6983 | 29.6446 |
| Tanzania | Issa Valley, Ugalla | Field | 1 | -5.6 | 30.5 |
| Tanzania | Mahale Mountains National Park | Field | 8 | -6.2667 | 29.9333 |
| Thailand | Khao Sam Roi Yot National Park | Field | 3 | 12.1286 | 99.9537 |
| Thailand | Khao Yai National Park | Field | 4 | 14.4386 | 101.3723 |
| Thailand | Laemson National Park | Field | 1 | 9.6044 | 98.4674 |
| Thailand | Lobi Bay, Yao Noi Island (Ao Phang-Nga National Park) | Field | 1 | 8.3066 | 98.4577 |
| Thailand | Piak Nam Yai Island | Field | 2 | 9.604 | 98.466 |
| Thailand | Prang Sam Yot shrine | Field | 1 | 14.80278 | 100.6142 |
| Uganda | Budongo Forest Reserve | Field | 27 | 1.7941 | 31.5827 |
| Uganda | Bulindi | Field | 3 | 1.4663 | 31.4442 |
| Uganda | Bwindi Impenetrable National Park | Field | 3 | -1.0521 | 29.6201 |
| Uganda | Kalinzu Forest Central Reserve | Field | 1 | -0.4142 | 30.055 |
| Uganda | Kibale National Park | Field | 13 | 0.4862 | 30.3897 |
| Uganda | Lake Nabugabo | Field | 3 | -0.35591 | 31.91256 |
| Uganda | Ngamba Island Chimpanzee Sanctuary | Sanctuary | 13 | -0.1005 | 32.65282 |
| UK | Blair Drummond Safari Park | Zoo | 1 | 56.158 | -4.0439 |
| UK | Bristol Zoo | Zoo | 1 | 51.4637 | -2.622 |
| UK | Camperdown Wildlife Centre | Zoo | 1 | 56.4814 | -3.0416 |
| UK | Chester Zoo | Zoo | 2 | 53.2273 | -2.8844 |
| UK | Department of Physiology, Development and Neuroscience, University of Cambridge | Research Center/University | 1 | 52.2024 | 0.1228 |
| UK | Edinburgh Zoo | Zoo | 9 | 55.94531 | -3.26704 |
| UK | Fife Wildlife Park | Zoo | 1 | 56.2984 | -3.1573 |
| UK | Howletts Wild Animal Park | Zoo | 4 | 51.2698 | 1.1566 |
| UK | Marwell Wildlife Zoological Park | Zoo | 4 | 50.9911 | -1.2834 |
| UK | Medical Research Council Human Reproductive Sciences Unit | Research Center/University | 1 | 55.92133 | -3.12781 |
| UK | Monkey Haven | Sanctuary | 1 | 50.70579 | -1.26275 |
| UK | Paignton Zoo | Zoo | 3 | 50.4274 | -3.5827 |
| UK | Port Lympne animal park | Zoo | 2 | 51.0783 | 1.0007 |
| UK | Trentham Monkey Forest | Zoo | 2 | 52.95068 | -2.19839 |
| UK | Trotters World of Animals | Zoo | 1 | 54.68185 | -3.22692 |
| UK | Twycross Zoo | Zoo | 4 | 52.6524 | -1.5291 |
| UK | University of Stirling Primate Unit | Research Center/University | 1 | 56.14598 | -3.91835 |
| UK | Whipsnade Zoo | Zoo | 1 | 51.8502 | -0.5442 |
| UK | Woburn Safari Park | Zoo | 1 | 52.0114 | -0.5827 |
| USA | Alpha Genesis, Inc. | Research Center/University | 2 | 32.6801 | -80.8421 |
| USA | Animal Educators | Zoo | 1 | 33.29118 | -116.999 |
| USA | Bucknell University | Research Center/University | 9 | 40.95476 | -76.8851 |
| USA | Buffalo Zoo | Zoo | 4 | 42.93716 | -78.8516 |
| USA | California National Primate Research Center | Research Center/University | 3 | 38.53985 | -121.804 |
| USA | Callitrichid Research Center (CRC) at the University of Nebraska | Research Center/University | 1 | 41.25753 | -96.0055 |
| USA | Carleton College | Research Center/University | 4 | 44.46131 | -93.1558 |
| USA | Chimpanzee and Human Communication Institute | Research Center/University | 1 | 47.0073 | -120.536 |
| USA | Columbus Zoo and Aquarium | Zoo | 1 | 40.1562 | -83.118 |
| USA | Department of Psychology, 4S-105, College of Staten Island | Research Center/University | 1 | 40.5961 | -74.1496 |
| USA | Detroit Zoo | Zoo | 3 | 42.4768 | -83.149 |
| USA | Duke Lemur Center | Research Center/University | 14 | 35.9942 | -78.9623 |
| USA | Duke University | Research Center/University | 4 | 36.00191 | -78.9354 |
| USA | DuMond Conservancy for Primates and Tropical Forests, Monkey Jungle | Zoo | 1 | 25.56635 | -80.4314 |
| USA | Food and Drug Administration's National Center for Toxicological Research (NCTR) | Research Center/University | 1 | 34.36648 | -92.1128 |
| USA | Franklin and Marshall College | Research Center/University | 4 | 40.04881 | -76.3199 |
| USA | Gibbon Conservation Center | Sanctuary | 4 | 34.46538 | -118.47 |
| USA | Great Ape Trust, Iowa | Research Center/University | 3 | 41.5469 | -93.5323 |
| USA | Harvard University | Research Center/University | 7 | 42.377 | -71.1167 |
| USA | Infant primate research laboratory | Research Center/University | 1 | 47.65432 | -122.308 |
| USA | Jacksonville Zoo and Gardens | Zoo | 2 | 30.4022 | -81.6433 |
| USA | Johns Hopkins University | Research Center/University | 1 | 39.3299 | -76.6205 |
| USA | Kent State Primate facility | Research Center/University | 2 | 41.15167 | -81.3471 |
| USA | Language Research Center, Georgia State University | Research Center/University | 99 | 33.75307 | -84.3853 |
| USA | Lemur Conservation Foundation | Sanctuary | 1 | 27.40482 | -82.1063 |
| USA | Lincoln Park Zoo | Zoo | 12 | 41.92109 | -87.634 |
| USA | Lowry Park Zoo | Zoo | 1 | 28.01427 | -82.47 |
| USA | Michale E. Keeling Center for Comparative Medicine and Research | Research Center/University | 17 | 30.2098 | -97.3 |
| USA | Milwaukee County Zoo | Zoo | 2 | 43.0327 | -88.0377 |
| USA | Mobile Alabama Zoo | Zoo | 1 | 30.69436 | -88.0431 |
| USA | National Institute of Mental Health | Research Center/University | 3 | 39.05663 | -77.1203 |
| USA | National Institutes of Health Animal Center | Research Center/University | 5 | 39.12913 | -77.484 |
| USA | NEOMED Comparative Medicine Unit | Research Center/University | 1 | 41.10314 | -81.2449 |
| USA | New England Primate Research Center | Research Center/University | 4 | 42.32629 | -71.4987 |
| USA | New Iberia Primate Research Center | Research Center/University | 3 | 30.04278 | -91.8722 |
| USA | New York State Psychiatric Institute | Research Center/University | 5 | 40.8427 | -73.9438 |
| USA | Ohio State University | Research Center/University | 4 | 40.0067 | -83.0305 |
| USA | Penn State University | Research Center/University | 3 | 40.7982 | -77.8599 |
| USA | Rosamond Gifford Zoo | Zoo | 1 | 43.0433 | -76.1811 |
| USA | San Diego Wild Animal Park | Zoo | 4 | 33.09693 | -116.996 |
| USA | San Diego Zoo | Zoo | 3 | 32.73599 | -117.151 |
| USA | San Francisco Zoo | Zoo | 1 | 37.7331 | -122.505 |
| USA | Seneca Park Zoo | Zoo | 1 | 43.2051 | -77.6239 |
| USA | Smithsonian National Zoological Park | Zoo | 9 | 38.9299 | -77.0494 |
| USA | Southwest National Primate Research Center (Texas Biomedical Research Institute, San Antonio, Texas) | Research Center/University | 1 | 29.44018 | -98.6392 |
| USA | Staten Island Zoo | Zoo | 1 | 40.6251 | -74.1154 |
| USA | Suncoast Primate Sanctuary | Sanctuary | 1 | 28.1188 | -82.7635 |
| USA | Tampa Lowry Park Zoo | Zoo | 1 | 28.0138 | -82.47 |
| USA | The Gorilla Foundation | Research Center/University | 1 | 37.42837 | -122.251 |
| USA | University of Georgia | Research Center/University | 7 | 33.94798 | -83.3774 |
| USA | University of Iowa | Research Center/University | 2 | 41.6627 | -91.555 |
| USA | University of Louisiana | Research Center/University | 1 | 30.2114 | -92.0204 |
| USA | University of Massachusetts | Research Center/University | 3 | 42.3868 | -72.5301 |
| USA | University of Rochester | Research Center/University | 3 | 43.12984 | -77.6288 |
| USA | University of Texas Health Science Center at Houston | Research Center/University | 7 | 29.7032 | -95.4032 |
| USA | University of Wisconsin | Research Center/University | 6 | 43.0766 | -89.4125 |
| USA | Washington National Primate Research Centre | Research Center/University | 1 | 47.61772 | -122.355 |
| USA | Winsconsin National Primate Research Center (WNPRC) | Research Center/University | 1 | 43.06871 | -89.4069 |
| USA | Yale University | Research Center/University | 7 | 41.31178 | -72.9248 |
| USA | Yerkes National Primate Research Center (YNPRC) | Research Center/University | 62 | 33.80368 | -84.3186 |
| USA | Zoo Atlanta | Zoo | 6 | 33.73424 | -84.3719 |
| Venezuela | Guri Lake | Field | 2 | 7.0602 | -62.9677 |
| Zambia | Chimfunshi Wildlife Orphanage Trust | Sanctuary | 7 | -12.3814 | 27.5138 |
