# Supplementary material for: Primate cognition in zoos: Reviewing the impact of zoo‐based research over 15 years
Source: Am J Primatol. 2022 Mar 14;84(10):e23369. doi: 10.1002/ajp.23369 (PMC9786910; doi:10.1002/ajp.23369)
Supplement: Supplementary file 3 [file AJP-84-e23369-s003.odt]

References Included in Review

Abreu, F., Souto, A., & Schiel, N. (2020). Wild common marmosets (Callithrix jacchus) employ spatial cognitive abilities to improve their food search and consumption: an experimental approach in small-scale space. Primates, 61(6), 807–816. https://doi.org/10.1007/s10329-020-00826-1

Abzug, Z. M., & Sommer, M. A. (2018). Serial decision-making in monkeys during an oculomotor task. Journal of Experimental Psychology: Animal Learning and Cognition, 44(1), 95–102. https://doi.org/10.1037/xan0000154

Adachi, I., Anderson, J. R., & Fujita, K. (2011). Reverse-reward learning in squirrel monkeys (Saimiri sciureus): retesting after 5 years, and assessment on qualitative transfer. Journal of Comparative Psychology, 125(1), 84–90. https://doi.org/10.1037/a0021041

Adachi, I., & Fujita, K. (2007). Cross-modal representation of human caretakers in squirrel monkeys. Behavioural Processes, 74(1), 27–32. https://doi.org/10.1016/j.beproc.2006.09.004

Adams, D. B., & Kitchen, D. M. (2020). Model vs. playback experiments: The impact of sensory mode on predator-specific escape responses in saki monkeys. Ethology, 126(5), 563–575. https://doi.org/10.1111/eth.13008

Addessi, E., Chiarotti, F., Visalberghi, E., & Anzenberger, G. (2007). Response to novel food and the role of social influences in common marmosets (Callithrix jacchus) and Goeldi’s monkeys (Callimico goeldii). American Journal of Primatology, 69(11), 1210–1222. https://doi.org/10.1002/ajp.20429

Addessi, E., Crescimbene, L., & Visalberghi, E. (2008). Food and token quantity discrimination in capuchin monkeys (Cebus apella). Animal Cognition, 11(2), 275–282. https://doi.org/10.1007/s10071-007-0111-6

Addessi, E., Mancini, A., Crescimbene, L., Ariely, D., & Visalberghi, E. (2010). How to spend a token? Trade-offs between food variety and food preference in tufted capuchin monkeys (Cebus apella). Behavioural Processes, 83(3), 267–275. https://doi.org/10.1016/j.beproc.2009.12.012

Addessi, E., Mancini, A., Crescimbene, L., & Visalberghi, E. (2011). How social context, token value, and time course affect token exchange in capuchin monkeys (Cebus apella). International Journal of Primatology, 32(1), 83–98. https://doi.org/10.1007/s10764-010-9440-4

Addessi, E., Paglieri, F., Beran, M. J., Evans, T. A., Macchitella, L., De Petrillo, F., & Focaroli, V. (2013). Delay choice versus delay maintenance: Different measures of delayed gratification in capuchin monkeys (cebus apella). Journal of Comparative Psychology, 127(4), 392–398. https://doi.org/10.1037/a0031869

Adiseshan, A., Adiseshan, T., & Isbell, L. A. (2011). Affiliative relationships and reciprocity among adult male bonnet macaques (Macaca radiata) at Arunachala Hill, India. American Journal of Primatology, 73(11), 1107–1113. https://doi.org/10.1002/ajp.20987

Agrillo, C., Gori, S., & Beran, M. J. (2015). Do rhesus monkeys (Macaca mulatta) perceive illusory motion? Animal Cognition, 18(4), 895–910. https://doi.org/10.1007/s10071-015-0860-6

Agrillo, C., Parrish, A. E., & Beran, M. J. (2014). Do primates see the solitaire illusion differently? A comparative assessment of humans (Homo sapiens), chimpanzees (Pan troglodytes), rhesus monkeys (Macaca mulatta), and capuchin monkeys (Cebus apella). Journal of Comparative Psychology, 128(4), 402–413. https://doi.org/10.1037/a0037499

Albiach-Serrano, A., Bugnyar, T., & Call, J. (2012). Apes (Gorilla gorilla, Pan paniscus, P. troglodytes, Pongo abelii) versus corvids (Corvus corax, C. corone) in a support task: The effect of pattern and functionality. Journal of Comparative Psychology, 126(4), 355–367. https://doi.org/10.1037/a0028050

Albiach-Serrano, A., & Call, J. (2014). A reversed-reward contingency task reveals causal knowledge in chimpanzees (Pan troglodytes). Animal Cognition, 17(5), 1167–1176. https://doi.org/10.1007/s10071-014-0749-9

Albiach-Serrano, A., Call, J., & Barth, J. (2010). Great apes track hidden objects after changes in the objects’ position and in subject’s orientation. American Journal of Primatology, 72(4), 349–359. https://doi.org/10.1002/ajp.20790

Albiach-Serrano, A., Guillén-Salazar, F., & Call, J. (2007). Mangabeys (Cercocebus torquatus lunulatus) solve the reverse contingency task without a modified procedure. Animal Cognition, 10(4), 387–396. https://doi.org/10.1007/s10071-007-0076-5

Allanic, M., Hayashi, M., & Matsuzawa, T. (2020). Investigating the function of mutual grooming in captive bonobos (Pan paniscus) and chimpanzees (Pan troglodytes). Folia Primatologica, 91(5), 481–494. https://doi.org/10.1159/000506308

Allritz, M., Call, J., & Borkenau, P. (2016). How chimpanzees (Pan troglodytes) perform in a modified emotional Stroop task. Animal Cognition, 19(3), 435–449. https://doi.org/10.1007/s10071-015-0944-3

Allritz, M., Tennie, C., & Call, J. (2013). Food washing and placer mining in captive great apes. Primates, 54(4), 361–370. https://doi.org/10.1007/s10329-013-0355-5

Altschul, D., Terrace, H., & Weiss, A. (2016). Serial cognition and personality in macaques. Animal Behavior and Cognition, 3(1), 46–64. https://doi.org/10.12966/abc.02.04.2016

Amici, F., Aureli, F., & Call, J. (2014). Response facilitation in the four great apes: Is there a role for empathy? Primates, 55(1), 113–118. https://doi.org/10.1007/s10329-013-0375-1

Amici, F., Aureli, F., Mundry, R., Amaro, A. S., Barroso, A. M., Ferretti, J., & Call, J. (2014). Calculated reciprocity? A comparative test with six primate species. Primates, 55(3), 447–457. https://doi.org/10.1007/s10329-014-0424-4

Amici, F., Aureli, F., Visalberghi, E., & Call, J. (2009). Spider monkeys (Ateles geoffroyi) and capuchin monkeys (Cebus apella) follow gaze around barriers: evidence for perspective taking? Journal of Comparative Psychology, 123(4), 368–374. https://doi.org/10.1037/a0017079

Andersen, L. M., Basile, B. M., & Hampton, R. R. (2014). Dissociation of visual localization and visual detection in rhesus monkeys (Macaca mulatta). Animal Cognition, 17(3), 681–687. https://doi.org/10.1007/s10071-013-0699-7

Anderson, J. R., Bucher, B., Kuroshima, H., & Fujita, K. (2016). Evaluation of third-party reciprocity by squirrel monkeys (Saimiri sciureus) and the question of mechanisms. Animal Cognition, 19(4), 813–818. https://doi.org/10.1007/s10071-016-0980-7

Anderson, J. R., Hattori, Y., & Fujita, K. (2008). Quality Before Quantity: Rapid Learning of Reverse-Reward Contingency by Capuchin Monkeys (Cebus apella). Journal of Comparative Psychology, 122(4), 445–448. https://doi.org/10.1037/a0012624

Anderson, J. R., Hubert-Brierre, X., & McGrew, W. C. (2017). Reflections in the rainforest: full-length mirrors facilitate behavioral observations of unhabituated, wild chimpanzees. Primates, 58(1), 51–61. https://doi.org/10.1007/s10329-016-0574-7

Anderson, J. R., Kuroshima, H., & Fujita, K. (2010). Delay of Gratification in Capuchin Monkeys (Cebus apella) and Squirrel Monkeys (Saimiri sciureus). Journal of Comparative Psychology, 124(2), 205–210. https://doi.org/10.1037/a0018240

Anderson, J. R., Kuroshima, H., Hattori, Y., & Fujita, K. (2010). Flexibility in the use of requesting gestures in squirrel monkeys (Saimiri sciureus). American Journal of Primatology, 72(8), 707–714. https://doi.org/10.1002/ajp.20827

Anderson, J. R., Kuroshima, H., Paukner, A., & Fujita, K. (2009). Capuchin monkeys (Cebus apella) respond to video images of themselves. Animal Cognition, 12(1), 55–62. https://doi.org/10.1007/s10071-008-0170-3

Anderson, J. R., Kuwahata, H., & Fujita, K. (2007). Gaze alternation during “pointing” by squirrel monkeys (Saimiri sciureus)? Animal Cognition, 10(2), 267–271. https://doi.org/10.1007/s10071-006-0065-0

Anderson, M. R. (2012). Comprehension of object permanence and single transposition in gibbons. Behaviour, 149(3–4), 441–459. https://doi.org/10.1163/156853912X639769

Anderson, U. S., Stoinski, T. S., Bloomsmith, M. A., & Maple, T. L. (2007). Relative numerousness judgment and summation in young, middle-aged, and older adult orangutans (Pongo pygmaeus abelii and Pongo pygmaeus pygmaeus). Journal of Comparative Psychology, 121(1), 1–11. https://doi.org/10.1037/0735-7036.121.1.1

Annabelle Lee Cheong Lem, V., Harris, J. A., & Livesey, E. J. (2015). Testing the limits of the perruchet effect in choice response time tasks. Journal of Experimental Psychology: Animal Learning and Cognition, 41(4), 385–394. https://doi.org/10.1037/xan0000079

Arre, A. M., Clark, C. S., & Santos, L. R. (2020). Do young rhesus macaques know what others see?: A comparative developmental perspective. American Journal of Primatology, 82(11), 1–9. https://doi.org/10.1002/ajp.23054

Asensio, N., Brockelman, W. Y., Malaivijitnond, S., & Reichard, U. H. (2011). Gibbon travel paths are goal oriented. Animal Cognition, 14(3), 395–405. https://doi.org/10.1007/s10071-010-0374-1

Ash, H., Ziegler, T. E., & Colman, R. J. (2020). Early learning in the common marmoset (Callithrix jacchus): Behavior in the family group is related to preadolescent cognitive performance. American Journal of Primatology, 82(8). https://doi.org/10.1002/ajp.23159

Askew, J. A., & Morrogh-Bernard, H. C. (2017). Acoustic characteristics of long calls produced by male orang-utans (Pongo pygmaeus wurmbii): Advertising individual identity, context, and travel direction. Folia Primatologica, 87(5), 305–319. https://doi.org/10.1159/000452304

Atsumi, T., & Nagasaka, Y. (2015). Perception of chasing in squirrel monkeys (Saimiri sciureus). Animal Cognition, 18(6), 1243–1253. https://doi.org/10.1007/s10071-015-0893-x

Avdagic, E., Jensen, G., Altschul, D., & Terrace, H. S. (2014). Rapid cognitive flexibility of rhesus macaques performing psychophysical task-switching. Animal Cognition, 17(3), 619–631. https://doi.org/10.1007/s10071-013-0693-0

Balasubramaniam, K. N., Berman, C. M., Ogawa, H., & Li, J. (2011). Using biological markets principles to examine patterns of grooming exchange in Macaca thibetana. American Journal of Primatology, 73(12), 1269–1279. https://doi.org/10.1002/ajp.20999

Ban, S. D., Boesch, C., & Janmaat, K. R. L. (2014). Taï chimpanzees anticipate revisiting high-valued fruit trees from further distances. Animal Cognition, 17(6), 1353–1364. https://doi.org/10.1007/s10071-014-0771-y

Bandini, E., & Tennie, C. (2019). Individual acquisition of “stick pounding” behavior by naïve chimpanzees. American Journal of Primatology, 81(6). https://doi.org/10.1002/ajp.22987

Bania, A. E., Harris, S., Kinsley, H. R., & Boysen, S. T. (2009). Constructive and deconstructive tool modification by chimpanzees (Pan troglodytes). Animal Cognition, 12(1), 85–95. https://doi.org/10.1007/s10071-008-0173-0

Bania, A. E., & Stromberg, E. E. (2013). The effect of body orientation on judgments of human visual attention in western lowland gorillas (Gorilla gorilla gorilla). Journal of Comparative Psychology, 127(1), 82–90. https://doi.org/10.1037/a0027261

Barbet, I., & Fagot, J. (2011). Processing of Contour Closure by Baboons (Papio papio). Journal of Experimental Psychology: Animal Behavior Processes, 37(4), 407–419. https://doi.org/10.1037/a0025365

Bard, K. A. (2007). Neonatal imitation in chimpanzees (Pan troglodytes) tested with two paradigms. Animal Cognition, 10(2), 233–242. https://doi.org/10.1007/s10071-006-0062-3

Bard, K. A., Dunbar, S., Maguire-Herring, V., Veira, Y., Hayes, K. G., & Mcdonald, K. (2014). Gestures and social-emotional communicative development in chimpanzee infants. American Journal of Primatology, 76(1), 14–29. https://doi.org/10.1002/ajp.22189

Bard, K. A., Maguire-Herring, V., Tomonaga, M., & Matsuzawa, T. (2019). The gesture ‘Touch’: Does meaning-making develop in chimpanzees’ use of a very flexible gesture? Animal Cognition, 22(4), 535–550. https://doi.org/10.1007/s10071-017-1136-0

Barelli, C., Boesch, C., Heistermann, M., & Reichard, U. H. (2008). Female white-handed gibbons (Hylobates lar) lead group movements and have priority of access to food resources. Behaviour, 145(7), 965–981. https://doi.org/10.1163/156853908784089243

Barelli, C., Reichard, U. H., & Mundry, R. (2011). Is grooming used as a commodity in wild white-handed gibbons, Hylobates lar? Animal Behaviour, 82(4), 801–809. https://doi.org/10.1016/j.anbehav.2011.07.012

Barth, J., & Call, J. (2006). Tracking the displacement of objects: A series of tasks with great apes (Pan troglodytes, Pan paniscus, Gorilla gorilla, and Pongo pygmaeus) and young children (Homo sapiens). Journal of Experimental Psychology: Animal Behavior Processes, 32(3), 239–252. https://doi.org/10.1037/0097-7403.32.3.239

Basile, B. M., & Hampton, R. R. (2013). Monkeys show recognition without priming in a classification task. Behavioural Processes, 93, 50–61. https://doi.org/10.1016/j.beproc.2012.08.005

Basile, B. M., & Hampton, R. R. (2010). Rhesus monkeys (Macaca mulatta) show robust primacy and recency in memory for lists from small, but not large, image sets. Behavioural Processes, 83(2), 183–190. https://doi.org/10.1016/j.beproc.2009.12.013

Basile, B. M., Hampton, R. R., Suomi, S. J., & Murray, E. A. (2009). An assessment of memory awareness in tufted capuchin monkeys (Cebus apella). Animal Cognition, 12(1), 169–180. https://doi.org/10.1007/s10071-008-0180-1

Basile, B. M., Moylan, E. J., Charles, D. P., & Murray, E. A. (2015). Two-item same/different discrimination in rhesus monkeys (Macaca mulatta). Animal Cognition, 18(6), 1221–1230. https://doi.org/10.1007/s10071-015-0891-z

Bastian, M. L., Van Noordwijk, M. A., & Van Schaik, C. P. (2012). Innovative behaviors in wild Bornean orangutans revealed by targeted population comparison. Behaviour, 149(3–4), 275–297. https://doi.org/10.1163/156853912X636726

Beaune, D., Hohmann, G., Serckx, A., Sakamaki, T., Narat, V., & Fruth, B. (2017). How bonobo communities deal with tannin rich fruits: Re-ingestion and other feeding processes. Behavioural Processes, 142, 131–137. https://doi.org/10.1016/j.beproc.2017.06.007

Bebko, A. O., & Russon, A. E. (2015). Social Learning Opportunities in Captive Orangutans (Pongo abelii) and Mandrills (Mandrillus sphinx). International Journal of Primatology, 36(5), 1014–1035. https://doi.org/10.1007/s10764-015-9870-0

Beisner, B. A., & Mccowan, B. (2014). Signaling context modulates social function of silent bared-teeth displays in rhesus macaques (Macaca mulatta). American Journal of Primatology, 76(2), 111–121. https://doi.org/10.1002/ajp.22214

Beran, M. J., Perdue, B. M., Kelly, A. J., & Parrish, A. E. (2017). What’s in a face (made of foods)? Comparing children’s and monkey’s perception of faces in face-like images of food. Animal Behavior and Cognition, 4(3), 324–339. https://doi.org/10.26451/abc.04.03.10.2017

Beran, M. J. (2012). Quantity judgments of auditory and visual stimuli by Chimpanzees (Pan troglodytes). Journal of Experimental Psychology: Animal Behavior Processes, 38(1), 23–29. https://doi.org/10.1037/a0024965

Beran, M. J. (2011). Chimpanzees (Pan troglodytes) show the isolation effect during serial list recognition memory tests. Animal Cognition, 14(5), 637–645. https://doi.org/10.1007/s10071-011-0398-1

Beran, M. J. (2010). Use of exclusion by a chimpanzee (Pan troglodytes) during speech perception and auditory-visual matching-to-sample. Behavioural Processes, 83(3), 287–291. https://doi.org/10.1016/j.beproc.2010.01.009

Beran, M. J. (2010). Chimpanzees (Pan troglodytes) accurately compare poured liquid quantities. Animal Cognition, 13(4), 641–649. https://doi.org/10.1007/s10071-010-0314-0

Beran, M. J. (2008). Monkeys (Macaca mulatta and Cebus apella) Track, Enumerate, and Compare Multiple Sets of Moving Items. Journal of Experimental Psychology: Animal Behavior Processes, 34(1), 63–74. https://doi.org/10.1037/0097-7403.34.1.63

Beran, M. J. (2008). Capuchin monkeys (Cebus apella) succeed in a test of quantity conservation. Animal Cognition, 11(1), 109–116. https://doi.org/10.1007/s10071-007-0094-3

Beran, M. J. (2007). Rhesus monkeys (Macaca mulatta) succeed on a computerized test designed to assess conservation of discrete quantity. Animal Cognition, 10(1), 37–45. https://doi.org/10.1007/s10071-006-0028-5

Beran, M. J. (2007). Rhesus monkeys (Macaca mulatta) enumerate large and small sequentially presented sets of items using analog numerical representations. Journal of Experimental Psychology: Animal Behavior Processes, 33(1), 42–54. https://doi.org/10.1037/0097-7403.33.1.42

Beran, M. J., & Evans, T. A. (2012). Language-Trained Chimpanzees (Pan troglodytes) Delay Gratification by Choosing Token Exchange Over Immediate Reward Consumption. American Journal of Primatology, 74(9), 864–870. https://doi.org/10.1002/ajp.22042

Beran, M. J., & Evans, T. A. (2009). Delay of gratification by chimpanzees (Pan troglodytes) in working and waiting situations. Behavioural Processes, 80(2), 177–181. https://doi.org/10.1016/j.beproc.2008.11.008

Beran, M. J., & Evans, T. A. (2006). Maintenance of delay of gratification by four chimpanzees (Pan troglodytes): The effects of delayed reward visibility, experimenter presence, and extended delay intervals. Behavioural Processes, 73(3), 315–324. https://doi.org/10.1016/j.beproc.2006.07.005

Beran, M. J., Evans, T. A., & Hoyle, D. (2011). Numerical Judgments by Chimpanzees (Pan troglodytes) in a Token Economy. Journal of Experimental Psychology: Animal Behavior Processes, 37(2), 165–174. https://doi.org/10.1037/a0021472

Beran, M. J., Evans, T. A., Leighty, K. A., Harris, E. H., & Rice, D. (2008). Summation and quantity judgments of sequentially presented sets by capuchin monkeys (Cebus apella). American Journal of Primatology, 70(2), 191–194. https://doi.org/10.1002/ajp.20474

Beran, M. J., Evans, T. A., Paglieri, F., McIntyre, J. M., Addessi, E., & Hopkins, W. D. (2014). Chimpanzees (Pan troglodytes) can wait, when they choose to: A study with the hybrid delay task. Animal Cognition, 17(2), 197–205. https://doi.org/10.1007/s10071-013-0652-9

Beran, M. J., Evans, T. A., & Ratliff, C. L. (2009). Perception of Food Amounts by Chimpanzees (Pan troglodytes): The Role of Magnitude, Contiguity, and Wholeness. Journal of Experimental Psychology: Animal Behavior Processes, 35(4), 516–524. https://doi.org/10.1037/a0015488

Beran, M. J., French, K., Smith, T. R., & Parrish, A. E. (2019). Limited Evidence of Number-Space Mapping in Rhesus Monkeys (Macaca mulatta) and Capuchin Monkeys (Sapajus apella). Journal of Comparative Psychology. https://doi.org/10.1037/com0000177

Beran, M. J., Harris, E. H., Evans, T. A., Klein, E. D., Chan, B., Flemming, T. M., & Washburn, D. A. (2008). Ordinal Judgments of Symbolic Stimuli by Capuchin Monkeys (Cebus apella) and Rhesus Monkeys (Macaca mulatta): The Effects of Differential and Nondifferential Reward. Journal of Comparative Psychology, 122(1), 52–61. https://doi.org/10.1037/0735-7036.122.1.52

Beran, M. J., James, B. T., Whitham, W., & Parrish, A. E. (2016). Chimpanzees can point to smaller amounts of food to accumulate larger amounts but they still fail the reverse-reward contingency task. Journal of Experimental Psychology: Animal Learning and Cognition, 42(4), 347–358. https://doi.org/10.1037/xan0000115

Beran, M. J., McIntyre, J. M., Garland, A., & Evans, T. A. (2013). What counts for “counting”? Chimpanzees, Pan troglodytes, respond appropriately to relevant and irrelevant information in a quantity judgment task. Animal Behaviour, 85(5), 987–993. https://doi.org/10.1016/j.anbehav.2013.02.022

Beran, M. J., & Parrish, A. E. (2012). Sequential responding and planning in capuchin monkeys (Cebus apella). Animal Cognition, 15(6), 1085–1094. https://doi.org/10.1007/s10071-012-0532-8

Beran, M. J., Parrish, A. E., Futch, S. E., Evans, T. A., & Perdue, B. M. (2015). Looking ahead? Computerized maze task performance by chimpanzees (Pan troglodytes), rhesus monkeys (Macaca mulatta), capuchin monkeys (Cebus apella), and human children (Homo sapiens). Journal of Comparative Psychology, 129(2), 160–173. https://doi.org/10.1037/a0038936

Beran, M. J., Perdue, B. M., Church, B. A., & Smith, J. D. (2016). Capuchin monkeys (Cebus apella) modulate their use of an uncertainty response depending on risk. Journal of Experimental Psychology: Animal Learning and Cognition, 42(1), 32–43. https://doi.org/10.1037/xan0000080

Beran, M. J., Perdue, B. M., & David Smith, J. (2014). What are my chances? Closing the gap in uncertainty monitoring between rhesus monkeys (Macaca mulatta) and capuchin monkeys (Cebus apella). Journal of Experimental Psychology: Animal Behavior Processes, 40(3), 303–316. https://doi.org/10.1037/xan0000020

Beran, M. J., Perdue, B. M., Rossettie, M. S., James, B. T., Whitham, W., Walker, B., Futch, S. E., & Parrish, A. E. (2016). Self-control assessments of capuchin monkeys with the rotating tray task and the accumulation task. Behavioural Processes, 129, 68–79. https://doi.org/10.1016/j.beproc.2016.06.007

Beran, M. J., Rossettie, M. S., & Parrish, A. E. (2016). Trading up: chimpanzees (Pan troglodytes) show self-control through their exchange behavior. Animal Cognition, 19(1), 109–121. https://doi.org/10.1007/s10071-015-0916-7

Beran, M. J., Smith, J. D., Coutinho, M. V. C., Couchman, J. J., & Boomer, J. (2009). The Psychological Organization of “Uncertainty” Responses and “Middle” Responses: A Dissociation in Capuchin Monkeys (Cebus apella). Journal of Experimental Psychology: Animal Behavior Processes, 35(3), 371–381. https://doi.org/10.1037/a0014626

Berghänel, A., Ostner, J., Schröder, U., & Schülke, O. (2011). Social bonds predict future cooperation in male Barbary macaques, Macaca sylvanus. Animal Behaviour, 81(6), 1109–1116. https://doi.org/10.1016/j.anbehav.2011.02.009

Berman, C. M., Ionica, C., & Li, J. (2007). Supportive and tolerant relationships among male Tibetan macaques at Huangshan, China. Behaviour, 144(6), 631–661. https://doi.org/10.1163/156853907781347790

Bernstein-Kurtycz, L. M., Hopper, L. M., Ross, S. R., & Tennie, C. (2020). Zoo-Housed Chimpanzees Can Spontaneously Use Tool Sets But Perseverate on Previously Successful Tool-Use Methods. Animal Behavior and Cognition, 7(3), 288–309. https://doi.org/10.26451/abc.07.03.03.2020

Bettle, R., & Rosati, A. G. (2019). Flexible gaze-following in rhesus monkeys. Animal Cognition, 22(5), 673–686. https://doi.org/10.1007/s10071-019-01263-4

Bigelow, J., & Poremba, A. (2016). Audiovisual integration facilitates monkeys’ short-term memory. Animal Cognition, 19(4), 799–811. https://doi.org/10.1007/s10071-016-0979-0

Bigelow, J., & Poremba, A. (2013). Auditory memory in monkeys: Costs and benefits of proactive interference. American Journal of Primatology, 75(5), 425–434. https://doi.org/10.1002/ajp.22076

Blanchard, T. C., Wilke, A., & Hayden, B. Y. (2014). Hot-hand bias in rhesus monkeys. Journal of Experimental Psychology: Animal Behavior Processes, 40(3), 280–286. https://doi.org/10.1037/xan0000033

Bogart, S. L., & Pruetz, J. D. (2008). Ecological context of savanna chimpanzee (Pan troglodytes verus) termite fishing at Fongoli, Senegal. American Journal of Primatology, 70(6), 605–612. https://doi.org/10.1002/ajp.20530

Boggiani, L., Addessi, E., & Schino, G. (2018). Receiving aggression triggers attention bias in tufted capuchin monkeys. Animal Behaviour, 146, 173–180. https://doi.org/10.1016/j.anbehav.2018.10.021

Bohn, M., Call, J., & Tomasello, M. (2016). The role of past interactions in great apes’ communication about absent entities. Journal of Comparative Psychology, 130(4), 351–357. https://doi.org/10.1037/com0000042

Bonnie, K. E., Bernstein-Kurtycz, L. M., Shender, M. A., Ross, S. R., & Hopper, L. M. (2019). Foraging in a social setting: a comparative analysis of captive gorillas and chimpanzees. Primates, 60(2), 125–131. https://doi.org/10.1007/s10329-018-00712-x

Bonnie, K. E., & De Waal, F. B. M. (2007). Copying without rewards: Socially influenced foraging decisions among brown capuchin monkeys. Animal Cognition, 10(3), 283–292. https://doi.org/10.1007/s10071-006-0069-9

Bonnie, K. E., & de Waal, F. B. M. (2006). Affiliation promotes the transmission of a social custom: Handclasp grooming among captive chimpanzees. Primates, 47(1), 27–34. https://doi.org/10.1007/s10329-005-0141-0

Bonnie, K. E., Milstein, M. S., Calcutt, S. E., Ross, S. R., Wagner, K. E., & Lonsdorf, E. V. (2012). Flexibility and Persistence of Chimpanzee (Pan Troglodytes) Foraging Behavior in a Captive Environmentxht. American Journal of Primatology, 74(7), 661–668. https://doi.org/10.1002/ajp.22020

Boose, K. J., White, F. J., & Meinelt, A. (2013). Sex differences in tool use acquisition in bonobos (pan paniscus). American Journal of Primatology, 75(9), 917–926. https://doi.org/10.1002/ajp.22155

Borgeaud, C., & Bshary, R. (2018). Testing for anticipation of partners’ reciprocity and other social parameters: An experimental approach in Wild Vervet Monkeys (Chlorocebus pygerythrus). Journal of Comparative Psychology, 132(4), 464–472. https://doi.org/10.1037/com0000156

Botting, J. L., Wiper, M. L., & Anderson, J. R. (2011). Brown (Eulemur fulvus) and ring-tailed lemurs (Lemur catta) use human head orientation as a cue to gaze direction in a food choice task. Folia Primatologica, 82(3), 165–176. https://doi.org/10.1159/000333142

Botting, J., & Bastian, M. (2019). Orangutans (Pongo pygmaeus and Hybrid) and Gorillas (Gorilla gorilla gorilla) Modify Their Visual, but Not Auditory, Communicative Behaviors, Depending on the Attentional State of a Human Experimenter. International Journal of Primatology, 40(2), 244–262. https://doi.org/10.1007/s10764-019-00083-0

Botting, J., & Bastian, M. (2019). Examining the Use of Auditory Signals as “Attention-Getters” in Zoo-Housed Gorillas (Gorilla gorilla gorilla) and Orangutans (Pongo pygmaeus and Hybrid). International Journal of Primatology, 40(4–5), 573–586. https://doi.org/10.1007/s10764-019-00100-2

Botting, J., Whiten, A., Grampp, M., & van de Waal, E. (2018). Field experiments with wild primates reveal no consistent dominance-based bias in social learning. Animal Behaviour, 136, 1–12. https://doi.org/10.1016/j.anbehav.2017.11.025

Bouchet, H., Koda, H., & Lemasson, A. (2017). Age-dependent change in attention paid to vocal exchange rules in Japanese macaques. Animal Behaviour, 129, 81–92. https://doi.org/10.1016/j.anbehav.2017.05.012

Bourjade, M., Call, J., Pelé, M., Maumy, M., & Dufour, V. (2014). Bonobos and orangutans, but not chimpanzees, flexibly plan for the future in a token-exchange task. Animal Cognition, 17(6), 1329–1340. https://doi.org/10.1007/s10071-014-0768-6

Bourjade, M., Canteloup, C., Meguerditchian, A., Vauclair, J., & Gaunet, F. (2015). Training experience in gestures affects the display of social gaze in baboons’ communication with a human. Animal Cognition, 18(1), 239–250. https://doi.org/10.1007/s10071-014-0793-5

Bourjade, M., Gaunet, F., Maugard, A., & Meguerditchian, A. (2019). Manipulating social cues in baboon gesture learning: what does it tell us about the evolution of communication? Animal Cognition, 22(1), 113–125. https://doi.org/10.1007/s10071-018-1227-6

Bourjade, M., Meguerditchian, A., Maille, A., Gaunet, F., & Vauclair, J. (2014). Olive baboons, Papio anubis, adjust their visual and auditory intentional gestures to the visual attention of others. Animal Behaviour, 87(C), 121–128. https://doi.org/10.1016/j.anbehav.2013.10.019

Bourjade, M., Thierry, B., Call, J., & Dufour, V. (2012). Are monkeys able to plan for future exchange? Animal Cognition, 15(5), 783–795. https://doi.org/10.1007/s10071-012-0502-1

Bovet, D., & Deputte, B. L. (2009). Matching vocalizations to faces of familiar conspecifics in grey-cheeked mangabeys (Lophocebus albigena). Folia Primatologica, 80(3), 220–232. https://doi.org/10.1159/000235688

Bramlett, J. L., Perdue, B. M., Evans, T. A., & Beran, M. J. (2012). Capuchin monkeys (Cebus apella) let lesser rewards pass them by to get better rewards. Animal Cognition, 15(5), 963–969. https://doi.org/10.1007/s10071-012-0522-x

Brannon, E. M., Cantlon, J. F., & Terrace, H. S. (2006). The role of reference points in ordinal numerical comparisons by rhesus macaques (Macaca mulatto). Journal of Experimental Psychology: Animal Behavior Processes, 32(2), 120–134. https://doi.org/10.1037/0097-7403.32.2.120

Bräuer, J., & Call, J. (2015). Apes produce tools for future use. American Journal of Primatology, 77(3), 254–263. https://doi.org/10.1002/ajp.22341

Bräuer, J., & Call, J. (2011). The magic cup: Great apes and domestic dogs (Canis familiaris) individuate objects according to their properties. Journal of Comparative Psychology, 125(3), 353–361. https://doi.org/10.1037/a0023009

Bräuer, J., Call, J., & Tomasello, M. (2009). Are apes inequity averse? New data on the token-exchange paradigm. American Journal of Primatology, 71(2), 175–181. https://doi.org/10.1002/ajp.20639

Bräuer, J., Call, J., & Tomasello, M. (2008). Chimpanzees do not take into account what others can hear in a competitive situation. Animal Cognition, 11(1), 175–178. https://doi.org/10.1007/s10071-007-0097-0

Bräuer, J., Call, J., & Tomasello, M. (2007). Chimpanzees really know what others can see in a competitive situation. Animal Cognition, 10(4), 439–448. https://doi.org/10.1007/s10071-007-0088-1

Bray, J., Krupenye, C., & Hare, B. (2014). Ring-tailed lemurs (Lemur catta) exploit information about what others can see but not what they can hear. Animal Cognition, 17(3), 735–744. https://doi.org/10.1007/s10071-013-0705-0

Bril, B., Dietrich, G., Foucart, J., Fuwa, K., & Hirata, S. (2009). Tool use as a way to assess cognition: How do captive chimpanzees handle the weight of the hammer when cracking a nut? Animal Cognition, 12(2), 217–235. https://doi.org/10.1007/s10071-008-0184-x

Broihanne, M. H., Romain, A., Call, J., Thierry, B., Wascher, C. A. F., De Marco, A., & Verrier, D. (2018). Monkeys (Sapajus apella and Macaca tonkeana) and Great Apes (Gorilla gorilla, Pongo abelii, Pan paniscus, and Pan troglodytes) Play for the Highest Bid. Journal of Comparative Psychology. https://doi.org/10.1037/com0000153

Brosnan, S. F., & Beran, M. J. (2009). Trading Behavior Between Conspecifics in Chimpanzees, Pan troglodytes. Journal of Comparative Psychology, 123(2), 181–194. https://doi.org/10.1037/a0015092

Brosnan, S. F., & de Waal, F. B. M. (2009). Cebus apella tolerate intermittent unreliability in human experimenters. International Journal of Primatology, 30(5), 663–674. https://doi.org/10.1007/s10764-009-9366-x

Brosnan, S. F., Flemming, T., Talbot, C. F., Mayo, L., & Stoinski, T. (2011). Orangutans (Pongo pygmaeus) Do not form expectations based on their partner’s outcomes. Folia Primatologica, 82(1), 56–70. https://doi.org/10.1159/000328142

Brosnan, S. F., Freeman, C., & De Waal, F. B. M. (2006). Partner’s behavior, not reward distribution, determines success in an unequal cooperative task in capuchin monkeys. American Journal of Primatology, 68(7), 713–724. https://doi.org/10.1002/ajp.20261

Brosnan, S. F., Talbot, C., Ahlgren, M., Lambeth, S. P., & Schapiro, S. J. (2010). Mechanisms underlying responses to inequitable outcomes in chimpanzees, Pan troglodytes. Animal Behaviour, 79(6), 1229–1237. https://doi.org/10.1016/j.anbehav.2010.02.019

Brosnan, S. F., Silk, J. B., Henrich, J., Mareno, M. C., Lambeth, S. P., & Schapiro, S. J. (2009). Chimpanzees (Pan troglodytes) do not develop contingent reciprocity in an experimental task. Animal Cognition, 12(4), 587–597. https://doi.org/10.1007/s10071-009-0218-z

Brotcorne, F., Giraud, G., Gunst, N., Fuentes, A., Wandia, I. N., Beudels-Jamar, R. C., Poncin, P., Huynen, M. C., & Leca, J. B. (2017). Intergroup variation in robbing and bartering by long-tailed macaques at Uluwatu Temple (Bali, Indonesia). Primates, 58(4), 505–516. https://doi.org/10.1007/s10329-017-0611-1

Brotcorne, F., Holzner, A., Jorge-Sales, L., Gunst, N., Hambuckers, A., Wandia, I. N., & Leca, J. B. (2020). Social influence on the expression of robbing and bartering behaviours in Balinese long-tailed macaques. Animal Cognition, 23(2), 311–326. https://doi.org/10.1007/s10071-019-01335-5

Brown, E. K., Basile, B. M., Templer, V. L., & Hampton, R. R. (2019). Dissociation of memory signals for metamemory in rhesus monkeys (Macaca mulatta). Animal Cognition, 22(3), 331–341. https://doi.org/10.1007/s10071-019-01246-5

Brown, E. K., Templer, V. L., & Hampton, R. R. (2017). An assessment of domain-general metacognitive responding in rhesus monkeys. Behavioural Processes, 135, 132–144. https://doi.org/10.1016/j.beproc.2016.12.004

Brown, J., Kaplan, G., Rogers, L. J., & Vallortigara, G. (2010). Perception of biological motion in common marmosets (Callithrix jacchus): By females only. Animal Cognition, 13(3), 555–564. https://doi.org/10.1007/s10071-009-0306-0

Brunon, A., Bovet, D., Bourgeois, A., & Pouydebat, E. (2014). Motivation and manipulation capacities of the blue and yellow macaw and the tufted capuchin: A comparative approach. Behavioural Processes, 107, 1–14. https://doi.org/10.1016/j.beproc.2014.06.006

Bucher, B., Bourgeois, M., Anderson, J. R., Kuroshima, H., & Fujita, K. (2020). Investigating reactions of squirrel monkeys (Saimiri sciureus) towards unequal food distributions in a tray-pulling paradigm. Primates, 61(5), 717–727. https://doi.org/10.1007/s10329-020-00821-6

Bueno-Guerra, N., Völter, C. J., de las Heras, Á., Colell, M., & Call, J. (2019). Bargaining in Chimpanzees (Pan troglodytes): The Effect of Cost, Amount of Gift, Reciprocity, and Communication. Journal of Comparative Psychology, 133(4), 542. https://doi.org/10.1037/com0000189

Bulgheroni, M., Camperio-Ciani, A., Straulino, E., Sartori, L., D’Amico, E., & Castiello, U. (2017). Selective reaching in macaques: evidence for action-centred attention. Animal Cognition, 20(2), 359–366. https://doi.org/10.1007/s10071-016-1062-6

Bullinger, A. F., Burkart, J. M., Melis, A. P., & Tomasello, M. (2013). Bonobos, Pan paniscus, chimpanzees, Pan troglodytes, and marmosets, Callithrix jacchus, prefer to feed alone. Animal Behaviour, 85(1), 51–60. https://doi.org/10.1016/j.anbehav.2012.10.006

Bullinger, A. F., Melis, A. P., & Tomasello, M. (2014). Chimpanzees (Pan troglodytes) instrumentally help but do not communicate in a mutualistic cooperative task. Journal of Comparative Psychology, 128(3), 251–260. https://doi.org/10.1037/a0035645

Bullinger, A. F., Melis, A. P., & Tomasello, M. (2011). Chimpanzees, Pan troglodytes, prefer individual over collaborative strategies towards goals. Animal Behaviour, 82(5), 1135–1141. https://doi.org/10.1016/j.anbehav.2011.08.008

Bulloch, M. J., Boysen, S. T., & Furlong, E. E. (2008). Visual attention and its relation to knowledge states in chimpanzees, Pan troglodytes. Animal Behaviour, 76(4), 1147–1155. https://doi.org/10.1016/j.anbehav.2008.01.033

Burkart, J. M., & van Schaik, C. P. (2020). Marmoset prosociality is intentional. Animal Cognition, 23(3), 581–594. https://doi.org/10.1007/s10071-020-01363-6

Burkart, J. M., & Van Schaik, C. (2013). Group service in macaques (macaca fuscata), capuchins (cebus apella) and marmosets (callithrix jacchus): A comparative approach to identifying proactive prosocial motivations. Journal of Comparative Psychology, 127(2), 212–225. https://doi.org/10.1037/a0026392

Burkart, J., Kupferberg, A., Glasauer, S., & van Schaik, C. (2012). Even simple forms of social learning rely on intention attribution in marmoset monkeys (Callithrix jacchus). Journal of Comparative Psychology, 126(2), 129–138. https://doi.org/10.1037/a0026025

Buttelmann, D., Call, J., & Tomasello, M. (2008). Behavioral cues that great apes use to forage for hidden food. Animal Cognition, 11(1), 117–128. https://doi.org/10.1007/s10071-007-0095-2

Buttelmann, D., Carpenter, M., Call, J., & Tomasello, M. (2013). Chimpanzees, Pan troglodytes, recognize successful actions, but fail toimitate them. Animal Behaviour, 86(4), 755–761. https://doi.org/10.1016/j.anbehav.2013.07.015

Buttelmann, D., Schütte, S., Carpenter, M., Call, J., & Tomasello, M. (2012). Great apes infer others’ goals based on context. Animal Cognition, 15(6), 1037–1053. https://doi.org/10.1007/s10071-012-0528-4

Byrne, R. W., Hobaiter, C., & Klailova, M. (2011). Local traditions in gorilla manual skill: Evidence for observational learning of behavioral organization. Animal Cognition, 14(5), 683–693. https://doi.org/10.1007/s10071-011-0403-8

Byrnit, J. T. (2009). Gorillas’ (Gorilla gorilla) use of experimenter-given manual and facial cues in an object-choice task. Animal Cognition, 12(2), 401–404. https://doi.org/10.1007/s10071-008-0200-1

Byrnit, J. T., Høgh-Olesen, H., & Makransky, G. (2015). Share your sweets: Chimpanzee (Pan troglodytes) and bonobo (Pan paniscus) willingness to share highly attractive, monopolizable food sources. Journal of Comparative Psychology, 129(3), 218–228. https://doi.org/10.1037/a0039351

Cacchione, T., & Burkart, J. M. (2012). Dissociation between seeing and acting: Insights from common marmosets (Callithrix jacchus). Behavioural Processes, 89(1), 52–60. https://doi.org/10.1016/j.beproc.2011.10.010

Cacchione, T., Call, J., & Zingg, R. (2009). Gravity and Solidity in Four Great Ape Species (Gorilla gorilla, Pongo pygmaeus, Pan troglodytes, Pan paniscus): Vertical and Horizontal Variations of the Table Task. Journal of Comparative Psychology, 123(2), 168–180. https://doi.org/10.1037/a0013580

Cacchione, T., Hrubesch, C., & Call, J. (2013). Chimpanzees (Pan troglodytes) and bonobos (Pan paniscus) quantify split solid objects. Animal Cognition, 16(1), 1–10. https://doi.org/10.1007/s10071-012-0545-3

Cacchione, T., Hrubesch, C., Call, J., & Rakoczy, H. (2016). Are apes essentialists? Scope and limits of psychological essentialism in great apes. Animal Cognition, 19(5), 921–937. https://doi.org/10.1007/s10071-016-0991-4

Calcutt, S. E., Lonsdorf, E. V., Bonnie, K. E., Milstein, M. S., & Ross, S. R. (2014). Captive chimpanzees share diminishing resources. Behaviour, 151(14), 1967–1982. https://doi.org/10.1163/1568539X-00003225

Calcutt, S. E., Rubin, T. L., Pokorny, J. J., & de Waal, F. B. M. (2017). Discrimination of emotional facial expressions by tufted capuchin monkeys (Sapajus apella). Journal of Comparative Psychology, 131(1), 40–49. https://doi.org/10.1037/com0000055

Call, J. (2010). Do apes know that they could be wrong? Animal Cognition, 13(5), 689–700. https://doi.org/10.1007/s10071-010-0317-x

Call, J. (2006). Inferences by exclusion in the great apes: The effect of age and species. Animal Cognition, 9(4), 393–403. https://doi.org/10.1007/s10071-006-0037-4

Campbell, L. A. D., Tkaczynski, P. J., Mouna, M., Qarro, M., Waterman, J., & Majolo, B. (2016). Behavioral responses to injury and death in wild Barbary macaques (Macaca sylvanus). Primates, 57(3), 309–315. https://doi.org/10.1007/s10329-016-0540-4

Campbell, M. W., Watzek, J., Suchak, M., Berman, S. M., & de Waal, F. B. M. (2020). Chimpanzees (Pan troglodytes) tolerate some degree of inequity while cooperating but refuse to donate effort for nothing. American Journal of Primatology, 82(1). https://doi.org/10.1002/ajp.23084

Campennì, M., Manciocco, A., Vitale, A., & Schino, G. (2015). Exchanging grooming, but not tolerance and aggression in common marmosets (Callithrix jacchus). American Journal of Primatology, 77(2), 222–228. https://doi.org/10.1002/ajp.22324

Canale, G. R., Guidorizzi, C. E., Kierulff, M. C. M., & Gatto, C. A. F. R. (2009). First record of tool use by wild populations of the yellow-breasted capuchin monkey (Cebus xanthosternos) and new records for the bearded capuchin (cebus libidinosus). American Journal of Primatology, 71(5), 366–372. https://doi.org/10.1002/ajp.20648

Candiotti, A., Zuberbühler, K., & Lemasson, A. (2013). Voice discrimination in four primates. Behavioural Processes, 99, 67–72. https://doi.org/10.1016/j.beproc.2013.06.010

Cangiano, M., & Palagi, E. (2020). First evidence of stone handling in geladas: From simple to more complex forms of object play. Behavioural Processes, 180, 104253. https://doi.org/10.1016/j.beproc.2020.104253

Canteloup, C., Bovet, D., & Meunier, H. (2015). Intentional gestural communication and discrimination of human attentional states in rhesus macaques (Macaca mulatta). Animal Cognition, 18(4), 875–883. https://doi.org/10.1007/s10071-015-0856-2

Canteloup, C., Bovet, D., & Meunier, H. (2015). Do Tonkean macaques (Macaca tonkeana) tailor their gestural and visual signals to fit the attentional states of a human partner? Animal Cognition, 18(2), 451–461. https://doi.org/10.1007/s10071-014-0814-4

Cantlon, J. F., & Brannon, E. M. (2007). How much does number matter to a monkey (Macaca mulatta)? Journal of Experimental Psychology: Animal Behavior Processes, 33(1), 32–41. https://doi.org/10.1037/0097-7403.33.1.32

Cantlon, J. F., Merritt, D. J., & Brannon, E. M. (2016). Monkeys display classic signatures of human symbolic arithmetic. Animal Cognition, 19(2), 405–415. https://doi.org/10.1007/s10071-015-0942-5

Carducci, P., Squillace, V., Manzi, G., & Truppa, V. (2020). Touch improves visual discrimination of object features in capuchin monkeys (Sapajus spp.). Behavioural Processes, 172, 104044. https://doi.org/10.1016/j.beproc.2020.104044

Carne, C., Wiper, S., & Semple, S. (2011). Reciprocation and interchange of grooming, agonistic support, feeding tolerance, and aggression in semi-free-ranging Barbary macaques. American Journal of Primatology, 73(11), 1127–1133. https://doi.org/10.1002/ajp.20979

Carrasco, L., Posada, S., & Colell, M. (2009). New Evidence on Imitation in an Enculturated Chimpanzee (Pan troglodytes). Journal of Comparative Psychology, 123(4), 385–390. https://doi.org/10.1037/a0016275

Cartmill, E. A., & Byrne, R. W. (2010). Semantics of primate gestures: Intentional meanings of orangutan gestures. Animal Cognition, 13(6), 793–804. https://doi.org/10.1007/s10071-010-0328-7

Carvalho, S., Biro, D., McGrew, W. C., & Matsuzawa, T. (2009). Tool-composite reuse in wild chimpanzees (Pan troglodytes): Archaeologically invisible steps in the technological evolution of early hominins? Animal Cognition, 12(1 SUPPL), 103–114. https://doi.org/10.1007/s10071-009-0271-7

Casanova, C., Mondragon-Ceballos, R., & Lee, P. C. (2008). Innovative social behavior in chimpanzees (Pan troglodytes). American Journal of Primatology, 70(1), 54–61. https://doi.org/10.1002/ajp.20456

Cäsar, C., Byrne, R. W., Hoppitt, W., Young, R. J., & Zuberbühler, K. (2012). Evidence for semantic communication in titi monkey alarm calls. Animal Behaviour, 84(2), 405–411. https://doi.org/10.1016/j.anbehav.2012.05.010

Casey, A. H., Silberberg, A., Paukner, A., & Suomi, S. J. (2014). Defining reward value by cross-modal scaling. Animal Cognition, 17(2), 177–183. https://doi.org/10.1007/s10071-013-0650-y

Cash-Padgett, T., Azab, H., Yoo, S. B. M., & Hayden, B. Y. (2018). Opposing pupil responses to offered and anticipated reward values. Animal Cognition, 21(5), 671–684. https://doi.org/10.1007/s10071-018-1202-2

Chapman, K. M., & Weiss, D. J. (2013). Pulling to scale: Motor planning for sequences of repeated actions by cotton-top Tamarins (Saguinus oedipus). Journal of Experimental Psychology: Animal Behavior Processes, 39(2), 180–186. https://doi.org/10.1037/a0031775

Charpentier, M. J. E., Harté, M., Ngoubangoye, B., Herbert, A., & Kappeler, P. M. (2017). Visual Discrimination of Kin in Mandrills. Ethology, 123(3), 251–259. https://doi.org/10.1111/eth.12596

Chelonis, J. J., Cox, A. R., Karr, M. J., Prunty, P. K., Baldwin, R. L., & Paule, M. G. (2014). Comparison of delayed matching-to-sample performance in monkeys and children. Behavioural Processes, 103, 261–268. https://doi.org/10.1016/j.beproc.2014.01.002

Chen, T., Gao, J., Tan, J., Tao, R., & Su, Y. (2017). Variation in gaze-following between two Asian colobine monkeys. Primates, 58(4), 525–534. https://doi.org/10.1007/s10329-017-0612-0

Chertoff, S., Margulis, S., & Rodgers, J. D. (2018). Visual Processing of Faces in Juvenile Western Lowland Gorillas without the Use of Training or Reinforcement: A Pilot Study. Animal Behavior and Cognition, 5(3), 292–299. https://doi.org/10.26451/abc.05.03.04.2018

Clara, E., Regolin, L., Vallortigara, G., & Rogers, L. (2007). Perception of the stereokinetic illusion by the common marmoset (Callithrix jacchus). Animal Cognition, 10(2), 135–140. https://doi.org/10.1007/s10071-006-0046-3

Clara, E., Tommasi, L., & Rogers, L. J. (2008). Social mobbing calls in common marmosets (Callithrix jacchus): Effects of experience and associated cortisol levels. Animal Cognition, 11(2), 349–358. https://doi.org/10.1007/s10071-007-0125-0

Clark, F. E., & Smith, L. J. (2013). Effect of a cognitive challenge device containing food and non-food rewards on chimpanzee well-being. American Journal of Primatology, 75(8), 807–816. https://doi.org/10.1002/ajp.22141

Clay, Z., Ravaux, L., De Waal, F. B. M., & Zuberbüehler, K. (2016). Bonobos (Pan paniscus) vocally protest against violations of social expectations. Journal of Comparative Psychology, 130(1), 44–54. https://doi.org/10.1037/a0040088

Clay, Z., & Zuberbühler, K. (2011). The Structure of Bonobo Copulation Calls During Reproductive and Non-Reproductive Sex. Ethology, 117(12), 1158–1169. https://doi.org/10.1111/j.1439-0310.2011.01975.x

Clay, Z., & Zuberbühler, K. (2009). Food-associated calling sequences in bonobos. Animal Behaviour, 77(6), 1387–1396. https://doi.org/10.1016/j.anbehav.2009.02.016

Close, J., & Call, J. (2015). From colour photographs to black-and-white line drawings: an assessment of chimpanzees’ (Pan troglodytes’) transfer behaviour. Animal Cognition, 18(2), 437–449. https://doi.org/10.1007/s10071-014-0813-5

Coelho, C. G., Falótico, T., Izar, P., Mannu, M., Resende, B. D., Siqueira, J. O., & Ottoni, E. B. (2015). Social learning strategies for nut-cracking by tufted capuchin monkeys (Sapajus spp.). Animal Cognition, 18(4), 911–919. https://doi.org/10.1007/s10071-015-0861-5

Cohen, P. M., & Santos, L. R. (2017). Capuchins (Cebus apella) fail to show an asymmetric dominance effect. Animal Cognition, 20(2), 331–345. https://doi.org/10.1007/s10071-016-1055-5

Collier-Baker, E., Davis, J. M., Nielsen, M., & Suddendorf, T. (2006). Do chimpanzees (Pan troglodytes) understand single invisible displacement? Animal Cognition, 9(1), 55–61. https://doi.org/10.1007/s10071-005-0004-5

Collier-Baker, E., & Suddendorf, T. (2006). Do chimpanzees (Pan troglodytes) and 2-year-old children (Homo sapiens) understand double invisible displacement? Journal of Comparative Psychology, 120(2), 89–97. https://doi.org/10.1037/0735-7036.120.2.89

Corat, C., Siqueira, J., & Ottoni, E. B. (2016). Sequential organization and optimization of the nut-cracking behavior of semi-free tufted capuchin monkeys (Sapajus sp.). Primates, 57(1), 113–121. https://doi.org/10.1007/s10329-015-0491-1

Cords, M., & Nikitopoulos, E. (2015). Maternal kin bias in affiliative behavior among wild adult female blue monkeys. American Journal of Primatology, 77(1), 109–123. https://doi.org/10.1002/ajp.22315

Corp, N., Hayaki, H., Matsusaka, T., Fujita, S., Hosaka, K., Kutsukake, N., Nakamura, M., Nakamura, M., Nishie, H., Shimada, M., Zamma, K., Wallauer, W., & Nishida, T. (2009). Prevalence of muzzle-rubbing and hand-rubbing behavior in wild chimpanzees in Mahale Mountains National Park, Tanzania. Primates, 50(2), 184–189. https://doi.org/10.1007/s10329-008-0126-x

Costes-Thiré, M., Levé, M., Uhlrich, P., De Marco, A., & Thierry, B. (2015). Lack of evidence that Tonkean macaques understand what others can hear. Animal Cognition, 18(1), 251–258. https://doi.org/10.1007/s10071-014-0795-3

Costes-Thiré, M., Levé, M., Uhlrich, P., Pasquaretta, C., Marco, A. De, & Thierry, B. (2015). Evidence that monkeys (Macaca tonkeana and Sapajus apella) read moves, but no evidence that they read goals. Journal of Comparative Psychology, 129(3), 304–310. https://doi.org/10.1037/a0039294

Couchman, J. J. (2015). Humans and monkeys distinguish between self-generated, opposing, and random actions. Animal Cognition, 18(1), 231–238. https://doi.org/10.1007/s10071-014-0792-6

Couchman, J. J., Coutinho, M. V. C., Beran, M. J., & Smith, J. D. (2010). Beyond stimulus cues and reinforcement signals: A new approach to animal metacognition. Journal of Comparative Psychology, 124(4), 356–368. https://doi.org/10.1037/a0020129

Couchman, J. J., Coutinho, M. V. C., & Smith, J. D. (2010). Rules and Resemblance: Their Changing Balance in the Category Learning of Humans (Homo sapiens) and Monkeys (Macaca mulatta). Journal of Experimental Psychology: Animal Behavior Processes, 36(2), 172–183. https://doi.org/10.1037/a0016748

Coye, C., Ouattara, K., Arlet, M. E., Lemasson, A., & Zuberbühler, K. (2018). Flexible use of simple and combined calls in female Campbell’s monkeys. Animal Behaviour, 141, 171–181. https://doi.org/10.1016/j.anbehav.2018.05.014

Coye, C., Zuberbühler, K., & Lemasson, A. (2016). Morphologically structured vocalizations in female Diana monkeys. Animal Behaviour, 115, 97–105. https://doi.org/10.1016/j.anbehav.2016.03.010

Crast, J., Hardy, J. M., & Fragaszy, D. (2010). Inducing traditions in captive capuchin monkeys (Cebus apella). Animal Behaviour, 80(6), 955–964. https://doi.org/10.1016/j.anbehav.2010.08.023

Crockford, C., Wittig, R. M., & Zuberbühler, K. (2015). An intentional vocalization draws others’ attention: A playback experiment with wild chimpanzees. Animal Cognition, 18(3), 581–591. https://doi.org/10.1007/s10071-014-0827-z

Cronin, K. A., & Snowdon, C. T. (2008). The effects of unequal reward distributions on cooperative problem solving by cottontop tamarins, Saguinus oedipus. Animal Behaviour, 75(1), 245–257. https://doi.org/10.1016/j.anbehav.2007.04.032

Cronin, K. A., van Leeuwen, E. J. C., Mulenga, I. C., & Bodamer, M. D. (2011). Behavioral response of a chimpanzee mother toward her dead infant. American Journal of Primatology, 73(5), 415–421. https://doi.org/10.1002/ajp.20927

Cunningham, C. L., Anderson, J. R., & Mootnick, A. R. (2006). Object manipulation to obtain a food reward in hoolock gibbons, Bunopithecus hoolock. Animal Behaviour, 71(3), 621–629. https://doi.org/10.1016/j.anbehav.2005.05.013

Cunningham, C., Anderson, J., & Mootnick, A. (2011). A sex difference in effect of prior experience on object-mediated problem-solving in gibbons. Animal Cognition, 14(4), 599–605. https://doi.org/10.1007/s10071-011-0380-y

Cunningham, E. P., & Janson, C. H. (2013). Effect of Fruit Scarcity on Use of Spatial Memory in a Seed Predator, White-faced Saki (Pithecia pithecia). International Journal of Primatology, 34(4), 808–822. https://doi.org/10.1007/s10764-013-9697-5

Cunningham, E., & Janson, C. (2007). Integrating information about location and value of resources by white-faced saki monkeys (Pithecia pithecia). Animal Cognition, 10(3), 293–304. https://doi.org/10.1007/s10071-007-0077-4

Custance, D., Prato-Previde, E., Spiezio, C., Rigamonti, M. M., & Poli, M. (2006). Social learning in pig-tailed macaques (Macaca nemestrina) and adult humans (Homo sapiens) on a two-action artificial fruit. Journal of Comparative Psychology, 120(3), 303–313. https://doi.org/10.1037/0735-7036.120.3.303

Da Cunha, R. G. T., & Byrne, R. W. (2006). Roars of black howler monkeys (Alouatta caraya): Evidence for a function in inter-group spacing. Behaviour, 143(10), 1169–1199. https://doi.org/10.1163/156853906778691568

D’Agostino, J., & Cunningham, C. (2015). Preliminary investigation of flexibility in learning color-reward associations in gibbons (Hylobatidae). American Journal of Primatology, 77(8), 854–868. https://doi.org/10.1002/ajp.22410

Damerius, L. A., Graber, S. M., Willems, E. P., & van Schaik, C. P. (2017). Curiosity boosts orang-utan problem-solving ability. Animal Behaviour, 134, 57–70. https://doi.org/10.1016/j.anbehav.2017.10.005

Daoudi, S., Badihi, G., & Buchanan-Smith, H. (2017). Is mixed-species living cognitively enriching? Enclosure use and welfare in two captive groups of tufted capuchins (Sapajus apella) and squirrel monkeys (Saimiri sciureus). Animal Behavior and Cognition, 4(1), 91–104. https://doi.org/10.12966/abc.06.02.2017

Darusman, H. S., Call, J., Sajuthi, D., Schapiro, S. J., Gjedde, A., Kalliokoski, O., & Hau, J. (2014). Delayed response task performance as a function of age in cynomolgus monkeys (Macaca fascicularis). Primates, 55(2), 259–267. https://doi.org/10.1007/s10329-013-0397-8

David Smith, J., Boomer, J., Church, B. A., Zakrzewski, A. C., Beran, M. J., & Baum, M. L. (2018). I scan, therefore i decline: The time course of difficulty monitoring in humans (Homo sapiens) and macaques (macaca mulatta). Journal of Comparative Psychology. https://doi.org/10.1037/com0000100

David Smith, J., Zakrzewski, A. C., Johnston, J. J. R., Roeder, J. L., Boomer, J., Gregory Ashby, F., & Church, B. A. (2015). Generalization of category knowledge and dimensional categorization in humans (Homo sapiens) and nonhuman primates (Macaca mulatta). Journal of Experimental Psychology: Animal Learning and Cognition, 41(4), 322–335. https://doi.org/10.1037/xan0000071

Davila-Ross, M., Hutchinson, J., Russell, J. L., Schaeffer, J., Billard, A., Hopkins, W. D., & Bard, K. A. (2014). Triggering social interactions: Chimpanzees respond to imitation by a humanoid robot and request responses from it. Animal Cognition, 17(3), 589–595. https://doi.org/10.1007/s10071-013-0689-9

Davis, S. J., Schapiro, S. J., Lambeth, S. P., Wood, L. A., & Whiten, A. (2019). Behavioral conservatism is linked to complexity of behavior in chimpanzees (Pan troglodytes): Implications for cognition and cumulative culture. Journal of Comparative Psychology, 133(1), 20–35. https://doi.org/10.1037/com0000123

De Lillo, C., Spinozzi, G., Palumbo, M., & Giustino, G. (2011). Attention allocation modulates the processing of hierarchical visual patterns: A comparative analysis of capuchin monkeys (Cebus apella) and humans. Journal of Experimental Psychology: Animal Behavior Processes, 37(3), 341–352. https://doi.org/10.1037/a0022989

De Marco, A., Cozzolino, R., & Thierry, B. (2018). Prolonged transport and cannibalism of mummified infant remains by a Tonkean macaque mother. Primates, 59(1), 55–59. https://doi.org/10.1007/s10329-017-0633-8

De Moraes, B. L. C., Da Silva Souto, A., & Schiel, N. (2014). Adaptability in stone tool use by wild capuchin monkeys (Sapajus libidinosus). American Journal of Primatology, 76(10), 967–977. https://doi.org/10.1002/ajp.22286

De Petrillo, F., Caroli, M., Gori, E., Micucci, A., Gastaldi, S., Bourgeois-Gironde, S., & Addessi, E. (2019). Evolutionary origins of money categorization and exchange: an experimental investigation in tufted capuchin monkeys (Sapajus spp.). Animal Cognition, 22(2), 169–186. https://doi.org/10.1007/s10071-018-01233-2

De Petrillo, F., Gori, E., Micucci, A., Ponsi, G., Paglieri, F., & Addessi, E. (2015). When is it worth waiting for? Food quantity, but not food quality, affects delay tolerance in tufted capuchin monkeys. Animal Cognition, 18(5), 1019–1029. https://doi.org/10.1007/s10071-015-0869-x

De Petrillo, F., & Rosati, A. G. (2020). Logical inferences from visual and auditory information in ruffed lemurs and sifakas. Animal Behaviour, 164, 193–204. https://doi.org/10.1016/j.anbehav.2020.03.010

De Petrillo, F., Tonachella, G., & Addessi, E. (2017). Emotional correlates of probabilistic decision making in tufted capuchin monkeys (Sapajus spp.). Animal Behaviour, 129, 249–256. https://doi.org/10.1016/j.anbehav.2017.06.001

De Petrillo, F., Ventricelli, M., Ponsi, G., & Addessi, E. (2015). Do tufted capuchin monkeys play the odds? Flexible risk preferences in Sapajus spp. Animal Cognition, 18(1), 119–130. https://doi.org/10.1007/s10071-014-0783-7

de Raad, A. L., & Hill, R. A. (2019). Topological spatial representation in wild chacma baboons (Papio ursinus). Animal Cognition, 22, 397–412. https://doi.org/10.1007/s10071-019-01253-6

de Souza, F. S. C., & Calouro, A. M. (2018). Predation of army ants by Toppin’s titi monkey, Plecturocebus toppini Thomas 1914 (Primates: Pitheciidae), in an urban forest fragment in eastern Acre, Brazil. Primates, 59(5), 469–474. https://doi.org/10.1007/s10329-018-0677-4

Dean, L. G., Hoppitt, W., Laland, K. N., & Kendal, R. L. (2011). Sex ratio affects sex-specific innovation and learning in captive ruffed lemurs (Varecia variegata and Varecia rubra). American Journal of Primatology, 73(12), 1210–1221. https://doi.org/10.1002/ajp.20991

Deblauwe, I., Guislain, P., Dupain, J., & Van Elsacker, L. (2006). Use of a tool-set by Pan troglodytes troglodytes to obtain termites (Macrotermes) in the periphery of the Dja Biosphere Reserve, southeast Cameroon. American Journal of Primatology, 68(12), 1191–1196. https://doi.org/10.1002/ajp.20318

Defolie, C., Malassis, R., Serre, M., & Meunier, H. (2015). Tufted capuchins (Cebus apella) adapt their communicative behaviour to human’s attentional states. Animal Cognition, 18(3), 747–755. https://doi.org/10.1007/s10071-015-0841-9

Dekleva, M., van den Berg, L., Spruijt, B. M., & Sterck, E. H. M. (2012). Take it or leave it: Transport of tools for future use by long-tailed macaques (Macaca fascicularis). Behavioural Processes, 90(3), 392–401. https://doi.org/10.1016/j.beproc.2012.04.003

Dell’Mour, V., Range, F., & Huber, L. (2009). Social learning and mother’s behavior in manipulative tasks in infant marmosets. American Journal of Primatology, 71(6), 503–509. https://doi.org/10.1002/ajp.20682

DeLong, C. M., & Burnett, C. (2020). Bornean Orangutans (Pongo pygmaeus pygmaeus) Use Water as a Tool in the Floating Object Task. Animal Behavior and Cognition, 7(3), 327–342. https://doi.org/10.26451/abc.07.03.05.2020

Demuru, E., Ferrari, P. F., & Palagi, E. (2015). Emotionality and intentionality in bonobo playful communication. Animal Cognition, 18(1), 333–344. https://doi.org/10.1007/s10071-014-0804-6

Deppe, A. M., Wright, P. C., & Szelistowski, W. A. (2009). Object permanence in lemurs. Animal Cognition, 12(2), 381–388. https://doi.org/10.1007/s10071-008-0197-5

Di Fiore, A., & Suarez, S. A. (2007). Route-based travel and shared routes in sympatric spider and woolly monkeys: Cognitive and evolutionary implications. Animal Cognition, 10(3), 317–329. https://doi.org/10.1007/s10071-006-0067-y

Digweed, S. M., Fedigan, L. M., & Rendall, D. (2007). Who cares who calls? Selective responses to the lost calls of socially dominant group members in the white-faced capuchin (Cebus capucinus). American Journal of Primatology, 69(7), 829–835. https://doi.org/10.1002/ajp.20398

Dillis, C., Humle, T., & Snowdon, C. T. (2010). Socially biased learning among adult cottontop tamarins (Saguinus oedipus). American Journal of Primatology, 72(4), 287–295. https://doi.org/10.1002/ajp.20778

Dindo, M., & De Waal, F. B. M. (2007). Partner effects on food consumption in brown capuchin monkeys. American Journal of Primatology, 69(4), 448–456. https://doi.org/10.1002/ajp.20362

Dindo, M., Leimgruber, K. L., Ahmed, R., Whiten, A., & de Waal, F. B. M. (2011). Observer choices during experimental foraging tasks in brown capuchin monkeys (Cebus apella). American Journal of Primatology, 73(9), 920–927. https://doi.org/10.1002/ajp.20962

Dindo, M., Thierry, B., de Waal, F. B. M., & Whiten, A. (2010). Conditional Copying Fidelity in Capuchin Monkeys (Cebus apella). Journal of Comparative Psychology, 124(1), 29–37. https://doi.org/10.1037/a0018005

Dindo, M., Whiten, A., & De Waal, F. (2009). Social facilitation of exploratory foraging behavior in capuchin monkeys (Cebus apella). American Journal of Primatology, 71(5), 419–426. https://doi.org/10.1002/ajp.20669

Do Nascimento Castro, S. C., Da Silva Souto, A., Schiel, N., Biondi, L. M., & Caselli, C. B. (2017). Techniques Used by Bearded Capuchin Monkeys (Sapajus libidinosus) to Access Water in a Semi-Arid Environment of North-Eastern Brazil. Folia Primatologica, 88(3), 267–273. https://doi.org/10.1159/000479106

Dolins, F. L. (2009). Captive cotton-top tamarins’ (Saguinus oedipus oedipus) use of landmarks to localize hidden food items. American Journal of Primatology, 71(4), 316–323. https://doi.org/10.1002/ajp.20654

Dolins, F. L., Klimowicz, C., Kelley, J., & Menzel, C. R. (2014). Using virtual reality to investigate comparative spatial cognitive abilities in chimpanzees and humans. American Journal of Primatology, 76(5), 496–513. https://doi.org/10.1002/ajp.22252

Drayton, L. A., Brosnan, S. F., Carrigan, J., & Stoinski, T. S. (2013). Endowment effects in gorillas (gorilla gorilla). Journal of Comparative Psychology, 127(4), 365–369. https://doi.org/10.1037/a0031902

Drayton, L. A., & Santos, L. R. (2017). Do rhesus macaques, Macaca mulatta, understand what others know when gaze following? Animal Behaviour, 134, 193–199. https://doi.org/10.1016/j.anbehav.2017.10.016

Drayton, L. A., & Santos, L. R. (2014). Capuchins’ (Cebus apella) sensitivity to others’ goal-directed actions in a helping context. Animal Cognition, 17(3), 689–700. https://doi.org/10.1007/s10071-013-0700-5

Drayton, L. A., Varman, L., & Santos, L. R. (2016). Capuchins (Cebus apella) are limited in their ability to infer others’ goals based on context. Journal of Comparative Psychology, 130(1), 71–75. https://doi.org/10.1037/com0000016

Drucker, C. B., Rossa, M. A., & Brannon, E. M. (2016). Comparison of discrete ratios by rhesus macaques (Macaca mulatta). Animal Cognition, 19(1), 75–89. https://doi.org/10.1007/s10071-015-0914-9

Duarte, R. B. M., Patrono, E., Borges, A. C., César, A. A. S., Tomaz, C., Ventura, R., Gasbarri, A., Puglisi-Allegra, S., & Barros, M. (2014). Consumption of a highly palatable food induces a lasting place-conditioning memory in marmoset monkeys. Behavioural Processes, 107, 163–166. https://doi.org/10.1016/j.beproc.2014.08.021

Dufour, V., Pelé, M., Sterck, E. H. M., & Thierry, B. (2007). Chimpanzee (Pan troglodytes) anticipation of food return: Coping with waiting time in an exchange task. Journal of Comparative Psychology, 121(2), 145–155. https://doi.org/10.1037/0735-7036.121.2.145

Dufour, V., & Sterck, E. H. M. (2008). Chimpanzees fail to plan in an exchange task but succeed in a tool-using procedure. Behavioural Processes, 79(1), 19–27. https://doi.org/10.1016/j.beproc.2008.04.003

Dufour, V., Pascalis, O., & Petit, O. (2006). Face processing limitation to own species in primates: A comparative study in brown capuchins, Tonkean macaques and humans. Behavioural Processes, 73(1), 107–113. https://doi.org/10.1016/j.beproc.2006.04.006

Duguid, S., Wyman, E., Grueneisen, S., & Tomasello, M. (2020). The Strategies Used by Chimpanzees (Pan troglodytes) and Children (Homo sapiens) to Solve a Simple Coordination Problem. Journal of Comparative Psychology, 134(4), 401. https://doi.org/10.1037/com0000220

Dutton, P., & Chapman, H. (2015). New tools suggest local variation in tool use by a montane community of the rare Nigeria–Cameroon chimpanzee, Pan troglodytes ellioti, in Nigeria. Primates, 56(1), 89–100. https://doi.org/10.1007/s10329-014-0451-1

Ebel, S. J., Schmelz, M., Herrmann, E., & Call, J. (2019). Innovative problem solving in great apes: the role of visual feedback in the floating peanut task. Animal Cognition, 22(5), 791–805. https://doi.org/10.1007/s10071-019-01275-0

Ebel, S. J., & Call, J. (2018). The interplay of prior experience and motivation in great ape problem-solving (Gorilla gorilla, Pan paniscus, Pan troglodytes, and Pongo abelii). Journal of Comparative Psychology, 132(3), 294–305. https://doi.org/10.1037/com0000117

Eberle, M., & Rappeler, P. M. (2008). Mutualism, reciprocity, or kin selection? cooperative rescue of a conspecific from a boa in a nocturnal solitary forager the gray mouse lemur. American Journal of Primatology, 70(4), 410–414. https://doi.org/10.1002/ajp.20496

Eckert, J., Rakoczy, H., & Call, J. (2017). Are great apes able to reason from multi-item samples to populations of food items? American Journal of Primatology, 79(10), 1–14. https://doi.org/10.1002/ajp.22693

Elkins, D. N. (2012). Toward a common focus in psychotherapy research. Psychotherapy, 49(4), 450–454. https://doi.org/10.1037/a0027797

Elmore, L. C., Magnotti, J. F., Katz, J. S., & Wright, A. A. (2012). Change detection by rhesus monkeys (Macaca mulatta) and pigeons (Columba livia). Journal of Comparative Psychology, 126(3), 203–212. https://doi.org/10.1037/a0026356

Elmore, L. C., & Wright, A. A. (2015). Monkey visual short-term memory directly compared to humans. Journal of Experimental Psychology: Animal Learning and Cognition, 41(1), 32–38. https://doi.org/10.1037/xan0000050

Emidio, R. A., & Ferreira, R. G. (2012). Energetic Payoff of Tool Use for Capuchin Monkeys in the Caatinga: Variation by Season and Habitat Type. American Journal of Primatology, 74(4), 332–343. https://doi.org/10.1002/ajp.22009

Emigh, H., Truax, J., Highfill, L., & Vonk, J. (2020). Not by the same token: A female orangutan (Pongo pygmaeus) is selectively prosocial. Primates, 61(2), 237–247. https://doi.org/10.1007/s10329-019-00780-7

Endress, A. D., Carden, S., Versace, E., & Hauser, M. D. (2010). The apes’ edge: Positional learning in chimpanzees and humans. Animal Cognition, 13(3), 483–495. https://doi.org/10.1007/s10071-009-0299-8

Engelmann, J. M., Herrmann, E., & Tomasello, M. (2016). The effects of being watched on resource acquisition in chimpanzees and human children. Animal Cognition, 19(1), 147–151. https://doi.org/10.1007/s10071-015-0920-y

Engh, A. L., Hoffmeier, R. R., Cheney, D. L., & Seyfarth, R. M. (2006). Who, me? Can baboons infer the target of vocalizations? Animal Behaviour, 71(2), 381–387. https://doi.org/10.1016/j.anbehav.2005.05.009

Englerova, K., Klement, D., Frynta, D., Rokyta, R., & Nekovarova, T. (2019). Reactions to novel objects in monkeys: what does it mean to be neophobic? Primates, 60(4), 347–353. https://doi.org/10.1007/s10329-019-00731-2

Eppley, T. M., Suchak, M., Crick, J., & de Waal, F. B. M. (2013). Perseverance and food sharing among closely affiliated female chimpanzees. Primates, 54(4), 319–324. https://doi.org/10.1007/s10329-013-0374-2

Erhart, E. M., & Overdorff, D. J. (2008). Spatial memory during foraging in prosimian primates: Propithecus edwardsi and Eulemur fulvus rufus. Folia Primatologica, 79(4), 185–196. https://doi.org/10.1159/000112911

Eshchar, Y., Izar, P., Visalberghi, E., Resende, B., & Fragaszy, D. (2016). When and where to practice: social influences on the development of nut-cracking in bearded capuchins (Sapajus libidinosus). Animal Cognition, 19(3), 605–618. https://doi.org/10.1007/s10071-016-0965-6

Essler, J. L., Schwartz, L. P., Rossettie, M. S., & Judge, P. G. (2017). Capuchin monkeys’ use of human and conspecific cues to solve a hidden object-choice task. Animal Cognition, 20(5), 985–998. https://doi.org/10.1007/s10071-017-1118-2

Estienne, V., Robira, B., Mundry, R., Deschner, T., & Boesch, C. (2019). Acquisition of a complex extractive technique by the immature chimpanzees of Loango National Park, Gabon. Animal Behaviour, 147, 61–76. https://doi.org/10.1016/j.anbehav.2018.11.002

Evans, T. A., Beran, M. J., Paglieri, F., & Addessi, E. (2012). Delaying gratification for food and tokens in capuchin monkeys (Cebus apella) and chimpanzees (Pan troglodytes): When quantity is salient, symbolic stimuli do not improve performance. Animal Cognition, 15(4), 539–548. https://doi.org/10.1007/s10071-012-0482-1

Evans, T. A., Beran, M. J., & Addessi, E. (2010). Can nonhuman primates use tokens to represent and sum quantities? Journal of Comparative Psychology, 124(4), 369–380. https://doi.org/10.1037/a0019855

Evans, T. A., Beran, M. J., Harris, E. H., & Rice, D. F. (2009). Quantity judgments of sequentially presented food items by capuchin monkeys (Cebus apella). Animal Cognition, 12(1), 97–105. https://doi.org/10.1007/s10071-008-0174-z

Evans, T. A., Perdue, B. M., Parrish, A. E., & Beran, M. J. (2014). Working and waiting for better rewards: Self-control in two monkey species (Cebus apella and Macaca mulatta). Behavioural Processes, 103, 236–242. https://doi.org/10.1016/j.beproc.2014.01.001

Evans, T. A., Smith, J. D., Sayers, K., Menzel, E., & Beran, M. J. (2015). The misbehaviour of a metacognitive monkey. Behaviour, 152(6), 727–756. https://doi.org/10.1163/1568539X-00003251

Evans, T. A., & Westergaard, G. C. (2006). Self-control and tool use in tufted capuchin monkeys (Cebus Apella). Journal of Comparative Psychology, 120(2), 163–166. https://doi.org/10.1037/0735-7036.120.2.163

Evans, T., & Beran, M. (2014). Monkeys Wait to Begin a Computer Task when Waiting Makes Their Responses More Effective. Animal Behavior and Cognition, 1(1), 36–50. https://doi.org/10.12966/abc.02.03.2014

Fagot, J., Barbet, I., Parron, C., & Deruelle, C. (2006). Amodal completion by baboons (Papio papio): Contribution of background depth cues. Primates, 47(2), 145–150. https://doi.org/10.1007/s10329-005-0165-5

Fagot, J., Bonté, E., & Hopkins, W. D. (2013). Age-dependant behavioral strategies in a visual search task in baboons (papio papio) and their relation to inhibitory control. Journal of Comparative Psychology, 127(2), 194–201. https://doi.org/10.1037/a0026385

Fagot, J., Bonté, E., & Parron, C. (2009). Concept of uprightness in baboons: Assessment with pictures of realistic scenes. Animal Cognition, 12(2), 369–379. https://doi.org/10.1007/s10071-008-0196-6

Fagot, J., & Parron, C. (2010). Relational Matching in Baboons (Papio papio) With Reduced Grouping Requirements. Journal of Experimental Psychology: Animal Behavior Processes, 36(2), 184–193. https://doi.org/10.1037/a0017169

Fallon, B. L., Neumann, C., Byrne, R. W., & Zuberbühler, K. (2016). Female chimpanzees adjust copulation calls according to reproductive status and level of female competition. Animal Behaviour, 113, 87–92. https://doi.org/10.1016/j.anbehav.2015.12.028

Falótico, T., Coutinho, P. H. M., Bueno, C. Q., Rufo, H. P., & Ottoni, E. B. (2018). Stone tool use by wild capuchin monkeys (Sapajus libidinosus) at Serra das Confusões National Park, Brazil. Primates, 59(4), 385–394. https://doi.org/10.1007/s10329-018-0660-0

Falótico, T., Luncz, L. V., Svensson, M. S., & Haslam, M. (2017). Cashew Nut Positioning during Stone Tool Use by Wild Bearded Capuchin Monkeys (Sapajus libidinosus). Folia Primatologica, 87(6), 392–397. https://doi.org/10.1159/000459621

Falótico, T., & Ottoni, E. B. (2016). The manifold use of pounding stone tools by wild capuchin monkeys of Serra da Capivara National Park, Brazil. Behaviour, 153(4), 421–442. https://doi.org/10.1163/1568539X-00003357

Falótico, T., & Ottoni, E. B. (2014). Sexual bias in probe tool manufacture and use by wild bearded capuchin monkeys. Behavioural Processes, 108, 117–122. https://doi.org/10.1016/j.beproc.2014.09.036

Faraut, L., & Fischer, J. (2019). How life in a tolerant society affects the attention to social information in baboons. Animal Behaviour, 152, 11–17. https://doi.org/10.1016/j.anbehav.2019.04.004

Faraut, L., Siviter, H., Pesco, F. D., & Fischer, J. (2019). How life in a tolerant society affects the usage of grunts: evidence from female and male Guinea baboons. Animal Behaviour, 153, 83–93. https://doi.org/10.1016/j.anbehav.2019.05.003

Fedor, A., Skollár, G., Szerencsy, N., & Ujhelyi, M. (2008). Object Permanence Tests on Gibbons (Hylobatidae). Journal of Comparative Psychology, 122(4), 403–417. https://doi.org/10.1037/0735-7036.122.4.403

Fedurek, P., & Slocombe, K. E. (2013). The social function of food-associated calls in male chimpanzees. American Journal of Primatology, 75(7), 726–739. https://doi.org/10.1002/ajp.22122

Fedurek, P., Slocombe, K. E., & Zuberbühler, K. (2015). Chimpanzees communicate to two different audiences during aggressive interactions. Animal Behaviour, 110, 21–28. https://doi.org/10.1016/j.anbehav.2015.09.010

Ferreira, R. G., Emidio, R. A., & Jerusalinsky, L. (2010). Three stones for three seeds: Natural occurrence of selective tool use by capuchins (Cebus libidinosus) based on an analysis of the weight of stones found at nutting sites. American Journal of Primatology, 72(3), 270–275. https://doi.org/10.1002/ajp.20771

Fichtel, C., & Kappeler, P. M. (2011). Variation in the Meaning of Alarm Calls in Verreaux’s and Coquerel’s Sifakas (Propithecus verreauxi, P. coquereli). International Journal of Primatology, 32(2), 346–361. https://doi.org/10.1007/s10764-010-9472-9

Finestone, E., Bonnie, K. E., Hopper, L. M., Vreeman, V. M., Lonsdorf, E. V., & Ross, S. R. (2014). The interplay between individual, social, and environmental influences on chimpanzee food choices. Behavioural Processes, 105, 71–78. https://doi.org/10.1016/j.beproc.2014.03.006

Fiore, A. M., Cronin, K. A., Ross, S. R., & Hopper, L. M. (2020). Food Cleaning by Japanese Macaques: Innate, Innovative or Cultural? Folia Primatologica, 91(4), 433–444. https://doi.org/10.1159/000506127

Flemming, T. M. (2011). Conceptual thresholds for same and different in old-(Macaca mulatta) and new-world (Cebus apella) monkeys. Behavioural Processes, 86(3), 316–322. https://doi.org/10.1016/j.beproc.2011.01.001

Flemming, T. M., Beran, M. J., Thompson, R. K. R., Kleider, H. M., & Washburn, D. A. (2008). What Meaning Means for Same and Different: Analogical Reasoning in Humans (Homo sapiens), Chimpanzees (Pan troglodytes), and Rhesus Monkeys (Macaca mulatta). Journal of Comparative Psychology, 122(2), 176–185. https://doi.org/10.1037/0735-7036.122.2.176

Flemming, T. M., & Kennedy, E. H. (2011). Chimpanzee (Pan troglodytes) Relational Matching: Playing by Their Own (Analogical) Rules. Journal of Comparative Psychology, 125(2), 207–215. https://doi.org/10.1037/a0022098

Flemming, T. M., Thompson, R. K. R., Beran, M. J., & Washburn, D. A. (2011). Analogical reasoning and the differential outcome effect: Transitory bridging of the conceptual gap for rhesus monkeys (Macaca mulatta). Journal of Experimental Psychology: Animal Behavior Processes, 37(3), 353–360. https://doi.org/10.1037/a0022142

Flemming, T. M., Thompson, R. K. R., & Fagot, J. (2013). Baboons, like humans, solve analogy by categorical abstraction of relations. Animal Cognition, 16(3), 519–524. https://doi.org/10.1007/s10071-013-0596-0

Fletcher, G. E. (2008). Attending to the outcome of others: Disadvantageous inequity aversion in male capuchin monkeys (Cebus apella). American Journal of Primatology, 70(9), 901–905. https://doi.org/10.1002/ajp.20576

Fontenot, M. B., Watson, S. L., Roberts, K. A., & Miller, R. W. (2007). Effects of food preferences on token exchange and behavioural responses to inequality in tufted capuchin monkeys, Cebus apella. Animal Behaviour, 74(3), 487–496. https://doi.org/10.1016/j.anbehav.2007.01.015

Forbanka, D. N. (2020). Playback of Fork-Marked Lemurs’ Loud Calls in Kirindy Forest, Madagascar. Folia Primatologica, 91(2), 83–95. https://doi.org/10.1159/000501506

Fowler, A., & Sommer, V. (2007). Subsistence technology of Nigerian chimpanzees. International Journal of Primatology, 28(5), 997–1023. https://doi.org/10.1007/s10764-007-9166-0

Fragaszy, D., Pickering, T., Liu, Q., Izar, P., Ottoni, E., & Visalberghi, E. (2010). Bearded capuchin monkeys’ and a human’s efficiency at cracking palm nuts with stone tools: field experiments. Animal Behaviour, 79(2), 321–332. https://doi.org/10.1016/j.anbehav.2009.11.004

Fragaszy, D. M., Barton, S. A., Keo, S., Patel, R., Izar, P., Visalberghi, E., & Haslam, M. (2020). Adult and juvenile bearded capuchin monkeys handle stone hammers differently during nut-cracking. American Journal of Primatology, 82(7). https://doi.org/10.1002/ajp.23156

Fragaszy, D. M., Deputte, B., Cooper, E. J., Colbert-White, E. N., & Hémery, C. (2011). When and how well can human-socialized capuchins match actions demonstrated by a familiar human? American Journal of Primatology, 73(7), 643–654. https://doi.org/10.1002/ajp.20941

Fragaszy, D. M., Greenberg, R., Visalberghi, E., Ottoni, E. B., Izar, P., & Liu, Q. (2010). How wild bearded capuchin monkeys select stones and nuts to minimize the number of strikes per nut cracked. Animal Behaviour, 80(2), 205–214. https://doi.org/10.1016/j.anbehav.2010.04.018

Fragaszy, D. M., Kennedy, E., Murnane, A., Menzel, C., Brewer, G., Johnson-Pynn, J., & Hopkins, W. (2009). Navigating two-dimensional mazes: Chimpanzees (Pan troglodytes) and capuchins (Cebus apella sp.) profit from experience differently. Animal Cognition, 12(3), 491–504. https://doi.org/10.1007/s10071-008-0210-z

Fragaszy, D. M., Stone, B. W., Scott, N. M., & Menzel, C. (2011). How tufted capuchin monkeys (Cebus apella spp) and common chimpanzees (Pan troglodytes) align objects to surfaces: Insights into spatial reasoning and implications for tool use. American Journal of Primatology, 73(10), 1012–1030. https://doi.org/10.1002/ajp.20966

Frank, R. E., & Silk, J. B. (2009). Impatient traders or contingent reciprocators? Evidence for the extended time-course of grooming exchanges in baboons. Behaviour, 146(8), 1123–1135. https://doi.org/10.1163/156853909X406455

Frank, R. E., & Silk, J. B. (2009). Grooming exchange between mothers and non-mothers: The price of natal attraction in wild baboons (Papio anubis). Behaviour, 146(7), 889–906. https://doi.org/10.1163/156853908X396656

Fratellone, G. P., Li, J. H., Sheeran, L. K., Wagner, R. S., Wang, X., & Sun, L. (2019). Social connectivity among female Tibetan macaques (Macaca thibetana) increases the speed of collective movements. Primates, 60(3), 183–189. https://doi.org/10.1007/s10329-018-0691-6

Fredman, T., & Whiten, A. (2008). Observational learning from tool using models by human-reared and mother-reared capuchin monkeys (Cebus apella). Animal Cognition, 11(2), 295–309. https://doi.org/10.1007/s10071-007-0117-0

Freeman, K. B., Green, L., Myerson, J., & Woolverton, W. L. (2009). Delay discounting of saccharin in rhesus monkeys. Behavioural Processes, 82(2), 214–218. https://doi.org/10.1016/j.beproc.2009.06.002

Fröhlich, M., Wittig, R. M., & Pika, S. (2016). Should I stay or should I go? Initiation of joint travel in mother–infant dyads of two chimpanzee communities in the wild. Animal Cognition, 19(3), 483–500. https://doi.org/10.1007/s10071-015-0948-z

Fruteau, C., Lemoine, S., Hellard, E., van Damme, E., & Noë, R. (2011). When females trade grooming for grooming: Testing partner control and partner choice models of cooperation in two primate species. Animal Behaviour, 81(6), 1223–1230. https://doi.org/10.1016/j.anbehav.2011.03.008

Fruteau, C., van de Waal, E., van Damme, E., & Noë, R. (2011). Infant access and handling in sooty mangabeys and vervet monkeys. Animal Behaviour, 81(1), 153–161. https://doi.org/10.1016/j.anbehav.2010.09.028

Fujita, K. (2009). Metamemory in tufted capuchin monkeys (Cebus apella). Animal Cognition, 12(4), 575–585. https://doi.org/10.1007/s10071-009-0217-0

Fujita, K., Sato, Y., & Kuroshima, H. (2011). Learning and Generalization of Tool Use by Tufted Capuchin Monkeys (Cebus apella) in Tasks Involving Three Factors: Reward, Tool, and Hindrance. Journal of Experimental Psychology: Animal Behavior Processes, 37(1), 10–19. https://doi.org/10.1037/a0020274

Furlong, E. E., Boose, K. J., & Boysen, S. T. (2008). Raking it in: The impact of enculturation on chimpanzee tool use. Animal Cognition, 11(1), 83–97. https://doi.org/10.1007/s10071-007-0091-6

Furuichi, T., Sanz, C., Koops, K., Sakamaki, T., Ryu, H., Tokuyama, N., & Morgan, D. (2015). Why do wild bonobos not use tools like chimpanzees do? Behaviour, 152(3–4), 425–460. https://doi.org/10.1163/1568539X-00003226

Gagne, M., Levesque, K., Nutile, L., & Locurto, C. (2012). Performance on patterned string problems by common marmosets (Callithrix jacchus). Animal Cognition, 15(5), 1021–1030. https://doi.org/10.1007/s10071-012-0511-0

Gao, J., Su, Y., Tomonaga, M., & Matsuzawa, T. (2018). Learning the rules of the rock–paper–scissors game: chimpanzees versus children. Primates, 59(1), 7–17. https://doi.org/10.1007/s10329-017-0620-0

Gao, J., & Tomonaga, M. (2020). Body perception in chimpanzees (Pan troglodytes): The effect of body structure changes. Journal of Comparative Psychology, 134(2), 222–231. https://doi.org/10.1037/com0000214

Garber, P. A., Gomes, D. F., & Bicca-Marques, J. C. (2012). Experimental field study of problem-solving using tools in free-ranging capuchins (Sapajus nigritus, formerly Cebus nigritus). American Journal of Primatology, 74(4), 344–358. https://doi.org/10.1002/ajp.20957

Garber, P. A., & Porter, L. M. (2014). Navigating in small-scale space: The role of landmarks and resource monitoring in understanding saddleback tamarin travel. American Journal of Primatology, 76(5), 447–459. https://doi.org/10.1002/ajp.22196

Gazes, R. P. axto., Lazareva, O. F., Bergene, C. N., & Hampton, R. R. (2014). Effects of spatial training on transitive inference performance in humans and rhesus monkeys. Journal of Experimental Psychology. Animal Learning and Cognition, 40(4), 477–489. https://doi.org/10.1037/xan0000038

Gazes, R. P., Brown, E. K., Basile, B. M., & Hampton, R. R. (2013). Automated cognitive testing of monkeys in social groups yields results comparable to individual laboratory-based testing. Animal Cognition, 16(3), 445–458. https://doi.org/10.1007/s10071-012-0585-8

Gazes, R. P., Chee, N. W., & Hampton, R. R. (2012). Cognitive mechanisms for transitive inference performance in rhesus monkeys: Measuring the influence of associative strength and inferred order. Journal of Experimental Psychology: Animal Behavior Processes, 38(4), 331–345. https://doi.org/10.1037/a0030306

Geissmann, T., & Mutschler, T. (2006). Diurnal distribution of loud calls in sympatric wild indris (indri indri) and ruffed lemurs (varecia variegata): Implications for call functions. Primates, 47(4), 393–396. https://doi.org/10.1007/s10329-006-0189-5

Genty, E. (2019). Vocal–gestural combinations in infant bonobos: new insights into signal functional specificity. Animal Cognition, 22(4), 505–518. https://doi.org/10.1007/s10071-019-01267-0

Genty, E., Breuer, T., Hobaiter, C., & Byrne, R. W. (2009). Gestural communication of the gorilla (Gorilla gorilla): Repertoire, intentionality and possible origins. Animal Cognition, 12(3), 527–546. https://doi.org/10.1007/s10071-009-0213-4

Genty, E., & Byrne, R. W. (2010). Why do gorillas make sequences of gestures? Animal Cognition, 13(2), 287–301. https://doi.org/10.1007/s10071-009-0266-4

Genty, E., Chung, P. C. S., & Roeder, J. J. (2011). Testing brown lemurs (Eulemur fulvus) on the reverse-reward contingency task without a modified procedure. Behavioural Processes, 86(1), 133–137. https://doi.org/10.1016/j.beproc.2010.10.006

Genty, E., Foltz, J., & Roeder, J. J. (2008). Can brown lemurs (Eulemur fulvus) learn to deceive a human competitor? Animal Cognition, 11(2), 255–266. https://doi.org/10.1007/s10071-007-0107-2

Genty, E., Karpel, H., & Silberberg, A. (2012). Time preferences in long-tailed macaques (Macaca fascicularis) and humans (Homo sapiens). Animal Cognition, 15(6), 1161–1172. https://doi.org/10.1007/s10071-012-0540-8

Genty, E., & Roeder, J. J. (2011). Can lemurs (Eulemur fulvus and E. macaco) use abstract representations of quantities to master the reverse-reward contingency task? Primates, 52(3), 253–260. https://doi.org/10.1007/s10329-011-0245-7

Genty, E., & Roeder, J. J. (2007). Transfer of Self-Control in Black (Eulemur macaco) and Brown (Eulemur fulvus) Lemurs: Choice of a Less Preferred Food Item Under a Reverse-Reward Contingency. Journal of Comparative Psychology, 121(4), 354–362. https://doi.org/10.1037/0735-7036.121.4.354

Genty, E., & Roeder, J. J. (2006). Can lemurs learn to deceive? A study in the black lemur (Eulemur macaco). Journal of Experimental Psychology: Animal Behavior Processes, 32(2), 196–200. https://doi.org/10.1037/0097-7403.32.2.196

Ghasemian, S., Vardanjani, M. M., Sheibani, V., & Mansouri, F. A. (2021). Color-hierarchies in executive control of monkeys’ behavior. American Journal of Primatology, 83(2), 23231. https://doi.org/10.1002/ajp.23231

Gibson, K. N. (2010). Male mating tactics in spider monkeys: Sneaking to compete. American Journal of Primatology, 72(9), 794–804. https://doi.org/10.1002/ajp.20835

Girard, P., Jouffrais, C., & Kirchner, C. H. (2008). Ultra-rapid categorisation in non-human primates. Animal Cognition, 11(3), 485–493. https://doi.org/10.1007/s10071-008-0139-2

Girard-Buttoz, C., Surbeck, M., Samuni, L., Boesch, C., Fruth, B., Crockford, C., Hohmann, G., & Wittig, R. M. (2020). Variable use of polyadic grooming and its effect on access to social partners in wild chimpanzees and bonobos. Animal Behaviour, 168, 211–224. https://doi.org/10.1016/j.anbehav.2020.08.021

Girndt, A., Meier, T., & Call, J. (2008). Task Constraints Mask Great Apes’ Ability to Solve the Trap-Table Task. Journal of Experimental Psychology: Animal Behavior Processes, 34(1), 54–62. https://doi.org/10.1037/0097-7403.34.1.54

Goldstone, L. G., Sommer, V., Nurmi, N., Stephens, C., & Fruth, B. (2016). Food begging and sharing in wild bonobos (Pan paniscus): assessing relationship quality? Primates, 57(3), 367–376. https://doi.org/10.1007/s10329-016-0522-6

Gomes, D. F., & Bicca-Marques, J. C. (2012). Capuchin monkeys (Cebus nigritus) use spatial and visual information during within-patch foraging. American Journal of Primatology, 74(1), 58–67. https://doi.org/10.1002/ajp.21009

Goossens, B. M. A., Dekleva, M., Reader, S. M., Sterck, E. H. M., & Bolhuis, J. J. (2008). Gaze following in monkeys is modulated by observed facial expressions. Animal Behaviour, 75(5), 1673–1681. https://doi.org/10.1016/j.anbehav.2007.10.020

Goossens, B. M. A., Van Den Berg, L. M., Reader, S. M., & Stercka, E. H. M. (2012). An analysis of gaze following to a hidden location in long-tailed macaques (Macaca fascicularis). Behaviour, 149(13–14), 1319–1337. https://doi.org/10.1163/1568539X-00003017

Gothard, K. M., Brooks, K. N., & Peterson, M. A. (2009). Multiple perceptual strategies used by macaque monkeys for face recognition. Animal Cognition, 12(1), 155–167. https://doi.org/10.1007/s10071-008-0179-7

Goto, K., Imura, T., & Tomonaga, M. (2012). Perception of emergent configurations in humans (Homo sapiens) and chimpanzees (Pan troglodytes). Journal of Experimental Psychology: Animal Behavior Processes, 38(2), 125–138. https://doi.org/10.1037/a0026899

Graham, K. E., Furuichi, T., & Byrne, R. W. (2017). The gestural repertoire of the wild bonobo (Pan paniscus): a mutually understood communication system. Animal Cognition, 20(2), 171–177. https://doi.org/10.1007/s10071-016-1035-9

Grampp, M., Sueur, C., van de Waal, E., & Botting, J. (2019). Social attention biases in juvenile wild vervet monkeys: implications for socialisation and social learning processes. Primates, 60, 261–275. https://doi.org/10.1007/s10329-019-00721-4

Green, S. J., Boruff, B. J., & Grueter, C. C. (2020). From ridge tops to ravines: landscape drivers of chimpanzee ranging patterns. Animal Behaviour, 163, 51–60. https://doi.org/10.1016/j.anbehav.2020.02.016

Greenberg, J. R., Hamann, K., Warneken, F., & Tomasello, M. (2010). Chimpanzee helping in collaborative and noncollaborative contexts. Animal Behaviour, 80(5), 873–880. https://doi.org/10.1016/j.anbehav.2010.08.008

Gregory, T., Mullett, A., & Norconk, M. A. (2014). Strategies for navigating large areas: A GIS spatial ecology analysis of the bearded saki monkey, chiropotes sagulatus, in suriname. American Journal of Primatology, 76(6), 586–595. https://doi.org/10.1002/ajp.22251

Gretscher, H., Haun, D. B. M., Liebal, K., & Kaminski, J. (2012). Orang-utans rely on orientation cues and egocentric rules when judging others’ perspectives in a competitive food task. Animal Behaviour, 84(2), 323–331. https://doi.org/10.1016/j.anbehav.2012.04.021

Gruber, T. (2016). Great Apes Do Not Learn Novel Tool Use Easily: Conservatism, Functional Fixedness, or Cultural Influence? International Journal of Primatology, 37(2), 296–316. https://doi.org/10.1007/s10764-016-9902-4

Gruber, T. (2014). Wild-Born Orangutans (Pongo abelii) Engage in Triadic Interactions During Play. International Journal of Primatology, 35(2), 411–424. https://doi.org/10.1007/s10764-013-9745-1

Gruber, T., Clay, Z., & Zuberbühler, K. (2010). A comparison of bonobo and chimpanzee tool use: Evidence for a female bias in the Pan lineage. Animal Behaviour, 80(6), 1023–1033. https://doi.org/10.1016/j.anbehav.2010.09.005

Gruber, T., Potts, K. B., Krupenye, C., Byrne, M. R., Mackworth-Young, C., McGrew, W. C., Reynolds, V., & Zuberbühler, K. (2012). The influence of ecology on chimpanzee (Pan troglodytes) cultural behavior: A case study of five ugandan chimpanzee communities. Journal of Comparative Psychology, 126(4), 446–457. https://doi.org/10.1037/a0028702

Grueter, C. C., Robbins, M. M., Ndagijimana, F., & Stoinski, T. S. (2013). Possible tool use in a mountain gorilla. Behavioural Processes, 100, 160–162. https://doi.org/10.1016/j.beproc.2013.09.006

Guerreiro Martins, E. M., Antonio, A. C., Finkenwirth, C., Griesser, M., & Burkart, J. M. (2019). Food Sharing Patterns in Three Species of Callitrichid Monkeys (Callithrix jacchus, Leontopithecus chrysomelas, Saguinus midas): Individual and Species Differences. Journal of Comparative Psychology, 133(4), 474. https://doi.org/10.1037/com0000169

Gumert, M. D., & Ho, M. H. R. (2008). The trade balance of grooming and its coordination of reciprocation and tolerance in Indonesian long-tailed macaques (Macaca fascicularis). Primates, 49(3), 176–185. https://doi.org/10.1007/s10329-008-0089-y

Gumert, M. D., Hoong, L. K., & Malaivijitnond, S. (2011). Sex differences in the stone tool-use behavior of a wild population of burmese long-tailed macaques (Macaca fascicularis aurea). American Journal of Primatology, 73(12), 1239–1249. https://doi.org/10.1002/ajp.20996

Gumert, M. D., Kluck, M., & Malaivijitnond, S. (2009). The physical characteristics and usage patterns of stone axe and pounding hammers used by long-tailed macaques in the Andaman sea region of Thailand. American Journal of Primatology, 71(7), 594–608. https://doi.org/10.1002/ajp.20694

Gunhold, T., Massen, J. J. M., Schiel, N., Souto, A., & Bugnyar, T. (2014). Memory, transmission and persistence of alternative foraging techniques in wild common marmosets. Animal Behaviour, 91, 79–91. https://doi.org/10.1016/j.anbehav.2014.02.023

Gunst, N., Boinski, S., & Fragaszy, D. M. (2007). Acquisition of foraging competence in wild brown capuchins (Cebus apella), with special reference to conspecifics’ foraging artefacts as an indirect social influence. Behaviour, 145(2), 195–229. https://doi.org/10.1163/156853907783244701

Gunst, N., Leca, J. B., Boinski, S., & Fragaszy, D. (2010). The ontogeny of handling hard-to-process food in wild brown capuchins (Cebus apella apella): Evidence from foraging on the fruit of maximiliana maripa. American Journal of Primatology, 72(11), 960–973. https://doi.org/10.1002/ajp.20856

Gupta, S., & Sinha, A. (2019). Gestural communication of wild bonnet macaques in the Bandipur National Park, Southern India. Behavioural Processes, 168, 103956. https://doi.org/10.1016/j.beproc.2019.103956

Gupta, S., & Sinha, A. (2016). Not here, there! Possible referential gesturing during allogrooming by wild bonnet macaques, Macaca radiata. Animal Cognition, 19(6), 1243–1248. https://doi.org/10.1007/s10071-016-1012-3

Gustafsson, E., Krief, S., & Saint Jalme, M. (2011). Neophobia and learning mechanisms: How captive orangutans discover medicinal plants. Folia Primatologica, 82(1), 45–55. https://doi.org/10.1159/000326796

Gustafsson, E., Saint Jalme, M., Bomsel, M. C., & Krief, S. (2014). Food Neophobia and Social Learning Opportunities in Great Apes. International Journal of Primatology, 35(5), 1037–1071. https://doi.org/10.1007/s10764-014-9796-y

Gustafsson, E., Saint Jalme, M., Kamoga, D., Mugisha, L., Snounou, G., Bomsel, M. C., & Krief, S. (2016). Food Acceptance and Social Learning Opportunities in Semi-Free Eastern Chimpanzees (Pan troglodytes schweinfurthii). Ethology, 122(2), 158–170. https://doi.org/10.1111/eth.12458

Ha, J. C., Mandell, D. J., & Gray, J. (2011). Two-item discrimination and Hamilton search learning in infant pigtailed macaque monkeys. Behavioural Processes, 86(1), 1–6. https://doi.org/10.1016/j.beproc.2010.07.010

Halina, M., Rossano, F., & Tomasello, M. (2013). The ontogenetic ritualization of bonobo gestures. Animal Cognition, 16(4), 653–666. https://doi.org/10.1007/s10071-013-0601-7

Hall, K., Oram, M. W., Campbell, M. W., Eppley, T. M., Byrne, R. W., & de Waal, F. B. M. (2017). Chimpanzee uses manipulative gaze cues to conceal and reveal information to foraging competitor. American Journal of Primatology, 79(3), 1–11. https://doi.org/10.1002/ajp.22622

Hall, K., Oram, M. W., Campbell, M. W., Eppley, T. M., Byrne, R. W., & De Waal, F. B. M. (2014). Using cross correlations to investigate how chimpanzees (Pan troglodytes) use conspecific gaze cues to extract and exploit information in a foraging competition. American Journal of Primatology, 76(10), 932–941. https://doi.org/10.1002/ajp.22279

Hall, K., Smith, M., Russell, J. L., Lambeth, S. P., Schapiro, S. J., & Brosnan, S. F. (2019). Chimpanzees Rarely Settle on Consistent Patterns of Play in the Hawk Dove, Assurance, and Prisoner’s Dilemma Games, in a Token Exchange Task. Animal Behavior and Cognition, 6(1), 48–70. https://doi.org/10.26451/abc.06.01.04.2019

Halsey, L. G., Bezerra, B. M., & Souto, A. S. (2006). Can wild common marmosets (Callithrix jacchus) solve the parallel strings task? Animal Cognition, 9(3), 229–233. https://doi.org/10.1007/s10071-006-0016-9

Hampton, R. R., & Hampstead, B. M. (2006). Spontaneous behavior of a rhesus monkey (Macaca mulatta) during memory tests suggests memory awareness. Behavioural Processes, 72(2), 184–189. https://doi.org/10.1016/j.beproc.2006.01.007

Hanus, D., & Call, J. (2011). Chimpanzee problem-solving: Contrasting the use of causal and arbitrary cues. Animal Cognition, 14(6), 871–878. https://doi.org/10.1007/s10071-011-0421-6

Hanus, D., & Call, J. (2007). Discrete Quantity Judgments in the Great Apes (Pan paniscus, Pan troglodytes, Gorilla gorilla, Pongo pygmaeus): The Effect of Presenting Whole Sets Versus Item-by-Item. Journal of Comparative Psychology, 121(3), 241–249. https://doi.org/10.1037/0735-7036.121.3.241

Hardus, M. E., Lameira, A. R., Wich, S. A., de Vries, H., Wahyudi, R., Shumaker, R. W., & Menken, S. B. J. (2015). Effect of repeated exposures and sociality on novel food acceptance and consumption by orangutans. Primates, 56(1), 21–27. https://doi.org/10.1007/s10329-014-0441-3

Harris, E. H., Gulledge, J. P., Beran, M. J., & Washburn, D. A. (2010). What Do Arabic Numerals Mean to Macaques (Macaca mulatta)? Journal of Experimental Psychology: Animal Behavior Processes, 36(1), 66–76. https://doi.org/10.1037/a0016485

Hashimoto, C., Isaji, M., Koops, K., & Furuichi, T. (2015). First records of tool-set use for ant-dipping by Eastern chimpanzees (Pan troglodytes schweinfurthii) in the Kalinzu Forest Reserve, Uganda. Primates, 56(4), 301–305. https://doi.org/10.1007/s10329-015-0478-y

Haslam, M., & Falótico, T. (2015). Nasal probe and toothpick tool use by a wild female bearded capuchin (Sapajus libidinosus). Primates, 56(3), 211–214. https://doi.org/10.1007/s10329-015-0470-6

Hassett, T. C., & Hampton, R. R. (2017). Change in the relative contributions of habit and working memory facilitates serial reversal learning expertise in rhesus monkeys. Animal Cognition, 20(3), 485–497. https://doi.org/10.1007/s10071-017-1076-8

Hattori, Y., Kuroshima, H., & Fujita, K. (2010). Tufted capuchin monkeys (Cebus apella) show understanding of human attentional states when requesting food held by a human. Animal Cognition, 13(1), 87–92. https://doi.org/10.1007/s10071-009-0248-6

Hattori, Y., Kuroshima, H., & Fujita, K. (2007). I know you are not looking at me: Capuchin monkeys’ (Cebus apella) sensitivity to human attentional states. Animal Cognition, 10(2), 141–148. https://doi.org/10.1007/s10071-006-0049-0

Hattori, Y., Leimgruber, K., Fujita, K., & De Waal, F. B. M. (2012). Food-related tolerance in capuchin monkeys (Cebus apella) varies with knowledge of the partner’s previous food-consumption. Behaviour, 149(2), 171–185. https://doi.org/10.1163/156853912X634124

Hauser, M. D., & Glynn, D. (2009). Can Free-Ranging Rhesus Monkeys (Macaca mulatta) Extract Artificially Created Rules Comprised of Natural Vocalizations? Journal of Comparative Psychology, 123(2), 161–167. https://doi.org/10.1037/a0015584

Hayashi, M. (2007). Stacking of blocks by chimpanzees: Developmental processes and physical understanding. Animal Cognition, 10(2), 89–103. https://doi.org/10.1007/s10071-006-0040-9

Hayashi, M., & Takeshita, H. (2009). Stacking of irregularly shaped blocks in chimpanzees (Pan troglodytes) and young humans (Homo sapiens). Animal Cognition, 12(1 SUPPL), 49–58. https://doi.org/10.1007/s10071-009-0273-5

Hedwig, D., Mundry, R., Robbins, M. M., & Boesch, C. (2015). Contextual correlates of syntactic variation in mountain and western gorilla close-distance vocalizations: Indications for lexical or phonological syntax? Animal Cognition, 18(2), 423–435. https://doi.org/10.1007/s10071-014-0812-6

Hedwig, D., Mundry, R., Robbins, M. M., & Boesch, C. (2015). Audience effects, but not environmental influences, explain variation in gorilla close distance vocalizations-A test of the acoustic adaptation hypothesis. American Journal of Primatology, 77(12), 1239–1252. https://doi.org/10.1002/ajp.22462

Heimbauer, L. A., Antworth, R. L., & Owren, M. J. (2012). Capuchin monkeys (Cebus apella) use positive, but not negative, auditory cues to infer food location. Animal Cognition, 15(1), 45–55. https://doi.org/10.1007/s10071-011-0430-5

Heimbauer, L. A., Conway, C. M., Christiansen, M. H., Beran, M. J., & Owren, M. J. (2018). Visual artificial grammar learning by rhesus macaques (Macaca mulatta): exploring the role of grammar complexity and sequence length. Animal Cognition, 21(2), 267–284. https://doi.org/10.1007/s10071-018-1164-4

Heimbauer, L. A., Johns, T. N., & Weiss, D. J. (2019). Inferential Reasoning in the Visual and Auditory Modalities by Cotton-Top Tamarins (Saguinus oedipus). Journal of Comparative Psychology. https://doi.org/10.1037/com0000184

Helme, A. E., Call, J., Clayton, N. S., & Emery, N. J. (2006). What do bonobos (Pan paniscus) understand about physical contact? Journal of Comparative Psychology, 120(3), 294–302. https://doi.org/10.1037/0735-7036.120.3.294

Herrmann, E., Keupp, S., Hare, B., Vaish, A., & Tomasello, M. (2013). Direct and indirect reputation formation in nonhuman great apes (Pan paniscus, pan troglodytes, Gorilla gorilla, Pongo pygmaeus) and human children (Homo sapiens). Journal of Comparative Psychology, 127(1), 63–75. https://doi.org/10.1037/a0028929

Herrmann, E., Melis, A. P., & Tomasello, M. (2006). Apes’ use of iconic cues in the object-choice task. Animal Cognition, 9(2), 118–130. https://doi.org/10.1007/s10071-005-0013-4

Herrmann, E., & Tomasello, M. (2015). Focusing and shifting attention in human children (Homo sapiens) and Chimpanzees (Pan troglodytes). Journal of Comparative Psychology, 129(3), 268–274. https://doi.org/10.1037/a0039384

Herrmann, E., Wobber, V., & Call, J. (2008). Great Apes’ (Pan troglodytes, Pan paniscus, Gorilla gorilla, Pongo pygmaeus) Understanding of Tool Functional Properties After Limited Experience. Journal of Comparative Psychology, 122(2), 220–230. https://doi.org/10.1037/0735-7036.122.2.220

Heschl, A., & Burkart, J. (2006). A new mark test for mirror self-recognition in non-human primates. Primates, 47(3), 187–198. https://doi.org/10.1007/s10329-005-0170-8

Hicks, T. C., Kühl, H. S., Boesch, C., Dieguez, P., Ayimisin, A. E., Fernandez, R. M., Zungawa, D. B., Kambere, M., Swinkels, J., Menken, S. B. J., Hart, J., Mundry, R., & Roessingh, P. (2019). Bili-Uéré: A Chimpanzee Behavioural Realm in Northern Democratic Republic of Congo. Folia Primatologica, 90(1), 3–64. https://doi.org/10.1159/000492998

Hicks, T. C., Kühl, H. S., Boesch, C., Menken, S. B. J., Hart, J., Roessingh, P., Ewango, C., & Mundry, R. (2020). The Relationship Between Tool Use and Prey Availability in Chimpanzees (Pan troglodytes schweinfurthii) of Northern Democratic Republic of Congo. International Journal of Primatology, 41(6), 936–959. https://doi.org/10.1007/s10764-020-00149-4

Higham, J. P., & Maestripieri, D. (2010). Revolutionary coalitions in male rhesus macaques. In Behaviour (Vol. 147, Issues 13–14, pp. 1889–1908). https://doi.org/10.1163/000579510X539709

Hill, A., Collier-Baker, E., & Suddendorf, T. (2011). Inferential Reasoning by Exclusion in Great Apes, Lesser Apes, and Spider Monkeys. Journal of Comparative Psychology, 125(1), 91–103. https://doi.org/10.1037/a0020867

Hirata, S. (2007). A note on the responses of chimpanzees (Pan troglodytes) to live self-images on television monitors. In Behavioural Processes (Vol. 75, Issue 1, pp. 85–90). Elsevier. https://doi.org/10.1016/j.beproc.2007.01.005

Hirata, S., & Fuwa, K. (2007). Chimpanzees (Pan troglodytes) learn to act with other individuals in a cooperative task. Primates, 48(1), 13–21. https://doi.org/10.1007/s10329-006-0022-1

Hirata, S., Fuwa, K., Sugama, K., Kusunoki, K., & Fujita, S. (2010). Facial perception of conspecifics: Chimpanzees (Pan troglodytes) preferentially attend to proper orientation and open eyes. Animal Cognition, 13(5), 679–688. https://doi.org/10.1007/s10071-010-0316-y

Hirata, S., Morimura, N., & Houki, C. (2009). How to crack nuts: Acquisition process in captive chimpanzees (Pan troglodytes) observing a model. Animal Cognition, 12(1 SUPPL), 87–101. https://doi.org/10.1007/s10071-009-0275-3

Hobaiter, C., & Byrne, R. W. (2011). The gestural repertoire of the wild chimpanzee. Animal Cognition, 14(5), 745–767. https://doi.org/10.1007/s10071-011-0409-2

Hobaiter, C., & Byrne, R. W. (2011). Serial gesturing by wild chimpanzees: Its nature and function for communication. Animal Cognition, 14(6), 827–838. https://doi.org/10.1007/s10071-011-0416-3

Hobaiter, C., Byrne, R. W., & Leavens, D. A. (2014). Deictic gesturing in wild chimpanzees (pan troglodytes)? Some possible cases. Journal of Comparative Psychology, 128(1), 82–87. https://doi.org/10.1037/a0033757

Hoffman, M. L., & Beran, M. J. (2006). Chimpanzees (Pan troglodytes) remember the location of a hidden food item after altering their orientation to a spatial array. Journal of Comparative Psychology, 120(4), 389–393. https://doi.org/10.1037/0735-7036.120.4.389

Hoffman, M. L., Beran, M. J., & Washburn, D. A. (2018). Rhesus monkeys (Macaca mulatta) remember agency information from past events and integrate this knowledge with spatial and temporal features in working memory. Animal Cognition, 21(1), 137–153. https://doi.org/10.1007/s10071-017-1147-x

Hoffman, M. L., Beran, M. J., & Washburn, D. A. (2009). Memory for “What”, “Where”, and “When” Information in Rhesus Monkeys (Macaca mulatta). Journal of Experimental Psychology: Animal Behavior Processes, 35(2), 143–152. https://doi.org/10.1037/a0013295

Hopkins, M. E. (2016). Mantled howler monkey spatial foraging decisions reflect spatial and temporal knowledge of resource distributions. Animal Cognition, 19(2), 387–403. https://doi.org/10.1007/s10071-015-0941-6

Hopkins, W. D., Mareno, M. C., Neal Webb, S. J., Schapiro, S. J., Raghanti, M. A., & Sherwood, C. C. (2021). Age-related changes in chimpanzee (Pan troglodytes) cognition: Cross-sectional and longitudinal analyses. American Journal of Primatology, 83(3), 1–9. https://doi.org/10.1002/ajp.23214

Hopkins, W. D., Mareno, M. C., & Schapiro, S. J. (2019). Further Evidence of a Left Hemisphere Specialization and Genetic Basis for Tool Use Skill in Chimpanzees (Pan troglodytes): Reproducibility in Two Genetically Isolated Populations of Apes. Journal of Comparative Psychology. https://doi.org/10.1037/com0000183

Hopkins, W. D., Taglialatela, J. P., & Leavens, D. A. (2007). Chimpanzees differentially produce novel vocalizations to capture the attention of a human. Animal Behaviour, 73(2), 281–286. https://doi.org/10.1016/j.anbehav.2006.08.004

Hopper, L. M., Lambeth, S. P., & Schapiro, S. J. (2012). An Evaluation of the Efficacy of Video Displays for Use With Chimpanzees (Pan troglodytes). American Journal of Primatology, 74(5), 442–449. https://doi.org/10.1002/ajp.22001

Hopper, L. M., Lambeth, S. P., Schapiro, S. J., & Brosnan, S. F. (2014). Social comparison mediates chimpanzees’ responses to loss, not frustration. Animal Cognition, 17(6), 1303–1311. https://doi.org/10.1007/s10071-014-0765-9

Hopper, L. M., Lambeth, S. P., Schapiro, S. J., & Whiten, A. (2015). The importance of witnessed agency in chimpanzee social learning of tool use. Behavioural Processes, 112, 120–129. https://doi.org/10.1016/j.beproc.2014.10.009

Hopper, L. M., Price, S. A., Freeman, H. D., Lambeth, S. P., Schapiro, S. J., & Kendal, R. L. (2014). Influence of personality, age, sex, and estrous state on chimpanzee problem-solving success. Animal Cognition, 17(4), 835–847. https://doi.org/10.1007/s10071-013-0715-y

Hopper, L. M., Schapiro, S. J., Lambeth, S. P., & Brosnan, S. F. (2011). Chimpanzees’ socially maintained food preferences indicate both conservatism and conformity. Animal Behaviour, 81(6), 1195–1202. https://doi.org/10.1016/j.anbehav.2011.03.002

Hopper, L. M., Spiteri, A., Lambeth, S. P., Schapiro, S. J., Horner, V., & Whiten, A. (2007). Experimental studies of traditions and underlying transmission processes in chimpanzees. Animal Behaviour, 73(6), 1021–1032. https://doi.org/10.1016/j.anbehav.2006.07.016

Hopper, L. M., Tennie, C., Ross, S. R., & Lonsdorf, E. V. (2015). Chimpanzees create and modify probe tools functionally: A study with zoo-housed chimpanzees. American Journal of Primatology, 77(2), 162–170. https://doi.org/10.1002/ajp.22319

Horner, V., & Whiten, A. (2007). Learning from others’ mistakes? Limits on understanding a trap-tube task by young chimpanzees (Pan troglodytes) and children (Homo sapiens). Journal of Comparative Psychology, 121(1), 12–21. https://doi.org/10.1037/0735-7036.121.1.12

Horton, K. E., & Caldwell, C. A. (2006). Visual co-orientation and expectations about attentional orientation in pileated gibbons (Hylobates pileatus). Behavioural Processes, 72(1), 65–73. https://doi.org/10.1016/j.beproc.2005.12.004

Hosaka, K., Nakamura, M., & Takahata, Y. (2020). Longitudinal changes in the targets of chimpanzee (Pan troglodytes) hunts at Mahale Mountains National Park: how and why did they begin to intensively hunt red colobus (Piliocolobus rufomitratus) in the 1980s? Primates, 61(3), 391–401. https://doi.org/10.1007/s10329-020-00803-8

Hostetter, A. B., Russell, J. L., Freeman, H., & Hopkins, W. D. (2007). Now you see me, now you don’t: Evidence that chimpanzees understand the role of the eyes in attention. Animal Cognition, 10(1), 55–62. https://doi.org/10.1007/s10071-006-0031-x

Howard, A. M., & Fragaszy, D. M. (2014). Multi-step routes of capuchin monkeys in a laser pointer traveling salesman task. American Journal of Primatology, 76(9), 828–841. https://doi.org/10.1002/ajp.22271

Howard, A. M., Nibbelink, N. P., Madden, M., Young, L. A., Bernardes, S., & Fragaszy, D. M. (2015). Landscape influences on the natural and artificially manipulated movements of bearded capuchin monkeys. Animal Behaviour, 106, 59–70. https://doi.org/10.1016/j.anbehav.2015.05.003

Howard, L. H., Festa, C., & Lonsdorf, E. V. (2018). Through their eyes: The influence of social models on attention and memory in capuchin monkeys (Sapajus apella). Journal of Comparative Psychology, 132(2), 210–219. https://doi.org/10.1037/com0000111

Hradec, M., Bolechová, P., & Svobodová, I. (2016). Production of a female-specific great call in an immature male gibbon, the Nomascus genus. Primates, 57(4), 445–448. https://doi.org/10.1007/s10329-016-0569-4

Hribar, A., & Call, J. (2011). Great apes use landmark cues over spatial relations to find hidden food. Animal Cognition, 14(5), 623–635. https://doi.org/10.1007/s10071-011-0397-2

Hribar, A., Haun, D., & Call, J. (2011). Great apes’ strategies to map spatial relations. Animal Cognition, 14(4), 511–523. https://doi.org/10.1007/s10071-011-0385-6

Hrubesch, C., Preuschoft, S., & Van Schaik, C. (2009). Skill mastery inhibits adoption of observed alternative solutions among chimpanzees (Pan troglodytes). Animal Cognition, 12(2), 209–216. https://doi.org/10.1007/s10071-008-0183-y

Huebner, F., & Fichtel, C. (2015). Innovation and behavioral flexibility in wild redfronted lemurs (Eulemur rufifrons). Animal Cognition, 18(3), 777–787. https://doi.org/10.1007/s10071-015-0844-6

Huffman, M. A., Spiezio, C., Sgaravatti, A., & Leca, J. B. (2010). Leaf swallowing behavior in chimpanzees (Pan troglodytes): Biased learning and the emergence of group level cultural differences. Animal Cognition, 13(6), 871–880. https://doi.org/10.1007/s10071-010-0335-8

Hughes, K. D., Mullo, E., & Santos, L. R. (2013). Solving small spaces: Investigating the use of landmark cues in brown capuchins (Cebus apella). Animal Cognition, 16(5), 803–817. https://doi.org/10.1007/s10071-013-0614-2

Hughes, K. D., & Santos, L. R. (2012). Rotational displacement skills in rhesus macaques (Macaca mulatta). Journal of Comparative Psychology, 126(4), 421–432. https://doi.org/10.1037/a0028757

Humle, T., & Snowdon, C. T. (2008). Socially biased learning in the acquisition of a complex foraging task in juvenile cottontop tamarins, Saguinus oedipus. Animal Behaviour, 75(1), 267–277. https://doi.org/10.1016/j.anbehav.2007.05.021

Humle, T., Snowdon, C. T., & Matsuzawa, T. (2009). Social influences on ant-dipping acquisition in the wild chimpanzees (Pan troglodytes verus) of Bossou, Guinea, West Africa. Animal Cognition, 12(1 SUPPL), 37–48. https://doi.org/10.1007/s10071-009-0272-6

Huskisson, S. M., Jacobson, S. L., Egelkamp, C. L., Ross, S. R., & Hopper, L. M. (2020). Using a Touchscreen Paradigm to Evaluate Food Preferences and Response to Novel Photographic Stimuli of Food in Three Primate Species (Gorilla gorilla gorilla, Pan troglodytes, and Macaca fuscata). International Journal of Primatology, 41(1), 5–23. https://doi.org/10.1007/s10764-020-00131-0

Iki, S., & Hasegawa, T. (2020). Face-to-face opening phase in Japanese macaques’ social play enhances and sustains participants’ engagement in subsequent play interaction. Animal Cognition, 23(1), 149–158. https://doi.org/10.1007/s10071-019-01325-7

Inoue, S., & Matsuzawa, T. (2011). Correlation Between Menstrual Cycle and Cognitive Performance in a Chimpanzee (Pan troglodytes). Journal of Comparative Psychology, 125(1), 104–111. https://doi.org/10.1037/a0020870

Inoue, S., & Matsuzawa, T. (2009). Acquisition and memory of sequence order in young and adult chimpanzees (Pan troglodytes). Animal Cognition, 12(1 SUPPL), 59–69. https://doi.org/10.1007/s10071-009-0274-4

Ischer, G., Zuberbühler, K., & Fedurek, P. (2020). The relationship between food calling and agonistic behaviour in wild chimpanzees. Behavioural Processes, 178, 104182. https://doi.org/10.1016/j.beproc.2020.104182

Iwata, Y. (2014). Food dropping as a food transfer mechanism among western lowland gorillas in Moukalaba-Doudou National Park, Gabon. Primates, 55(3), 353–358. https://doi.org/10.1007/s10329-014-0417-3

Izumi, A., Tsuchida, J., & Yamaguchi, C. (2013). Spontaneous alternation behavior in common marmosets (Callithrix jacchus). Journal of Comparative Psychology, 127(1), 76–81. https://doi.org/10.1037/a0026797

Jacobs, A., Maumy, M., & Petit, O. (2008). The influence of social organisation on leadership in brown lemurs (Eulemur fulvus fulvus) in a controlled environment. Behavioural Processes, 79(2), 111–113. https://doi.org/10.1016/j.beproc.2008.05.004

Jaeggi, A. V., Dunkel, L. P., van Noordwijk, M. A., Wich, S. A., Sura, A. A. L., & van Schaik, C. P. (2010). Social learning of diet and foraging skills by wild immature Bornean orangutans: Implications for culture. American Journal of Primatology, 72(1), 62–71. https://doi.org/10.1002/ajp.20752

Jaeggi, A. V., Van Noordwijk, M. A., & Van Schaik, C. P. (2008). Begging for information: Mother-offspring food sharing among wild bornean orangutans. American Journal of Primatology, 70(6), 533–541. https://doi.org/10.1002/ajp.20525

James, B. T., Webster, M. F., Menzel, C. R., Whitham, W., & Beran, M. J. (2020). Post-event misinformation effects in a language-trained chimpanzee (Pan troglodytes). Animal Cognition, 23(5), 861–869. https://doi.org/10.1007/s10071-020-01391-2

Janmaat, K. R. L., Chapman, C. A., Meijer, R., & Zuberbühler, K. (2012). The use of fruiting synchrony by foraging mangabey monkeys: A “simple tool” to find fruit. Animal Cognition, 15(1), 83–96. https://doi.org/10.1007/s10071-011-0435-0

Janmaat, K. R. L., Ban, S. D., & Boesch, C. (2013). Taï chimpanzees use botanical skills to discover fruit: What we can learn from their mistakes. Animal Cognition, 16(6), 851–860. https://doi.org/10.1007/s10071-013-0617-z

Janmaat, K. R. L., Ban, S. D., & Boesch, C. (2013). Chimpanzees use long-term spatial memory to monitor large fruit trees and remember feeding experiences across seasons. Animal Behaviour, 86(6), 1183–1205. https://doi.org/10.1016/j.anbehav.2013.09.021

Janmaat, K. R. L., Byrne, R. W., & Zuberbühler, K. (2006). Evidence for a spatial memory of fruiting states of rainforest trees in wild mangabeys. Animal Behaviour, 72(4), 797–807. https://doi.org/10.1016/j.anbehav.2005.12.009

Janson, C. H. (2007). Experimental evidence for route integration and strategic planning in wild capuchin monkeys. Animal Cognition, 10(3), 341–356. https://doi.org/10.1007/s10071-007-0079-2

Jensen, G., Alkan, Y., Muñoz, F., Ferrera, V. P., & Terrace, H. S. (2017). Transitive inference in humans (Homo sapiens) and rhesus macaques (Macaca mulatta) after massed training of the last two list items. Journal of Comparative Psychology, 131(3), 231–245. https://doi.org/10.1037/com0000065

Joly, M., Scheumann, M., & Zimmermann, E. (2008). Wild mouse lemurs revisit artificial feeding platforms: Implications for field experiments on sensory and cognitive abilities in small primates. American Journal of Primatology, 70(9), 892–896. https://doi.org/10.1002/ajp.20560

Joly, M., & Zimmermann, E. (2007). First evidence for relocation of stationary food resources during foraging in a strepsirhine primate (Microcebus murinus). American Journal of Primatology, 69(9), 1045–1052. https://doi.org/10.1002/ajp.20418

Jones, S. M., Cantlon, J. F., Merritt, D. J., & Brannon, E. M. (2010). Context affects the numerical semantic congruity effect in rhesus monkeys (Macaca mulatta). Behavioural Processes, 83(2), 191–196. https://doi.org/10.1016/j.beproc.2009.12.009

Jones, S. M., Pearson, J., DeWind, N. K., Paulsen, D., Tenekedjieva, A. M., & Brannon, E. M. (2014). Lemurs and macaques show similar numerical sensitivity. Animal Cognition, 17(3), 503–515. https://doi.org/10.1007/s10071-013-0682-3

Jordan, E. J., Townrow, L. A. J., Wright, C. I., & Seed, A. M. (2020). Understanding Solidity: Investigating Knowledge of a Functional Object Property in Brown Capuchin Monkeys (Sapajus apella) and Common Squirrel Monkeys (Saimiri sciureus). Animal Behavior and Cognition, 7(3), 365–391. https://doi.org/10.26451/abc.07.03.07.2020

Jordan, K. E., & Brannon, E. M. (2006). Weber’s Law influences numerical representations in rhesus macaques (Macaca mulatta). Animal Cognition, 9(3), 159–172. https://doi.org/10.1007/s10071-006-0017-8

Judge, P. G., & Bruno, S. (2012). Transport of Functionally Appropriate Tools by Capuchin Monkeys (Cebus apella). American Journal of Primatology, 74(3), 199–209. https://doi.org/10.1002/ajp.21987

Judge, P. G., Kurdziel, L. B., Wright, R. M., & Bohrman, J. A. (2012). Picture recognition of food by macaques (Macaca silenus). Animal Cognition, 15(3), 313–325. https://doi.org/10.1007/s10071-011-0455-9

Kaburu, S. S. K., & Newton-Fisher, N. E. (2015). Egalitarian despots: Hierarchy steepness, reciprocity and the grooming-trade model in wild chimpanzees, Pan troglodytes. Animal Behaviour, 99, 61–71. https://doi.org/10.1016/j.anbehav.2014.10.018

Kaburu, S. S. K., & Newton-Fisher, N. E. (2013). Social instability raises the stakes during social grooming among wild male chimpanzees. Animal Behaviour, 86(3), 519–527. https://doi.org/10.1016/j.anbehav.2013.06.003

Kaigaishi, Y., Nakamichi, M., & Yamada, K. (2019). High but not low tolerance populations of Japanese macaques solve a novel cooperative task. Primates, 60(5), 421–430. https://doi.org/10.1007/s10329-019-00742-z

Kaisin, O., Amaral, R. G., Bufalo, F. S., Brotcorne, F., & Culot, L. (2020). Spontaneous Tool Use by a Wild Black Lion Tamarin (Leontopithecus chrysopygus). International Journal of Primatology, 41(4), 559–561. https://doi.org/10.1007/s10764-020-00170-7

Kalan, A. K., Mundry, R., & Boesch, C. (2015). Wild chimpanzees modify food call structure with respect to tree size for a particular fruit species. Animal Behaviour, 101, 1–9. https://doi.org/10.1016/j.anbehav.2014.12.011

Kalan, A. K., & Rainey, H. J. (2009). Hand-clapping as a communicative gesture by wild female swamp gorillas. Primates, 50(3), 273–275. https://doi.org/10.1007/s10329-009-0130-9

Kangas, B. D., & Bergman, J. (2014). Repeated acquisition and discrimination reversal in the squirrel monkey (Saimiri sciureus). Animal Cognition, 17(2), 221–228. https://doi.org/10.1007/s10071-013-0654-7

Kangas, B. D., Bergman, J., & Coyle, J. T. (2016). Touchscreen assays of learning, response inhibition, and motivation in the marmoset (Callithrix jacchus). Animal Cognition, 19(3), 673–677. https://doi.org/10.1007/s10071-016-0959-4

Kanngiesser, P., & Call, J. (2010). Bonobos, chimpanzees, gorillas, and orang utans use feature and spatial cues in two spatial memory tasks. Animal Cognition, 13(3), 419–430. https://doi.org/10.1007/s10071-009-0291-3

Kanngiesser, P., Santos, L. R., Hood, B. M., & Call, J. (2011). The Limits of Endowment Effects in Great Apes (Pan paniscus, Pan troglodytes, Gorilla gorilla, Pongo pygmaeus). Journal of Comparative Psychology, 125(4), 436–445. https://doi.org/10.1037/a0024516

Kano, F., & Call, J. (2014). Cross-species variation in gaze following and conspecific preference among great apes, human infants and adults. Animal Behaviour, 91, 137–150. https://doi.org/10.1016/j.anbehav.2014.03.011

Kano, F., Call, J., & Tomonaga, M. (2012). Face and eye scanning in Gorillas (Gorilla gorilla), orangutans (Pongo abelii), and humans (Homo sapiens): Unique eye-viewing patterns in humans among hominids. Journal of Comparative Psychology, 126(4), 388–398. https://doi.org/10.1037/a0029615

Kano, F., Moore, R., Krupenye, C., Hirata, S., Tomonaga, M., & Call, J. (2018). Human ostensive signals do not enhance gaze following in chimpanzees, but do enhance object-oriented attention. Animal Cognition, 21(5), 715–728. https://doi.org/10.1007/s10071-018-1205-z

Kano, F., Tanaka, M., & Tomonaga, M. (2008). Enhanced recognition of emotional stimuli in the chimpanzee (Pan troglodytes). Animal Cognition, 11(3), 517–524. https://doi.org/10.1007/s10071-008-0142-7

Kano, F., & Tomonaga, M. (2010). Face scanning in chimpanzees and humans: continuity and discontinuity. Animal Behaviour, 79(1), 227–235. https://doi.org/10.1016/j.anbehav.2009.11.003

Kano, F., & Tomonaga, M. (2010). Attention to emotional scenes including whole-body expressions in chimpanzees (Pan troglodytes). Journal of Comparative Psychology, 124(3), 287–294. https://doi.org/10.1037/a0019146

Karg, K., Schmelz, M., Call, J., & Tomasello, M. (2016). Differing views: Can chimpanzees do Level 2 perspective-taking? Animal Cognition, 19(3), 555–564. https://doi.org/10.1007/s10071-016-0956-7

Karg, K., Schmelz, M., Call, J., & Tomasello, M. (2015). The goggles experiment: Can chimpanzees use self-experience to infer what a competitor can see? Animal Behaviour, 105, 211–221. https://doi.org/10.1016/j.anbehav.2015.04.028

Karg, K., Schmelz, M., Call, J., & Tomasello, M. (2015). Chimpanzees strategically manipulate what others can see. Animal Cognition, 18(5), 1069–1076. https://doi.org/10.1007/s10071-015-0875-z

Karg, K., Schmelz, M., Call, J., & Tomasello, M. (2014). All great ape species (gorilla gorilla, pan paniscus, pan troglodytes, pongo abelii) and two-and- a- half-year-old children (homo sapiens) discriminate appearance from reality. In Journal of Comparative Psychology (Vol. 128, Issue 4, pp. 431–439). American Psychological Association Inc. https://doi.org/10.1037/a0037385

Kasper, C., Voelkl, B., & Huber, L. (2008). Tolerated mouth-to-mouth food transfers in common marmosets. Primates, 49(2), 153–156. https://doi.org/10.1007/s10329-007-0069-7

Katsu, N., Yamada, K., & Nakamichi, M. (2017). Influence of social interactions with nonmother females on the development of call usage in Japanese macaques. Animal Behaviour, 123, 267–276. https://doi.org/10.1016/j.anbehav.2016.11.009

Katsu, N., Yamada, K., & Nakamichi, M. (2016). Function of grunts, girneys and coo calls of Japanese macaques (Macaca fuscata) in relation to call usage, age and dominance relationships. Behaviour, 153(2), 125–142. https://doi.org/10.1163/1568539X-00003330

Kaufhold, S. P., & Rossano, F. (2020). Proximate mechanisms and relational history: the interdependence of food transfers in socially housed orang-utans (Pongo abelii). Animal Behaviour, 167, 243–253. https://doi.org/10.1016/j.anbehav.2020.07.016

Kawaguchi, Y., Kano, F., & Tomonaga, M. (2019). Chimpanzees, but not bonobos, attend more to infant than adult conspecifics. Animal Behaviour, 154, 171–181. https://doi.org/10.1016/j.anbehav.2019.06.014

Kawaguchi, Y., Kuroshima, H., & Fujita, K. (2019). Age Categorization of Conspecific and Heterospecific Faces in Capuchin Monkeys (Sapajus apella). Journal of Comparative Psychology, 133(4), 502. https://doi.org/10.1037/com0000185

Kawai, N., & Koda, H. (2016). Japanese monkeys (Macaca fuscata) quickly detect snakes but not spiders: Evolutionary origins of fear-relevant animals. Journal of Comparative Psychology, 130(3), 299–303. https://doi.org/10.1037/com0000032

Kawai, N., Kubo, K., Masataka, N., & Hayakawa, S. (2016). Conserved evolutionary history for quick detection of threatening faces. Animal Cognition, 19(3), 655–660. https://doi.org/10.1007/s10071-015-0949-y

Kawai, N., Nakagami, A., Yasue, M., Koda, H., & Ichinohe, N. (2019). Common Marmosets (Callithrix jacchus) Evaluate Third- Party Social Interactions of Human Actors But Japanese Monkeys (Macaca fuscata) Do Not. Journal of Comparative Psychology. https://doi.org/10.1037/com0000182

Kazahari, N., & Agetsuma, N. (2010). Mechanisms determining relationships between feeding group size and foraging success in food patch use by Japanese macaques (Macaca fuscata). Behaviour, 147(11), 1481–1500. https://doi.org/10.1163/000579510X521573

Kazahari, N., & Agetsuma, N. (2008). Social factors enhancing foraging success of a wild group of Japanese macaques (Macaca fuscata) in a patchy food environment. Behaviour, 145(6), 843–860. https://doi.org/10.1163/156853908783929188

Kean, D., Tiddi, B., Fahy, M., Heistermann, M., Schino, G., & Wheeler, B. C. (2017). Feeling anxious? The mechanisms of vocal deception in tufted capuchin monkeys. Animal Behaviour, 130, 37–46. https://doi.org/10.1016/j.anbehav.2017.06.008

Kemp, C., & Kaplan, G. (2013). Facial expressions in common marmosets (Callithrix jacchus) and their use by conspecifics. Animal Cognition, 16(5), 773–788. https://doi.org/10.1007/s10071-013-0611-5

Kennedy, E. H., & Fragaszy, D. M. (2008). Analogical Reasoning in a Capuchin Monkey (Cebus apella). Journal of Comparative Psychology, 122(2), 167–175. https://doi.org/10.1037/0735-7036.122.2.167

Kersken, V., Zhang, D., Gomez, J.-C., Seed, A. M., & Ball, D. (2020). Capuchin Monkeys Individuate Objects Based on Spatio-temporal and Property/Kind Information: Evidence from Looking and Reaching Measures. Animal Behavior and Cognition, 7(3), 343–364. https://doi.org/10.26451/abc.07.03.06.2020

Kim, Y., Martinez, L., Choe, J. C., Lee, D. J., & Tomonaga, M. (2015). Orangutans (Pongo spp.) do not spontaneously share benefits with familiar conspecifics in a choice paradigm. Primates, 56(2), 193–200. https://doi.org/10.1007/s10329-015-0460-8

Kinani, J. F., & Zimmerman, D. (2015). Tool use for food acquisition in a wild mountain gorilla (Gorilla beringei beringei). American Journal of Primatology, 77(3), 353–357. https://doi.org/10.1002/ajp.22351

Kis, A., Gácsi, M., Range, F., & Virányi, Z. (2012). Object permanence in adult common marmosets (Callithrix jacchus): Not everything is an “A-not-B” error that seems to be one. Animal Cognition, 15(1), 97–105. https://doi.org/10.1007/s10071-011-0436-z

Kishimoto, R., Iwasaki, S., & Fujita, K. (2020). Capuchin monkeys (Sapajus apella) failed to seek information for their potential forgetting in a computerized task. Primates, 61(4), 623–632. https://doi.org/10.1007/s10329-020-00804-7

Kishimoto, R., Iwasaki, S., & Fujita, K. (2019). Do capuchins (Sapajus apella) know how well they will remember? Analysis of delay length-dependency with memory strategies. Journal of Comparative Psychology, 133(3), 340–350. https://doi.org/10.1037/com0000164

Kittler, K., Kappeler, P. M., & Fichtel, C. (2018). Instrumental problem-solving abilities in three lemur species (Microcebus murinus, Varecia variegata, and Lemur catta). Journal of Comparative Psychology, 132(3), 306–314. https://doi.org/10.1037/com0000113

Kitzmann, C. D., & Caine, N. G. (2009). Marmoset (callithrix geoffroyi) food-associated calls are functionally referential. Ethology, 115(5), 439–448. https://doi.org/10.1111/j.1439-0310.2009.01622.x

Klein, E. D., Evans, T. A., Schultz, N. B., & Beran, M. J. (2013). Learning how to “make a deal”: Human (Homo sapiens) and monkey (Macaca mulatta) performance when repeatedly faced with the monty hall dilemma. Journal of Comparative Psychology, 127(1), 103–108. https://doi.org/10.1037/a0029057

Knox, A., Markx, J., How, E., Azis, A., Hobaiter, C., van Veen, F. J. F., & Morrogh-Bernard, H. (2019). Gesture Use in Communication between Mothers and Offspring in Wild Orang-Utans (Pongo pygmaeus wurmbii) from the Sabangau Peat-Swamp Forest, Borneo. International Journal of Primatology, 40(3), 393–416. https://doi.org/10.1007/s10764-019-00095-w

Koba, R., & Izumi, A. (2008). Japanese monkeys (Macaca fuscata) discriminate between pictures of conspecific males and females without specific training. Behavioural Processes, 79(1), 70–73. https://doi.org/10.1016/j.beproc.2008.04.005

Koba, R., & Izumi, A. (2006). Sex categorization of conspecific pictures in Japanese monkeys (Macaca fuscata). Animal Cognition, 9(3), 183–191. https://doi.org/10.1007/s10071-006-0020-0

Koba, R., Takemoto, A., Miwa, M., & Nakamura, K. (2012). Characteristics of serial order learning in common marmosets (Callithrix jacchus). Journal of Comparative Psychology, 126(3), 279–287. https://doi.org/10.1037/a0026613

Koda, H., Oyakawa, C., Kato, A., & Masataka, N. (2007). Experimental evidence for the volitional control of vocal production in an immature gibbon. Behaviour, 144(6), 681–692. https://doi.org/10.1163/156853907781347817

Koda, H., Sato, A., & Kato, A. (2013). Is attentional prioritisation of infant faces unique in humans?: Comparative demonstrations by modified dot-probe task in monkeys. Behavioural Processes, 98, 31–36. https://doi.org/10.1016/j.beproc.2013.04.013

Koops, K., McGrew, W. C., & Matsuzawa, T. (2013). Ecology of culture: Do environmental factors influence foraging tool use in wild chimpanzees, Pan troglodytes verus? Animal Behaviour, 85(1), 175–185. https://doi.org/10.1016/j.anbehav.2012.10.022

Koops, K., McGrew, W. C., & Matsuzawa, T. (2010). Do chimpanzees (Pan troglodytes) use cleavers and anvils to fracture Treculia Africana fruits? Preliminary data on a new form of percussive technology. Primates, 51(2), 175–178. https://doi.org/10.1007/s10329-009-0178-6

Koops, K., Schöning, C., Mcgrew, W. C., & Matsuzawa, T. (2015). Chimpanzees prey on army ants at Seringbara, Nimba Mountains, Guinea: Predation patterns and tool use characteristics. American Journal of Primatology, 77(3), 319–329. https://doi.org/10.1002/ajp.22347

Koski, S. E., De Vries, H., Van Den Tweel, S. W., & Sterck, E. H. M. (2007). What to do after a fight? The determinants and inter-dependency of post-conflict interactions in chimpanzees. Behaviour, 144(5), 529–555. https://doi.org/10.1163/156853907780713082

Krachun, C., & Call, J. (2009). Chimpanzees (Pan troglodytes) know what can be seen from where. Animal Cognition, 12(2), 317–331. https://doi.org/10.1007/s10071-008-0192-x

Krachun, C., Lurz, R., Mahovetz, L. M., & Hopkins, W. D. (2019). Mirror self-recognition and its relationship to social cognition in chimpanzees. Animal Cognition, 22(6), 1171–1183. https://doi.org/10.1007/s10071-019-01309-7

Kralik, J. D. (2012). Rhesus macaques (Macaca mulatta) spontaneously generalize to novel quantities in a reverse-reward contingency task. Journal of Comparative Psychology, 126(3), 255–262. https://doi.org/10.1037/a0026019

Kralik, J. D., & Sampson, W. W. L. (2012). A fruit in hand is worth many more in the bush: Steep spatial discounting by free-ranging rhesus macaques (Macaca mulatta). Behavioural Processes, 89(3), 197–202. https://doi.org/10.1016/j.beproc.2011.09.010

Kret, M. E., Muramatsu, A., & Matsuzawa, T. (2018). Emotion processing across and within species: A comparison between humans (Homo sapiens) and chimpanzees (Pan troglodytes). Journal of Comparative Psychology, 132(4), 395–409. https://doi.org/10.1037/com0000108

Kubenova, B., Konecna, M., Majolo, B., Smilauer, P., Ostner, J., & Schülke, O. (2017). Triadic awareness predicts partner choice in male–infant–male interactions in Barbary macaques. Animal Cognition, 20(2), 221–232. https://doi.org/10.1007/s10071-016-1041-y

Kubo, N., Kato, A., & Nakamura, K. (2006). Deterioration of planning ability with age in Japanese monkeys (Macaca fuscata). Journal of Comparative Psychology, 120(4), 449–455. https://doi.org/10.1037/0735-7036.120.4.449

Kubo-Kawai, N., & Kawai, N. (2007). Interference effects by spatial proximity and age-related declines in spatial memory by Japanese monkeys (Macaca fuscata): Deficits in the combined use of multiple spatial cues. Journal of Comparative Psychology, 121(2), 189–197. https://doi.org/10.1037/0735-7036.121.2.189

Kumpan, L. T., Smeltzer, E. A., & Teichroeb, J. A. (2020). Animal cognition in the field: performance of wild vervet monkeys (Chlorocebus pygerythrus) on a reversal learning task. Animal Cognition, 23(3), 523–534. https://doi.org/10.1007/s10071-020-01356-5

Kuroshima, H., Kaiser, I., & Fragaszy, D. M. (2014). Does own experience affect perception of others’ actions in capuchin monkeys (Cebus apella)? Animal Cognition, 17(6), 1269–1279. https://doi.org/10.1007/s10071-014-0760-1

Kuroshima, H., Kuwahata, H., & Fujita, K. (2008). Learning from others’ mistakes in capuchin monkeys (Cebus apella). Animal Cognition, 11(4), 599–609. https://doi.org/10.1007/s10071-008-0150-7

la Cour, L. T., Stone, B. W., Hopkins, W., Menzel, C., & Fragaszy, D. M. (2014). What limits tool use in nonhuman primates? Insights from tufted capuchin monkeys (Sapajus spp.) and chimpanzees (Pan troglodytes) aligning three-dimensional objects to a surface. Animal Cognition, 17(1), 113–125. https://doi.org/10.1007/s10071-013-0643-x

LaClair, M., & Lacreuse, A. (2016). Reversal learning in gonadectomized marmosets with and without hormone replacement: are males more sensitive to punishment? Animal Cognition, 19(3), 619–630. https://doi.org/10.1007/s10071-016-0966-5

Lacreuse, A., Schatz, K., Strazzullo, S., King, H. M., & Ready, R. (2013). Attentional biases and memory for emotional stimuli in men and male rhesus monkeys. Animal Cognition, 16(6), 861–871. https://doi.org/10.1007/s10071-013-0618-y

Laidre, M. E. (2008). Spontaneous performance of wild baboons on three novel food-access puzzles. Animal Cognition, 11(2), 223–230. https://doi.org/10.1007/s10071-007-0104-5

Laidre, M. E. (2008). Do captive mandrills invent new gestures? Animal Cognition, 11(2), 179–187. https://doi.org/10.1007/s10071-007-0121-4

Laidre, M. E. (2006). Manipulation without mind-reading: Information suppression and leakage during food discovery by mandrills (Mandrillus sphinx). Behaviour, 143(3), 365–392. https://doi.org/10.1163/156853906775897851

Lamaury, A., Cochet, H., & Bourjade, M. (2019). Acquisition of joint attention by olive baboons gesturing toward humans. Animal Cognition, 22(4), 567–575. https://doi.org/10.1007/s10071-017-1111-9

Lamon, N., Neumann, C., & Zuberbühler, K. (2018). Development of object manipulation in wild chimpanzees. Animal Behaviour, 135, 121–130. https://doi.org/10.1016/j.anbehav.2017.11.003

Laporte, M. N. C., & Zuberbühler, K. (2010). Vocal greeting behaviour in wild chimpanzee females. Animal Behaviour, 80(3), 467–473. https://doi.org/10.1016/j.anbehav.2010.06.005

Large, E. W., & Gray, P. M. (2015). Spontaneous tempo and rhythmic entrainment in a bonobo (Pan paniscus). Journal of Comparative Psychology, 129(4), 317–328. https://doi.org/10.1037/com0000011

Larson, S. M., Ruiz-Lambides, A., Platt, M. L., & Brent, L. J. N. (2018). Social network dynamics precede a mass eviction in group-living rhesus macaques. Animal Behaviour, 136, 185–193. https://doi.org/10.1016/j.anbehav.2017.08.019

le Roux, A., Mathibane, N., & Nowak, K. (2019). Wild Samango Monkeys, Cercopithecus mitis, Balance Risk and Opportunity to Interact with Novel Objects in Village Gardens. International Journal of Primatology, 40(6), 661–670. https://doi.org/10.1007/s10764-019-00113-x

Leavens, D. A., Ely, J., Hopkins, W. D., & Bard, K. A. (2012). Effects of cage mesh on pointing: Hand shapes in chimpanzees (Pan troglodytes). Animal Cognition, 15(3), 437–441. https://doi.org/10.1007/s10071-011-0466-6

Leavens, D. A., Russell, J. L., & Hopkins, W. D. (2010). Multimodal communication by captive chimpanzees (Pan troglodytes). Animal Cognition, 13(1), 33–40. https://doi.org/10.1007/s10071-009-0242-z

Leca, J. B., Gunst, N., & Huffman, M. A. (2010). The first case of dental flossing by a Japanese macaque (Macaca fuscata): Implications for the determinants of behavioral innovation and the constraints on social transmission. Primates, 51(1), 13–22. https://doi.org/10.1007/s10329-009-0159-9

Leca, J. B., Gunst, N., & Huffman, M. A. (2010). Indirect social influence in the maintenance of the stone-handling tradition in Japanese macaques, Macaca fuscata. Animal Behaviour, 79(1), 117–126. https://doi.org/10.1016/j.anbehav.2009.09.035

Leca, J. B., Gunst, N., & Huffman, M. A. (2008). Food provisioning and stone handling tradition in Japanese macaques: a comparative study of ten troops. American Journal of Primatology, 70(8), 803–813. https://doi.org/10.1002/ajp.20551

Leca, J. B., Gunst, N., & Huffman, M. A. (2007). Japanese macaque cultures: Inter- and intra-troop behavioural variability of stone handling patterns across 10 troops. Behaviour, 144(3), 251–281. https://doi.org/10.1163/156853907780425712

Leca, J. B., Gunst, N., Watanabe, K., & Huffman, M. A. (2007). A new case of fish-eating in Japanese macaques: Implications for social constraints on the diffusion of feeding innovation. American Journal of Primatology, 69(7), 821–828. https://doi.org/10.1002/ajp.20401

Leca, J.-B., Gunst, N., Carrier, L. O., & Vasey, P. L. (2014). Inter-Group Variation in Non-Conceptive Sexual Activity in Female Japanese Macaques: Could it be Cultural? Animal Behavior and Cognition, 1(3), 387. https://doi.org/10.12966/abc.08.12.2014

LeFauve, M., & Margulis, S. (2015). Functionality in Tool Use in Western Lowland Gorillas (Gorilla gorilla gorilla). Animal Behavior and Cognition, 2(1), 96–104. https://doi.org/10.12966/abc.02.08.2015

Legrain, L., Stevens, J., Alegria Iscoa, J., & Destrebecqz, A. (2012). A case study of conflict management in bonobos: How does a bonobo (Pan paniscus) mother manage conflicts between her sons and her female coalition partner? Folia Primatologica, 82(4–5), 236–243. https://doi.org/10.1159/000334818

Lehner, S. R., Burkart, J. M., & van Schaik, C. P. (2011). Can Captive Orangutans (Pongo pygmaeus abelii) Be Coaxed Into Cumulative Build-Up of Techniques? Journal of Comparative Psychology, 125(4), 446–455. https://doi.org/10.1037/a0024413

Leising, K. J., Elmore, L. C., Rivera, J. J., Magnotti, J. F., Katz, J. S., & Wright, A. A. (2013). Testing visual short-term memory of pigeons (Columba livia) and a rhesus monkey (Macaca mulatta) with a location change detection task. Animal Cognition, 16(5), 839–844. https://doi.org/10.1007/s10071-013-0644-9

Lemasson, A., Guilloux, M., Rizaldi, Barbu, S., Lacroix, A., & Koda, H. (2013). Age- and sex-dependent contact call usage in Japanese macaques. Primates, 54(3), 283–291. https://doi.org/10.1007/s10329-013-0347-5

Leone, A., Mignini, M., Mancini, G., & Palagi, E. (2010). Aggression does not increase friendly contacts among bystanders in geladas (Theropithecus gelada). Primates, 51(4), 299–305. https://doi.org/10.1007/s10329-010-0204-8

Lewis, A., Bernsten, D., & Call, J. (2018). Remembering past exchanges: Apes fail to use social cues. Animal Behavior and Cognition, 5(1), 19–40. https://doi.org/10.26451/abc.05.01.03.2018

Liebal, K., & Kaminski, J. (2012). Gibbons (Hylobates pileatus, H. moloch, H. lar, Symphalangus syndactylus) follow human gaze, but do not take the visual perspective of others. Animal Cognition, 15(6), 1211–1216. https://doi.org/10.1007/s10071-012-0543-5

Liebal, K., & Rossano, F. (2017). The give and take of food sharing in Sumatran orang-utans, Pongo abelii, and chimpanzees, Pan troglodytes. Animal Behaviour, 133, 91–100. https://doi.org/10.1016/j.anbehav.2017.09.006

Lindshield, S. M., & Rodrigues, M. A. (2009). Tool use in wild spider monkeys (Ateles geoffroyi). Primates, 50(3), 269–272. https://doi.org/10.1007/s10329-009-0144-3

Liu, Q., Fragaszy, D., Wright, B., Wright, K., Izar, P., & Visalberghi, E. (2011). Wild bearded capuchin monkeys (Cebus libidinosus) place nuts in anvils selectively. Animal Behaviour, 81(1), 297–305. https://doi.org/10.1016/j.anbehav.2010.10.021

Livingstone, M. S., Srihasam, K., & Morocz, I. A. (2010). The benefit of symbols: Monkeys show linear, human-like, accuracy when using symbols to represent scalar value. Animal Cognition, 13(5), 711–719. https://doi.org/10.1007/s10071-010-0321-1

Locurto, C., Dillon, L., Collins, M., Conway, M., & Cunningham, K. (2013). Implicit chaining in cotton-top tamarins (Saguinus oedipus) with elements equated for probability of reinforcement. Animal Cognition, 16(4), 611–625. https://doi.org/10.1007/s10071-013-0598-y

Locurto, C., Gagne, M., & Levesque, K. (2009). Implicit Chaining in Cotton-Top Tamarins (Saguinus oedipus). Journal of Experimental Psychology: Animal Behavior Processes, 35(1), 116–122. https://doi.org/10.1037/a0012924

Locurto, C., Gagne, M., & Nutile, L. (2010). Characteristics of implicit chaining in cotton-top tamarins (Saguinus oedipus). Animal Cognition, 13(4), 617–629. https://doi.org/10.1007/s10071-010-0312-2

Lonsdorf, E. V., Bonnie, K. E., Grim, M., Krupnick, A., Prestipino, M., & Whyte, J. (2016). Seeding an arbitrary convention in capuchin monkeys: The effect of social context. Behaviour, 153(5), 633–654. https://doi.org/10.1163/1568539X-00003368

Lonsdorf, E. V., Ross, S. R., Linick, S. A., Milstein, M. S., & Melber, T. N. (2009). An experimental, comparative investigation of tool use in chimpanzees and gorillas. Animal Behaviour, 77(5), 1119–1126. https://doi.org/10.1016/j.anbehav.2009.01.020

Lonsdorf, E. V. (2006). What is the role of mothers in the acquisition of termite-fishing behaviors in wild chimpanzees (Pan troglodytes schweinfurthii)? Animal Cognition, 9(1), 36–46. https://doi.org/10.1007/s10071-005-0002-7

Luef, E. M., & Heschl, A. (2017). Triadic interactions with tools in a gorilla. Animal Behavior and Cognition, 4(2), 136–145. https://doi.org/10.12966/abc.01.05.2017

Luef, E. M., & Liebal, K. (2012). Infant-Directed Communication in Lowland Gorillas (Gorilla gorilla): Do Older Animals Scaffold Communicative Competence in Infants? American Journal of Primatology, 74(9), 841–852. https://doi.org/10.1002/ajp.22039

Luef, E. M., & Pika, S. (2019). Social relationships and greetings in wild chimpanzees (Pan troglodytes): use of signal combinations. Primates, 60(6), 507–515. https://doi.org/10.1007/s10329-019-00758-5

Lührs, M. L., Dammhahn, M., Kappeler, P. M., & Fichtel, C. (2009). Spatial memory in the grey mouse lemur (Microcebus murinus). Animal Cognition, 12(4), 599–609. https://doi.org/10.1007/s10071-009-0219-y

Luncz, L. V., & Boesch, C. (2014). Tradition over trend: Neighboring chimpanzee communities maintain differences in cultural behavior despite frequent immigration of adult females. American Journal of Primatology, 76(7), 649–657. https://doi.org/10.1002/ajp.22259

Luncz, L. V., Sirianni, G., Mundry, R., & Boesch, C. (2018). Costly culture: differences in nut-cracking efficiency between wild chimpanzee groups. Animal Behaviour, 137, 63–73. https://doi.org/10.1016/j.anbehav.2017.12.017

Luncz, L. V., Svensson, M. S., Haslam, M., Malaivijitnond, S., Proffitt, T., & Gumert, M. (2017). Technological Response of Wild Macaques (Macaca fascicularis) to Anthropogenic Change. International Journal of Primatology, 38(5), 872–880. https://doi.org/10.1007/s10764-017-9985-6

Lurz, R., Krachun, C., Mahovetz, L., Wilson, M. J. G., & Hopkins, W. (2018). Chimpanzees gesture to humans in mirrors: using reflection to dissociate seeing from line of gaze. Animal Behaviour, 135, 239-IBC. https://doi.org/10.1016/j.anbehav.2017.11.014

Lyn, H., Russell, J. L., Leavens, D. A., Bard, K. A., Boysen, S. T., Schaeffer, J. A., & Hopkins, W. D. (2014). Apes communicate about absent and displaced objects: Methodology matters. Animal Cognition, 17(1), 85–94. https://doi.org/10.1007/s10071-013-0640-0

Macé, M. J. M., Delorme, A., Richard, G., & Fabre-Thorpe, M. (2010). Spotting animals in natural scenes: Efficiency of humans and monkeys at very low contrasts. Animal Cognition, 13(3), 405–418. https://doi.org/10.1007/s10071-009-0290-4

Macellini, S., Ferrari, P. F., Bonini, L., Fogassi, L., & Paukner, A. (2010). A modified mark test for own-body recognition in pig-tailed macaques (Macaca nemestrina). Animal Cognition, 13(4), 631–639. https://doi.org/10.1007/s10071-010-0313-1

Machanda, Z. P., Gilby, I. C., & Wrangham, R. W. (2014). Mutual grooming among adult male chimpanzees: The immediate investment hypothesis. Animal Behaviour, 87(C), 165–174. https://doi.org/10.1016/j.anbehav.2013.10.028

MacLean, E. L., & Hare, B. (2015). Bonobos and chimpanzees exploit helpful but not prohibitive gestures. Behaviour, 152(3–4), 493–520. https://doi.org/10.1163/1568539X-00003203

MacLean, E. L., & Hare, B. (2012). Bonobos and chimpanzees infer the target of another’s attention. Animal Behaviour, 83(2), 345–353. https://doi.org/10.1016/j.anbehav.2011.10.026

MacLean, E. L., Mandalaywala, T. M., & Brannon, E. M. (2012). Variance-sensitive choice in lemurs: Constancy trumps quantity. Animal Cognition, 15(1), 15–25. https://doi.org/10.1007/s10071-011-0425-2

MacLean, E. L., Merritt, D. J., & Brannon, E. M. (2008). Social complexity predicts transitive reasoning in prosimian primates. Animal Behaviour, 76(2), 479–486. https://doi.org/10.1016/j.anbehav.2008.01.025

MacLean, E., & Hare, B. (2013). Spontaneous triadic engagement in bonobos (pan paniscus) and chimpanzees (pan troglodytes). Journal of Comparative Psychology, 127(3), 245–255. https://doi.org/10.1037/a0030935

Maille, A., & Roeder, J. J. (2012). Inferences about the location of food in lemurs (Eulemur macaco and Eulemur fulvus): A comparison with apes and monkeys. Animal Cognition, 15(6), 1075–1083. https://doi.org/10.1007/s10071-012-0531-9

Majecka, K., & Pietraszewski, D. (2018). Where’s the cookie? The ability of monkeys to track object transpositions. Animal Cognition, 21(4), 603–611. https://doi.org/10.1007/s10071-018-1195-x

Majolo, B., Schino, G., & Aureli, F. (2012). The relative prevalence of direct, indirect and generalized reciprocity in macaque grooming exchanges. Animal Behaviour, 83(3), 763–771. https://doi.org/10.1016/j.anbehav.2011.12.026

Malaivijitnond, S., Lekprayoon, C., Tandavanittj, N., Panha, S., Cheewatham, C., & Hamada, Y. (2007). Stone-tool usage by Thai long-tailed macaques (Macaca fascicularis). American Journal of Primatology, 69(2), 227–233. https://doi.org/10.1002/ajp.20342

Malassis, R., Gheusi, G., & Fagot, J. (2015). Assessment of metacognitive monitoring and control in baboons (Papio papio). Animal Cognition, 18(6), 1347–1362. https://doi.org/10.1007/s10071-015-0907-8

Mallavarapu, S., Perdue, B. M., Stoinski, T. S., & Maple, T. L. (2013). Can Black-and-White Ruffed Lemurs (Varecia variegata) Solve Object Permanence Tasks? American Journal of Primatology, 75(4), 376–386. https://doi.org/10.1002/ajp.22118

Mallavarapu, S., Stoinski, T. S., Perdue, B. M., & Maple, T. L. (2014). Double invisible displacement understanding in orangutans: testing in non-locomotor and locomotor space. Primates, 55(4), 549–557. https://doi.org/10.1007/s10329-014-0439-x

Manaka, Y., & Sugita, Y. (2009). Insufficient visual information leads to spontaneous bipedal walking in Japanese monkeys. Behavioural Processes, 80(1), 104–106. https://doi.org/10.1016/j.beproc.2008.10.010

Mandl, I., Schwitzer, C., & Holderied, M. (2019). Sahamalaza Sportive Lemur, Lepilemur sahamalaza, Vocal Communication: Call Use, Context and Gradation. Folia Primatologica, 90(5), 336–360. https://doi.org/10.1159/000493939

Mangalam, M., Izar, P., Visalberghi, E., & Fragaszy, D. M. (2016). Task-specific temporal organization of percussive movements in wild bearded capuchin monkeys. Animal Behaviour, 114, 129–137. https://doi.org/10.1016/j.anbehav.2016.01.011

Mangalam, M., Roles, L. K. R., & Fragaszy, D. M. (2020). Distinct perceptuomotor features of percussive tooling in humans (Homo sapiens) and wild bearded capuchin monkeys (Sapajus libidinosus). Journal of Comparative Psychology, 134(1), 84–97. https://doi.org/10.1037/com0000197

Mannu, M., & Ottoni, E. B. (2009). The enhanced tool-kit of two groups of wild bearded capuchin monkeys in the caatinga: Tool making, associative use, and secondary tools. American Journal of Primatology, 71(3), 242–251. https://doi.org/10.1002/ajp.20642

Manrique, H. M., & Call, J. (2015). Age-dependent cognitive inflexibility in great apes. Animal Behaviour, 102, 1–6. https://doi.org/10.1016/j.anbehav.2015.01.002

Manrique, H. M., & Call, J. (2011). Spontaneous use of tools as straws in great apes. Animal Cognition, 14(2), 213–226. https://doi.org/10.1007/s10071-010-0355-4

Manrique, H. M., Gross, A. N. M., & Call, J. (2010). Great Apes Select Tools on the Basis of Their Rigidity. Journal of Experimental Psychology: Animal Behavior Processes, 36(4), 409–422. https://doi.org/10.1037/a0019296

Manrique, H. M., Sabbatini, G., Call, J., & Visalberghi, E. (2011). Tool choice on the basis of rigidity in capuchin monkeys. Animal Cognition, 14(6), 775–786. https://doi.org/10.1007/s10071-011-0410-9

Manrique, H. M., Völter, C. J., & Call, J. (2013). Repeated innovation in great apes. Animal Behaviour, 85(1), 195–202. https://doi.org/10.1016/j.anbehav.2012.10.026

Marechal, L., Genty, E., & Roeder, J. J. (2010). Recognition of faces of known individuals in two lemur species (Eulemur fulvus and E. macaco). Animal Behaviour, 79(5), 1157–1163. https://doi.org/10.1016/j.anbehav.2010.02.022

Marsh, H. L. (2014). Metacognitive-like information seeking in lion-tailed macaques: A generalized search response after all? Animal Cognition, 17(6), 1313–1328. https://doi.org/10.1007/s10071-014-0767-7

Marsh, H. L., Adams, L., Floyd, C., & MacDonald, S. E. (2013). Feature versus spatial strategies by orangutans (pongo abelii) and human children (Homo sapiens) in a cross-dimensional task. Journal of Comparative Psychology, 127(2), 128–141. https://doi.org/10.1037/a0030591

Marsh, H. L., & Macdonald, S. E. (2012). Orangutans (Pongo abelii) “play the odds”: Information-seeking strategies in relation to cost, risk, and benefit. Journal of Comparative Psychology, 126(3), 263–278. https://doi.org/10.1037/a0025906

Marsh, H. L., & MacDonald, S. E. (2012). Information seeking by orangutans: A generalized search strategy? Animal Cognition, 15(3), 293–304. https://doi.org/10.1007/s10071-011-0453-y

Marsh, H. L., & MacDonald, S. E. (2008). The use of perceptual features in categorization by orangutans (Pongo abelli). Animal Cognition, 11(4), 569–585. https://doi.org/10.1007/s10071-008-0148-1

Marsh, H. L., Spetch, M. L., & MacDonald, S. E. (2011). Strategies in landmark use by orangutans and human children. Animal Cognition, 14(4), 487–502. https://doi.org/10.1007/s10071-011-0382-9

Marsh, H. L., Vining, A. Q., Levendoski, E. K., & Judge, P. G. (2015). Inference by exclusion in lion-tailed macaques (Macaca silenus), a hamadryas baboon (Papio hamadryas), capuchins (Sapajus apella), and squirrel monkeys (Saimiri sciureus). Journal of Comparative Psychology, 129(3), 256–267. https://doi.org/10.1037/a0039316

Marshall-Pescini, S., & Whiten, A. (2008). Chimpanzees (Pan troglodytes) and the question of cumulative culture: An experimental approach. Animal Cognition, 11(3), 449–456. https://doi.org/10.1007/s10071-007-0135-y

Martin, C. F., Biro, D., & Matsuzawa, T. (2011). Chimpanzees’ use of conspecific cues in matching-to-sample tasks: Public information use in a fully automated testing environment. Animal Cognition, 14(6), 893–902. https://doi.org/10.1007/s10071-011-0424-3

Martinez, L., & Matsuzawa, T. (2009). Effect of species-specificity in auditory-visual intermodal matching in a chimpanzee (Pan troglodytes) and humans. Behavioural Processes, 82(2), 160–163. https://doi.org/10.1016/j.beproc.2009.06.014

Martinez, L., & Matsuzawa, T. (2009). Auditory-visual intermodal matching based on individual recognition in a chimpanzee (Pan troglodytes). Animal Cognition, 12(1 SUPPL), 71–85. https://doi.org/10.1007/s10071-009-0269-1

Martin-Malivel, J. (2011). Discrimination of contour-deleted images in baboons (Papio papio) and chimpanzees (Pan troglodytes). Animal Cognition, 14(3), 415–426. https://doi.org/10.1007/s10071-010-0376-z

Martin-Ordas, G., Call, J., & Colmenares, F. (2008). Tubes, tables and traps: Great apes solve two functionally equivalent trap tasks but show no evidence of transfer across tasks. Animal Cognition, 11(3), 423–430. https://doi.org/10.1007/s10071-007-0132-1

Martin-Ordas, G., Haun, D., Colmenares, F., & Call, J. (2010). Keeping track of time: Evidence for episodic-like memory in great apes. Animal Cognition, 13(2), 331–340. https://doi.org/10.1007/s10071-009-0282-4

Martin-Ordas, G., Jaek, F., & Call, J. (2012). Barriers and traps: Great apes’ performance in two functionally equivalent tasks. Animal Cognition, 15(5), 1007–1013. https://doi.org/10.1007/s10071-012-0504-z

Masi, S. (2020). Reaction to allospecific death and to an unanimated gorilla infant in wild western gorillas: insights into death recognition and prolonged maternal carrying. Primates, 61(1), 83–92. https://doi.org/10.1007/s10329-019-00745-w

Massaro, L., Liu, Q., Visalberghi, E., & Fragaszy, D. (2012). Wild bearded capuchin (Sapajus libidinosus) select hammer tools on the basis of both stone mass and distance from the anvil. Animal Cognition, 15(6), 1065–1074. https://doi.org/10.1007/s10071-012-0530-x

Massen, J. J. M., Luyten, I. J. A. F., Spruijt, B. M., & Sterck, E. H. M. (2011). Benefiting friends or dominants: Prosocial choices mainly depend on rank position in long-tailed macaques (Macaca fascicularis). Primates, 52(3), 237–247. https://doi.org/10.1007/s10329-011-0244-8

Massen, J. J. M., Van Den Berg, L. M., Spruijt, B. M., & Sterck, E. H. M. (2012). Inequity aversion in relation to effort and relationship quality in long-tailed Macaques (Macaca fascicularis). American Journal of Primatology, 74(2), 145–156. https://doi.org/10.1002/ajp.21014

Matsuno, T., & Tomonaga, M. (2007). An advantage for concavities in shape perception by chimpanzees (Pan troglodytes). Behavioural Processes, 75(3), 253–258. https://doi.org/10.1016/j.beproc.2007.02.028

Matsusaka, T., Nishie, H., Shimada, M., Kutsukake, N., Zamma, K., Nakamura, M., & Nishida, T. (2006). Tool-use for drinking water by immature chimpanzees of Mahale: Prevalence of an unessential behavior. Primates, 47(2), 113–122. https://doi.org/10.1007/s10329-005-0158-4

Matthews, L. J. (2009). Intragroup behavioral variation in white-fronted capuchin monkeys (Cebus albifrons): Mixed evidence for social learning inferred from new and established analytical methods. Behaviour, 146(3), 295–324. https://doi.org/10.1163/156853909X410937

Matthews, S., & Snowdon, C. T. (2011). Long-term memory for calls of relatives in cotton-top tamarins (Saguinus oedipus). Journal of Comparative Psychology, 125(3), 366–369. https://doi.org/10.1037/a0023149

Maugard, A., Marzouki, Y., & Fagot, J. (2013). Contribution of working memory processes to relational matching-to-sample performance in baboons (papio papio). Journal of Comparative Psychology, 127(4), 370–379. https://doi.org/10.1037/a0032336

Maugard, A., Wasserman, E. A., Castro, L., & Fagot, J. (2014). Effects of training condition on the contribution of specific items to relational processing in baboons (Papio papio). Animal Cognition, 17(4), 911–924. https://doi.org/10.1007/s10071-013-0724-x

Mazumder, J., & Kaburu, S. S. K. (2020). Object Manipulation and Tool Use in Nicobar Long-Tailed Macaques (Macaca fascicularis umbrosus). International Journal of Primatology, 41(1), 141–159. https://doi.org/10.1007/s10764-020-00141-y

McAuliffe, K., Chang, L. W., Leimgruber, K. L., Spaulding, R., Blake, P. R., & Santos, L. R. (2015). Capuchin monkeys, Cebus apella, show no evidence for inequity aversion in a costly choice task. Animal Behaviour, 103, 65–74. https://doi.org/10.1016/j.anbehav.2015.02.014

McAuliffe, K., Shelton, N., & Stone, L. (2014). Does effort influence inequity aversion in cotton-top tamarins (Saguinus oedipus)? Animal Cognition, 17(6), 1289–1301. https://doi.org/10.1007/s10071-014-0764-x

McCarthy, M. S., Jensvold, M. L. A., & Fouts, D. H. (2013). Use of gesture sequences in captive chimpanzee (Pan troglodytes) play. Animal Cognition, 16(3), 471–481. https://doi.org/10.1007/s10071-012-0587-6

McGuire, M. C., & Vonk, J. (2018). Gorillas (Gorilla gorilla gorilla) fail to learn abstract cues of differential outcomes in a novel cognitive bias test. Animal Behavior and Cognition, 5(1), 103–117. https://doi.org/10.26451/abc.05.01.08.2018

McLendon, S., & Amoroso, C. R. (2020). Social Influences on Food Avoidance in Lemur catta. Folia Primatologica, 91(6), 622–629. https://doi.org/10.1159/000509101

McLennan, M. R. (2018). Tie one on: ‘nest tying’ by wild chimpanzees at Bulindi—a variant of a universal great ape behavior? Primates, 59(3), 227–233. https://doi.org/10.1007/s10329-018-0658-7

McLennan, M. R. (2014). Chimpanzee insectivory in the northern half of Uganda’s Rift Valley: Do Bulindi chimpanzees conform to a regional pattern? Primates, 55(2), 173–178. https://doi.org/10.1007/s10329-014-0408-4

McLennan, M. R. (2011). Tool-use to obtain honey by chimpanzees at Bulindi: New record from Uganda. Primates, 52(4), 315–322. https://doi.org/10.1007/s10329-011-0254-6

Méary, D., Li, Z., Li, W., Guo, K., & Pascalis, O. (2014). Seeing two faces together: preference formation in humans and rhesus macaques. Animal Cognition, 17(5), 1107–1119. https://doi.org/10.1007/s10071-014-0742-3

Medam, T., & Fagot, J. (2016). Behavioral assessment of combinatorial semantics in baboons (Papio papio). Behavioural Processes, 123, 54–62. https://doi.org/10.1016/j.beproc.2015.10.016

Medam, T., Marzouki, Y., Montant, M., & Fagot, J. (2016). Categorization does not promote symmetry in Guinea baboons (Papio papio). Animal Cognition, 19(5), 987–998. https://doi.org/10.1007/s10071-016-1003-4

Melis, A. P., Call, J., & Tomasello, M. (2006). Chimpanzees (Pan troglodytes) conceal visual and auditory information from others. Journal of Comparative Psychology, 120(2), 154–162. https://doi.org/10.1037/0735-7036.120.2.154

Melis, A. P., Hare, B., & Tomasello, M. (2009). Chimpanzees coordinate in a negotiation game. Evolution and Human Behavior, 30(6), 381–392. https://doi.org/10.1016/j.evolhumbehav.2009.05.003

Melis, A. P., Hare, B., & Tomasello, M. (2008). Do chimpanzees reciprocate received favours? Animal Behaviour, 76(3), 951–962. https://doi.org/10.1016/j.anbehav.2008.05.014

Melis, A. P., Hare, B., & Tomasello, M. (2006). Engineering cooperation in chimpanzees: tolerance constraints on cooperation. Animal Behaviour, 72(2), 275–286. https://doi.org/10.1016/j.anbehav.2005.09.018

Melis, A. P., Schneider, A. C., & Tomasello, M. (2011). Chimpanzees, Pan troglodytes, share food in the same way after collaborative and individual food acquisition. Animal Behaviour, 82(3), 485–493. https://doi.org/10.1016/j.anbehav.2011.05.024

Mendes, N., & Call, J. (2014). Chimpanzees form long-term memories for food locations after limited exposure. American Journal of Primatology, 76(5), 485–495. https://doi.org/10.1002/ajp.22248

Mendes, N., Rakoczy, H., & Call, J. (2011). Primates do not spontaneously use shape properties for object individuation: A competence or a performance problem? Animal Cognition, 14(3), 407–414. https://doi.org/10.1007/s10071-010-0375-0

Meno, W., Coss, R. G., & Perry, S. (2013). Development of Snake-Directed Antipredator Behavior by Wild White-Faced Capuchin Monkeys: II. Influence of the Social Environment. American Journal of Primatology, 75(3), 292–300. https://doi.org/10.1002/ajp.22109

Mercier, S., Neumann, C., van de Waal, E., Chollet, E., Meric de Bellefon, J., & Zuberbühler, K. (2017). Vervet monkeys greet adult males during high-risk situations. Animal Behaviour, 132, 229–245. https://doi.org/10.1016/j.anbehav.2017.07.021

Merritt, D. J., & Terrace, H. S. (2011). Mechanisms of Inferential Order Judgments in Humans (Homo sapiens) and Rhesus Monkeys (Macaca mulatta). Journal of Comparative Psychology, 125(2), 227–238. https://doi.org/10.1037/a0021572

Merritt, D., MacLean, E. L., Jaffe, S., & Brannon, E. M. (2007). A Comparative Analysis of Serial Ordering in Ring-Tailed Lemurs (Lemur catta). Journal of Comparative Psychology, 121(4), 363–371. https://doi.org/10.1037/0735-7036.121.4.363

Meunier, H., Leca, J. B., Deneubourg, J. L., & Petit, O. (2006). Group movement decisions in capuchin monkeys: The utility of an experimental study and a mathematical model to explore the relationship between individual and collective behaviours. Behaviour, 143(12), 1511–1527. https://doi.org/10.1163/156853906779366982

Meunier, H., Prieur, J., & Vauclair, J. (2013). Olive baboons communicate intentionally by pointing. Animal Cognition, 16(2), 155–163. https://doi.org/10.1007/s10071-012-0558-y

Micheletta, J., & Waller, B. M. (2012). Friendship affects gaze following in a tolerant species of macaque, Macaca nigra. Animal Behaviour, 83(2), 459–467. https://doi.org/10.1016/j.anbehav.2011.11.018

Micheletta, J., Whitehouse, J., Parr, L. A., & Waller, B. M. (2015). Facial expression recognition in crested macaques (Macaca nigra). Animal Cognition, 18(4), 985–990. https://doi.org/10.1007/s10071-015-0867-z

Mielke, A., Crockford, C., & Wittig, R. M. (2019). Snake alarm calls as a public good in sooty mangabeys. Animal Behaviour, 158, 201–209. https://doi.org/10.1016/j.anbehav.2019.10.001

Miller, C. T., Mandel, K., & Wang, X. (2010). The communicative content of the common marmoset phee call during antiphonal calling. American Journal of Primatology, 72(11), 974–980. https://doi.org/10.1002/ajp.20854

Mirville, M. O., Ridley, A. R., Samedi, J. P. M., Vecellio, V., Ndagijimana, F., Stoinski, T. S., & Grueter, C. C. (2018). Factors influencing individual participation during intergroup interactions in mountain gorillas. Animal Behaviour, 144, 75–86. https://doi.org/10.1016/j.anbehav.2018.08.003

Molesti, S., & Majolo, B. (2017). Evidence of direct reciprocity, but not of indirect and generalized reciprocity, in the grooming exchanges of wild Barbary macaques (Macaca sylvanus). American Journal of Primatology, 79(9), 1–12. https://doi.org/10.1002/ajp.22679

Molesti, S., & Majolo, B. (2016). Cooperation in wild Barbary macaques: factors affecting free partner choice. Animal Cognition, 19(1), 133–146. https://doi.org/10.1007/s10071-015-0919-4

Molesti, S., & Majolo, B. (2015). No short-term contingency between grooming and food tolerance in barbary macaques (Macaca sylvanus). Ethology, 121(4), 372–382. https://doi.org/10.1111/eth.12346

Molesti, S., Meguerditchian, A., & Bourjade, M. (2020). Gestural communication in olive baboons (Papio anubis): repertoire and intentionality. Animal Cognition, 23(1), 19–40. https://doi.org/10.1007/s10071-019-01312-y

Mondragón-Ceballos, R., Chiappa, P., Mayagoitia, L., & Lee, P. (2010). Sex differences in learning the allocation of social grooming in infant stumptailed macaques. Behaviour, 147(9), 1073–1099. https://doi.org/10.1163/000579510X505436

Monteza-Moreno, C. M., Dogandžić, T., McLean, K. A., Castillo-Caballero, P. L., Mijango-Ramos, Z., Del Rosario-Vargas, E., Crofoot, M. C., & Barrett, B. J. (2020). White-Faced Capuchin, Cebus capucinus imitator, Hammerstone and Anvil Tool Use in Riparian Habitats on Coiba Island, Panama. International Journal of Primatology, 41(3), 429–433. https://doi.org/10.1007/s10764-020-00156-5

Morgan, G., Kornell, N., Kornblum, T., & Terrace, H. S. (2014). Retrospective and prospective metacognitive judgments in rhesus macaques (Macaca mulatta). Animal Cognition, 17(2), 249–257. https://doi.org/10.1007/s10071-013-0657-4

Morimoto, Y., & Fujita, K. (2012). Capuchin monkeys (Cebus apella) use conspecifics’ emotional expressions to evaluate emotional valence of objects. Animal Cognition, 15(3), 341–347. https://doi.org/10.1007/s10071-011-0458-6

Morimoto, Y., & Fujita, K. (2011). Capuchin monkeys (Cebus apella) modify their own behaviors according to a conspecific’s emotional expressions. Primates, 52(3), 279–286. https://doi.org/10.1007/s10329-011-0249-3

Morimura, N., & Mori, Y. (2010). Effects of early rearing conditions on problem-solving skill in captive male chimpanzees (Pan troglodytes). American Journal of Primatology, 72(7), 626–633. https://doi.org/10.1002/ajp.20819

Morton, F. B., Brosnan, S. F., Prétôt, L., Buchanan-Smith, H. M., O’Sullivan, E., Stocker, M., D’Mello, D., & Wilson, V. A. D. (2016). Using photographs to study animal social cognition and behaviour: Do capuchins’ responses to photos reflect reality? Behavioural Processes, 124, 38–46. https://doi.org/10.1016/j.beproc.2015.10.005

Moscovice, L. R., & Snowdon, C. T. (2006). The role of social context and individual experience in novel task acquisition in cottontop tamarins, Saguinus oedipus. Animal Behaviour, 71(4), 933–943. https://doi.org/10.1016/j.anbehav.2005.09.007

Mugisha, S., Zuberbühler, K., & Hobaiter, C. (2016). First observation of Dorylus ant feeding in Budongo chimpanzees supports absence of stick-tool culture. Primates, 57(3), 389–394. https://doi.org/10.1007/s10329-016-0533-3

Mulcahy, N. J. (2016). Orangutans (Pongo abelii) seek information about tool functionality in a metacognition tubes task. Journal of Comparative Psychology, 130(4), 391–399. https://doi.org/10.1037/com0000046

Mulcahy, N. J., & Call, J. (2006). How great apes perform on a modified trap-tube task. Animal Cognition, 9(3), 193–199. https://doi.org/10.1007/s10071-006-0019-6

Mulcahy, N. J., & Schubiger, M. N. (2014). Can orangutans (Pongo abelii) infer tool functionality? Animal Cognition, 17(3), 657–669. https://doi.org/10.1007/s10071-013-0697-9

Mulcahy, N. J., Schubiger, M. N., & Suddendorf, T. (2013). Orangutans (Pongo pygmaeus and Pongo abelii) understand connectivity in the skewered grape tool task. Journal of Comparative Psychology, 127(1), 109–113. https://doi.org/10.1037/a0028621

Mulcahy, N. J., & Suddendorf, T. (2011). An Obedient Orangutan (Pongo abelii) Performs Perfectly in Peripheral Object-Choice Tasks but Fails the Standard Centrally Presented Versions. Journal of Comparative Psychology, 125(1), 112–115. https://doi.org/10.1037/a0020905

Mulholland, M. M., Mahovetz, L. M., Mareno, M. C., Reamer, L. A., Schapiro, S. J., & Hopkins, W. D. (2020). Differences in the mutual eye gaze of bonobos (Pan paniscus) and chimpanzees (Pan troglodytes). Journal of Comparative Psychology, 134(3), 318–322. https://doi.org/10.1037/com0000247

Murai, C., Tanaka, M., & Sakagami, M. (2011). Physical Intuitions About Support Relations in Monkeys (Macaca fuscata) and Apes (Pan troglodytes). Journal of Comparative Psychology, 125(2), 216–226. https://doi.org/10.1037/a0022099

Murray, L. E. (2020). Video-mediated behavior in gorillas (Gorilla gorilla gorilla): A stage in the development of self-recognition in a juvenile male? Journal of Comparative Psychology, 134(3), 293–302. https://doi.org/10.1037/com0000216

Murray, L., Schaffner, C. M., Aureli, F., & Amici, F. (2020). There is no other monkey in the mirror for spider monkeys (Ateles geoffroyi). Journal of Comparative Psychology, 134(3), 323–329. https://doi.org/10.1037/com0000243

Mustafar, F., De Luna, P., & Rainer, G. (2015). Enhanced visual exploration for real objects compared to pictures during free viewing in the macaque monkey. Behavioural Processes, 118, 8–20. https://doi.org/10.1016/j.beproc.2015.05.009

Mustoe, A. C., Harnisch, A. M., Hochfelder, B., Cavanaugh, J., & French, J. A. (2016). Inequity aversion strategies between marmosets are influenced by partner familiarity and sex but not by oxytocin. Animal Behaviour, 114, 69–79. https://doi.org/10.1016/j.anbehav.2016.01.025

Nahallage, C. A. D., & Huffman, M. A. (2008). Comparison of stone handling behavior in two macaque species: Implications for the role of phylogeny and environment in primate cultural variation. American Journal of Primatology, 70(12), 1124–1132. https://doi.org/10.1002/ajp.20610

Nahallage, C. A. D., & Huffman, M. A. (2007). Age-specific functions of stone handling, a solitary-object play behavior, in Japanese macaques (Macaca fuscata). American Journal of Primatology, 69(3), 267–281. https://doi.org/10.1002/ajp.20348

Nahallage, C. A. D., & Huffman, M. A. (2007). Acquisition and development of stone handling behavior in infant Japanese macaques. Behaviour, 144(10), 1193–1215. https://doi.org/10.1163/156853907781890959

Nakamura, M., & Nishida, T. (2013). Ontogeny of a Social Custom in Wild Chimpanzees: Age Changes in Grooming Hand-Clasp at Mahale. American Journal of Primatology, 75(2), 186–196. https://doi.org/10.1002/ajp.22098

Nakamura, M., & Nishida, T. (2006). Subtle behavioral variation in wild chimpanzees, with special reference to Imanishi’s concept of kaluchua. Primates, 47(1), 35–42. https://doi.org/10.1007/s10329-005-0142-z

Nakata, R., Eifuku, S., & Tamura, R. (2018). Crucial information for efficient face searching by humans and Japanese macaques. Animal Cognition, 21(1), 155–164. https://doi.org/10.1007/s10071-017-1148-9

Nakata, R., Eifuku, S., & Tamura, R. (2014). Effects of tilted orientations and face-like configurations on visual search asymmetry in macaques. Animal Cognition, 17(1), 67–76. https://doi.org/10.1007/s10071-013-0638-7

Nakata, R., & Osada, Y. (2012). The Thatcher illusion in squirrel monkeys (Saimiri sciureus). Animal Cognition, 15(4), 517–523. https://doi.org/10.1007/s10071-012-0479-9

Naqshbandi, M., & Roberts, W. A. (2006). Anticipation of future events in squirrel monkeys (Saimiri sciureus) and rats (Rattus norvegicus): Tests of the Bischof-Kohler hypothesis. Journal of Comparative Psychology, 120(4), 345–357. https://doi.org/10.1037/0735-7036.120.4.345

Negrey, J. D., & Langergraber, K. E. (2020). Corpse-directed play parenting by a sterile adult female chimpanzee. Primates, 61(1), 29–34. https://doi.org/10.1007/s10329-019-00734-z

Neiworth, J. J. (2013). Chasing sounds. Behavioural Processes, 93, 111–115. https://doi.org/10.1016/j.beproc.2012.11.009

Neiworth, J. J., Johnson, E. T., Whillock, K., Greenberg, J., & Brown, V. (2009). Is a Sense of Inequity an Ancestral Primate Trait? Testing Social Inequity in Cotton Top Tamarins (Saguinus oedipus). Journal of Comparative Psychology, 123(1), 10–17. https://doi.org/10.1037/a0012662

Neiworth, J. J., London, J. M., Flynn, M. J., Rupert, D. D., Alldritt, O., & Hyde, C. (2017). Artificial grammar learning in tamarins (Saguinus oedipus) in varying stimulus contexts. Journal of Comparative Psychology, 131(2), 128–138. https://doi.org/10.1037/com0000066

Neiworth, J. J., Whillock, K. M., Kim, S. H., Greenberg, J. R., Jones, K. B., Patel, A. R., Steefel-Moore, D. L., Shaw, A. J., Rupert, D. D., Gauer, J. L., & Kudura, A. G. (2014). Gestalt principle use in college students, children with autism, toddlers (homo sapiens), and cotton top tamarins (saguinus oedipus). Journal of Comparative Psychology, 128(2), 188–198. https://doi.org/10.1037/a0034840

Neldner, K., Collier-Baker, E., & Nielsen, M. (2015). Chimpanzees (Pan troglodytes) and human children (Homo sapiens) know when they are ignorant about the location of food. Animal Cognition, 18(3), 683–699. https://doi.org/10.1007/s10071-015-0836-6

Nettle, D., Cronin, K. A., & Bateson, M. (2013). Responses of chimpanzees to cues of conspecific observation. Animal Behaviour, 86(3), 595–602. https://doi.org/10.1016/j.anbehav.2013.06.015

Neves Filho, H. B., de Carvalho Neto, M. B., Taytelbaum, G. P. T., Malheiros, R. dos S., & Knaus, Y. C. (2016). Effects of different training histories upon manufacturing a tool to solve a problem: insight in capuchin monkeys (Sapajus spp.). Animal Cognition, 19(6), 1151–1164. https://doi.org/10.1007/s10071-016-1022-1

Newton-Fisher, N. E., & Kaburu, S. S. K. (2017). Grooming decisions under structural despotism: the impact of social rank and bystanders among wild male chimpanzees. Animal Behaviour, 128, 153–164. https://doi.org/10.1016/j.anbehav.2017.04.012

Newton-Fisher, N. E., & Lee, P. C. (2011). Grooming reciprocity in wild male chimpanzees. Animal Behaviour, 81(2), 439–446. https://doi.org/10.1016/j.anbehav.2010.11.015

Nishie, H. (2011). Natural history of Camponotus ant-fishing by the M group chimpanzees at the Mahale Mountains National Park, Tanzania. Primates, 52(4), 329–342. https://doi.org/10.1007/s10329-011-0270-6

Noë, R., & Laporte, M. (2014). Socio-spatial cognition in vervet monkeys. Animal Cognition, 17(3), 597–607. https://doi.org/10.1007/s10071-013-0690-3

Normand, E., Ban, S. D., & Boesch, C. (2009). Forest chimpanzees (Pan troglodytes verus) remember the location of numerous fruit trees. Animal Cognition, 12(6), 797–807. https://doi.org/10.1007/s10071-009-0239-7

Normand, E., & Boesch, C. (2009). Sophisticated Euclidean maps in forest chimpanzees. Animal Behaviour, 77(5), 1195–1201. https://doi.org/10.1016/j.anbehav.2009.01.025

Noser, R., & Byrne, R. W. (2015). Wild chacma baboons (Papio ursinus) remember single foraging episodes. Animal Cognition, 18(4), 921–929. https://doi.org/10.1007/s10071-015-0862-4

Noser, R., & Byrne, R. W. (2014). Change point analysis of travel routes reveals novel insights into foraging strategies and cognitive maps of wild baboons. American Journal of Primatology, 76(5), 399–409. https://doi.org/10.1002/ajp.22181

Noser, R., & Byrne, R. W. (2010). How do wild baboons (Papio ursinus) plan their routes? Travel among multiple high-quality food sources with inter-group competition. Animal Cognition, 13(1), 145–155. https://doi.org/10.1007/s10071-009-0254-8

Noser, R., & Byrne, R. W. (2007). Travel routes and planning of visits to out-of-sight resources in wild chacma baboons, Papio ursinus. Animal Behaviour, 73(2), 257–266. https://doi.org/10.1016/j.anbehav.2006.04.012

Noser, R., & Byrne, R. W. (2007). Mental maps in chacma baboons (Papio ursinus): Using inter-group encounters as a natural experiment. Animal Cognition, 10(3), 331–340. https://doi.org/10.1007/s10071-006-0068-x

Nowell, A. A., & Fletcher, A. W. (2007). The development of feeding behaviour in wild western lowland gorillas (Gorilla gorilla gorilla). Behaviour, 145(2), 171–193. https://doi.org/10.1163/156853907783244747

Nowell, A. A., & Fletcher, A. W. (2006). Food transfers in immature wild western lowland gorillas (gorilla gorilla gorilla). Primates, 47(4), 294–299. https://doi.org/10.1007/s10329-006-0181-0

Oettinger, B. C., Crockett, C. M., & Bellanca, R. U. (2007). Communicative contexts of the LEN facial expression of pigtailed macaques (Macaca nemestrina). Primates, 48(4), 293–302. https://doi.org/10.1007/s10329-007-0046-1

Oh, J., Šlipogor, V., & Tecumseh Fitch, W. (2019). Artificial visual stimuli for animal experiments: An experimental evaluation in a prey capture context with common marmosets (Callithrix jacchus). Journal of Comparative Psychology, 133(1), 72–80. https://doi.org/10.1037/com0000129

O’Hara, S. J., & Lee, P. C. (2006). High frequency of postcoital penis cleaning in Budongo chimpanzees. Folia Primatologica, 77(5), 353–358. https://doi.org/10.1159/000093700

Ohashi, G. (2015). Pestle-pounding and nut-cracking by wild chimpanzees at Kpala, Liberia. Primates, 56(2), 113–117. https://doi.org/10.1007/s10329-015-0459-1

O’Sullivan, E. P., Claidière, N., & Caldwell, C. A. (2017). Action-matching biases in monkeys (Sapajus spp.) in a stimulus-response compatibility task: Evaluating experience-dependent malleability. Journal of Comparative Psychology, 131(4), 337–347. https://doi.org/10.1037/com0000081

Osuna-Mascaró, A. J., Ortiz, C., Stolz, C., Musgrave, S., Sanz, C. M., Morgan, D. B., & Fragaszy, D. M. (2021). Dexterity and technique in termite fishing by chimpanzees (Pan troglodytes troglodytes) in the Goualougo Triangle, Republic of Congo. American Journal of Primatology, 83(1). https://doi.org/10.1002/ajp.23215

Osvath, M., & Osvath, H. (2008). Chimpanzee (Pan troglodytes) and orangutan (Pongo abelii) forethought: Self-control and pre-experience in the face of future tool use. Animal Cognition, 11(4), 661–674. https://doi.org/10.1007/s10071-008-0157-0

Otovic, P., Partan, S. R., Bryant, J. B., & Hutchinson, E. (2014). Let’s call a Truce...for now: The silent bared-teeth face expression in Mandrills (Mandrillus sphinx) during baseline and post-conflict conditions. Ethology, 120(11), 1118–1127. https://doi.org/10.1111/eth.12285

Ouattara, K., Lemasson, A., & Zuberbühler, K. (2009). Anti-predator strategies of free-ranging Campbell’s monkeys. Behaviour, 146(12), 1687–1708. https://doi.org/10.1163/000579509X12469533725585

Overduin-De Vries, A. M., Massen, J. J. M., Spruijt, B. M., & Sterck, E. H. M. (2012). Sneaky Monkeys: An Audience Effect of Male Rhesus Macaques (Macaca mulatta) on Sexual Behavior. American Journal of Primatology, 74(3), 217–228. https://doi.org/10.1002/ajp.21988

Overduin-de Vries, A. M., Spruijt, B. M., & Sterck, E. H. M. (2014). Long-tailed macaques (Macaca fascicularis) understand what conspecifics can see in a competitive situation. Animal Cognition, 17(1), 77–84. https://doi.org/10.1007/s10071-013-0639-6

Paglieri, F., Focaroli, V., Bramlett, J., Tierno, V., McIntyre, J. M., Addessi, E., Evans, T. A., & Beran, M. J. (2013). The hybrid delay task: Can capuchin monkeys (Cebus apella) sustain a delay after an initial choice to do so? Behavioural Processes, 94, 45–54. https://doi.org/10.1016/j.beproc.2012.12.002

Painter, M. C., Russell, R. C., & Judge, P. G. (2019). Capuchins (Sapajus apella) and Squirrel Monkeys (Saimiri sciureus) Fail to Attend to the Functional Spatial Relationship Between a Tool and a Reward. Journal of Comparative Psychology, 133(4), 463. https://doi.org/10.1037/com0000179

Pal, A., Kumara, H. N., Mishra, P. S., Velankar, A. D., & Singh, M. (2018). Extractive foraging and tool-aided behaviors in the wild Nicobar long-tailed macaque (Macaca fascicularis umbrosus). Primates, 59(2), 173–183. https://doi.org/10.1007/s10329-017-0635-6

Palacios-Romo, T. M., Castellanos, F., & Ramos-Fernandez, G. (2019). Uncovering the decision rules behind collective foraging in spider monkeys. Animal Behaviour, 149, 121–133. https://doi.org/10.1016/j.anbehav.2019.01.011

Palagi, E., Norscia, I., & Cordoni, G. (2019). Lowland Gorillas (Gorilla gorilla gorilla) Failed to Respond to Others’ Yawn: Experimental and Naturalistic Evidence. Journal of Comparative Psychology, 133(3), 406. https://doi.org/10.1037/com0000175

Pan, J., Kennedy, E. H., Pickering, T., Menzel, C. R., Stone, B. W., & Fragaszy, D. M. (2011). Development of maze navigation by tufted capuchins (Cebus apella). Behavioural Processes, 86(2), 206–215. https://doi.org/10.1016/j.beproc.2010.11.006

Pansini, R., & de Ruiter, J. R. (2011). Observation of tool use and modification for apparent hygiene purposes in a mandrill. Behavioural Processes, 88(1), 53–55. https://doi.org/10.1016/j.beproc.2011.06.003

Parr, L. A., & Heintz, M. (2009). Facial expression recognition in rhesus monkeys, Macaca mulatta. Animal Behaviour, 77(6), 1507–1513. https://doi.org/10.1016/j.anbehav.2009.02.024

Parr, L. A., & Heintz, M. (2008). Discrimination of faces and houses by rhesus monkeys: The role of stimulus expertise and rotation angle. Animal Cognition, 11(3), 467–474. https://doi.org/10.1007/s10071-008-0137-4

Parr, L. A., Heintz, M., Lonsdorf, E., & Wroblewski, E. (2010). Visual kin recognition in nonhuman primates: (Pan troglodytes and Macaca mulatta): Inbreeding avoidance or male distinctiveness? Journal of Comparative Psychology, 124(4), 343–350. https://doi.org/10.1037/a0020545

Parr, L. A., Heintz, M., & Pradhan, G. (2008). Rhesus Monkeys (Macaca mulatta) Lack Expertise in Face Processing. Journal of Comparative Psychology, 122(4), 390–402. https://doi.org/10.1037/0735-7036.122.4.390

Parrish, A. E., Afrifa, E., & Beran, M. J. (2018). Exploring decoy effects on computerized task preferences in rhesus monkeys (Macaca mulatta). Animal Behavior and Cognition, 5(2), 235–253. https://doi.org/10.26451/abc.05.02.06.2018

Parrish, A. E., & Beran, M. J. (2014). When less is more: Like humans, chimpanzees (Pan troglodytes) misperceive food amounts based on plate size. Animal Cognition, 17(2), 427–434. https://doi.org/10.1007/s10071-013-0674-3

Parrish, A. E., & Beran, M. J. (2014). Chimpanzees sometimes see fuller as better: Judgments of food quantities based on container size and fullness. Behavioural Processes, 103, 184–191. https://doi.org/10.1016/j.beproc.2013.12.011

Parrish, A. E., Beran, M. J., & Agrillo, C. (2019). Linear numerosity illusions in capuchin monkeys (Sapajus apella), rhesus macaques (Macaca mulatta), and humans (Homo sapiens). Animal Cognition, 22(5), 883–895. https://doi.org/10.1007/s10071-019-01288-9

Parrish, A. E., Brosnan, S. F., Beran, B. J., & Würsig, M. J. (2014). Differential Responding by Rhesus Monkeys (Macaca mulatta) and Humans (Homo sapiens) to Variable Outcomes in the Assurance Game. Animal Behavior and Cognition, 1(3), 215. https://doi.org/10.12966/abc.08.01.2014

Parrish, A. E., Evans, T. A., & Beran, M. J. (2015). Defining value through quantity and quality-Chimpanzees (Pan troglodytes) undervalue food quantities when items are broken. Behavioural Processes, 111, 118–126. https://doi.org/10.1016/j.beproc.2014.11.004

Parrish, A. E., French, K. A., Guild, A. S., Creamer, C. L., Rossettie, M. S., & Beran, M. J. (2020). The density bias: Capuchin monkeys (Sapajus apella) prefer densely arranged items in a food-choice task. Journal of Comparative Psychology, 134(2), 232–240. https://doi.org/10.1037/com0000213

Parrish, A. E., James, B. T., & Beran, M. J. (2017). Exploring whether nonhuman primates show a bias to overestimate dense quantities. Journal of Comparative Psychology, 131(1), 59–68. https://doi.org/10.1037/com0000058

Parrish, A. E., James, B. T., Rossettie, M. S., Smith, T. R., Otalora-Garcia, A., & Beran, M. J. (2018). Investigating the depletion effect: Self-control does not waiver in capuchin monkeys. Animal Behavior and Cognition, 5(1), 118–138. https://doi.org/10.26451/abc.05.01.09.2018

Parrish, A. E., Otalora-Garcia, A., & Beran, M. J. (2017). Dealing with interference: Chimpanzees respond to conflicting cues in a food-choice memory task. Journal of Experimental Psychology: Animal Learning and Cognition, 43(4), 366–376. https://doi.org/10.1037/xan0000151

Parrish, A. E., Perdue, B. M., Evans, T. A., & Beran, M. J. (2013). Chimpanzees (Pan troglodytes) transfer tokens repeatedly with a partner to accumulate rewards in a self-control task. Animal Cognition, 16(4), 627–636. https://doi.org/10.1007/s10071-013-0599-x

Parrish, A. E., Perdue, B. M., Stromberg, E. E., Bania, A. E., Evans, T. A., & Beran, M. J. (2014). Delay of gratification by orangutans (Pongo pygmaeus) in the accumulation task. Journal of Comparative Psychology, 128(2), 209–214. https://doi.org/10.1037/a0035660

Parrish, A., Brosnan, S., & Beran, M. (2015). Capuchin Monkeys Alternate Play and Reward in a Dual Computerized Task. Animal Behavior and Cognition, 2(4), 334–347. https://doi.org/10.12966/abc.11.03.2015

Parron, C., Call, J., & Fagot, J. (2008). Behavioural responses to photographs by pictorially naïve baboons (Papio anubis), gorillas (Gorilla gorilla) and chimpanzees (Pan troglodytes). Behavioural Processes, 78(3), 351–357. https://doi.org/10.1016/j.beproc.2008.01.019

Parron, C., Deruelle, C., & Fagot, J. (2007). Processing of Biological Motion Point-Light Displays by Baboons (Papio papio). Journal of Experimental Psychology: Animal Behavior Processes, 33(4), 381–391. https://doi.org/10.1037/0097-7403.33.4.381

Parron, C., & Fagot, J. (2008). Baboons (Papio papio) spontaneously process the first-order but not second-order configural properties of faces. American Journal of Primatology, 70(5), 415–422. https://doi.org/10.1002/ajp.20503

Parron, C., & Meguerditchian, A. (2016). Gaze following in baboons (Papio anubis): juveniles adjust their gaze and body position to human’s head redirections. American Journal of Primatology, 78(12), 1265–1271. https://doi.org/10.1002/ajp.22580

Parron, C., & Washburn, D. (2010). Contrasting the Edge- and Surface-Based Theories of Object Recognition: Behavioral Evidence From Macaques (Macaca mulatta). Journal of Experimental Psychology: Animal Behavior Processes, 36(1), 148–157. https://doi.org/10.1037/a0015629

Pascual-Garrido, A., Buba, U., Nodza, G., & Sommer, V. (2012). Obtaining raw material: Plants as tool sources for Nigerian Chimpanzees. Folia Primatologica, 83(1), 24–44. https://doi.org/10.1159/000338898

Pascual-Garrido, A., Umaru, B., Allon, O., & Sommer, V. (2013). Apes finding ants: Predator-prey dynamics in a chimpanzee habitat in Nigeria. American Journal of Primatology, 75(12), 1231–1244. https://doi.org/10.1002/ajp.22187

Pastor-Bernier, A., Volkmann, K., Stasiak, A., Grabenhorst, F., & Schultz, W. (2020). Experimentally revealed stochastic preferences for multicomponent choice options. Journal of Experimental Psychology: Animal Learning and Cognition, 46(4), 367–384. https://doi.org/10.1037/xan0000269

Paukner, A., Anderson, J. R., Fogassi, L., & Ferrari, P. F. (2007). Do facial gestures, visibility or speed of movement influence gaze following responses in pigtail macaques? Primates, 48(3), 241–244. https://doi.org/10.1007/s10329-006-0024-z

Paukner, A., Anderson, J. R., & Fujita, K. (2006). Redundant food searches by capuchin monkeys (Cebus apella): A failure of metacognition? Animal Cognition, 9(2), 110–117. https://doi.org/10.1007/s10071-005-0007-2

Paukner, A., Huntsberry, M. E., & Suomi, S. J. (2009). Tufted Capuchin Monkeys (Cebus apella) Spontaneously Use Visual but Not Acoustic Information to Find Hidden Food Items. Journal of Comparative Psychology, 123(1), 26–33. https://doi.org/10.1037/a0013128

Paukner, A., Wooddell, L. J., Lefevre, C. E., Lonsdorf, E., & Lonsdorf, E. (2017). Do capuchin monkeys (Sapajus apella) prefer symmetrical face shapes? Journal of Comparative Psychology, 131(1), 73–77. https://doi.org/10.1037/com0000052

Paxton Gazes, R., Chee, N. W., & Hampton, R. R. (2018). Monkeys choose, but do not learn, through exclusion. Animal Behavior and Cognition, 5(1), 9–18. https://doi.org/10.26451/abc.05.01.02.2018

Paxton, R., Basile, B. M., Adachi, I., Suzuki, W. A., Wilson, M. E., & Hampton, R. R. (2010). Rhesus monkeys (Macaca mulatta) rapidly learn to select dominant individuals in videos of artificial social interactions between unfamiliar conspecifics. Journal of Comparative Psychology, 124(4), 395–401. https://doi.org/10.1037/a0019751

Pelé, M., Dufour, V., Micheletta, J., & Thierry, B. (2010). Long-tailed macaques display unexpected waiting abilities in exchange tasks. Animal Cognition, 13(2), 263–271. https://doi.org/10.1007/s10071-009-0264-6

Pelé, M., Dufour, V., Thierry, B., & Call, J. (2009). Token Transfers Among Great Apes (Gorilla gorilla, Pongo pygmaeus, Pan paniscus, and Pan troglodytes): Species Differences, Gestural Requests, and Reciprocal Exchange. Journal of Comparative Psychology, 123(4), 375–384. https://doi.org/10.1037/a0017253

Pelé, M., Micheletta, J., Uhlrich, P., Thierry, B., & Dufour, V. (2011). Delay Maintenance in Tonkean Macaques (Macaca tonkeana) and Brown Capuchin Monkeys (Cebus apella). International Journal of Primatology, 32(1), 149–166. https://doi.org/10.1007/s10764-010-9446-y

Pelletier, A. N., Kaufmann, T., Mohak, S., Milan, R., Nahallage, C. A. D., Huffman, M. A., Gunst, N., Rompis, A., Wandia, I. N., Arta Putra, I. G. A., Pellis, S. M., & Leca, J.-B. (2017). Behavior Systems Approach to Object Play: Stone Handling Repertoire as a Measure of Propensity for Complex Foraging and Percussive Tool Use in the Genus Macaca. Animal Behavior and Cognition, 4(4), 455–473. https://doi.org/10.26451/abc.04.04.05.201

Perdue, B. (2017). Mechanisms underlying cognitive bias in nonhuman primates. Animal Behavior and Cognition, 4(1), 105–118. https://doi.org/10.12966/abc.08.02.2017

Perdue, B. M., Bramlett, J. L., Evans, T. A., & Beran, M. J. (2015). Waiting for what comes later: capuchin monkeys show self-control even for nonvisible delayed rewards. Animal Cognition, 18(5), 1105–1112. https://doi.org/10.1007/s10071-015-0878-9

Perdue, B. M., & Brown, E. R. (2018). Irrational choice behavior in human and nonhuman primates. Animal Cognition, 21(2), 227–234. https://doi.org/10.1007/s10071-017-1156-9

Perdue, B. M., Evans, T. A., Williamson, R. A., Gonsiorowski, A., & Beran, M. J. (2014). Prospective memory in children and chimpanzees. Animal Cognition, 17(2), 287–295. https://doi.org/10.1007/s10071-013-0661-8

Perlman, M., & Clark, N. (2015). Learned vocal and breathing behavior in an enculturated gorilla. Animal Cognition, 18(5), 1165–1179. https://doi.org/10.1007/s10071-015-0889-6

Persson, T., Sauciuc, G. A., & Madsen, E. A. (2018). Spontaneous cross-species imitation in interactions between chimpanzees and zoo visitors. Primates, 59(1), 19–29. https://doi.org/10.1007/s10329-017-0624-9

Petit, O., Dufour, V., Herrenschmidt, M., De Marco, A., Sterck, E. H. M., & Call, J. (2015). Inferences about food location in three cercopithecine species: an insight into the socioecological cognition of primates. Animal Cognition, 18(4), 821–830. https://doi.org/10.1007/s10071-015-0848-2

Pfefferle, D., Heistermann, M., Hodges, J. K., & Fischer, J. (2008). Male Barbary macaques eavesdrop on mating outcome: a playback study. Animal Behaviour, 75(6), 1885–1891. https://doi.org/10.1016/j.anbehav.2007.12.003

Pika, S., & Zuberbühler, K. (2008). Social games between bonobos and humans: Evidence for shared intentionality? American Journal of Primatology, 70(3), 207–210. https://doi.org/10.1002/ajp.20469

Pladevall, J., Mendes, N., Riba, D., Llorente, M., & Amici, F. (2020). No evidence of what-where-when memory in great apes (Pan troglodytes, Pan paniscus, Pongo abelii, and Gorilla gorilla). Journal of Comparative Psychology, 134(2), 252–261. https://doi.org/10.1037/com0000215

Ploog, B. O. (2011). Selective attention to visual compound stimuli in squirrel monkeys (Saimiri sciureus). Behavioural Processes, 87(1), 115–124. https://doi.org/10.1016/j.beproc.2010.12.015

Pokorny, J. J., & de Waal, F. B. M. (2009). Face Recognition in Capuchin Monkeys (Cebus apella). Journal of Comparative Psychology, 123(2), 151–160. https://doi.org/10.1037/a0014073

Pokorny, J. J., Webb, C. E., & de Waal, F. B. M. (2011). An inversion effect modified by expertise in capuchin monkeys. Animal Cognition, 14(6), 839–846. https://doi.org/10.1007/s10071-011-0417-2

Polizzi di Sorrentino, E., Sabbatini, G., Truppa, V., Bordonali, A., Taffoni, F., Formica, D., Baldassarre, G., Mirolli, M., Guglielmelli, E., & Visalberghi, E. (2014). Exploration and learning in capuchin monkeys (Sapajus spp.): The role of action-outcome contingencies. Animal Cognition, 17(5), 1081–1088. https://doi.org/10.1007/s10071-014-0740-5

Pomerantz, O., Terkel, J., Suomi, S. J., & Paukner, A. (2012). Stereotypic head twirls, but not pacing, are related to a ’pessimistic’-like judgment bias among captive tufted capuchins (Cebus apella). Animal Cognition, 15(4), 689–698. https://doi.org/10.1007/s10071-012-0497-7

Pope, S. M., Fagot, J., Meguerditchian, A., Watzek, J., Lew-Levy, S., Autrey, M. M., & Hopkins, W. D. (2020). Optional-switch cognitive flexibility in primates: Chimpanzees’ (Pan troglodytes) intermediate susceptibility to cognitive set. Journal of Comparative Psychology, 134(1), 98–109. https://doi.org/10.1037/com0000194

Pope, S. M., Meguerditchian, A., Hopkins, W. D., & Fagot, J. (2015). Baboons (Papio papio), but not humans, break cognitive set in a visuomotor task. Animal Cognition, 18(6), 1339–1346. https://doi.org/10.1007/s10071-015-0904-y

Posada, S., & Colell, M. (2007). Another gorilla (Gorilla gorilla gorilla) recognizes himself in a mirror. American Journal of Primatology, 69(5), 576–583. https://doi.org/10.1002/ajp.20355

Poss, S. R., Kuhar, C., Stoinski, T. S., & Hopkins, W. D. (2006). Differential use of attentional and visual communicative signaling by orangutans (Pongo pygmaeus) and gorillas (Gorilla gorilla) in response to the attentional status of a human. American Journal of Primatology, 68(10), 978–992. https://doi.org/10.1002/ajp.20304

Potì, P., & Saporiti, M. (2010). A Capuchin Monkey (Cebus apella) uses video to find food. Folia Primatologica, 81(1), 16–30. https://doi.org/10.1159/000277636

Pouydebat, E., Gorce, P., Bels, V., & Coppens, Y. (2006). Substrate optimization in nut cracking by capuchin monkeys (Cebus apella). American Journal of Primatology, 68(10), 1017–1024. https://doi.org/10.1002/ajp.20291

Povinelli, D. J., & Henley, T. (2020). More Rope Tricks Reveal Why More Task Variants Will Never Lead to Strong Inferences About Higher-Order Causal Reasoning in Chimpanzees. Animal Behavior and Cognition, 7(3), 392–418. https://doi.org/10.26451/abc.07.03.08.2020

Presotto, A., Verderane, M. P., Biondi, L., Mendonça-Furtado, O., Spagnoletti, N., Madden, M., & Izar, P. (2018). Intersection as key locations for bearded capuchin monkeys (Sapajus libidinosus) traveling within a route network. Animal Cognition, 21(3), 393–405. https://doi.org/10.1007/s10071-018-1176-0

Presotto, A., & Izar, P. (2010). Spatial reference of black capuchin monkeys in Brazilian Atlantic Forest: Egocentric or allocentric? Animal Behaviour, 80(1), 125–132. https://doi.org/10.1016/j.anbehav.2010.04.009

Prétôt, L., & Brosnan, S. F. (2019). Capuchin monkeys (Cebus [Sapajus] apella) show planning in a manual maze task. Journal of Comparative Psychology, 133(1), 81–91. https://doi.org/10.1037/com0000133

Prétôt, L., Bshary, R., & Brosnan, S. F. (2016). Erratum to “Factors influencing the different performance of fish and primates on a dichotomous choice task” (Animal Behaviour (2016) 119 (189–199)). Animal Behaviour, 121, 1. https://doi.org/10.1016/j.anbehav.2016.08.011

Prétôt, L., Bshary, R., & Brosnan, S. F. (2016). Comparing species decisions in a dichotomous choice task: adjusting task parameters improves performance in monkeys. Animal Cognition, 19(4), 819–834. https://doi.org/10.1007/s10071-016-0981-6

Prétôt, L., Mickelberg, J., Carrigan, J., Stoinski, T., Bshary, R., & Brosnan, S. F. (2021). Comparative performance of orangutans (Pongo spp.), gorillas (Gorilla gorilla gorilla), and drills (Mandrillus leucophaeus), in an ephemeral foraging task. American Journal of Primatology, 83(1). https://doi.org/10.1002/ajp.23212

Prétôt, L., Weiss, D. J., & Brosnan, S. F. (2018). Do nonhuman primates ascribe goals to the choices of conspecifics? Animal Behavior and Cognition, 5(1), 41–54. https://doi.org/10.26451/abc.05.01.04.2018

Price, E., & Caldwell, C. A. (2007). Artificially generated cultural variation between two groups of captive monkeys, Colobus guereza kikuyuensis. Behavioural Processes, 74(1), 13–20. https://doi.org/10.1016/j.beproc.2006.09.003

Price, T., Arnold, K., Zuberbühler, K., & Semple, S. (2009). Pyow but not hack calls of the male putty-nosed monkey (Cercopithcus nictitans) convey information about caller identity. Behaviour, 146(7), 871–888. https://doi.org/10.1163/156853908X396610

Prieur, J., & Pika, S. (2020). Gorillas’ (Gorilla g. gorilla) knowledge of conspecifics’ affordances: intraspecific social tool use for food acquisition. Primates, 61(4), 583–591. https://doi.org/10.1007/s10329-020-00805-6

Proctor, D., Williamson, R. A., Latzman, R. D., de Waal, F. B. M., & Brosnan, S. F. (2014). Gambling primates: Reactions to a modified Iowa Gambling Task in humans, chimpanzees and capuchin monkeys. Animal Cognition, 17(4), 983–995. https://doi.org/10.1007/s10071-014-0730-7

Qi, X. G., Grueter, C. C., Fang, G., Huang, P. Z., Zhang, J., Duan, Y. M., Huang, Z. P., Garber, P. A., & Li, B. G. (2020). Multilevel societies facilitate infanticide avoidance through increased extrapair matings. Animal Behaviour, 161, 127–137. https://doi.org/10.1016/j.anbehav.2019.12.014

Ramos-Fernández, G., Pinacho-Guendulain, B., Miranda-Pérez, A., & Boyer, D. (2011). No Evidence of Coordination Between Different Subgroups in the Fission-Fusion Society of Spider Monkeys (Ateles geoffroyi). International Journal of Primatology, 32(6), 1367–1382. https://doi.org/10.1007/s10764-011-9544-5

Rawlings, B., Davila-Ross, M., & Boysen, S. T. (2014). Semi-wild chimpanzees open hard-shelled fruits differently across communities. Animal Cognition, 17(4), 891–899. https://doi.org/10.1007/s10071-013-0722-z

Rayburn-Reeves, R. M., James, B. T., & Beran, M. J. (2017). Within-session reversal learning in rhesus macaques (Macaca mulatta). Animal Cognition, 20(5), 975–983. https://doi.org/10.1007/s10071-017-1117-3

Reddy, R. B., MacLean, E. L., Sandel, A. A., & Hare, B. (2015). Social inhibitory control in five lemur species. Primates, 56(3), 241–252. https://doi.org/10.1007/s10329-015-0467-1

Renner, E., Abramo, A. M., Karen Hambright, M., & Phillips, K. A. (2017). Insightful problem solving and emulation in brown capuchin monkeys. Animal Cognition, 20(3), 531–536. https://doi.org/10.1007/s10071-017-1080-z

Renner, E., Price, E. E., & Subiaul, F. (2016). Sequential recall of meaningful and arbitrary sequences by orangutans and human children: Does content matter? Animal Cognition, 19(1), 39–52. https://doi.org/10.1007/s10071-015-0911-z

Reran, M. J., Smith, J. D., Redford, J. S., & Washburn, D. A. (2006). Rhesus macaques (Macaca mulatto) monitor uncertainty during numerosity judgments. Journal of Experimental Psychology: Animal Behavior Processes, 32(2), 111–119. https://doi.org/10.1037/0097-7403.32.2.111

Resende, B. D., Nagy-Reis, M. B., Lacerda, F. N., Pagnotta, M., & Savalli, C. (2014). Tufted capuchin monkeys (Sapajus sp) learning how to crack nuts: Does variability decline throughout development? Behavioural Processes, 109(Part A), 89–94. https://doi.org/10.1016/j.beproc.2014.09.002

Reynolds, G., & Reed, P. (2018). The effect of stimulus duration on over-selectivity: Evidence for the role of within-compound associations. Journal of Experimental Psychology: Animal Learning and Cognition, 44(3), 293–308. https://doi.org/10.1037/xan0000175

Roberts, A. I., & Roberts, S. G. B. (2019). Persistence in gestural communication predicts sociality in wild chimpanzees. Animal Cognition, 22(5), 605–618. https://doi.org/10.1007/s10071-018-1219-6

Roberts, A. I., Roberts, S. G. B., & Vick, S. J. (2014). The repertoire and intentionality of gestural communication in wild chimpanzees. Animal Cognition, 17(2), 317–336. https://doi.org/10.1007/s10071-013-0664-5

Roberts, A. I., Vick, S. J., & Buchanan-Smith, H. M. (2013). Communicative intentions in wild chimpanzees: Persistence and elaboration in gestural signalling. Animal Cognition, 16(2), 187–196. https://doi.org/10.1007/s10071-012-0563-1

Roberts, A. I., Vick, S. J., & Buchanan-Smith, H. M. (2012). Usage and comprehension of manual gestures in wild chimpanzees. Animal Behaviour, 84(2), 459–470. https://doi.org/10.1016/j.anbehav.2012.05.022

Roberts, S. G. B., & Roberts, A. I. (2019). Visual attention, indicative gestures, and calls accompanying gestural communication are associated with sociality in wild chimpanzees (Pan troglodytes schweinfurthii). Journal of Comparative Psychology, 133(1), 56–71. https://doi.org/10.1037/com0000128

Roma, P. G., Silberberg, A., Huntsberry, M. E., Christensen, C. J., Ruggiero, A. M., & Suomi, S. J. (2007). Mark tests for mirror self-recognition in capuchin monkeys (Cebus apella) trained to touch marks. American Journal of Primatology, 69(9), 989–1000. https://doi.org/10.1002/ajp.20404

Roma, P. G., Silberberg, A., Ruggiero, A. M., & Suomi, S. J. (2006). Capuchin monkeys, inequity aversion, and the frustration effect. Journal of Comparative Psychology, 120(1), 67–73. https://doi.org/10.1037/0735-7036.120.1.67

Romero, T., & de Waal, F. B. M. (2010). Chimpanzee (Pan troglodytes) consolation: Third-party identity as a window on possible function. Journal of Comparative Psychology, 124(3), 278–286. https://doi.org/10.1037/a0019144

Rooijakkers, E. F., Kaminski, J., & Call, J. (2009). Comparing dogs and great apes in their ability to visually track object transpositions. Animal Cognition, 12(6), 789–796. https://doi.org/10.1007/s10071-009-0238-8

Rosati, A. G. (2015). Context influences spatial frames of reference in bonobos (Pan paniscus). Behaviour, 152(3–4), 375–406. https://doi.org/10.1163/1568539X-00003189

Rosati, A. G., & Hare, B. (2012). Decision making across social contexts: Competition increases preferences for risk in chimpanzees and bonobos. Animal Behaviour, 84(4), 869–879. https://doi.org/10.1016/j.anbehav.2012.07.010

Rosati, A. G., Rodriguez, K., & Hare, B. (2014). The ecology of spatial memory in four lemur species. Animal Cognition, 17(4), 947–961. https://doi.org/10.1007/s10071-014-0727-2

Rosati, A. G., & Santos, L. R. (2017). Tolerant Barbary macaques maintain juvenile levels of social attention in old age, but despotic rhesus macaques do not. Animal Behaviour, 130, 199–207. https://doi.org/10.1016/j.anbehav.2017.06.019

Rosati, A. G., Stevens, J. R., & Hauser, M. D. (2006). The effect of handling time on temporal discounting in two New World primates. Animal Behaviour, 71(6), 1379–1387. https://doi.org/10.1016/j.anbehav.2005.11.012

Ross, S. R., Milstein, M. S., Calcutt, S. E., & Lonsdorf, E. V. (2010). Preliminary assessment of methods used to demonstrate nut-cracking behavior to five captive chimpanzees (Pan troglodytes). Folia Primatologica, 81(4), 224–232. https://doi.org/10.1159/000322118

Rowe, A. K., Li, J. H., Sun, L., Sheeran, L. K., Wagner, R. S., Xia, D. P., Uhey, D. A., & Chen, R. (2018). Collective decision making in Tibetan macaques: how followers affect the rules and speed of group movement. Animal Behaviour, 146, 51–61. https://doi.org/10.1016/j.anbehav.2018.10.002

Ruiz, A., Gómez, J. C., Roeder, J. J., & Byrne, R. W. (2009). Gaze following and gaze priming in lemurs. Animal Cognition, 12(3), 427–434. https://doi.org/10.1007/s10071-008-0202-z

Russ, B. E., Comins, J. A., Smith, R., & Hauser, M. D. (2010). Recognizing and respecting claims over resources in free-ranging rhesus monkeys, Macaca mulatta. Animal Behaviour, 80(3), 563–569. https://doi.org/10.1016/j.anbehav.2010.06.026

Russell, Y. I., Call, J., & Dunbar, R. I. M. (2008). Image scoring in great apes. Behavioural Processes, 78(1), 108–111. https://doi.org/10.1016/j.beproc.2007.10.009

Russon, A. E., Handayani, D. P., Kuncoro, P., & Ferisa, A. (2007). Orangutan leaf-carrying for nest-building: Toward unraveling cultural processes. Animal Cognition, 10(2), 189–202. https://doi.org/10.1007/s10071-006-0058-z

Russon, A. E., Kuncoro, P., Ferisa, A., & Handayani, D. P. (2010). How Orangutans (Pongo pygmaeus) Innovate for Water. Journal of Comparative Psychology, 124(1), 14–28. https://doi.org/10.1037/a0017929

Sabbatini, G., Manrique, H. M., Trapanese, C., De Bortoli Vizioli, A., Call, J., & Visalberghi, E. (2014). Sequential use of rigid and pliable tools in tufted capuchin monkeys (Sapajus spp.). Animal Behaviour, 87, 213–220. https://doi.org/10.1016/j.anbehav.2013.10.033

Sabbatini, G., Truppa, V., Hribar, A., Gambetta, B., Call, J., & Visalberghi, E. (2012). Understanding the functional properties of tools: Chimpanzees (Pan troglodytes) and capuchin monkeys (Cebus apella) attend to tool features differently. Animal Cognition, 15(4), 577–590. https://doi.org/10.1007/s10071-012-0486-x

Sabbatini, G., & Visalberghi, E. (2008). Inferences About the Location of Food in Capuchin Monkeys (Cebus apella) in Two Sensory Modalities. Journal of Comparative Psychology, 122(2), 156–166. https://doi.org/10.1037/0735-7036.122.2.156

Salmi, R., & Muñoz, M. (2020). The context of chest beating and hand clapping in wild western gorillas (Gorilla gorilla gorilla). Primates, 61(2), 225–235. https://doi.org/10.1007/s10329-019-00782-5

Salmi, R., Presotto, A., Scarry, C. J., Hawman, P., & Doran-Sheehy, D. M. (2020). Spatial cognition in western gorillas (Gorilla gorilla): an analysis of distance, linearity, and speed of travel routes. Animal Cognition, 23(3), 545–557. https://doi.org/10.1007/s10071-020-01358-3

Samson, D. R., Vining, A., & Nunn, C. L. (2019). Sleep influences cognitive performance in lemurs. Animal Cognition, 22(5), 697–706. https://doi.org/10.1007/s10071-019-01266-1

Samuni, L., Mundry, R., Terkel, J., Zuberbühler, K., & Hobaiter, C. (2014). Socially learned habituation to human observers in wild chimpanzees. Animal Cognition, 17(4), 997–1005. https://doi.org/10.1007/s10071-014-0731-6

Sánchez-Amaro, A., Altınok, N., Heintz, C., & Call, J. (2019). Disentangling Great Apes’ Decoy-Effect Bias in a Food Choice Task. Animal Behavior and Cognition, 6(3), 213–222. https://doi.org/10.26451/abc.06.03.05.2019

Sánchez-Amaro, A., Duguid, S., Call, J., & Tomasello, M. (2016). Chimpanzees coordinate in a snowdrift game. Animal Behaviour, 116, 61–74. https://doi.org/10.1016/j.anbehav.2016.03.030

Sánchez-Amaro, A., Peretó, M., & Call, J. (2016). Differences in between-reinforcer value modulate the selective-value effect in great apes (Pan troglodytes, P. paniscus, Gorilla gorilla, Pongo abelii). Journal of Comparative Psychology, 130(1), 1–12. https://doi.org/10.1037/com0000014

Sánchez-Amaro, A., Tan, J., Kaufhold, S. P., & Rossano, F. (2020). Gibbons exploit information about what a competitor can see. Animal Cognition, 23(2), 289–299. https://doi.org/10.1007/s10071-019-01333-7

Sandel, A. A., MacLean, E. L., & Hare, B. (2011). Evidence from four lemur species that ringtailed lemur social cognition converges with that of haplorhine primates. Animal Behaviour, 81(5), 925–931. https://doi.org/10.1016/j.anbehav.2011.01.020

Santaca, M., Regaiolli, B., Miletto Petrazzini, M. E., Spiezio, C., & Agrillo, C. (2017). Preliminary study to investigate the Delboeuf illusion in ring-tailed lemurs (Lemur catta): Methodological Challenges. Animal Behavior and Cognition, 4(3), 365–377. https://doi.org/10.26451/abc.04.03.13.2017

Santos, L. R., Nissen, A. G., & Ferrugia, J. A. (2006). Rhesus monkeys, Macaca mulatta, know what others can and cannot hear. Animal Behaviour, 71(5), 1175–1181. https://doi.org/10.1016/j.anbehav.2005.10.007

Santos, L. R., Pearson, H. M., Spaepen, G. M., Tsao, F., & Hauser, M. D. (2006). Probing the limits of tool competence: Experiments with two non-tool-using species (Cercopithecus aethiops and Saguinus oedipus). Animal Cognition, 9(2), 94–109. https://doi.org/10.1007/s10071-005-0001-8

Sanz, C. M., Caspar, S., & Morgan, D. B. (2010). Chimpanzees prey on army ants with specialized tool set. American Journal of Primatology, 72(1), 17–24. https://doi.org/10.1002/ajp.20744

Sanz, C. M., & Morgan, D. B. (2009). Flexible and persistent tool-using strategies in honey-gathering by wild chimpanzees. International Journal of Primatology, 30(3), 411–427. https://doi.org/10.1007/s10764-009-9350-5

Sato, Y., Hirata, S., & Kano, F. (2019). Spontaneous attention and psycho-physiological responses to others’ injury in chimpanzees. Animal Cognition, 22(5), 807–823. https://doi.org/10.1007/s10071-019-01276-z

Sauciuc, G. A., Persson, T., Bååth, R., Bobrowicz, K., & Osvath, M. (2016). Affective forecasting in an orangutan: predicting the hedonic outcome of novel juice mixes. Animal Cognition, 19(6), 1081–1092. https://doi.org/10.1007/s10071-016-1015-0

Sawyer, S. C., & Robbins, M. M. (2009). A novel food processing technique by a wild mountain gorilla (Gorilla beringei beringei). Folia Primatologica, 80(2), 83–88. https://doi.org/10.1159/000217680

Scarf, D., & Colombo, M. (2009). Eye Movements During List Execution Reveal No Planning in Monkeys (Macaca fascicularis). Journal of Experimental Psychology: Animal Behavior Processes, 35(4), 587–592. https://doi.org/10.1037/a0014020

Scarf, D., Danly, E., Morgan, G., Colombo, M., & Terrace, H. S. (2011). Sequential planning in rhesus monkeys (Macaca mulatta). Animal Cognition, 14(3), 317–324. https://doi.org/10.1007/s10071-010-0365-2

Schaffner, C. M., Slater, K. Y., & Aureli, F. (2012). Age related variation in male-male relationships in wild spider monkeys (Ateles geoffroyi yucatanensis). Primates, 53(1), 49–56. https://doi.org/10.1007/s10329-011-0271-5

Schamberg, I., Cheney, D. L., Clay, Z., Hohmann, G., & Seyfarth, R. M. (2016). Call combinations, vocal exchanges and interparty movement in wild bonobos. Animal Behaviour, 122, 109–116. https://doi.org/10.1016/j.anbehav.2016.10.003

Schamberg, I., Cheney, D. L., & Seyfarth, R. M. (2017). Bonobos (Pan paniscus) Perform Branch Drag Displays before Long-Distance Travel. International Journal of Primatology, 38(3), 500–512. https://doi.org/10.1007/s10764-017-9957-x

Scheel, M. H., & Edwards, D. (2012). Captive spider monkeys (Ateles geoffroyi) arm-raise to solicit allo-grooming. Behavioural Processes, 89(3), 311–313. https://doi.org/10.1016/j.beproc.2011.12.005

Schel, A. M., Candiotti, A., & Zuberbühler, K. (2010). Predator-deterring alarm call sequences in Guereza colobus monkeys are meaningful to conspecifics. Animal Behaviour, 80(5), 799–808. https://doi.org/10.1016/j.anbehav.2010.07.012

Schel, A. M., Machanda, Z., Townsend, S. W., Zuberbühler, K., & Slocombe, K. E. (2013). Chimpanzee food calls are directed at specific individuals. Animal Behaviour, 86(5), 955–965. https://doi.org/10.1016/j.anbehav.2013.08.013

Schel, A. M., Tranquilli, S., & Zuberbühler, K. (2009). The Alarm Call System of Two Species of Black-and-White Colobus Monkeys (Colobus polykomos and Colobus guereza). Journal of Comparative Psychology, 123(2), 136–150. https://doi.org/10.1037/a0014280

Schel, A. M., & Zuberbühler, K. (2009). Responses to leopards are independent of experience in Guereza colobus monkeys. Behaviour, 146(12), 1709–1737. https://doi.org/10.1163/000579509X12483520922007

Schell, A., Rieck, K., Schell, K., Hammerschmidt, K., & Fischer, J. (2011). Adult but not juvenile Barbary macaques spontaneously recognize group members from pictures. Animal Cognition, 14(4), 503–509. https://doi.org/10.1007/s10071-011-0383-8

Schiel, N., & Huber, L. (2006). Social influences on the development of foraging behavior in free-living common marmosets (Callithrix jacchus). American Journal of Primatology, 68(12), 1150–1160. https://doi.org/10.1002/ajp.20284

Schino, G., & Alessandrini, A. (2018). Mutual grooming functions to prolong grooming received in ringtailed lemurs. Animal Behaviour, 146, 31–35. https://doi.org/10.1016/j.anbehav.2018.09.006

Schino, G., Carducci, P., & Truppa, V. (2020). Attention to social stimuli is modulated by sex and exposure time in tufted capuchin monkeys. Animal Behaviour, 161, 39–47. https://doi.org/10.1016/j.anbehav.2019.12.019

Schino, G., Di Giuseppe, F., & Visalberghi, E. (2009). The time frame of partner choice in the grooming reciprocation of Cebus apella. Ethology, 115(1), 70–76. https://doi.org/10.1111/j.1439-0310.2008.01581.x

Schino, G., Di Sorrentino, E. P., & Tiddi, B. (2007). Grooming and coalitions in Japanese macaques (Macaca fuscata): Partner choice and the time frame reciprocation. Journal of Comparative Psychology, 121(2), 181–188. https://doi.org/10.1037/0735-7036.121.2.181

Schino, G., & Lasio, F. (2019). Mandrills represent their own dominance hierarchy on a cardinal, not ordinal, scale. Animal Cognition, 22(6), 1159–1169. https://doi.org/10.1007/s10071-019-01308-8

Schino, G., Massimei, R., Pinzaglia, M., & Addessi, E. (2016). Grooming, social rank and ‘optimism’ in tufted capuchin monkeys: a study of judgement bias. Animal Behaviour, 119, 11–16. https://doi.org/10.1016/j.anbehav.2016.06.017

Schino, G., & Pellegrini, B. (2011). Grooming and the Expectation of Reciprocation in Mandrills (Mandrillus sphinx). International Journal of Primatology, 32(2), 406–414. https://doi.org/10.1007/s10764-010-9477-4

Schino, G., & Pellegrini, B. (2009). Grooming in mandrills and the time frame of reciprocal partner choice. American Journal of Primatology, 71(10), 884–888. https://doi.org/10.1002/ajp.20719

Schino, G., & Pinzaglia, M. (2018). Age-related changes in the social behavior of tufted capuchin monkeys. American Journal of Primatology, 80(3), 1–7. https://doi.org/10.1002/ajp.22746

Schino, G., & Sciarretta, M. (2016). Patterns of Social Attention in Mandrills, Mandrillus sphinx. International Journal of Primatology, 37(6), 752–761. https://doi.org/10.1007/s10764-016-9936-7

Schino, G., Tiddi, B., & Di Sorrentino, E. P. (2006). Simultaneous classification by rank and kinship in Japanese macaques. Animal Behaviour, 71(5), 1069–1074. https://doi.org/10.1016/j.anbehav.2005.07.019

Schino, G., Tiddi, B., & Sorrentino, E. P. Di. (2007). Agonistic support in juvenile Japanese macaques: Cognitive and functional implications. Ethology, 113(12), 1151–1157. https://doi.org/10.1111/j.1439-0310.2007.01426.x

Schloegl, C., Waldmann, M. R., & Fischer, J. (2013). Understanding of and reasoning about object-object relationships in long-tailed macaques? Animal Cognition, 16(3), 493–507. https://doi.org/10.1007/s10071-012-0591-x

Schmelz, M., Grueneisen, S., & Tomasello, M. (2020). The psychological mechanisms underlying reciprocal prosociality in chimpanzees (Pan troglodytes). Journal of Comparative Psychology, 134(2), 149–157. https://doi.org/10.1037/com0000200

Schmidtke, D., Ammersdörfer, S., Joly, M., & Zimmermann, E. (2018). First comparative approach to touchscreen-based visual object-location paired-associates learning in humans (Homo sapiens) and a nonhuman primate (Microcebus murinus). Journal of Comparative Psychology, 132(3), 315–325. https://doi.org/10.1037/com0000116

Schmitt, V., Federspiel, I., Eckert, J., Keupp, S., Tschernek, L., Faraut, L., Schuster, R., Michels, C., Sennhenn-Reulen, H., Bugnyar, T., Mussweiler, T., & Fischer, J. (2016). Do monkeys compare themselves to others? Animal Cognition, 19(2), 417–428. https://doi.org/10.1007/s10071-015-0943-4

Schmitt, V., Kröger, I., Zinner, D., Call, J., & Fischer, J. (2013). Monkeys perform as well as apes and humans in a size discrimination task. Animal Cognition, 16(5), 829–838. https://doi.org/10.1007/s10071-013-0616-0

Schneider, C., Call, J., & Liebal, K. (2012). What Role Do Mothers Play in the Gestural Acquisition of Bonobos (Pan paniscus) and Chimpanzees (Pan troglodytes)? International Journal of Primatology, 33(1), 246–262. https://doi.org/10.1007/s10764-011-9570-3

Schneider, C., Call, J., & Liebal, K. (2012). Onset and early use of gestural communication in nonhuman great apes. American Journal of Primatology, 74(2), 102–113. https://doi.org/10.1002/ajp.21011

Schnoell, A. V., Dittmann, M. T., & Fichtel, C. (2014). Human-introduced long-term traditions in wild redfronted lemurs? Animal Cognition, 17(1), 45–54. https://doi.org/10.1007/s10071-013-0636-9

Schnoell, A. V., & Fichtel, C. (2013). A novel feeding behaviour in wild redfronted lemurs (Eulemur rufifrons): Depletion of spider nests. Primates, 54(4), 371–375. https://doi.org/10.1007/s10329-013-0356-4

Schnoell, A. V., & Fichtel, C. (2012). Wild redfronted lemurs (Eulemur rufifrons) use social information to learn new foraging techniques. Animal Cognition, 15(4), 505–516. https://doi.org/10.1007/s10071-012-0477-y

Schrauf, C., & Call, J. (2011). Great apes use weight as a cue to find hidden food. American Journal of Primatology, 73(4), 323–334. https://doi.org/10.1002/ajp.20899

Schrauf, C., & Call, J. (2009). Great apes’ performance in discriminating weight and achromatic color. Animal Cognition, 12(4), 567–574. https://doi.org/10.1007/s10071-009-0216-1

Schrauf, C., Huber, L., & Visalberghi, E. (2008). Do capuchin monkeys use weight to select hammer tools? Animal Cognition, 11(3), 413–422. https://doi.org/10.1007/s10071-007-0131-2

Schreier, A. L., & Grove, M. (2014). Recurrent patterning in the daily foraging routes of hamadryas baboons (Papio hamadryas): Spatial memory in large-scale versus small-scale space. American Journal of Primatology, 76(5), 421–435. https://doi.org/10.1002/ajp.22192

Schroepfer-Walker, K., Wobber, V., & Hare, B. (2015). Experimental evidence that grooming and play are social currency in bonobos and chimpanzees. Behaviour, 152(3–4), 545–562. https://doi.org/10.1163/1568539X-00003258

Schubiger, M. N., Kissling, A., & Burkart, J. M. (2016). How task format affects cognitive performance: a memory test with two species of New World monkeys. Animal Behaviour, 121, 33–39. https://doi.org/10.1016/j.anbehav.2016.08.005

Schuppli, C., Meulman, E. J. M., Forss, S. I. F., Aprilinayati, F., van Noordwijk, M. A., & van Schaik, C. P. (2016). Observational social learning and socially induced practice of routine skills in immature wild orang-utans. Animal Behaviour, 119, 87–98. https://doi.org/10.1016/j.anbehav.2016.06.014

Schwartz, J. W., Engelberg, J. W. M., & Gouzoules, H. (2020). Evolving views on cognition in animal vocal communication: Contributions from scream research. Animal Behavior and Cognition, 7(2), 192–213. https://doi.org/10.26451/abc.07.02.12.2020

Schweinfurth, M. K., DeTroy, S. E., van Leeuwen, E. J. C., Call, J., & Haun, D. B. M. (2018). Spontaneous social tool use in Chimpanzees (Pan troglodytes). Journal of Comparative Psychology, 132(4), 455–463. https://doi.org/10.1037/com0000127

Schweitzer, C., Bec, P., & Blois-Heulin, C. (2007). Does the complexity of the task influence manual laterality in De Brazza’s monkeys (Cercopithecus neglectus)? Ethology, 113(10), 983–994. https://doi.org/10.1111/j.1439-0310.2007.01405.x

Scopa, C., & Palagi, E. (2016). Mimic me while playing! social tolerance and rapid facial mimicry in macaques (macaca tonkeana and macaca fuscata). Journal of Comparative Psychology, 130(2), 153–161. https://doi.org/10.1037/com0000028

Scott, N. M. (2013). Gesture Use by Chimpanzees (Pan troglodytes): Differences Between Sexes in Inter- and Intra-Sexual Interactions. American Journal of Primatology, 75(6), 555–567. https://doi.org/10.1002/ajp.22133

Seed, A. M., Call, J., Emery, N. J., & Clayton, N. S. (2009). Chimpanzees Solve the Trap Problem When the Confound of Tool-Use is Removed. Journal of Experimental Psychology: Animal Behavior Processes, 35(1), 23–34. https://doi.org/10.1037/a0012925

Shaffer, C. A. (2014). Spatial foraging in free ranging bearded sakis: Traveling salesmen or Lévy walkers? American Journal of Primatology, 76(5), 472–484. https://doi.org/10.1002/ajp.22227

Shepherd, S. V., & Platt, M. L. (2008). Spontaneous social orienting and gaze following in ringtailed lemurs (Lemur catta). Animal Cognition, 11(1), 13–20. https://doi.org/10.1007/s10071-007-0083-6

Shorland, G., Genty, E., Guéry, J. P., & Zuberbühler, K. (2019). Social learning of arbitrary food preferences in bonobos. Behavioural Processes, 167, 103912. https://doi.org/10.1016/j.beproc.2019.103912

Silberberg, A., Crescimbene, L., Addessi, E., Anderson, J. R., & Visalberghi, E. (2009). Does inequity aversion depend on a frustration effect? A test with capuchin monkeys (Cebus apella). Animal Cognition, 12(3), 505–509. https://doi.org/10.1007/s10071-009-0211-6

Silk, J. B., Brosnan, S. F., Henrich, J., Lambeth, S. P., & Shapiro, S. (2013). Chimpanzees share food for many reasons: The role of kinship, reciprocity, social bonds and harassment on food transfers. Animal Behaviour, 85(5), 941–947. https://doi.org/10.1016/j.anbehav.2013.02.014

Sirianni, G., Mundry, R., & Boesch, C. (2015). When to choose which tool: Multidimensional and conditional selection of nut-cracking hammers in wild chimpanzees. Animal Behaviour, 100, 152–165. https://doi.org/10.1016/j.anbehav.2014.11.022

Sirianni, G., & Visalberghi, E. (2013). Wild Bearded Capuchins Process Cashew Nuts Without Contacting Caustic Compounds. American Journal of Primatology, 75(4), 387–393. https://doi.org/10.1002/ajp.22119

Sirianni, G., Wittig, R. M., Gratton, P., Mundry, R., Schüler, A., & Boesch, C. (2018). Do chimpanzees anticipate an object’s weight? A field experiment on the kinematics of hammer-lifting movements in the nut-cracking Taï chimpanzees. Animal Cognition, 21(1), 109–118. https://doi.org/10.1007/s10071-017-1144-0

Skerry, A. E., Sheskin, M., & Santos, L. R. (2011). Capuchin monkeys are not prosocial in an instrumental helping task. Animal Cognition, 14(5), 647–654. https://doi.org/10.1007/s10071-011-0399-0

Slocombe, K. E., & Zuberbühler, K. (2006). Food-associated calls in chimpanzees: responses to food types or food preferences? Animal Behaviour, 72(5), 989–999. https://doi.org/10.1016/j.anbehav.2006.01.030

Slocombe, K. E., Townsend, S. W., & Zuberbühler, K. (2009). Wild chimpanzees (Pan troglodytes schweinfurthii) distinguish between different scream types: Evidence from a playback study. Animal Cognition, 12(3), 441–449. https://doi.org/10.1007/s10071-008-0204-x

Smith, J. D., Beran, M. J., Crossley, M. J., Boomer, J., & Ashby, F. G. (2010). Implicit and Explicit Category Learning by Macaques (Macaca mulatta) and Humans (Homo sapiens). Journal of Experimental Psychology: Animal Behavior Processes, 36(1), 54–65. https://doi.org/10.1037/a0015892

Smith, J. D., Chapman, W. P., & Redford, J. S. (2010). Stages of Category Learning in Monkeys (Macaca mulatta) and Humans (Homo sapiens). Journal of Experimental Psychology: Animal Behavior Processes, 36(1), 39–53. https://doi.org/10.1037/a0016573

Smith, J. D., Coutinho, M. V. C., & Couchman, J. J. (2011). The Learning of Exclusive-Or Categories by Monkeys (Macaca mulatta) and Humans (Homo sapiens). Journal of Experimental Psychology: Animal Behavior Processes, 37(1), 20–29. https://doi.org/10.1037/a0019497

Smith, J. D., Crossley, M. J., Boomer, J., Church, B. A., Beran, M. J., & Ashby, F. G. (2012). Implicit and explicit category learning by capuchin monkeys (Cebus apella). Journal of Comparative Psychology, 126(3), 294–304. https://doi.org/10.1037/a0026031

Smith, J. D., Jackson, B. N., & Church, B. A. (2020). Monkeys (Macaca mulatta) learn two-choice discriminations under displaced reinforcement. Journal of Comparative Psychology, 134(4), 423–434. https://doi.org/10.1037/com0000227

Smith, J. D., Redford, J. S., Beran, M. J., & Washburn, D. A. (2010). Rhesus monkeys (Macaca mulatta) adaptively monitor uncertainty while multi-tasking. Animal Cognition, 13(1), 93–101. https://doi.org/10.1007/s10071-009-0249-5

Smith, J. D., Redford, J. S., Haas, S. M., Coutinho, M. V. C., & Couchman, J. J. (2008). The comparative psychology of same-different judgments by humans (Homo sapiens) and monkeys (Macaca mulatta). Journal of Experimental Psychology. Animal Behavior Processes, 34(3), 361–374. https://doi.org/10.1037/0097-7403.34.3.361

Smith, M. F., Leverett, K. L., Wilson, B. J., & Brosnan, S. F. (2019). Capuchin monkeys (Sapajus [Cebus] apella) play Nash equilibria in dynamic games, but their decisions are likely not influenced by oxytocin. American Journal of Primatology, 81(4), 22973. https://doi.org/10.1002/ajp.22973

Snowdon, C. T., & Roskos, T. R. (2017). Stick-weaving: Innovative behavior in tamarins (Saguinus oedipus). Journal of Comparative Psychology, 131(2), 174–178. https://doi.org/10.1037/com0000071

Sonnweber, R., Ravignani, A., & Fitch, W. T. (2015). Non-adjacent visual dependency learning in chimpanzees. Animal Cognition, 18(3), 733–745. https://doi.org/10.1007/s10071-015-0840-x

Sousa, C., Biro, D., & Matsuzawa, T. (2009). Leaf-tool use for drinking water by wild chimpanzees (Pan troglodytes): Acquisition patterns and handedness. Animal Cognition, 12(1 SUPPL), 115–125. https://doi.org/10.1007/s10071-009-0278-0

Southgate, V., & Gomez, J. C. (2006). Searching beneath the shelf in macaque monkeys: Evidence for a gravity bias or a foraging bias? Journal of Comparative Psychology, 120(3), 314–321. https://doi.org/10.1037/0735-7036.120.3.314

Spagnoletti, N., Visalberghi, E., Verderane, M. P., Ottoni, E., Izar, P., & Fragaszy, D. (2012). Stone tool use in wild bearded capuchin monkeys, Cebus libidinosus. Is it a strategy to overcome food scarcity? Animal Behaviour, 83(5), 1285–1294. https://doi.org/10.1016/j.anbehav.2012.03.002

Sperber, A. L., Werner, L. M., Kappeler, P. M., & Fichtel, C. (2017). Grunt to go—Vocal coordination of group movements in redfronted lemurs. Ethology, 123(12), 894–905. https://doi.org/10.1111/eth.12663

Spillmann, B., Dunkel, L. P., van Noordwijk, M. A., Amda, R. N. A., Lameira, A. R., Wich, S. A., & van Schaik, C. P. (2010). Acoustic properties of long calls given by flanged male orang-utans (Pongo pygmaeus wurmbii) reflect both individual identity and context. Ethology, 116(5), 385–395. https://doi.org/10.1111/j.1439-0310.2010.01744.x

Spinozzi, G., De Lillo, C., Truppa, V., & Castorina, G. (2009). The Relative Use of Proximity, Shape Similarity, and Orientation as Visual Perceptual Grouping Cues in Tufted Capuchin Monkeys (Cebus apella) and Humans (Homo sapiens). Journal of Comparative Psychology, 123(1), 56–68. https://doi.org/10.1037/a0012674

Sproul, C., Palleroni, A., & Hauser, M. D. (2006). Cottontop tamarin, Saguinus oedipus, alarm calls contain sufficient information for recognition of individual identity. Animal Behaviour, 72(6), 1379–1385. https://doi.org/10.1016/j.anbehav.2006.04.006

Stahlman, W. D., Roberts, S., & Blaisdell, A. P. (2010). Effect of Reward Probability on Spatial and Temporal Variation. Journal of Experimental Psychology: Animal Behavior Processes, 36(1), 77–91. https://doi.org/10.1037/a0015971

Sterck, E. H. M., Olesen, C. U., & Massen, J. J. M. (2015). No costly prosociality among related long-tailed macaques (Macaca fascicularis). Journal of Comparative Psychology, 129(3), 275–282. https://doi.org/10.1037/a0039180

Stevens, J. R. (2010). Donor payoffs and other-regarding preferences in cotton-top tamarins (Saguinus oedipus). Animal Cognition, 13(4), 663–670. https://doi.org/10.1007/s10071-010-0309-x

Stevens, J. R., & Mühlhoff, N. (2012). Intertemporal choice in lemurs. Behavioural Processes, 89(2), 121–127. https://doi.org/10.1016/j.beproc.2011.10.002

Stevens, J. R., Wood, J. N., & Hauser, M. D. (2007). When quantity trumps number: Discrimination experiments in cotton-top tamarins (Saguinus oedipus) and common marmosets (Callithrix jacchus). Animal Cognition, 10(4), 429–437. https://doi.org/10.1007/s10071-007-0081-8

Stewart, F. A., & Piel, A. K. (2014). Termite fishing by wild chimpanzees: New data from Ugalla, western Tanzania. Primates, 55(1), 35–40. https://doi.org/10.1007/s10329-013-0362-6

Stewart, F. A., Piel, A. K., & O’ Malley, R. C. (2012). Responses of chimpanzees to a recently dead community member at Gombe National Park, Tanzania. In American Journal of Primatology (Vol. 74, Issue 1, pp. 1–7). John Wiley & Sons, Ltd. https://doi.org/10.1002/ajp.20994

Suarez, S. A. (2014). Ecological factors predictive of wild spider monkey (Ateles belzebuth) foraging decisions in Yasuní, Ecuador. American Journal of Primatology, 76(12), 1185–1195. https://doi.org/10.1002/ajp.22303

Suarez, S. A., Karro, J., Kiper, J., Farler, D., Mcelroy, B., Rogers, B. C., Stockwell, B., & Young, T. (2014). A comparison of computer-generated and naturally occurring foraging patterns in route-network-constrained spider monkeys. American Journal of Primatology, 76(5), 460–471. https://doi.org/10.1002/ajp.22222

Subiaul, F., Vonk, J., Okamoto-Barth, S., & Barth, J. (2008). Do chimpanzees learn reputation by observation? Evidence from direct and indirect experience with generous and selfish strangers. Animal Cognition, 11(4), 611–623. https://doi.org/10.1007/s10071-008-0151-6

Suchak, M., Watzek, J., Quarles, L. F., & de Waal, F. B. M. (2018). Novice chimpanzees cooperate successfully in the presence of experts, but may have limited understanding of the task. Animal Cognition, 21(1), 87–98. https://doi.org/10.1007/s10071-017-1142-2

Suda-King, C. (2008). Do orangutans (Pongo pygmaeus) know when they do not remember? Animal Cognition, 11(1), 21–42. https://doi.org/10.1007/s10071-007-0082-7

Suda-King, C., Bania, A. E., Stromberg, E. E., & Subiaul, F. (2013). Gorillas’ use of the escape response in object choice memory tests. Animal Cognition, 16(1), 65–84. https://doi.org/10.1007/s10071-012-0551-5

Sueur, C., & Petit, O. (2008). Shared or unshared consensus decision in macaques? Behavioural Processes, 78(1), 84–92. https://doi.org/10.1016/j.beproc.2008.01.004

Sueur, C., Petit, O., & Deneubourg, J. L. (2009). Selective mimetism at departure in collective movements of Macaca tonkeana: an experimental and theoretical approach. Animal Behaviour, 78(5), 1087–1095. https://doi.org/10.1016/j.anbehav.2009.07.029

Sueur, C., & Petit, O. (2010). Signals use by leaders in Macaca tonkeana and Macaca mulatta: Group-mate recruitment and behaviour monitoring. Animal Cognition, 13(2), 239–248. https://doi.org/10.1007/s10071-009-0261-9

Sugiura, H. (2007). Adjustment of temporal call usage during vocal exchange coo calls in Japanese macaques. Ethology, 113(6), 528–533. https://doi.org/10.1111/j.1439-0310.2007.01349.x

Surbeck, M., & Hohmann, G. (2015). Social preferences influence the short-term exchange of social grooming among male bonobos. Animal Cognition, 18(2), 573–579. https://doi.org/10.1007/s10071-014-0826-0

Taglialatela, J. P., Russell, J. L., Pope, S. M., Morton, T., Bogart, S., Reamer, L. A., Schapiro, S. J., & Hopkins, W. D. (2015). Multimodal communication in chimpanzees. American Journal of Primatology, 77(11), 1143–1148. https://doi.org/10.1002/ajp.22449

Takagi, S., & Fujita, K. (2018). Do capuchin monkeys (Sapajus apella) know the contents of memory traces?: A study of metamemory for compound stimuli. Journal of Comparative Psychology, 132(1), 88–96. https://doi.org/10.1037/com0000087

Takimoto, A., & Fujita, K. (2011). I acknowledge your help: Capuchin monkeys’ sensitivity to others’ labor. Animal Cognition, 14(5), 715–725. https://doi.org/10.1007/s10071-011-0406-5

Takimoto, A., Kuroshima, H., & Fujita, K. (2010). Capuchin monkeys (Cebus apella) are sensitive to others’ reward: An experimental analysis of food-choice for conspecifics. Animal Cognition, 13(2), 249–261. https://doi.org/10.1007/s10071-009-0262-8

Talbot, C. F., Leverett, K. L., & Brosnan, S. F. (2016). Capuchins recognize familiar faces. Animal Behaviour, 122, 37–45. https://doi.org/10.1016/j.anbehav.2016.09.017

Talbot, C. F., Parrish, A. E., Watzek, J., Essler, J. L., Leverett, K. L., Paukner, A., & Brosnan, S. F. (2018). The influence of reward quality and quantity and spatial proximity on the responses to inequity and contrast in capuchin monkeys (Cebus [Sapajus] apella). Journal of Comparative Psychology, 132(1), 75–87. https://doi.org/10.1037/com0000088

Talebi, M. G., Beltrão-Mendes, R., & Lee, P. C. (2009). Intra-Community coalitionary lethal attack of an adult male southern muriqui (Brachyteles arachnoides). American Journal of Primatology, 71(10), 860–867. https://doi.org/10.1002/ajp.20713

Tan, A. W. Y. (2017). From play to proficiency: The ontogeny of stone-tool use in coastal-foraging long-tailed macaques (Macaca fascicularis) from a comparative perception-action perspective. Journal of Comparative Psychology, 131(2), 89–114. https://doi.org/10.1037/com0000068

Tan, A. W. Y., Hemelrijk, C. K., Malaivijitnond, S., & Gumert, M. D. (2018). Young macaques (Macaca fascicularis) preferentially bias attention towards closer, older, and better tool users. Animal Cognition, 21(4), 551–563. https://doi.org/10.1007/s10071-018-1188-9

Tan, A. W. Y., Luncz, L., Haslam, M., Malaivijitnond, S., & Gumert, M. D. (2016). Complex processing of prickly pear cactus (Opuntia sp.) by free-ranging long-tailed macaques: Preliminary analysis for hierarchical organisation. Primates, 57(2), 141–147. https://doi.org/10.1007/s10329-016-0525-3

Tan, J., Kwetuenda, S., & Hare, B. (2015). Preference or paradigm? Bonobos show no evidence of other-regard in the standard prosocial choice task. Behaviour, 152(3–4), 521–544. https://doi.org/10.1163/1568539X-00003230

Tan, J., Tao, R., & Su, Y. (2014). Testing the Cognition of the Forgotten Colobines: A First Look at Golden Snub-Nosed Monkeys (Rhinopithecus roxellana). International Journal of Primatology, 35(2), 376–393. https://doi.org/10.1007/s10764-013-9741-5

Tanaka, M. (2007). Recognition of pictorial representations by chimpanzees (Pan troglodytes). Animal Cognition, 10(2), 169–179. https://doi.org/10.1007/s10071-006-0056-1

Tanaka, M., & Yamamoto, S. (2009). Token transfer between mother and offspring chimpanzees (Pan troglodytes): Mother-offspring interaction in a competitive situation. Animal Cognition, 12(1 SUPPL), 19–26. https://doi.org/10.1007/s10071-009-0270-8

Tanner, J. E., & Byrne, R. W. (2010). Triadic and collaborative play by gorillas in social games with objects. Animal Cognition, 13(4), 591–607. https://doi.org/10.1007/s10071-009-0308-y

Tarnaud, L., & Yamagiwa, J. (2008). Age-dependent patterns of intensive observation on elders by free-ranging juvenile Japanese macaques (Macaca fuscata yakui) within foraging context on Yakushima. American Journal of Primatology, 70(12), 1103–1113. https://doi.org/10.1002/ajp.20603

Taubert, J. (2010). Evidence of Human-like, Holistic Face Processing in Spider Monkeys (Ateles geoffroyi). Journal of Comparative Psychology, 124(1), 57–65. https://doi.org/10.1037/a0017704

Taubert, J., & Parr, L. A. (2011). Geometric distortions affect face recognition in chimpanzees (Pan troglodytes) and monkeys (Macaca mulatta). Animal Cognition, 14(1), 35–43. https://doi.org/10.1007/s10071-010-0341-x

Taubert, J., Qureshi, A. A., & Parr, L. A. (2012). The composite face effect in chimpanzees (Pan troglodytes) and rhesus monkeys (Macaca mulatta). Journal of Comparative Psychology, 126(4), 339–346. https://doi.org/10.1037/a0027287

Taubert, J., Weldon, K. B., & Parr, L. A. (2017). Robust representations of individual faces in chimpanzees (Pan troglodytes) but not monkeys (Macaca mulatta). Animal Cognition, 20(2), 321–329. https://doi.org/10.1007/s10071-016-1054-6

Taylor, H. K., Cunningham, C. L., & Hardie, S. (2020). Do lemurs know when they could be wrong? An investigation of information seeking in three species of lemur (Lemur catta, Eulemur rubriventer, and Varecia variegata). Journal of Comparative Psychology, 134(2), 241–251. https://doi.org/10.1037/com0000211

Tecwyn, E. C., Denison, S., Messer, E. J. E., & Buchsbaum, D. (2017). Intuitive probabilistic inference in capuchin monkeys. Animal Cognition, 20(2), 243–256. https://doi.org/10.1007/s10071-016-1043-9

Tecwyn, E. C., Thorpe, S. K. S., & Chappell, J. (2013). A novel test of planning ability: Great apes can plan step-by-step but not in advance of action. Behavioural Processes, 100, 174–184. https://doi.org/10.1016/j.beproc.2013.09.016

Tecwyn, E. C., Thorpe, S. K. S., & Chappell, J. (2012). What cognitive strategies do orangutans (Pongo pygmaeus) use to solve a trial-unique puzzle-tube task incorporating multiple obstacles? Animal Cognition, 15(1), 121–133. https://doi.org/10.1007/s10071-011-0438-x

Teichroeb, J. A., & Aguado, W. D. (2016). Foraging vervet monkeys optimize travel distance when alone but prioritize high-reward food sites when in competition. Animal Behaviour, 115, 1–10. https://doi.org/10.1016/j.anbehav.2016.02.020

Teichroeb, J. A., & Chapman, C. A. (2014). Sensory information and associative cues used in food detection by wild vervet monkeys. Animal Cognition, 17(3), 517–528. https://doi.org/10.1007/s10071-013-0683-2

Teichroeb, J. A., & Vining, A. Q. (2019). Navigation strategies in three nocturnal lemur species: diet predicts heuristic use and degree of exploratory behavior. Animal Cognition, 22(3), 343–354. https://doi.org/10.1007/s10071-019-01247-4

Tempelmann, S., Kaminski, J., & Liebal, K. (2011). Focus on the essential: All great apes know when others are being attentive. Animal Cognition, 14(3), 433–439. https://doi.org/10.1007/s10071-011-0378-5

Templer, V. L., & Hampton, R. R. (2012). Rhesus monkeys (Macaca mulatta) show robust evidence for memory awareness across multiple generalization tests. Animal Cognition, 15(3), 409–419. https://doi.org/10.1007/s10071-011-0468-4

Tennie, C., Call, J., & Tomasello, M. (2006). Push or pull: Imitation vs. emulation in great apes and human children. Ethology, 112(12), 1159–1169. https://doi.org/10.1111/j.1439-0310.2006.01269.x

Tennie, C., Greve, K., Gretscher, H., & Call, J. (2010). Two-year-old children copy more reliably and more often than nonhuman great apes in multiple observational learning tasks. Primates, 51(4), 337–351. https://doi.org/10.1007/s10329-010-0208-4

Tennie, C., Hedwig, D., Call, J., & Tomasello, M. (2008). An experimental study of nettle feeding in captive gorillas. American Journal of Primatology, 70(6), 584–593. https://doi.org/10.1002/ajp.20532

Tennie, C., Völter, C. J., Vonau, V., Hanus, D., Call, J., & Tomasello, M. (2019). Chimpanzees use observed temporal directionality to learn novel causal relations. Primates, 60(6), 517–524. https://doi.org/10.1007/s10329-019-00754-9

Thompson, C. L., Blanck, L. M., Pearson, M., Scheidel, C., & Vinyard, C. J. (2018). Do Common Marmosets (Callithrix jacchus) Use Scent to Communicate Information about Food Resources? Folia Primatologica, 89(5), 305–315. https://doi.org/10.1159/000490702

Thompson, R. K. R., Flemming, T. M., & Hagmann, C. E. (2016). Can old-world and new-world monkeys judge spatial above/below relations to be the same or different? Some of them, but not all of them. Behavioural Processes, 123, 74–83. https://doi.org/10.1016/j.beproc.2015.11.010

Thunström, M., Kuchenbuch, P., & Young, C. (2014). Concealing of facial expressions by a wild Barbary macaque (Macaca sylvanus). Primates, 55(3), 369–375. https://doi.org/10.1007/s10329-014-0423-5

Tia, B., Viaro, R., & Fadiga, L. (2018). Tool-use training temporarily enhances cognitive performance in long-tailed macaques (Macaca fascicularis). Animal Cognition, 21(3), 365–378. https://doi.org/10.1007/s10071-018-1173-3

Toader, A. C., Rao, H. M., Ryoo, M., Bohlen, M. O., Cruger, J. S., OhDescher, H., Ferrari, S., Egner, T., Beck, J., & Sommer, M. A. (2019). Probabilistic Inferential Decision-Making Under Time Pressure in Rhesus Macaques (Macaca mulatta). Journal of Comparative Psychology, 133(3), 380. https://doi.org/10.1037/com0000168

Tombak, K. J., Wikberg, E. C., Rubenstein, D. I., & Chapman, C. A. (2019). Reciprocity and rotating social advantage among females in egalitarian primate societies. Animal Behaviour, 157, 189–200. https://doi.org/10.1016/j.anbehav.2019.09.010

Tomonaga, M. (2008). Relative numerosity discrimination by chimpanzees (Pan troglodytes): Evidence for approximate numerical representations. Animal Cognition, 11(1), 43–57. https://doi.org/10.1007/s10071-007-0089-0

Tomonaga, M. (2007). Visual search for orientation of faces by a chimpanzee (Pan troglodytes): Face-specific upright superiority and the role of facial configural properties. Primates, 48(1), 1–12. https://doi.org/10.1007/s10329-006-0011-4

Tomonaga, M. (2007). Is chimpanzee (Pan troglodytes) spatial attention reflexively triggered by gaze cue? Journal of Comparative Psychology, 121(2), 156–170. https://doi.org/10.1037/0735-7036.121.2.156

Tomonaga, M., & Imura, T. (2009). Human gestures trigger different attentional shifts in chimpanzees (Pan troglodytes) and humans (Homo sapiens). Animal Cognition, 12(1 SUPPL), 11. https://doi.org/10.1007/s10071-009-0268-2

Torralvo, K., Rabelo, R. M., Andrade, A., & Botero-Arias, R. (2017). Tool use by Amazonian capuchin monkeys during predation on caiman nests in a high-productivity forest. Primates, 58(2), 279–283. https://doi.org/10.1007/s10329-017-0603-1

Tournier, E., Tournier, V., Van de Waal, E., Barrett, A., Brown, L., & Bshary, R. (2014). Differences in diet between six neighbouring groups of vervet monkeys. Ethology, 120(5), 471–482. https://doi.org/10.1111/eth.12218

Trébouet, F., Reichard, U. H., Pinkaew, N., & Malaivijitnond, S. (2018). Extractive foraging of toxic caterpillars in wild northern pig-tailed macaques (Macaca leonina). Primates, 59(2), 185–196. https://doi.org/10.1007/s10329-017-0638-3

Treichler, F. R., & Raghanti, M. A. (2010). Serial list combination by monkeys (Macaca mulatta): Test cues and linking. Animal Cognition, 13(1), 121–131. https://doi.org/10.1007/s10071-009-0251-y

Treichler, F. R., Raghanti, M. A., & Van Tilburg, D. N. (2007). Serial List Linking by Macaque Monkeys (Macaca mulatta): List Property Limitations. Journal of Comparative Psychology, 121(3), 250–259. https://doi.org/10.1037/0735-7036.121.3.250

Troisi, C. A., Hoppitt, W. J. E., Ruiz-Miranda, C. R., & Laland, K. N. (2018). Food-Offering Calls in Wild Golden Lion Tamarins (Leontopithecus rosalia): Evidence for Teaching Behavior? International Journal of Primatology, 39(6), 1105–1123. https://doi.org/10.1007/s10764-018-0069-z

Truppa, V., De Simone, D. A., & Lillo, C. De. (2016). Short-term memory effects on visual global/local processing in tufted capuchin monkeys (sapajus spp.). Journal of Comparative Psychology, 130(2), 162–173. https://doi.org/10.1037/com0000018

Truppa, V., Garofoli, D., Castorina, G., Mortari, E. P., Natale, F., & Visalberghi, E. (2010). Identity concept learning in matching-to-sample tasks by tufted capuchin monkeys (Cebus apella). Animal Cognition, 13(6), 835–848. https://doi.org/10.1007/s10071-010-0332-y

Truppa, V., Spinozzi, G., Stegagno, T., & Fagot, J. (2009). Picture processing in tufted capuchin monkeys (Cebus apella). Behavioural Processes, 82(2), 140–152. https://doi.org/10.1016/j.beproc.2009.05.004

Tsutsumi, S., Ushitani, T., Tomonaga, M., & Fujita, K. (2012). Infant monkeys’ concept of animacy: The role of eyes and fluffiness. Primates, 53(2), 113–119. https://doi.org/10.1007/s10329-011-0289-8

Tu, H. W., & Hampton, R. R. (2014). Control of working memory in rhesus monkeys (Macaca mulatta). Journal of Experimental Psychology. Animal Learning and Cognition, 40(4), 467–476. https://doi.org/10.1037/xan0000030

Tu, H. W., & Hampton, R. R. (2013). One-trial memory and habit contribute independently to matching-to-sample performance in rhesus monkeys (macaca mulatta). Journal of Comparative Psychology, 127(3), 319–328. https://doi.org/10.1037/a0030496

Tu, H. W., Pani, A. A., & Hampton, R. R. (2015). Rhesus monkeys (Macaca mulatta) adaptively adjust information seeking in response to information accumulated. Journal of Comparative Psychology, 129(4), 347–355. https://doi.org/10.1037/a0039595

Tujague, M. P., & Janson, C. H. (2017). Wild capuchin monkeys anticipate the amount of ripe fruit in natural trees. Animal Cognition, 20(5), 841–853. https://doi.org/10.1007/s10071-017-1105-7

Tujague, M. P., Janson, C. H., & Lahitte, H. B. (2015). Long-Term Spatial Memory and Learning Set Formation in Captive Capuchin Monkeys (Cebus libidinosus = Sapajus cay). International Journal of Primatology, 36(6), 1067–1085. https://doi.org/10.1007/s10764-015-9878-5

Ueno, M., Yamada, K., & Nakamichi, M. (2014). The effect of solicitations on grooming exchanges among female Japanese macaques at Katsuyama. Primates, 55(1), 81–87. https://doi.org/10.1007/s10329-013-0371-5

Uher, J., & Call, J. (2008). How the Great Apes (Pan troglodytes, Pongo pygmaeus, Pan paniscus, Gorilla gorilla) Perform on the Reversed Reward Contingency Task II: Transfer to New Quantities, Long-Term Retention, and the Impact of Quantity Ratios. Journal of Comparative Psychology, 122(2), 204–212. https://doi.org/10.1037/0735-7036.122.2.204

Vale, G. L., Flynn, E. G., Lambeth, S. P., Schapiro, S. J., & Kendal, R. L. (2014). Public information use in chimpanzees (Pan troglodytes) and children (Homo sapiens). Journal of Comparative Psychology, 128(2), 215–223. https://doi.org/10.1037/a0034420

Vale, G. L., Flynn, E. G., Pender, L., Price, E., Whiten, A., Lambeth, S. P., Schapiro, S. J., & Kendal, R. L. (2016). Robust retention and transfer of tool construction techniques in chimpanzees (Pan troglodytes). Journal of Comparative Psychology, 130(1), 24–35. https://doi.org/10.1037/a0040000

Vale, G. L., Davis, S. J., van de Waal, E., Schapiro, S. J., Lambeth, S. P., & Whiten, A. (2017). Lack of conformity to new local dietary preferences in migrating captive chimpanzees. Animal Behaviour, 124, 135–144. https://doi.org/10.1016/j.anbehav.2016.12.007

Vale, G. L., Williams, L. E., Schapiro, S. J., Lambeth, S. P., & Brosnan, S. F. (2019). Responses to Economic Games of Cooperation and Conflict in Squirrel Monkeys (Saimiri boliviensis). Animal Behavior and Cognition, 6(1), 32–47. https://doi.org/10.26451/abc.06.01.03.2019

Valero, A., & Byrne, R. W. (2007). Spider monkey ranging patterns in Mexican subtropical forest: Do travel routes reflect planning? Animal Cognition, 10(3), 305–315. https://doi.org/10.1007/s10071-006-0066-z

Van De Waal, E., Bshary, R., & Renevey, N. (2013). Philopatric vervet monkey females are the focus of social attention rather independently of rank. Behaviour, 150(6), 599–615. https://doi.org/10.1163/1568539X-00003072

Van De Waal, E., & Bshary, R. (2011). Social-learning abilities of wild vervet monkeys in a two-step task artificial fruit experiment. Animal Behaviour, 81(2), 433–438. https://doi.org/10.1016/j.anbehav.2010.11.013

Van de Waal, E., Bshary, R., & Whiten, A. (2014). Wild vervet monkey infants acquire the food-processing variants of their mothers. Animal Behaviour, 90, 41–45. https://doi.org/10.1016/j.anbehav.2014.01.015

van de Waal, E., Claidière, N., & Whiten, A. (2015). Wild vervet monkeys copy alternative methods for opening an artificial fruit. Animal Cognition, 18(3), 617–627. https://doi.org/10.1007/s10071-014-0830-4

van de Waal, E., Claidière, N., & Whiten, A. (2013). Social learning and spread of alternative means of opening an artificial fruit in four groups of vervet monkeys. Animal Behaviour, 85(1), 71–76. https://doi.org/10.1016/j.anbehav.2012.10.008

van Hooff, J. A. R. A. M., & Lukkenaar, B. (2015). Captive chimpanzee takes down a drone: tool use toward a flying object. Primates, 56(4), 289–292. https://doi.org/10.1007/s10329-015-0482-2

van Leeuwen, E. J. C., & Call, J. (2017). Conservatism and “copy-if-better” in chimpanzees (Pan troglodytes). Animal Cognition, 20(3), 575–579. https://doi.org/10.1007/s10071-016-1061-7

van Leeuwen, E. J. C., Cronin, K. A., & Haun, D. B. M. (2014). A group-specific arbitrary tradition in chimpanzees (Pan troglodytes). Animal Cognition, 17(6), 1421–1425. https://doi.org/10.1007/s10071-014-0766-8

van Leeuwen, E. J. C., Mulenga, I. C., & Chidester, D. L. (2014). Early social deprivation negatively affects social skill acquisition in chimpanzees (Pan troglodytes). Animal Cognition, 17(2), 407–414. https://doi.org/10.1007/s10071-013-0672-5

Van Schaik, C. P., Van Noordwijk, M. A., & Wich, S. A. (2006). Innovation in wild bornean orangutans (Pongo pygmaeus wurmbii). Behaviour, 143(7), 839–876. https://doi.org/10.1163/156853906778017944

Vandercone, R., Premachandra, K., Wijethunga, G. P., Dinadh, C., Ranawana, K., & Bahar, S. (2013). Random walk analysis of ranging patterns of sympatric langurs in a complex resource landscape. American Journal of Primatology, 75(12), 1209–1219. https://doi.org/10.1002/ajp.22183

VanMarle, K., Aw, J., McCrink, K., & Santos, L. R. (2006). How capuchin monkeys (Cebus apella) quantify objects and substances. Journal of Comparative Psychology, 120(4), 416–426. https://doi.org/10.1037/0735-7036.120.4.416

Ventricelli, M., Focaroli, V., De Petrillo, F., Macchitella, L., Paglieri, F., & Addessi, E. (2013). How capuchin monkeys (Cebus apella) behaviorally cope with increasing delay in a self-control task. Behavioural Processes, 100, 146–152. https://doi.org/10.1016/j.beproc.2013.09.001

Ventura, R., Majolo, B., Koyama, N. F., Hardie, S., & Schino, G. (2006). Reciprocation and interchange in wild Japanese macaques: Grooming, cofeeding, and agonistic support. American Journal of Primatology, 68(12), 1138–1149. https://doi.org/10.1002/ajp.20314

Versace, E., Endress, A. D., & Hauser, M. D. (2008). Pattern recognition mediates flexible timing of vocalizations in nonhuman primates: experiments with cottontop tamarins. Animal Behaviour, 76(6), 1885–1892. https://doi.org/10.1016/j.anbehav.2008.08.015

Versace, E., Rogge, J. R., Shelton-May, N., & Ravignani, A. (2019). Positional encoding in cotton-top tamarins (Saguinus oedipus). Animal Cognition, 22(5), 825–838. https://doi.org/10.1007/s10071-019-01277-y

Vick, S. J., Toxopeus, I., & Anderson, J. R. (2006). Pictorial gaze cues do not enhance long-tailed macaques’ performance on a computerised object-location task. Behavioural Processes, 73(3), 308–314. https://doi.org/10.1016/j.beproc.2006.07.003

Vining, A. Q., & Marsh, H. L. (2015). Information seeking in capuchins (Cebus apella): A rudimentary form of metacognition? Animal Cognition, 18(3), 667–681. https://doi.org/10.1007/s10071-015-0835-7

Visalberghi, E., Barca, V., Izar, P., Fragaszy, D., & Truppa, V. (2021). Optional tool use: The case of wild bearded capuchins (Sapajus libidinosus) cracking cashew nuts by biting or by using percussors. American Journal of Primatology, 83(1), 1–15. https://doi.org/10.1002/ajp.23221

Visalberghi, E., Di Bernardi, C., Marino, L. A., Fragaszy, D., & Izar, P. (2017). Female bearded capuchin monkeys (Sapajus libidinosus) use objects to solicit the sexual partner. Journal of Comparative Psychology, 131(3), 207–213. https://doi.org/10.1037/com0000072

Vlamings, P. H. J. M., Hare, B., & Call, J. (2010). Reaching around barriers: The performance of the great apes and 3-5-year-old children. Animal Cognition, 13(2), 273–285. https://doi.org/10.1007/s10071-009-0265-5

Voelkl, B., & Huber, L. (2007). Common marmosets (Callithrix jacchus) do not utilize social information in three simultaneous social foraging tasks. Animal Cognition, 10(2), 149–158. https://doi.org/10.1007/s10071-006-0053-4

Völter, C. J., & Call, J. (2018). Intuitive optics: what great apes infer from mirrors and shadows. Animal Cognition, 21(4), 493–512. https://doi.org/10.1007/s10071-018-1184-0

Völter, C. J., & Call, J. (2014). The cognitive underpinnings of flexible tool use in great apes. Journal of Experimental Psychology: Animal Behavior Processes, 40(3), 287–302. https://doi.org/10.1037/xan0000025

Völter, C. J., & Call, J. (2014). Great apes (pan paniscus, pan troglodytes, gorilla gorilla, pongo abelii) follow visual trails to locate hidden food. Journal of Comparative Psychology, 128(2), 199–208. https://doi.org/10.1037/a0035434

Völter, C. J., & Call, J. (2012). Problem solving in great apes (Pan paniscus, Pan troglodytes, Gorilla gorilla, and Pongo abelii): The effect of visual feedback. Animal Cognition, 15(5), 923–936. https://doi.org/10.1007/s10071-012-0519-5

Völter, C. J., Rossano, F., & Call, J. (2015). From exploitation to cooperation: Social tool use in orang-utan mother-offspring dyads. Animal Behaviour, 100, 126–134. https://doi.org/10.1016/j.anbehav.2014.11.025

Vonk, J. (2014). Quantity matching by an orangutan (Pongo abelii). Animal Cognition, 17(2), 297–306. https://doi.org/10.1007/s10071-013-0662-7

Vonk, J., & Hamilton, J. (2014). Orangutans (Pongo abelii) and a gorilla (Gorilla gorilla gorilla) match features in familiar and unfamiliar individuals. Animal Cognition, 17(5), 1089–1105. https://doi.org/10.1007/s10071-014-0741-4

Vonk, J., & Subiaul, F. (2009). Do chimpanzees know what others can and cannot do? Reasoning about “capability.” Animal Cognition, 12(2), 267–286. https://doi.org/10.1007/s10071-008-0189-5

Vonk, J., Torgerson-White, L., McGuire, M., Thueme, M., Thomas, J., & Beran, M. J. (2014). Quantity estimation and comparison in western lowland gorillas (Gorilla gorilla gorilla). Animal Cognition, 17(3), 755–765. https://doi.org/10.1007/s10071-013-0707-y

Waga, I. C., Dacier, A. K., Pinha, P. S., & Tavares, M. C. H. (2006). Spontaneous tool use by wild capuchin monkeys (Cebus libidinosus) in the Cerrado. Folia Primatologica, 77(5), 337–344. https://doi.org/10.1159/000093698

Wagner, K. E., Hopper, L. M., & Ross, S. R. (2016). Asymmetries in the production of self-directed behavior by chimpanzees and gorillas during a computerized cognitive test. Animal Cognition, 19(2), 343–350. https://doi.org/10.1007/s10071-015-0937-2

Wakita, M. (2020). Common marmosets (Callithrix jacchus) cannot recognize global configurations of sound patterns but can recognize adjacent relations of sounds. Behavioural Processes, 176, 104136. https://doi.org/10.1016/j.beproc.2020.104136

Wakita, M. (2019). Auditory sequence perception in common marmosets (Callithrix jacchus). Behavioural Processes, 162, 55–63. https://doi.org/10.1016/j.beproc.2019.01.014

Wakita, M. (2012). Monkeys perceive the orientation of objects relative to the vertical axis. Animal Cognition, 15(6), 1205–1209. https://doi.org/10.1007/s10071-012-0535-5

Wakita, M. (2008). Orientation perception in rhesus monkeys (Macaca mulatta). Animal Cognition, 11(3), 535–545. https://doi.org/10.1007/s10071-008-0146-3

Walkup, K. R., Shumaker, R. W., & Pruetz, J. D. (2010). Orangutans (Pongo spp.) may prefer tools with rigid properties to flimsy tools. Journal of Comparative Psychology, 124(4), 351–355. https://doi.org/10.1037/a0019566

Waller, B. M., Whitehouse, J., & Micheletta, J. (2016). Macaques can predict social outcomes from facial expressions. Animal Cognition, 19(5), 1031–1036. https://doi.org/10.1007/s10071-016-0992-3

Washburn, D. A., Smith, J. D., & Shields, W. E. (2006). Rhesus monkeys (Macaca mulatta) immediately generalize the uncertain response. Journal of Experimental Psychology: Animal Behavior Processes, 32(2), 185–189. https://doi.org/10.1037/0097-7403.32.2.185

Watanabe, K., Urasopon, N., & Malaivijitnond, S. (2007). Long-tailed macaques use human hair as dental floss. American Journal of Primatology, 69(8), 940–944. https://doi.org/10.1002/ajp.20403

Watson, C. F. I., & Caldwell, C. A. (2010). Neighbor effects in marmosets: Social contagion of agonism and affiliation in captive Callithrix jacchus. American Journal of Primatology, 72(6), 549–558. https://doi.org/10.1002/ajp.20805

Watson, K. K., Ghodasra, J. H., Furlong, M. A., & Platt, M. L. (2012). Visual preferences for sex and status in female rhesus macaques. Animal Cognition, 15(3), 401–407. https://doi.org/10.1007/s10071-011-0467-5

Watson, S. K., Lambeth, S. P., Schapiro, S. J., & Whiten, A. (2018). Chimpanzees prioritise social information over pre-existing behaviours in a group context but not in dyads. Animal Cognition, 21(3), 407–418. https://doi.org/10.1007/s10071-018-1178-y

Watts, D. P. (2020). Responses to dead and dying conspecifics and heterospecifics by wild mountain gorillas (Gorilla beringei beringei) and chimpanzees (Pan troglodytes schweinfurthii). Primates, 61(1), 55–68. https://doi.org/10.1007/s10329-019-00735-y

Watts, D. P. (2016). Production of grooming-associated sounds by chimpanzees (Pan troglodytes) at Ngogo: variation, social learning, and possible functions. Primates, 57(1), 61–72. https://doi.org/10.1007/s10329-015-0497-8

Watts, D. P. (2008). Tool use by chimpanzees at Ngogo, Kibale National Park, Uganda. International Journal of Primatology, 29(1), 83–94. https://doi.org/10.1007/s10764-007-9227-4

Webb, C. E., Franks, B., Romero, T., Higgins, E. T., & de Waal, F. B. M. (2014). Individual differences in chimpanzee reconciliation relate to social switching behaviour. Animal Behaviour, 90, 57–63. https://doi.org/10.1016/j.anbehav.2014.01.014

Weigel, E. A., & Berman, C. M. (2018). Body signals used during social play in captive immature western lowland gorillas. Primates, 59(3), 253–265. https://doi.org/10.1007/s10329-017-0646-3

Weiss, D. J., & Wark, J. (2009). Hysteresis Effects in a Motor Task With Cotton-Top Tamarins (Sanguinus oedipus). Journal of Experimental Psychology: Animal Behavior Processes, 35(3), 427–433. https://doi.org/10.1037/a0013964

Weldon, K. B., Taubert, J., Smith, C. L., & Parr, L. A. (2013). How the Thatcher illusion reveals evolutionary differences in the face processing of primates. Animal Cognition, 16(5), 691–700. https://doi.org/10.1007/s10071-013-0604-4

Wergård, E. M., Temrin, H., Forkman, B., Spångberg, M., Fredlund, H., & Westlund, K. (2015). Training pair-housed Rhesus macaques (Macaca mulatta) using a combination of negative and positive reinforcement. Behavioural Processes, 113, 51–59. https://doi.org/10.1016/j.beproc.2014.12.008

West, S., Jett, S. E., Beckman, T., & Vonk, J. (2010). The phylogenetic roots of cognitive dissonance. Journal of Comparative Psychology, 124(4), 425–432. https://doi.org/10.1037/a0019932

Westergaard, G. C., Evans, T. A., & Howell, S. (2007). Token mediated tool exchange between tufted capuchin monkeys (Cebus apella). Animal Cognition, 10(4), 407–414. https://doi.org/10.1007/s10071-007-0072-9

Wheeler, B. C. (2010). Decrease in alarm call response among tufted capuchins in competitive feeding contexts: Possible evidence for counterdeception. International Journal of Primatology, 31(4), 665–675. https://doi.org/10.1007/s10764-010-9419-1

Wheeler, B. C., Fahy, M., & Tiddi, B. (2019). Experimental evidence for heterospecific alarm signal recognition via associative learning in wild capuchin monkeys. Animal Cognition, 22(5), 687–695. https://doi.org/10.1007/s10071-019-01264-3

Wheeler, B. C., & Hammerschmidt, K. (2013). Proximate factors underpinning receiver responses to deceptive false alarm calls in wild tufted capuchin monkeys: Is it counterdeception? American Journal of Primatology, 75(7), 715–725. https://doi.org/10.1002/ajp.22097

Wheeler, B. C., Tiddi, B., & Heistermann, M. (2014). Competition-induced stress does not explain deceptive alarm calling in tufted capuchin monkeys. Animal Behaviour, 93, 49–58. https://doi.org/10.1016/j.anbehav.2014.04.016

Whitehouse, J., Micheletta, J., Kaminski, J., & Waller, B. M. (2016). Macaques attend to scratching in others. Animal Behaviour, 122, 169–175. https://doi.org/10.1016/j.anbehav.2016.10.020

Whitham, W., & Washburn, D. A. (2020). Strategy use in probabilistic categorization by rhesus macaques (Macaca mulatta) and capuchin monkeys (Cebus [Sapajus] apella). Journal of Comparative Psychology, 134(4), 379–390. https://doi.org/10.1037/com0000221

Wich, S. A., & Sterck, E. H. M. (2007). Familiarity and threat of opponents determine variation in Thomas langur (Presbytis thomasi) male behaviour during between-group encounters. Behaviour, 144(12), 1583–1598. https://doi.org/10.1163/156853907782512065

Wich, S. A., Swartz, K. B., Hardus, M. E., Lameira, A. R., Stromberg, E., & Shumaker, R. W. (2009). A case of spontaneous acquisition of a human sound by an orangutan. Primates, 50(1), 56–64. https://doi.org/10.1007/s10329-008-0117-y

Wilfried, E. E. G., & Yamagiwa, J. (2014). Use of tool sets by chimpanzees for multiple purposes in Moukalaba-Doudou National Park, Gabon. Primates, 55(4), 467–472. https://doi.org/10.1007/s10329-014-0431-5

Wilke, C., Kavanagh, E., Donnellan, E., Waller, B. M., Machanda, Z. P., & Slocombe, K. E. (2017). Production of and responses to unimodal and multimodal signals in wild chimpanzees, Pan troglodytes schweinfurthii. Animal Behaviour, 123, 305–316. https://doi.org/10.1016/j.anbehav.2016.10.024

Wilson, D. A., & Tomonaga, M. (2018). Visual discrimination of primate species based on faces in chimpanzees. Primates, 59(3), 243–251. https://doi.org/10.1007/s10329-018-0649-8

Wilson, M. L., Hauser, M. D., & Wrangham, R. W. (2007). Chimpanzees (Pan troglodytes) modify grouping and vocal behaviour in response to location-specific risk. Behaviour, 144(12), 1621–1653. https://doi.org/10.1163/156853907782512137

Wittiger, L., & Sunderland-Groves, J. L. (2007). Tool use during display behavior in wild Cross River gorillas. American Journal of Primatology, 69(11), 1307–1311. https://doi.org/10.1002/ajp.20436

Wobber, V., & Herrmann, E. (2015). The influence of testosterone on cognitive performance in bonobos and chimpanzees. Behaviour, 152(3–4), 407–423. https://doi.org/10.1163/1568539X-00003202

Wolovich, C. K., Feged, A., Evans, S., & Green, S. M. (2006). Social patterns of food sharing in monogamous owl monkeys. American Journal of Primatology, 68(7), 663–674. https://doi.org/10.1002/ajp.20238

Wombolt, J. R., & Caine, N. G. (2016). Patterns on serpentine shapes elicit visual attention in marmosets (Callithrix jacchus). American Journal of Primatology, 78(9), 928–936. https://doi.org/10.1002/ajp.22563

Wood, L. A., & Whiten, A. (2017). Visible spatial contiguity of social information and reward affects social learning in brown capuchins (Sapajus apella) and children (Homo sapiens). Journal of Comparative Psychology, 131(4), 304–316. https://doi.org/10.1037/com0000076

Workman, K. P., Healey, B., Carlotto, A., & Lacreuse, A. (2019). One-year change in cognitive flexibility and fine motor function in middle-aged male and female marmosets (Callithrix jacchus). American Journal of Primatology, 81(2), e22924. https://doi.org/10.1002/ajp.22924

Wright, A. A. (2010). Functional relationships for determining similarities and differences in comparative cognition. Behavioural Processes, 85(3), 246–251. https://doi.org/10.1016/j.beproc.2010.07.018

Wright, A. A., & Katz, J. S. (2006). Mechanisms of same/different concept learning in primates and avians. Behavioural Processes, 72(3), 234–254. https://doi.org/10.1016/j.beproc.2006.03.009

Wu, C. F., Liao, Z. J., Sueur, C., Sha, J. C. M., Zhang, J., & Zhang, P. (2018). The influence of kinship and dominance hierarchy on grooming partner choice in free-ranging Macaca mulatta brevicaudus. Primates, 59(4), 377–384. https://doi.org/10.1007/s10329-018-0662-y

Xia, D. P., Li, J. H., Garber, P. A., Matheson, M. D., Sun, B. H., & Zhu, Y. (2013). Grooming reciprocity in male Tibetan macaques. American Journal of Primatology, 75(10), 1009–1020. https://doi.org/10.1002/ajp.22165

Xia, D., Li, J., Garber, P. A., Sun, L., Zhu, Y., & Sun, B. (2012). Grooming Reciprocity in Female Tibetan Macaques Macaca Thibetana. American Journal of Primatology, 74(6), 569–579. https://doi.org/10.1002/ajp.21985

Xiang, Z. F., Yang, B. H., Yu, Y., Yao, H., Grueter, C. C., Garber, P. A., & Li, M. (2014). Males collectively defend their one-male units against bachelor males in a multi-level primate society. American Journal of Primatology, 76(7), 609–617. https://doi.org/10.1002/ajp.22254

Yamaguchi, C., & Izumi, A. (2006). Effect of others’ attentional states on vocalizations in Japanese monkeys (Macaca fuscata). Behavioural Processes, 73(3), 285–289. https://doi.org/10.1016/j.beproc.2006.07.002

Yamamoto, S. (2015). Non-reciprocal but peaceful fruit sharing in wild bonobos in Wamba. Behaviour, 152(3–4), 335–357. https://doi.org/10.1163/1568539X-00003257

Yamamoto, S., & Tanaka, M. (2010). The influence of kin relationship and reciprocal context on chimpanzees’ other-regarding preferences. Animal Behaviour, 79(3), 595–602. https://doi.org/10.1016/j.anbehav.2009.11.034

Yamamoto, S., & Tanaka, M. (2009). Selfish strategies develop in social problem situations in chimpanzee (Pan troglodytes) mother-infant pairs. Animal Cognition, 12(1 SUPPL), 27–36. https://doi.org/10.1007/s10071-009-0276-2

Yamamoto, S., Yamakoshi, G., Humle, T., & Matsuzawa, T. (2008). Invention and modification of a new tool use behavior: Ant-fishing in trees by a wild chimpanzee (Pan troglodytes verus) at Bossou, Guinea. American Journal of Primatology, 70(7), 699–702. https://doi.org/10.1002/ajp.20544

Yamanashi, Y., Bando, H., Matsunaga, M., Tanaka, M., Nogami, E., & Hirata, S. (2020). Development of bed-building behaviors in captive chimpanzees (Pan troglodytes): Implication for critical period hypothesis and captive management. Primates, 61(5), 639–646. https://doi.org/10.1007/s10329-020-00839-w

Yamazaki, Y., Iriki, A., & Watanabe, S. (2011). Modulation of physical understanding by common marmosets (Callithrix jacchus). Animal Cognition, 14(2), 175–186. https://doi.org/10.1007/s10071-010-0351-8

Yamazaki, Y., Saiki, M., Inada, M., Iriki, A., & Watanabe, S. (2014). Transposition and its generalization in common marmosets. Journal of Experimental Psychology: Animal Behavior Processes, 40(3), 317–326. https://doi.org/10.1037/xan0000027

Yocom, A. M., & Boysen, S. T. (2010). Capuchins (Cebus apella) can solve a means-end problem. Journal of Comparative Psychology, 124(3), 271–277. https://doi.org/10.1037/a0019369

Yu, L., & Tomonaga, M. (2016). Unidirectional adaptation in tempo in pairs of chimpanzees during simultaneous tapping movement: An examination under face-to-face setup. Primates, 57(2), 181–185. https://doi.org/10.1007/s10329-016-0512-8

Zakrzewski, A. C., Church, B. A., & David Smith, J. (2018). The transfer of category knowledge by Macaques (Macaca mulatta) and humans (Homo sapiens). Journal of Comparative Psychology, 132(1), 58–74. https://doi.org/10.1037/com0000095

Zakrzewski, A. C., Perdue, B. M., Beran, M. J., Church, B. A., & Smith, J. D. (2014). Cashing out: The decisional flexibility of uncertainty responses in rhesus macaques (Macaca mulatta) and humans (Homo sapiens). Journal of Experimental Psychology. Animal Learning and Cognition, 40(4), 490–501. https://doi.org/10.1037/xan0000041

Zander, S. L., & Judge, P. G. (2015). Brown capuchin monkeys (Sapajus apella) plan their movements on a grasping task. Journal of Comparative Psychology, 129(2), 181–188. https://doi.org/10.1037/a0038850

Zander, S. L., Weiss, D. J., & Judge, P. G. (2013). The interface between morphology and action planning: A comparison of two species of New World monkeys. Animal Behaviour, 86(6), 1251–1258. https://doi.org/10.1016/j.anbehav.2013.09.028

Zarei, S. A., Sheibani, V., & Mansouri, F. A. (2019). Interaction of music and emotional stimuli in modulating working memory in macaque monkeys. American Journal of Primatology, 81(7), 81 22999. https://doi.org/10.1002/ajp.22999

Zimmermann, F., Zemke, F., Call, J., & Gómez, J. C. (2009). Orangutans (Pongo pygmaeus) and bonobos (Pan paniscus) point to inform a human about the location of a tool. Animal Cognition, 12(2), 347–358. https://doi.org/10.1007/s10071-008-0194-8

Zürcher, Y., & Burkart, J. M. (2017). Evidence for Dialects in Three Captive Populations of Common Marmosets (Callithrix jacchus). International Journal of Primatology, 38(4), 780–793. https://doi.org/10.1007/s10764-017-9979-4
